# Bibliometric analysis study on cognitive function in developmental coordination disorder from 2010 to 2022

**DOI:** 10.3389/fpsyg.2022.1040208

**Published:** 2022-12-06

**Authors:** Zhiguang Ji, Liyan Wang, Ming Cai, Le Lu, Hongbiao Wang

**Affiliations:** ^1^Department of Physical Education, Shanghai University of Medicine and Health Sciences, Shanghai, China; ^2^College of Rehabilitation Sciences, Shanghai University of Medicine and Health Sciences, Shanghai, China

**Keywords:** developmental coordination disorder, cognitive function, web of science, CiteSpace, VOSviewer

## Abstract

**Objective:**

To identify the research hotspots on cognitive function in developmental coordination disorder (DCD) in recent years, predict the research frontier and development trend, and provide more perspectives for the study of the DCD population.

**Methods:**

Using CiteSpace and VOSviewer software to draw charts, 1,082 pieces of literature about DCD and cognitive function in the Web of Science core collection database from 2010 to 2022 were visually analyzed.

**Results and conclusion:**

Interest in the cognitive function of DCD has been on the rise in the past 10 years. Over 40 countries and regions, 117 institutions and 200 researchers have participated in the corresponding research, mainly in the United States, and their institutions have published more highly influential results. The hot keywords are DCD, children, attention, working memory, performance, and attention-deficit/hyperactivity disorder (ADHD), and the main research hot topics include functional performance, population, cognitive psychology. The research directions include “DCD,” “Asperger syndrome,” “memory,” “infant,” “clumsiness,” “neurodevelopmental disorder,” “occupational therapy,” “preschool children,” “motor competence,” “model,” and “online control.” Future research should focus on motor imagery and intrinsic models and use more neurophysiological techniques to reveal the cognitive characteristics of children with DCD and develop intervention programs.

## Introduction

Developmental coordination disorder (DCD) is a neurodevelopmental disorder that affects 5–6% of children, characterized by severe impairment of an individual’s ability to perform everyday motor tasks, including difficulties with self-care (such as shoe lacing), learning tasks (such as writing), and leisure activities (such as cycling; American Psychiatric Association, 2013). The diagnosis of DCD is based on four diagnostic criteria (American Psychiatric Association, 2013): (a) significantly lower than expected performance on motor coordination tasks compared to peers; (b) motor coordination impairments that can severely impact activities of daily living or academic performance; (c) the impairments begin early in development; and (d) the impairments cannot be attributed to an intellectual disability or neurological disorder (e.g., cerebral palsy). Although DCD is common, it is often underestimated by healthcare and education professionals (Wilson et al., 2013). Children with DCD show differences in motor skill learning and performance compared with typically developing children, which may affect activities of daily living and academic performance (Bernardi et al., 2018).

Existing studies have shown that children with DCD are more likely to have other additional neurodevelopmental disorders. Approximately half of children with DCD also have attention deficit/hyperactivity disorder (ADHD) symptoms (Kingdon et al., 2016) such as inattentive, impulsive behavior (Kenny et al., 2019) and executive dysfunction (Zelazo, 2020), and other neurodevelopmental disorders (Anderson and Levi, 2019). Executive functions have been the main cognitive function studied (Michel et al., 2018), although their structure in childhood is still currently debated (Laureys et al., 2022). Executive dysfunction is thought to be central to poor academic and occupational performance. DCD is also characterized by abnormalities in cognitive control (Hyde and Wilson, 2011), fine motor skills and balance (Zwicker et al., 2013). Poor motor performance in children with DCD could be due to their poor cognitive and psychomotor development (Bonney et al., 2017).

The underlying mechanisms of DCD remain poorly understood. Ecological approaches lay more emphasis on the dynamics of the interaction between individuals, tasks and the environment workplace (Wilson et al., 2017). The relationships between brain function, cognition, and behavior can be determined through systematic analytical studies of cognitive neuroscience and ecological approaches to assess motor behavior and motor skills in DCD. Cognitive neuroscience focuses on understanding the biological processes underlying cognition and action, and the causal relationships existing between brain function, cognition, and behavior. Motor control processes are also thought to provide insight into an individual’s ability to generate internal models of action that subserve efficient motor planning, control, and development. However, the combination of cognitive and motor disorders in children could have an impact on later development.

More robust evidence showed that DCD is not only a motor problem but that cognitive factors also contribute (Wilson et al., 2017). However, most studies focused on the behavior features of DCD and paid little attention to cognitive factors. It is necessary to summarize previous studies and update the current frontiers in this field. Thus, Citespace and VOSviewer were used to visualize the research trend and hotspots. This study will help us to better understand the current situation and frontiers. It will also provide new insights into the research trend and hotspots in this field, and future research advancement patterns in this area.

## Data and methods

### Data collection

The bibliometric analysis data were obtained from the Web of Science (WOS) Core Collection search indices, including science citation index expanded (SCI-E), social sciences citation index (SSCI), arts & humanities citation index (A&HCI), conference proceedings citation index-science (CPCI-S), conference proceedings citation index-social sciences and humanities (CPCI-SSH), emerging sources citations index (ESCI), current chemical reactions (CCR-EXPANDED), and index chemicus (IC). The search terms used the following search strategy: keywords = (developmental coordination disorder or poor motor coordination or low motor competence or poor motor skill) and (cognitive or executive function or motor control) and (Children). The retrieval time for published results was 2010–2022, and the retrieval date was August 21, 2022.

### Inclusion criteria

Inclusion criteria were: (1) peer-reviewed published original articles on cognitive function in children with developmental coordination disorder (DCD), including articles and reviews; (2) published in English; and (3) articles published from 2010 to 2022 on WOS.

### Exclusion criteria

Exclusion criteria were: (1) publication type was animal experiment; (2) articles not officially published; (3) conference abstracts and proceedings and corrigendum documents; (4) unrelated articles; and (5) non-article-type documents (e.g., book review, notification, editorial materials, meeting abstracts, proceedings papers, letters, news items, and corrections).

**Figure 1:**
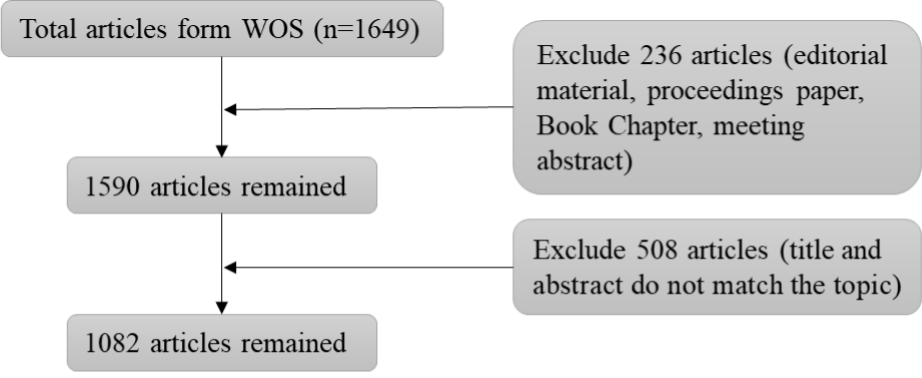
The steps of the inclusion process.

The search for publications was conducted from January 1, 2010, to August 21, 2022. A total of 1,885 records were initially retrieved; after the removal of articles that did not match the inclusion criterion, a total of 1,649 articles were finally determined as research objects. The flow chart of literature screening is shown in Figure 1.

### Analysis methods and tools

Clarity analysis, VOSviewer (1.6.16), and CiteSpace (5.7. R3) are used for bibliometric analysis to analyze the structure and measure clusters, links between clusters, and key or pivot points by building a visual index map. CiteSpace and VOSviewer are Java web-based data processing and visualization applications (Chen, 2004). The Web of Science database is the main source of input data for bibliometric analysis. We used the Microsoft Excel 2019 software to describe and predict the publication trend of articles.

VOSviewer is primarily used to generate collaborative network visualizations, average annual publication year maps, optimized network visualizations, and density visualizations (Van Eck and Waltman, 2010). In VOSviewer, collaborative partnerships are represented by connections between nodes, and the thickness of the connections indicates the closeness of the cooperation. The different colors represent different clusters, and the lines between the circles represent the partnerships between different points. In the average annual publication year map, different colors correspond to different years. In density visualization, the redder the color, the higher the density. CiteSpace is primarily used for counting centrality and generating collaboration network visualizations and citation burst year visualizations (Cui et al., 2018).

## Results

### Bibliometric analysis of publication outputs

**Figure 2:**
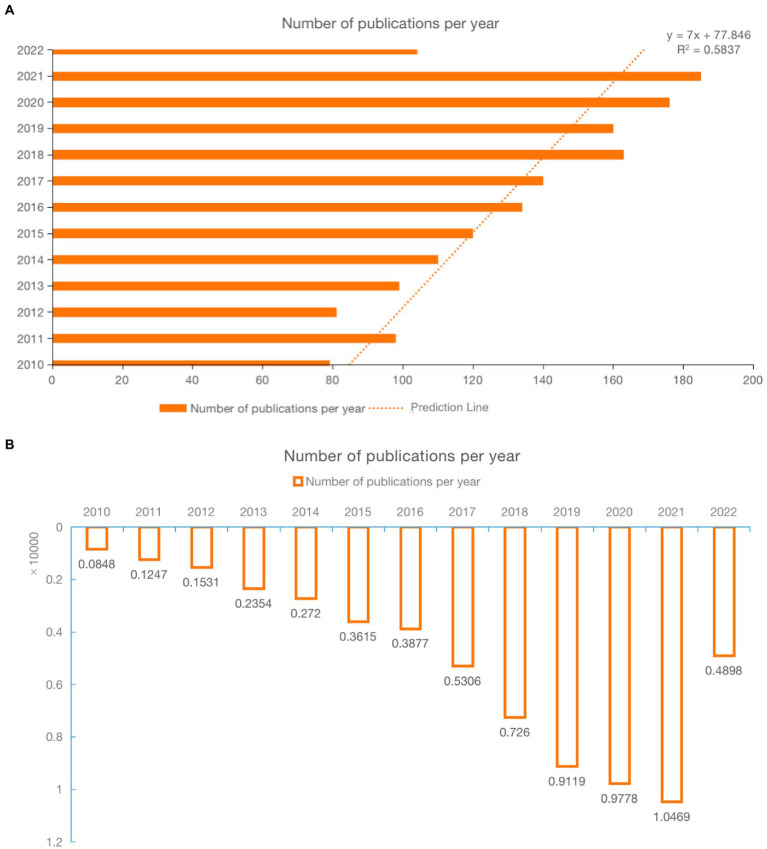
**(A)** The combination chart of the number of publications and Growth Trend Forecast Model. **(B)** Annual citations of publications.

This bibliometric analysis included 1,082 articles from 2010 to 2022. The annual publication and citation distributions of cognitive function in children with DCD are shown in Figure 2. In the last decade, the number of published papers showed a steady rise, increasing yearly from 2015. There has been a remarkable increase in the number of published papers in the last 5 years, indicating that cognitive function in DCD has received increasing attention. A growth trend model (R^2^ = 0.58) indicated that DCD and its mechanisms have received increasing attention. As shown in Figure 2A, the number of citations per year increased in the last decade and reached its peak in 2021.

### Bibliometric analysis of countries and regions

**Figure 3:**
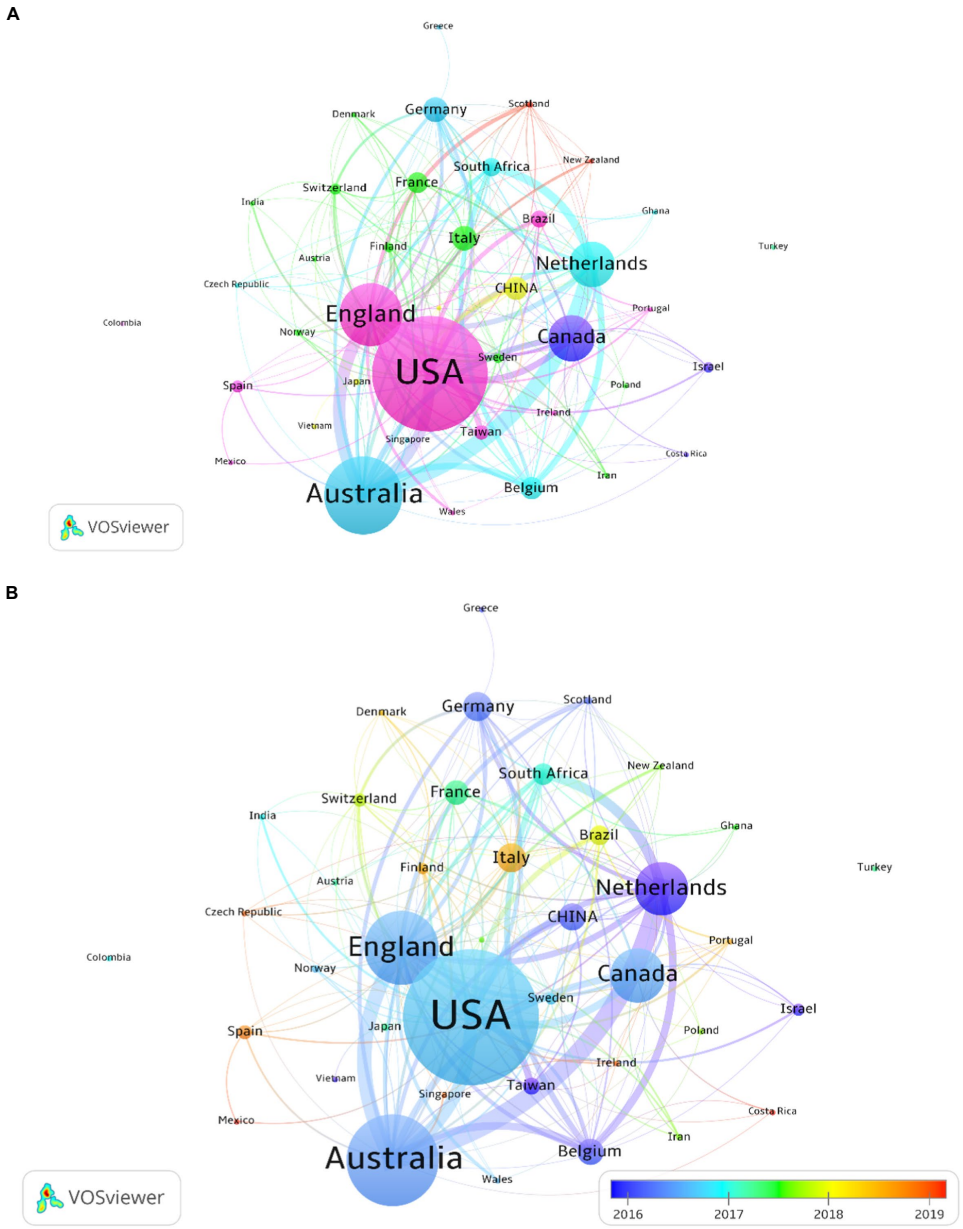
**(A)** Cooperation network visualization map of countries and regions based on VOSviewer; **(B)** Average annual publication year map of countries and regions based on VOSviewer.

Over 40 countries and regions published papers in the field of DCD and cognitive function in the WOS database. Table 1 lists the United States published the most articles (17.76%), followed by Australia (12.02%), England (9.69%), Canada (7.10%), Netherlands (6.97%), Germany (3.89%), Italy (3.83%), China (3.62%), Belgium (3.48%), and France (3.21%). Among them, the United States, England, Australia, Canada, and the Netherlands published the largest number of papers from 2010 to 2022, while papers from Italy, Belgium, France, South Africa, and Brazil were mainly published after 2017. The United States published 256 papers with 5,717 citations, with an average citation of 21.98 per year. A map of country and region cooperation through VOSviewer is shown in Figure 3. In this field, the United States obviously had close cooperation with many countries because of its huge amount of research, and Australia also showed extensive cooperation. In contrast, there was less cooperation between other countries. From these two maps, the United States was found to have the highest centrality and density, and the greatest influence.

**Table 1:** Countries and regions’ publication number, centrality, and citations.

Country	Count	Total citations	Average citations
All	1464.00	29675.00	17.25
USA	260.00	5717.00	21.99
Australia	176.00	4318.00	24.53
England	142.00	3100.00	21.83
Canada	104.00	2707.00	26.03
Netherlands	102.00	3272.00	32.08
Germany	57.00	1128.00	19.79
Italy	56.00	612.00	10.93
China	53.00	655.00	12.36
Belgium	51.00	1272.00	24.94
France	47.00	698.00	14.85

### Bibliometric analysis of institutions

**Figure 4:**
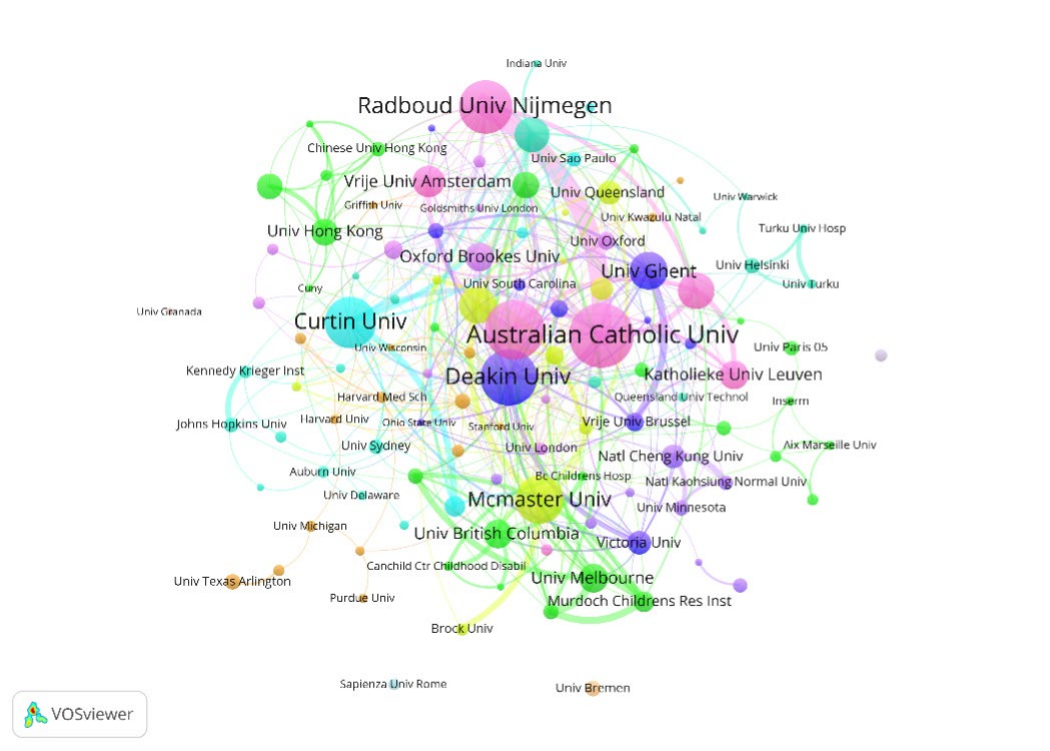
The cooperation network visualization map of institutions based on VOSviewer.

A total of 117 institutions published articles on DCD and cognitive function, and the most active institutions can be found through the visualization map (Figure 4). Table 2 lists the top 10 institutions with the highest output, with a total of 199 papers published, accounting for 37% of the total number of papers. Australia had three of the most productive institutions, the Netherlands and Canada both had two, and Belgium, South Africa, and England each had one (Table 2). It was found that most of the initial studies at institutions started before 2013, with only that of the University of Cape Town starting in 2015. Australian Catholic University was the most active institution in this field in terms of the number and density of publications. In addition, there was more cross-regional cooperation between Australian Catholic University, University of Groningen, and Deakin University in this field.

**Table 2:** Top ten institutions’ publication number, country, and first publication year.

Institution	Publications	Country	First publication year
Australian Catholic Univ	40	Australia	2012
Univ Groningen	37	Netherlands	2010
Deakin Univ	35	Australia	2013
Radboud Univ Nijmegen	33	Netherlands	2010
Curtin Univ	32	Australia	2011
Mcmaster Univ	30	Canada	2010
Univ Ghent	24	Belgium	2012
Univ Cape Town	23	South Africa	2015
Univ Toronto	23	Canada	2013
Univ Leeds	22	England	2010

Univ = university.

### Bibliometric analysis of active journals

**Figure 5:**
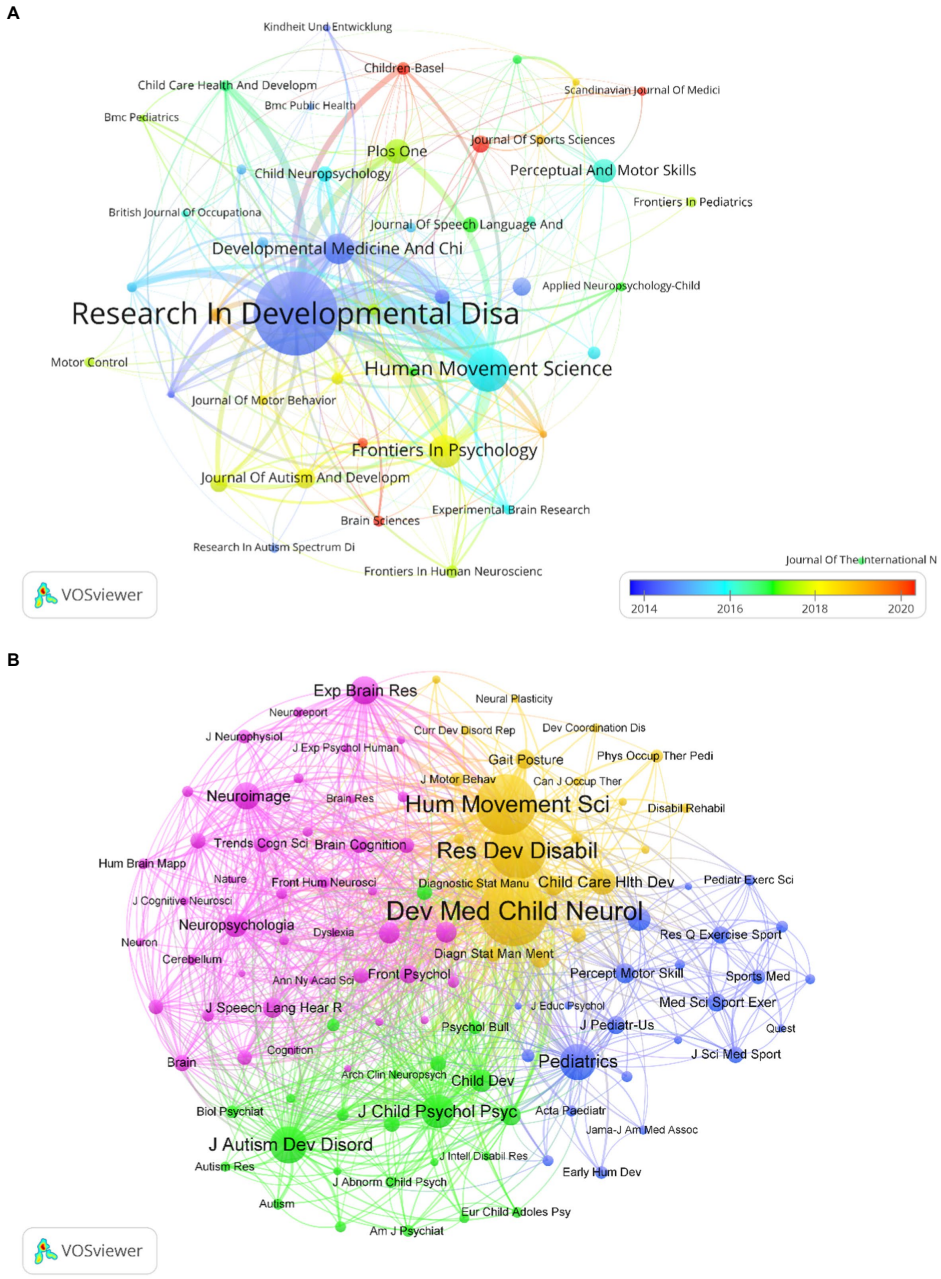
**(A)** Average annual publication year map of journals based on VOSviewer; **(B)** Co-citation network visualization map of co-cited journals based on VOSviewer.

A total of 110 journals participated in the publication of articles on cognitive function in DCD. Table 3 lists the top 5 journals and co-cited journals in the field. The top five journals in terms of the number of publications were *Research in Developmental Disabilities, Human Movement Science, Frontiers in Psychology, Developmental Medicine and Child Neurology, and PLOS One* (Table 3). The co-cited journals, which refers to the number of times a journal is co-cited in an article, represents the attention and contribution of the journal to the field. The top five co-cited journals were *Developmental Medicine & Child Neurology, Human Movement Science, Research in Developmental Disabilities, the Journal of Autism and Developmental Disorders, and Pediatrics* (Table 3). In terms of the number of publications, density, and citations, *Research in Developmental Disabilities* was the most important in this field (Figure 5). In terms of influence, co-cited journals and journals with a high impact factor (IF), such as *Pediatrics,* were also quite active in this field. Although these journals do not have many publications, they have a very high number of co-citations.

**Table 3:** Number of publications from the top five journals and co-cited journals.

Journal	Publications	Citations	IF (2022)	Journal	Co-citations	IF (2022)
Res Dev Disabil	112	2,770	3.000	Dev Med Child Neurol	2,284	4.864
Hum Movement Sci	47	893	2.397	Hum Movement Sci	1884	2.397
FRONT PSYCHOL	32	449	4.232	Res Dev Disabil	1718	3.000
Dev Med Child Neurol	30	1,063	4.864	J Autism Dev Disord	920	4.633
PLOS ONE	22	255	3.752	Pediatrics	911	9.703

### Bibliometric analysis of authors and authors’ co-citations

**Figure 6:**
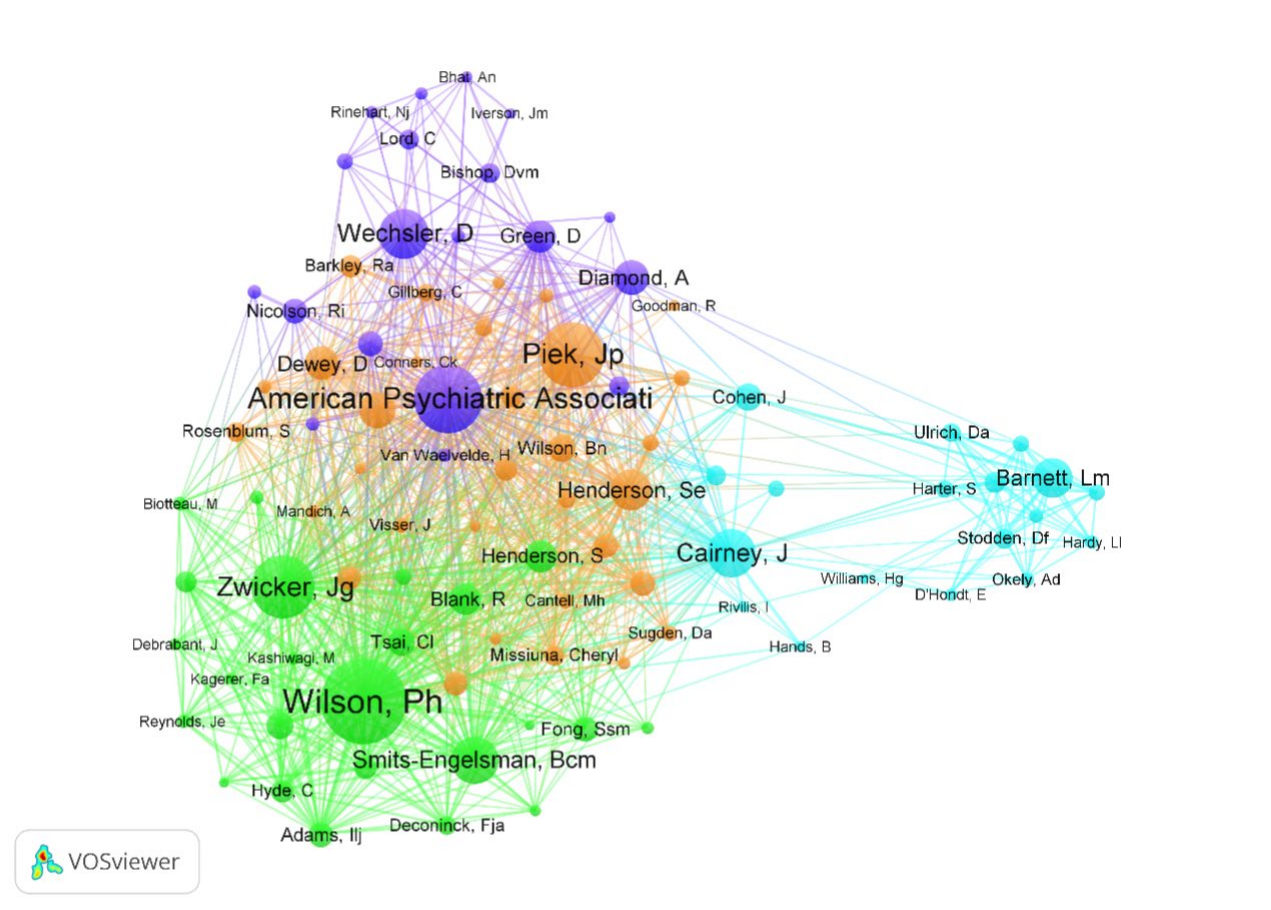
Co-citation network visualization map of authors based on VOSviewer.

A total of 200 researchers were found to have published research in this area Since 2010. The top five cited authors were Wilson, P. H., Barnett, L. M., Zwicker, J. G., Missiuna, C., and Steenbergen, B. The first five co-authors were Wilson, P. H., American Psychiatric Association, Piek, J. P., Zwicker, J. G., and Wechsler, D (Table 4). In terms of the number of papers and citations, Wilson, P.H. from Australian Catholic University was the most influential and contributing author in this field. He was found to have the most cited article in this field, focusing on the anticipatory control of movement in DCD, the basic processes of motor learning, and the wide-ranging deficits in cognitive control. Visualization maps can provide information about potential collaborators and can help researchers clarify collaboration relationships (Figure 6).

**Table 4:** Top five cited authors and top five co-cited authors.

Author	Publications	Citations	Co-author	Co-citations	Publications
Wilson, P. H.	19	854	Wilson, P. H.	435	200
Barnett, L. M.	17	651	American Psychiatric Association	345	263
Zwicker, J. G.	15	623	Piek, J. P.	320	174
Missiuna, C.	11	614	Zwicker, J. G.	313	143
Steenbergen, B.	23	547	Wechsler, D.	241	145

### Bibliometric analysis of references

**Figure 7:**
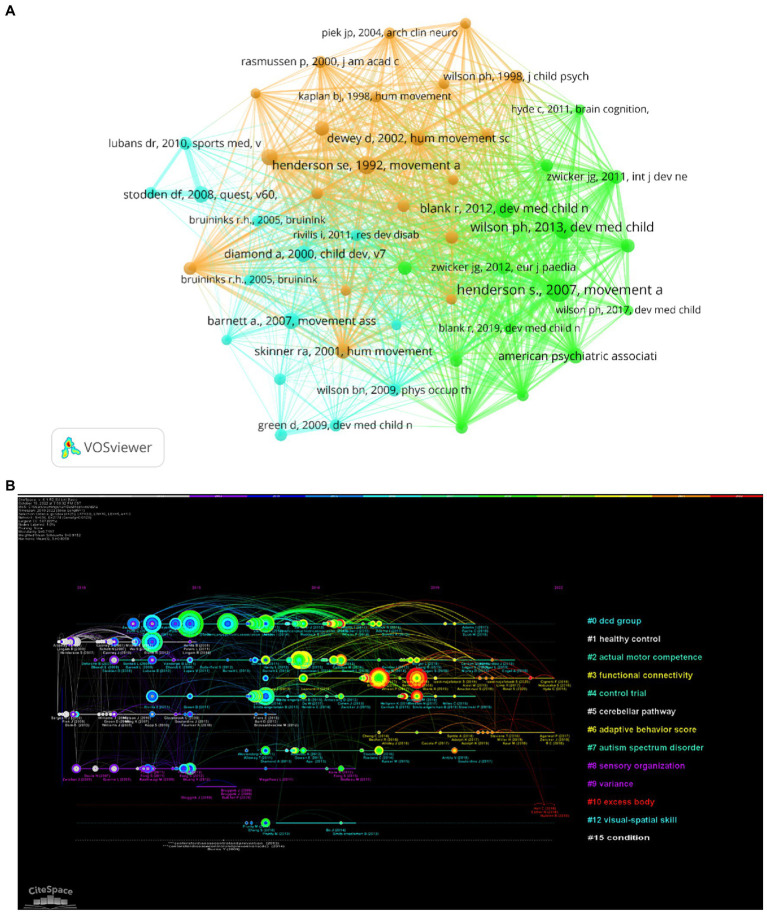
**(A)** Co-citation network visualization map of references based on VOSviewer; **(B)** Timeline view based on the reference co-citation analysis on CiteSpace.

Over the past 10 years, the top 5 co-cited references are shown in Table 5. Figure 7A shows the articles associated to research field between 2010 and 2022 were divided into 3 major research hotspots. Each cluster represented the citation index, achievement field and key literature series. The timeline view for the clusters, which narrated the time interval and the progress and evolution of the research, is shown in Figure 7B. Articles and their co-citation relevant data were searched to construct the major clusters. “DCD group” was the most significant cluster (#0) out of the 15 clusters, followed by cluster #1 (aerobic power) and cluster #2 (developmental coordination disorder).

**Table 5:** Top five co-cited references.

Rank	Co-cited (count)	Reference	First author (year)
1	146	Movement Assessment Battery for Children, second edition.	Henderson, S. 2007
2	126	Understanding performance deficits in developmental coordination disorder: a meta-analysis of recent research.	Wilson, P. H. 2013
3	98	European Academy for Childhood Disability (EACD): recommendations on the definition, diagnosis, and intervention of developmental coordination disorder (long version).	Blank, R. 2012
4	92	The Movement Assessment Battery for Children.	Henderson, S. 1992
5	80	Diagnostic and statistical manual of mental disorders: Text revision.	American Psychiatric Association 2000

### Bibliometric analysis of keywords

**Figure 8:**
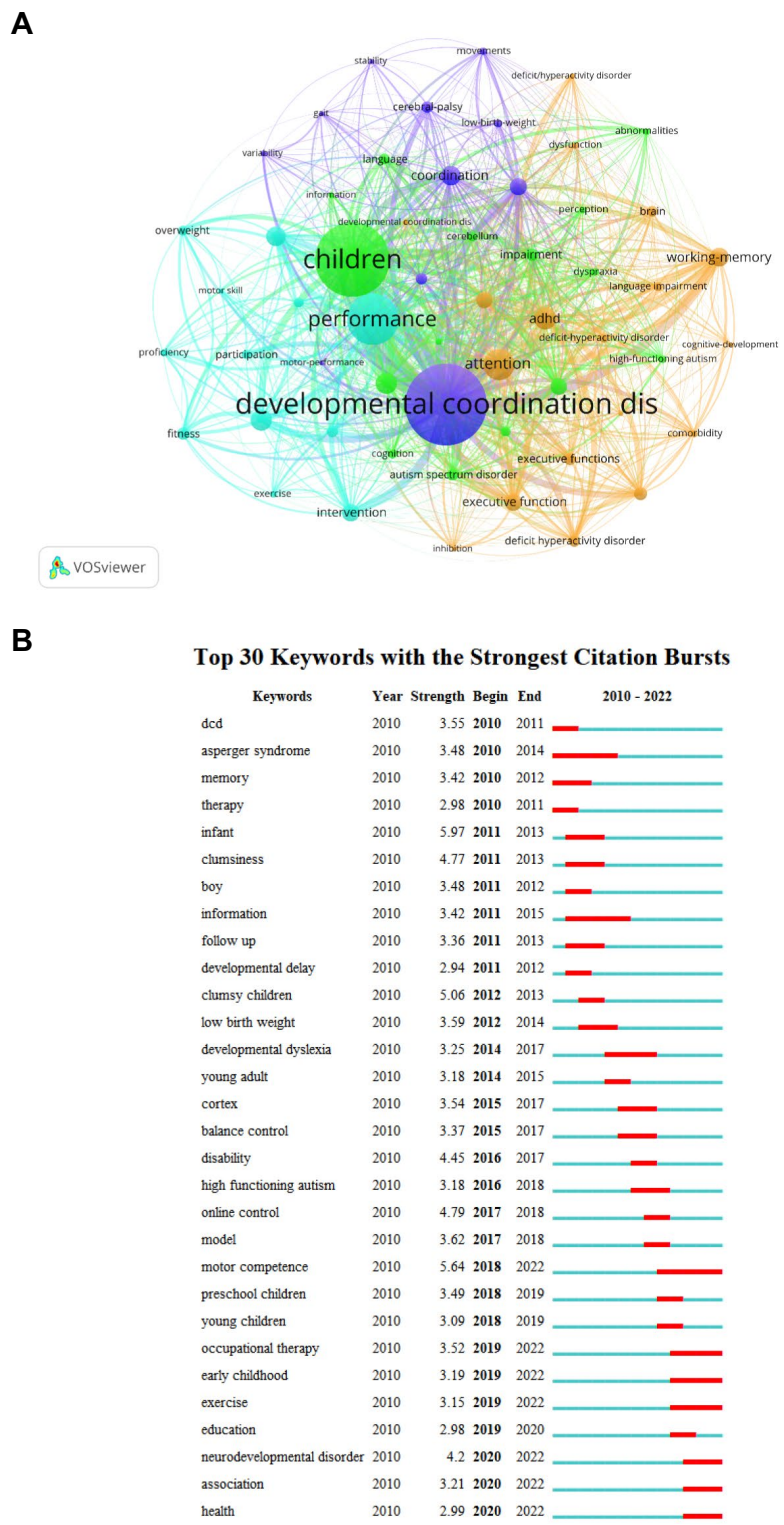
**(A)** Co-citation network visualization map of keywords based on VOSviewer; **(B)** Top 30 keywords with the strongest citation bursts based on CiteSpace. The red horizontal stripes represent the years with the most frequent keyword use. The green horizontal stripes represent the years with the most infrequent keyword use.

In this research field, keyword analysis can classify high-frequency keywords and determine the strong relationship between keywords by examining the frequency of the keywords in papers. It can identify the internal structure of an academic field and reveal the research frontiers of the discipline. Figure 8A shows the network and density of keywords, and four different keyword clusters were generated (Table 6). There was a greater correlation between the keywords in each cluster. The top 20 keywords with the strongest occurrence burst are shown in Figure 8B.

**Table 6:** Cluster analysis of keywords regarding cognitive function in DCD.

Cluster	Key words
1	ADHD, attention, brain, deficit hyperactivity, dysfunction, executive function, inhibition, language impairment, working-memory
2	Abnormalities, autism, behavior, cerebellum, information, perception, impairment
3	Exercise, fitness, intervention, motor skill, overweight, performance, physical activity
4	Gait, low birth weight, motor control, motor performance, movements, postural control, stability

The purple clusters comprise developmental coordination disorders, cerebral-palsy, stability, movements, and gait. The green clusters comprise children, high-functioning autism, abnormalities, deficit-hyperactivity disorder, cerebellum, and dyspraxia. The blue clusters comprise information on the intervention, such as motor skill, participation, proficiency, motor performance, overweight, exercise, and fitness. The relatively scattered connections in the orange clusters contain keywords such as executive functions, attention, deficit hyperactivity disorder, working-memory, brain, and ADHD. The top 50 burst references are shown in Figure 8B. We found that there were significant year-on-year bursts for keywords. Between 2010 and 2015, the keywords with the highest citations were “DCD,” “Asperger syndrome,” “memory,” “infant,” and “clumsiness.” “Neurodevelopmental disorder,” “occupational therapy,” “preschool children,” “motor competence,” “model,” and “online control” were the keywords with the high burst in the recent 5 years.

## Discussions

The bibliometric analysis results of DCD and cognitive function show that the number of articles published in recent years has gradually increased, indicating that an increasing number of researchers are paying attention to the field of DCD and cognitive function.

According to the analysis of countries and institutions, Australian, Dutch and Canadian institutions were found to have made significant contributions in this field. Links between countries and organizations remain weak, and strengthening international cooperation is a top priority in this area. Through the analysis of journals and authors, we found that “*research in developmental disabilities*” and “*human movement science*” were the most important journals in this field, but few high impact factor journals have made great contributions to the research. Scientists such as Wilson, P. H., Barnett, L. M., Zwicker, J. G., Missiuna, C., and Steenbergen, B were major players in this field. In the analysis by reference, “DCD group” was the most significant cluster. Children with DCD were considered as atypically motor developing children based on sensorimotor integration problems (Wilson et al., 2013). The articles published by Wilson, P. H. were the most influential in the field.

From the analysis of reference and keywords distribution presented in Figures 7, 8, the last decade of research could be summarized in three domains: (i) the poor cognitive performance; (ii) underlying cognitive mechanisms; and (iii) the intervention to improve performance. Analyses of these three domains follows:

### The poor cognitive-related performance of children with DCD

As other children with developmental disabilities, children with DCD may have cognitive impairments, such as inhibition, working memory and other cognitive functions (Subara-Zukic et al., 2022). DCD can occur alone or with other diseases and disorders. In particular, it can coexist with ADHD (Su et al., 2022). In addition, studies have confirmed that children with DCD exhibit sensorimotor disorders, balance and postural control disorders (Fong et al., 2013), motor planning deficits (Hyde and Wilson, 2013), and visuospatial deficits (Alloway, 2011), which are important factors in the occurrence of DCD. In recent years, research on DCD and cognitive function has mainly focused on the mirror neuron system (Barhoun et al., 2019; Reynolds et al., 2019), dyslexia, and optic–spatial dysfunction (Blank et al., 2019).

In addition to cognition, cognitive-related skills are also affected. Several studies have found that children with DCD have significant writing impairments, which can seriously affect their learning ability (Prunty et al., 2016). Nobusako and colleagues found that Children with DCD had more obvious visual deviations than children with typical development (TD), and the degree of visual deviations was significantly correlated with hand dexterity (Nobusako et al., 2021). Giofrè and colleagues found that children with DCD had difficulty copying new movements or responding to verbal cues (Giofre et al., 2014). Evidence confirms the relationship between DCD motor performance and executive functions (Rigoli et al., 2012). Michel et al.’s study found that children with DCD have poorer performance on inhibition and conversion compared with children without motor coordination disorders, making them prone to having lower preschool skills. Moreover, for those with lower preschool skills, education begins with a significant disadvantage (Michel et al., 2011). Tal-Saban and colleagues found that the nonacademic and academic functioning, including the use of executive strategies, goal setting, planning, and performance self-assessment strategies, of young adults with DCD in complex task performance was less than in controls (Saban et al., 2012). Rosenblum and colleagues found that adults with DCD have significant deficits in executive functions abilities (such as attention, planning, and organization), and that handwriting is a complex human activity that can serve as a sensitive indicator of executive functions, as reflected in the daily functioning of various activities (Rosenblum, 2013).

### Cognitive mechanisms underlying motor performance deficits in children with DCD

Although the underlying mechanisms of DCD is currently unclear, according to the atypical brain development hypothesis (Kaplan et al., 2006), motor dysfunction occurs due to changes in brain structure and function (Brown-Lum and Zwicker, 2015). Unlike earlier studies, which focused more on attention, executive functions, and working memory, recent studies have focused on the cortex, online control, and neurodevelopment disorders. Subara-Zukic and colleagues found that children with DCD have deficits in visual–motor mapping and cognitive–motor integration, manifested in the abnormal maturation of motor networks, and an area of practical compensation for motor control deficits (Subara-Zukic et al., 2022). So the relationship between cognitive functions and motor control has also been highlighted in DCD (Costini et al., 2018). Some studies have also suggested that abnormal brain motor imagery is an important factor leading to the impairment of motor activity in DCD (Barhoun et al., 2019). Children with DCD had less activation of the left brain, especially those with mirror neuron system and sensory integration functions. Therefore, the intervention should activate the left-brain visuospatial processing area (Irie et al., 2021).

Although the neural mechanisms underlying DCD are poorly understood, several research hotspots can be summarized. First, the ability to use cognitive resources during movement was limited, especially as children with DCD were slower in the dual task conditions (Schott et al., 2016). Based on Fawcett’s research on children with dyslexia explored the content of the cerebellar ensemble involved in the automation of motor skills in children with DCD and suggested the priority of postural control/walking, sensorimotor–cognitive interaction, various types of combined tasks, structural and functional changes in the brain, automation deficits, and other possible factors (Fawcett and Nicolson, 1992; Geuze et al., 2007; Schott et al., 2016). Children with DCD are thought to show activation of multiple diffuse but not specific brain regions (especially the cerebellum, prefrontal cortex, parietal cortex, and striatum), resulting in widespread motor and cognitive impairment. Second, regarding spatial cognition, children with DCD have deficits in closed-loop feedback processes or the ability to internalize visuospatial sensory information. For example, children with DCD perform worse in working memory of visuospatial material (Alloway and Kathryn, 2007). In addition, poor spatial ability may lead to other problems, such as illegible handwriting or poor drawing skills. Children with DCD also have impairments in spatiotemporal integration (Schott et al., 2016) and errors in judging speed (Purcell et al., 2011). Visuospatial attention is also different from the same age (Wilson et al., 2013), which will affect the daily life of children with DCD to varying degrees. Finally, numerous studies have confirmed the deficits in executive functions in children with DCD (Schott et al., 2016). Some studies have reported that the development of the prefrontal lobe in children with DCD may be one of the reasons for executive dysfunction (He et al., 2018), especially in the performance of inhibition and interference control (Livesey et al., 2006).

### Interventions to improve cognitive performance in children with DCD

Research on interventions for children with DCD has resulted in numerous interventions such as perceptual motor therapy (PMT), sensory integration therapy (SIT), group formats, individual coaching, motor imagery, weight-bearing exercises, writing exercises, kinesthetic training, specific skills training, and others (Miyahara et al., 2017). Current interventions can be broadly divided into two categories: process-oriented and task-oriented (Irie et al., 2021). These approaches are based on the fact that interventions reinforce underlying deficient processes and improve task performance by correcting impairments (Noordstar et al., 2017). Process-oriented approaches include sensory integration and sensory-motor interventions. The intervention hypothesis was that interventions improve physical functions such as perception, sensory integration, muscle strength, and visual–motor perception, which would lead to better skill performance (Blank et al., 2019). Task-oriented interventions are based on motor control and motor learning theories, including task-specific interventions, neuromotor task training, cognitive and daily performance training, and ecological interventions. Practical single-function early interventions and more extensive and comprehensive interventions are recommended in recent studies (Blank et al., 2019). Moreover, it seems better to use a combined task- and process-oriented form of intervention (Zwicker et al., 2012).

Similar to other studies in children (Pan et al., 2019), exercise was found to be an effective intervention in children with DCD (Pan et al., 2019). Yu found that, except for one study, 85% of the 59 studies that assessed exercise performance reported positive intervention effects, and 17 studies even showed significant sustained intervention effects. Most of the studies measuring cognitive function reported a significant and positive intervention effect after the intervention (Yu et al., 2018). In addition, an increasing number of studies are combining cognition and physical fitness in children with DCD (Keating et al., 2022), and exercise may be an appropriate choice. Exercise intervention can effectively improve the motor ability and the cognitive, emotional, and psychological performance of children with DCD in the short term, and the more comprehensive benefits are also an important reason for selection (Yu et al., 2018).

## Research limitations

The limitations that existed in the study should be taken into consideration in further research. First, the retrieve algorithm of WOS was not based on full text; therefore, a few relevant articles may have been missed. Second, we did not perform categorical searches for cognitive function, so relevant discussion is limited. Therefore, meta-analysis can be used in future research to provide more information.

## Conclusion

In summarizing the current research, it was found that there are various forms of cognitive deficits in children with DCD, and the mechanisms of damage and pathogenesis are still unclear. Compared with children with typical development, cognitive functions of children with DCD are frequently impaired, which may affect motor performance. Therefore, it is recommended that future research should deeply analyze the cognitive deficits of children with DCD and make advances in related research to explore the pathogenesis of DCD and find potential targets for DCD intervention. Future studies could use neurophysiological techniques to reveal more features and underlying mechanisms of DCD. In addition, effect of cognitive improvement on motor performance is yet to be established. In future research, cognitive function can be used as the output to analyze and improve the current definition, diagnosis, and intervention.

## Data availability statement

The original contributions presented in the study are included in the article/Supplementary material; further inquiries can be directed to the corresponding author.

## Ethical statement

The study was carried out ethically and approved by the Shanghai University of Medicine and Health Science.

## Author contributions

I have substantial contributions to the conception or design of the acquisition, analysis, or interpretation of data foe the work. I have drafted the work or revised it critically foe important intellectual content. I have approved the final version to be published. I agree to be accountable for all aspects of the work in ensuring that questions related to the accuracy or integrity of any part of the work are appropriately investigated and resolved. ZJ: conceptualization, data curation, investigation, software, and writing—original draft. LW, MC, HW, and LL: project administration, resources, supervision, visualization, and writing—review and editing. All authors contributed to the article and approved the submitted version.

## Funding

This work was supported by a grant from the funding of Education and Scientific Research Project of Shanghai (C2022009).

## Conflict of interest

The authors declare that the research was conducted in the absence of any commercial or financial relationships that could be construed as a potential conflict of interest.

## Publisher’s note

All claims expressed in this article are solely those of the authors and do not necessarily represent those of their affiliated organizations, or those of the publisher, the editors and the reviewers. Any product that may be evaluated in this article, or claim that may be made by its manufacturer, is not guaranteed or endorsed by the publisher.
