# Supplementary figures and images for: Bibliometric analysis study on cognitive function in developmental coordination disorder from 2010 to 2022
Source: Front Psychol. 2022 Dec 6;13:1040208. doi: 10.3389/fpsyg.2022.1040208 (PMC9764009; doi:10.3389/fpsyg.2022.1040208)

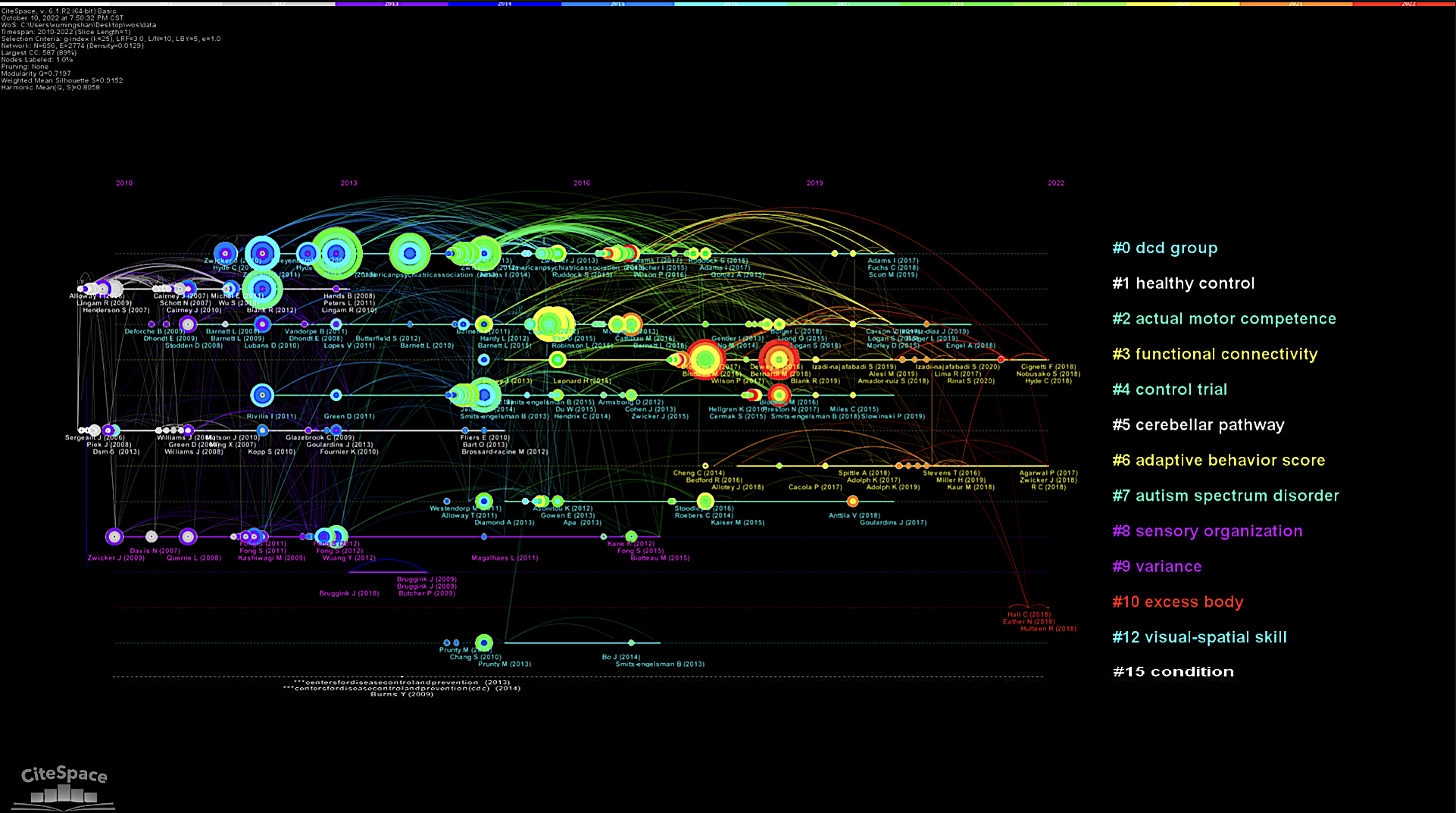

Supplement: Supplementary file 1 [file Image_1.PNG]
