# Supplementary material for: Bibliometric analysis study on cognitive function in developmental coordination disorder from 2010 to 2022
Source: Front Psychol. 2022 Dec 6;13:1040208. doi: 10.3389/fpsyg.2022.1040208 (PMC9764009; doi:10.3389/fpsyg.2022.1040208)
Supplement: Supplementary file 2 [file Table_1.DOCX]

FN Clarivate Analytics Web of Science

VR 1.0

PT J

AU Rafique, SA

Northway, N

AF Rafique, Sara A.

Northway, Nadia

TI Relationship of ocular accommodation and motor skills performance in

developmental coordination disorder

SO HUMAN MOVEMENT SCIENCE

LA English

DT Article

DE Developmental coordination disorder; Motor skills; Ocular accommodation;

Ocular motor

ID VISUAL PERCEPTUAL DEFICITS; FASTIGIAL NUCLEUS; YOUNG-ADULTS; CHILDREN;

CEREBELLUM; ATTENTION; INFORMATION; VERGENCE; SACCADES; LANGUAGE

AB Ocular accommodation provides a well-focussed image, feedback for accurate eye movement control, and cues for depth perception. To accurately perform visually guided motor tasks, integration of ocular motor systems is essential. Children with motor coordination impairment are established to be at higher risk of accommodation anomalies. The aim of the present study was to examine the relationship between ocular accommodation and motor tasks, which are often overlooked, in order to better understand the problems experienced by children with motor coordination impairment. Visual function, gross and fine motor skills were assessed in children with developmental coordination disorder (DCD) and typically developing control children. Children with DCD had significantly poorer accommodation facility and amplitude dynamics compared to controls. Results indicate a relationship between impaired accommodation and motor skills. Specifically, accommodation anomalies correlated with visual motor, upper limb and fine dexterity task performance. Consequently, we argue accommodation anomalies influence the ineffective coordination of action and perception in DCD. Furthermore, reading disabilities were related to poorer motor performance. We postulate the role of the fastigial nucleus as a common pathway for accommodation and motor deficits. Implications of the findings and recommended visual screening protocols are discussed. (C) 2015 Elsevier B.V. All rights reserved.

C1 [Rafique, Sara A.; Northway, Nadia] Glasgow Caledonian Univ, Sch Hlth & Life Sci, Glasgow G4 0BA, Lanark, Scotland.

RP Northway, N (通讯作者)，Glasgow Caledonian Univ, Cowcaddens Rd, Glasgow G4 0BA, Lanark, Scotland.

EM n.northway@gcu.ac.uk

OI Rafique, Sara/0000-0003-3636-4001

CR Abdi S, 2005, DOC OPHTHALMOL, V111, P65, DOI 10.1007/s10633-005-4722-4

Alloway TP, 2007, J EXP CHILD PSYCHOL, V96, P20, DOI 10.1016/j.jecp.2006.07.002

American Psychiatric Association, 2000, CAT 315 340 DC DIAGN, P53

Anderson HA, 2008, INVEST OPHTH VIS SCI, V49, P2919, DOI 10.1167/iovs.07-1492

Aziz S, 2006, STRABISMUS, V14, P183, DOI 10.1080/09273970601026185

Bauman ML, 1997, INT REV NEUROBIOL, V41, P367, DOI 10.1016/S0074-7742(08)60360-8

Benjamin W. J., 2006, BORISHS CLIN REFRACT, V2

Bingham GP, 2001, J EXP PSYCHOL HUMAN, V27, P1314, DOI 10.1037//0096-1523.27.6.1314

Brambring M., 2001, VISUAL IMPAIRMENT RE, V3, P41, DOI DOI 10.1076/VIMR.3.1.41.4415

Brenner E, 1998, VISION RES, V38, P493, DOI 10.1016/S0042-6989(97)00236-8

Bruininks R.H., 2005, BRUININKS OSERETSKY

CANTELL MH, 1994, ADAPT PHYS ACT Q, V11, P115, DOI 10.1123/apaq.11.2.115

Cantin N, 2007, HUM MOVEMENT SCI, V26, P491, DOI 10.1016/j.humov.2007.03.004

Castellanos FX, 2001, ARCH GEN PSYCHIAT, V58, P289, DOI 10.1001/archpsyc.58.3.289

Cermak S. A., 2002, DEV COORDINATION DIS

Ciuffreda KJ, 2006, BORISHS CLIN REFRACT, P93

CIUFFREDA KJ, 1995, EYE MOVEMENT BASICS

de Oliveira RF, 2010, EXP BRAIN RES, V205, P387, DOI 10.1007/s00221-010-2373-5

Dewey D, 2002, HUM MOVEMENT SCI, V21, P905, DOI 10.1016/S0167-9457(02)00163-X

Duckman R H, 1984, J Am Optom Assoc, V55, P281

Dudley L. M., 2010, J BEHAV OPTOMETRY, V21, P39

DUHAMEL JR, 1992, SCIENCE, V255, P90

ENRIGHT JT, 1986, J PHYSIOL-LONDON, V371, P69, DOI 10.1113/jphysiol.1986.sp015962

Evans B. J. W., 2002, PICKWELLS BINOCULAR

Evans BJW, 1996, OPHTHAL PHYSL OPT, V16, P3

Fabbro F, 2000, J NEUROLINGUIST, V13, P83, DOI 10.1016/S0911-6044(00)00005-1

Fawcett AJ, 1999, J MOTOR BEHAV, V31, P68, DOI 10.1080/00222899909601892

FAWCETT AJ, 1995, J MOTOR BEHAV, V27, P235, DOI 10.1080/00222895.1995.9941713

Field DT, 2007, J NEUROSCI, V27, P8002, DOI 10.1523/JNEUROSCI.2130-07.2007

Fogassi L, 2005, CURR OPIN NEUROBIOL, V15, P626, DOI 10.1016/j.conb.2005.10.015

Gamlin PDR, 1999, OPHTHAL PHYSL OPT, V19, P81, DOI 10.1046/j.1475-1313.1999.00434.x

Garcia A, 2002, OPTOMETRY VISION SCI, V79, P779, DOI 10.1097/00006324-200212000-00010

Geuze Reint H., 2005, Neural Plasticity, V12, P183, DOI 10.1155/NP.2005.183

Goss DA, 2001, OPHTHAL PHYSL OPT, V21, P484, DOI 10.1046/j.1475-1313.2001.00610.x

Hayes GJ, 1998, OPTOMETRY VISION SCI, V75, P506, DOI 10.1097/00006324-199807000-00019

Hill EL, 2001, INT J LANG COMM DIS, V36, P149, DOI 10.1080/13682820010019874

Howard I.P., 1995, BINOCULAR VISION STE

HULME C, 1984, CHILD CARE HLTH DEV, V10, P117, DOI 10.1111/j.1365-2214.1984.tb00171.x

HULME C, 1982, NEUROPSYCHOLOGIA, V20, P475, DOI 10.1016/0028-3932(82)90046-X

Hung GK, 1996, OPHTHAL PHYSL OPT, V16, P31, DOI 10.1016/0275-5408(95)00110-7

HUNG GK, 1980, IEEE T BIO-MED ENG, V27, P439, DOI 10.1109/TBME.1980.326752

Hurst CMF, 2006, OPHTHAL PHYSL OPT, V26, P199, DOI 10.1111/j.1475-1313.2006.00366.x

Ito M, 2000, BRAIN RES, V886, P237, DOI 10.1016/S0006-8993(00)03142-5

Ivry Richard B., 2003, Neural Plasticity, V10, P141, DOI 10.1155/NP.2003.141

JEANNEROD M, 1995, TRENDS NEUROSCI, V18, P314, DOI 10.1016/0166-2236(95)93921-J

Kagerer FA, 2004, MOTOR CONTROL, V8, P450, DOI 10.1123/mcj.8.4.450

Kaufman P. L., 2003, ADLERS PHYSL EYE CLI

Kirby A, 2007, J ROY SOC MED, V100, P182, DOI 10.1258/jrsm.100.4.182

Kirby A, 2008, J RES SPEC EDUC NEED, V8, P120, DOI 10.1111/j.1471-3802.2008.00111.x

Knowlton M., 1995, RE VIEW, V27, P133

Konczak J, 2005, BRAIN, V128, P1428, DOI 10.1093/brain/awh385

KRISHNAN VV, 1973, VISION RES, V13, P1545, DOI 10.1016/0042-6989(73)90013-8

Kulp MT, 1996, OPTOMETRY VISION SCI, V73, P255, DOI 10.1097/00006324-199604000-00007

Langaas T, 1998, VISION RES, V38, P1817, DOI 10.1016/S0042-6989(97)00399-4

Latash ML, 2006, MOTOR CONTROL AND LEARNING, P141, DOI 10.1007/0-387-28287-4_13

Leat S. J., 1996, OPHTHAL PHYSL OPT, V16, P375

Leat SJ, 1996, OPHTHAL PHYSL OPT, V16, P375

Lee DN., 1985, VISUAL TIMING INTERC, P1, DOI 10.1007/978-94-009-5071-9_1

LORD R, 1987, DEV MED CHILD NEUROL, V29, P250

Maxwell J, 2012, VISION RES, V62, P93, DOI 10.1016/j.visres.2012.03.013

Mazow M L, 1989, Trans Am Ophthalmol Soc, V87, P158

McClelland JF, 2003, OPHTHAL PHYSL OPT, V23, P243, DOI 10.1046/j.1475-1313.2003.00113.x

McClelland JF, 2006, INVEST OPHTH VIS SCI, V47, P1824, DOI 10.1167/iovs.05-0825

MILLODOT M, 1989, OPHTHAL PHYSL OPT, V9, P126, DOI 10.1111/j.1475-1313.1989.tb00831.x

MISSIUNA C, 2006, J PAEDIATR CHILD H, V11, P507

Molinari M, 1997, BRAIN, V120, P1753, DOI 10.1093/brain/120.10.1753

Mon-Williams M, 1999, PERCEPTION, V28, P167, DOI 10.1068/p2737

Mon-Williams M, 2000, ERGONOMICS, V43, P391, DOI 10.1080/001401300184486

MONWILLIAMS MA, 1994, ADAPT PHYS ACT Q, V11, P170

MYERS GA, 1990, OPHTHAL PHYSL OPT, V10, P175, DOI 10.1111/j.1475-1313.1990.tb00972.x

Nicolson RI, 1999, LANCET, V353, P1662, DOI 10.1016/S0140-6736(98)09165-X

Nixon PD, 2003, CEREBELLUM, V2, P114, DOI 10.1080/14734220309410

O'Hare Anne, 2002, Dyslexia, V8, P234, DOI 10.1002/dys.230

Parush S, 1998, PERCEPT MOTOR SKILL, V86, P291, DOI 10.2466/pms.1998.86.1.291

Pauc R., 2008, CLIN CHIROPRACTIC, V11, P130

Piek JP, 1999, J INT NEUROPSYCH SOC, V5, P320, DOI 10.1017/S1355617799544032

ROBINSON FR, 1993, J NEUROPHYSIOL, V70, P1741, DOI 10.1152/jn.1993.70.5.1741

Rosenblum S, 2008, HUM MOVEMENT SCI, V27, P200, DOI 10.1016/j.humov.2008.02.011

ROSENFIELD M, 1995, OPHTHAL PHYSL OPT, V15, P231, DOI 10.1016/0275-5408(95)90576-N

Ruz M, 2005, NEUROIMAGE, V27, P852, DOI 10.1016/j.neuroimage.2005.05.031

Scheiman MM., 2006, OPTOMETRIC MANAGEMEN, V2nd ed

Schmahmann JD, 2007, CEREBELLUM, V6, P254, DOI 10.1080/14734220701490995

Schoemaker MM, 2005, DEV MED CHILD NEUROL, V47, P390, DOI 10.1017/S0012162205000769

Schor CM, 1999, VISION RES, V39, P3769, DOI 10.1016/S0042-6989(99)00094-2

Scudder CA, 2002, ANN NY ACAD SCI, V978, P63, DOI 10.1111/j.1749-6632.2002.tb07556.x

Stack D. M., 1989, EARLY CHILD DEV CARE, V43, P1

STEIN JF, 1992, PHYSIOL REV, V72, P967, DOI 10.1152/physrev.1992.72.4.967

Sterner B, 1999, DOC OPHTHALMOL, V99, P93, DOI 10.1023/A:1002623107251

Sterner B, 2001, ACCOMMODATIVE FACILI

Stowe LA, 2004, BRAIN LANG, V89, P290, DOI 10.1016/S0093-934X(03)00359-6

Sun L. W., 2013, OPEN J NEUROSCIENCE, V3, P1

Swinny J. D., 2005, Neural Plasticity, V12, P153, DOI 10.1155/NP.2005.153

Takeda T, 1999, VISION RES, V39, P2087, DOI 10.1016/S0042-6989(98)00258-2

Thach WT, 1998, NEUROBIOL LEARN MEM, V70, P177, DOI 10.1006/nlme.1998.3846

TIFFIN J, 1968, PURDUE PEGBOARD EXAM

Torgesen J.K., 1999, TEST WORD READING EF

Van Waelvelde H, 2004, DEV MED CHILD NEUROL, V46, P661, DOI 10.1017/s0012162204001112

Visser J, 2003, HUM MOVEMENT SCI, V22, P479, DOI 10.1016/j.humov.2003.09.005

Wilkins AJ, 1996, OPHTHAL PHYSL OPT, V16, P491, DOI 10.1046/j.1475-1313.1996.96000282.x

Wilmut K, 2007, DISABIL REHABIL, V29, P47, DOI 10.1080/09638280600947765

WILSON BN, 1995, AM J OCCUP THER, V49, P8, DOI 10.5014/ajot.49.1.8

Wilson PH, 1998, J CHILD PSYCHOL PSYC, V39, P829, DOI 10.1111/1469-7610.00384

WINN B, 1991, OPHTHAL PHYSL OPT, V11, P335, DOI 10.1016/0275-5408(91)90050-S

Wold JE, 2003, J CATARACT REFR SURG, V29, P1878, DOI 10.1016/S0886-3350(03)00667-9

WOODHOUSE JM, 1993, INVEST OPHTH VIS SCI, V34, P2382

Zwicker JG, 2011, INT J DEV NEUROSCI, V29, P145, DOI 10.1016/j.ijdevneu.2010.12.002

NR 106

TC 7

Z9 7

U1 1

U2 32

PU ELSEVIER SCIENCE BV

PI AMSTERDAM

PA PO BOX 211, 1000 AE AMSTERDAM, NETHERLANDS

SN 0167-9457

EI 1872-7646

J9 HUM MOVEMENT SCI

JI Hum. Mov. Sci.

PD AUG

PY 2015

VL 42

BP 1

EP 14

DI 10.1016/j.humov.2015.04.006

PG 14

WC Neurosciences; Psychology; Psychology, Experimental; Sport Sciences

WE Science Citation Index Expanded (SCI-EXPANDED); Social Science Citation Index (SSCI)

SC Neurosciences & Neurology; Psychology; Sport Sciences

GA CN5LJ

UT WOS:000358470500001

PM 25912514

DA 2022-08-17

ER

PT J

AU Zwicker, JG

Missiuna, C

Harris, SR

Boyd, LA

AF Zwicker, Jill G.

Missiuna, Cheryl

Harris, Susan R.

Boyd, Lara A.

TI Brain activation associated with motor skill practice in children with

developmental coordination disorder: an fMRI study

SO INTERNATIONAL JOURNAL OF DEVELOPMENTAL NEUROSCIENCE

LA English

DT Article

DE Developmental coordination disorder, DCD; fMRI; Motor skill acquisition

ID SPATIAL WORKING-MEMORY; DEFICIT HYPERACTIVITY DISORDER;

POSITRON-EMISSION-TOMOGRAPHY; HAND TRACKING MOVEMENTS; CEREBELLAR

CONTRIBUTIONS; FRONTAL-CORTEX; FORCE CONTROL; ATTENTION; TASK;

PERFORMANCE

AB Children with developmental coordination disorder (DCD) have difficulty in learning new motor skills. At present, it is not known whether these children employ a different set of brain regions than typically developing (TD) children during skilled motor practice. Using functional magnetic resonance imaging, we mapped brain activity associated with skilled motor practice of a trail-tracing task in 7 children with DCD and 7 age-matched controls (aged 8-12 years). We indexed change in motor performance as a reduction in tracing error from early practice to retention. Children with DCD showed less blood-oxygen-level-dependent signal as compared to TD children in a network of brain regions associated with skilled motor practice: bilateral inferior parietal lobules (Brodmann Area (BA) 40), right lingual gyrus (BA 18), right middle frontal gyrus (BA 9), left fusiform gyrus (BA 37), right cerebellar crus I, left cerebellar lobule VI, and left cerebellar lobule IX. While no statistically significant differences were detected, effect size testing revealed that children with DCD demonstrated poorer tracing accuracy than TD children at retention (d = 0.48). Our results suggest that, compared to TD peers, children with DCD demonstrate under-activation in cerebellar-parietal and cerebellar-prefrontal networks and in brain regions associated with visual-spatial learning. These data suggest a neurobiological correlation with impaired learning of motor skills in children with DCD, which will need to be confirmed with a larger sample. (C) 2010 ISDN. Published by Elsevier Ltd. All rights reserved.

C1 [Zwicker, Jill G.] Univ British Columbia, Fac Med, Vancouver, BC V6T 2B5, Canada.

[Missiuna, Cheryl] McMaster Univ, Sch Rehabil Sci & CanChild, Fac Hlth Sci, Hamilton, ON L8S 1C7, Canada.

[Harris, Susan R.; Boyd, Lara A.] Univ British Columbia, Fac Med, Dept Phys Therapy, Vancouver, BC V6T 1Z3, Canada.

RP Zwicker, JG (通讯作者)，Child & Family Res Inst, L408-4480 Oak St, Vancouver, BC V6H 3V4, Canada.

EM jzwicker@cw.bc.ca; missiuna@mcmaster.ca; shar@interchange.ubc.ca;

lara.boyd@ubc.ca

RI Boyd, Lara/CAF-0372-2022; Hassan, Randa/AAW-3624-2021

OI Harris, Susan R/0000-0003-2679-6548; Zwicker, Jill/0000-0002-2986-6938

FU Vancouver Coastal Health Research Institute and Foundation; Canadian

Institutes of Health Research Musculoskeletal and Arthritis Institute;

Michael Smith Foundation for Health Research (MSFHR); Ontario Ministry

of Health and Long Term Care; Ontario Neurotrauma Foundation

FX This study was funded by a grant from the Vancouver Coastal Health

Research Institute and Foundation awarded to Dr. Boyd. Dr. Zwicker was

supported by a Quality of Life Strategic Training Fellowship in

Rehabilitation Research from the Canadian Institutes of Health Research

Musculoskeletal and Arthritis Institute and a Senior Graduate Training

Scholarship from the Michael Smith Foundation for Health Research

(MSFHR). Dr. Missiuna is supported by a Rehabilitation Scientist award

from the Ontario Ministry of Health and Long Term Care and the Ontario

Neurotrauma Foundation. Dr. Boyd is Canada Research Chair in

Neurobiology of Motor Learning and a MSFHR Career Investigator.

CR Allen G, 2005, NEUROIMAGE, V28, P39, DOI 10.1016/j.neuroimage.2005.06.013

American Psychological Association, 2000, DIAGN STAT MAN MENT, DOI DOI 10.1176/APPI.BOOKS.9780890423349

Anguera JA, 2010, J COGNITIVE NEUROSCI, V22, P1917, DOI 10.1162/jocn.2009.21351

Boyd LA, 2004, NEUROREHAB NEURAL RE, V18, P134, DOI 10.1177/0888439004269072

Boyd LA, 2009, EXP BRAIN RES, V194, P527, DOI 10.1007/s00221-009-1726-4

Cantin N, 2007, HUM MOVEMENT SCI, V26, P491, DOI 10.1016/j.humov.2007.03.004

CLEMENTSSTEPHEN.AM, 2009, NEUROSCI LETT, V16, P155

Clower DM, 2001, J NEUROSCI, V21, P6283, DOI 10.1523/JNEUROSCI.21-16-06283.2001

COHEN J, 1988, STAT POWER ANAL BEHA, pR21

CONNERS CK, 2001, CONNERS RATING SCALE

Courtney SM, 1998, SCIENCE, V279, P1347, DOI 10.1126/science.279.5355.1347

Cox RW, 1996, COMPUT BIOMED RES, V29, P162, DOI 10.1006/cbmr.1996.0014

Dewey D, 2002, HUM MOVEMENT SCI, V21, P905, DOI 10.1016/S0167-9457(02)00163-X

Dickstein SG, 2006, J CHILD PSYCHOL PSYC, V47, P1051, DOI 10.1111/j.1469-7610.2006.01671.x

Diedrichsen J, 2005, J NEUROSCI, V25, P9919, DOI 10.1523/JNEUROSCI.1874-05.2005

Doyon J, 1996, EUR J NEUROSCI, V8, P637, DOI 10.1111/j.1460-9568.1996.tb01249.x

Doyon J, 2002, P NATL ACAD SCI USA, V99, P1017, DOI 10.1073/pnas.022615199

Doyon J, 2003, NEUROPSYCHOLOGIA, V41, P252, DOI 10.1016/S0028-3932(02)00158-6

Estil LB, 2002, EXP BRAIN RES, V147, P153, DOI 10.1007/s00221-002-1193-7

Fassbender C, 2004, COGNITIVE BRAIN RES, V20, P132, DOI 10.1016/j.cogbrainres.2004.02.007

FITTS PM, 1954, J EXP PSYCHOL, V47, P381, DOI 10.1037/h0055392

Geuze Reint H., 2005, Neural Plasticity, V12, P183, DOI 10.1155/NP.2005.183

GHEZ C, 2000, PRINCIPLES NEURAL SC, P832

Grafton ST, 2008, NEUROIMAGE, V39, P1383, DOI 10.1016/j.neuroimage.2007.09.062

Gramsbergen Albert, 2003, Neural Plasticity, V10, P129, DOI 10.1155/NP.2003.129

Graydon FX, 2005, COGNITIVE BRAIN RES, V22, P373, DOI 10.1016/j.cogbrainres.2004.09.007

Habas C, 2009, J NEUROSCI, V29, P8586, DOI 10.1523/JNEUROSCI.1868-09.2009

Halsband U, 2006, J PHYSIOL-PARIS, V99, P414, DOI 10.1016/j.jphysparis.2006.03.007

Henderson S., 2007, MOVEMENT ASSESSMENT

HENDERSON SE, 1992, MOVEMENT ASSESSMENT

Hwang EJ, 2005, J NEURAL ENG, V2, DOI 10.1088/1741-2560/2/3/S09

Ivry Richard B., 2003, Neural Plasticity, V10, P141, DOI 10.1155/NP.2003.141

JENKINS IH, 1994, J NEUROSCI, V14, P3775

Johnston LM, 2002, HUM MOVEMENT SCI, V21, P583, DOI 10.1016/S0167-9457(02)00153-7

Jueptner M, 1997, J NEUROPHYSIOL, V77, P1325, DOI 10.1152/jn.1997.77.3.1325

Jueptner M, 1997, J NEUROPHYSIOL, V77, P1313, DOI 10.1152/jn.1997.77.3.1313

Kagerer FA, 2006, HUM MOVEMENT SCI, V25, P622, DOI 10.1016/j.humov.2006.06.003

Kagerer FA, 2004, MOTOR CONTROL, V8, P450, DOI 10.1123/mcj.8.4.450

Kashiwagi M, 2009, NEUROREPORT, V20, P1319, DOI 10.1097/WNR.0b013e32832f4d87

Kaufman A. S., 2004, K BIT 2 KAUFMAN BRIE

Lang CE, 1999, J NEUROPHYSIOL, V82, P2108, DOI 10.1152/jn.1999.82.5.2108

LUNDYEKMAN L, 1991, J COGNITIVE NEUROSCI, V3, P367, DOI 10.1162/jocn.1991.3.4.367

MARCHIORI GE, 1987, ADAPTED PHYSICAL ACT, V4, P305

Miall RC, 2000, EXP BRAIN RES, V135, P22, DOI 10.1007/s002210000491

Miall RC, 2005, EXP BRAIN RES, V166, P170, DOI 10.1007/s00221-005-2351-5

Miall RC, 2001, NAT NEUROSCI, V4, P638, DOI 10.1038/88465

Milham MP, 2003, NEUROIMAGE, V18, P483, DOI 10.1016/S1053-8119(02)00050-2

Missiuna Cheryl, 2006, Can J Occup Ther, V73, P7

Orban P, 2010, NEUROIMAGE, V49, P694, DOI 10.1016/j.neuroimage.2009.08.055

PASCUALLEONE A, 1993, ANN NEUROL, V34, P594, DOI 10.1002/ana.410340414

PAVLIDES C, 1993, J NEUROPHYSIOL, V70, P733, DOI 10.1152/jn.1993.70.2.733

Piek JP, 1999, J INT NEUROPSYCH SOC, V5, P320, DOI 10.1017/S1355617799544032

Polatajko Helene J, 2005, Semin Pediatr Neurol, V12, P250

Querne L, 2008, BRAIN RES, V1244, P89, DOI 10.1016/j.brainres.2008.07.066

Reuter-Lorenz PA, 2000, J COGNITIVE NEUROSCI, V12, P174, DOI 10.1162/089892900561814

REVIE G, 1993, ADAPT PHYS ACT Q, V10, P29, DOI DOI 10.1123/APAQ.10.1.29

Sakai K, 1998, J NEUROSCI, V18, P1827

Schmahmann JD, 1999, NEUROIMAGE, V10, P233, DOI 10.1006/nimg.1999.0459

Seidler RD, 2002, SCIENCE, V296, P2043, DOI 10.1126/science.1068524

Silk T, 2005, BRIT J PSYCHIAT, V187, P282, DOI 10.1192/bjp.187.3.282

Stoodley CJ, 2009, NEUROIMAGE, V44, P489, DOI 10.1016/j.neuroimage.2008.08.039

Talairach J., 1988, COPLANAR STEREOTAXIC

THOMAS JR, 1991, RES Q EXERCISE SPORT, V62, P344, DOI 10.1080/02701367.1991.10608733

Thoroughman KA, 1999, J NEUROSCI, V19, P8573

Toni I, 1998, NEUROIMAGE, V8, P50, DOI 10.1006/nimg.1998.0349

Vidoni ED, 2010, NEUROBIOL LEARN MEM, V93, P532, DOI 10.1016/j.nlm.2010.01.011

Vidoni ED, 2009, BEHAV BRAIN FUNCT, V5, DOI 10.1186/1744-9081-5-36

Williams J, 2006, CHILD CARE HLTH DEV, V32, P633, DOI 10.1111/j.1365-2214.2006.00688.x

Wilson B. N., 2007, DEV COORDINATION QUE

Wilson PH, 2004, DEV MED CHILD NEUROL, V46, P754, DOI 10.1017/S001216220400129X

Wilsonl PH, 2001, HUM MOVEMENT SCI, V20, P135, DOI 10.1016/S0167-9457(01)00032-X

Zwicker JG, 2010, PEDIATRICS, V126, pE678, DOI 10.1542/peds.2010-0059

Zwicker JG, 2009, J CHILD NEUROL, V24, P1273, DOI 10.1177/0883073809333537

NR 73

TC 127

Z9 133

U1 2

U2 54

PU PERGAMON-ELSEVIER SCIENCE LTD

PI OXFORD

PA THE BOULEVARD, LANGFORD LANE, KIDLINGTON, OXFORD OX5 1GB, ENGLAND

SN 0736-5748

EI 1873-474X

J9 INT J DEV NEUROSCI

JI Int. J. Dev. Neurosci.

PD APR

PY 2011

VL 29

IS 2

BP 145

EP 152

DI 10.1016/j.ijdevneu.2010.12.002

PG 8

WC Developmental Biology; Neurosciences

WE Science Citation Index Expanded (SCI-EXPANDED)

SC Developmental Biology; Neurosciences & Neurology

GA 735IT

UT WOS:000288408200006

PM 21145385

DA 2022-08-17

ER

PT J

AU Leonard, HC

Bernardi, M

Hill, EL

Henry, LA

AF Leonard, Hayley C.

Bernardi, Marialivia

Hill, Elisabeth L.

Henry, Lucy A.

TI Executive Functioning, Motor Difficulties, and Developmental

Coordination Disorder

SO DEVELOPMENTAL NEUROPSYCHOLOGY

LA English

DT Article

ID WORKING-MEMORY; LANGUAGE IMPAIRMENT; AGED CHILDREN; PERFORMANCE;

DYSFUNCTION; ATTENTION; AUTISM; DCD; QUESTIONNAIRE; METAANALYSIS

AB The current study assessed a comprehensive range of executive functions (EFs) in children with poor motor skills, comparing profiles of children with a diagnosis of developmental coordination disorder (DCD) and those identified with motor difficulties (MD). Children in both groups performed more poorly than typically developing controls on nonverbal measures of working memory, inhibition, planning, and fluency, but not on tests of switching. The similar patterns of strengths and weaknesses in children with MD and DCD have important implications for parents, teachers, and clinicians, as children with MD may struggle with EF tasks even though their motor difficulties are not identified.

C1 [Leonard, Hayley C.; Hill, Elisabeth L.] Univ London, Dept Psychol, Goldsmiths, London SE14 6NW, England.

[Bernardi, Marialivia; Henry, Lucy A.] City Univ London, Div Language & Commun Sci, London EC1V 0HB, England.

RP Leonard, HC (通讯作者)，Univ London, Dept Psychol, Goldsmiths, London SE14 6NW, England.

EM h.leonard@gold.ac.uk

OI Hill, Elisabeth/0000-0003-3130-1271; Bernardi,

Marialivia/0000-0002-6844-0358

FU Waterloo Foundation [1121/1555]

FX This research was supported by a grant from The Waterloo Foundation to

L. Henry (Ref: 1121/1555).

CR Achenbach T. M., 1991, MANUAL CHILD BEHAV C

Alloway TP, 2007, J EXP CHILD PSYCHOL, V96, P20, DOI 10.1016/j.jecp.2006.07.002

Alloway TP, 2011, CHILD NEUROPSYCHOL, V17, P483, DOI 10.1080/09297049.2011.553590

Alloway TP, 2008, J LEARN DISABIL-US, V41, P251, DOI 10.1177/0022219408315815

American Psychiatric Association, 2013, DIAGN STAT MAN MENT, V5th edition, DOI [10.1176/appi.books.9780890425596, DOI 10.1176/APPI.BOOKS.9780890425596]

American Psychological Association, 2000, DIAGN STAT MAN MENT, DOI DOI 10.1176/APPI.BOOKS.9780890423349

Asonitou K, 2010, PROCD SOC BEHV, V5, P1702, DOI 10.1016/j.sbspro.2010.07.350

Best JR, 2011, LEARN INDIVID DIFFER, V21, P327, DOI 10.1016/j.lindif.2011.01.007

Best JR, 2010, CHILD DEV, V81, P1641, DOI 10.1111/j.1467-8624.2010.01499.x

Bishop DVM, 2002, AM J MED GENET, V114, P56, DOI 10.1002/ajmg.1630

Cambridge Cognition, 2006, CAMBR NEUR TEST AUT

Castellanos FX, 2006, TRENDS COGN SCI, V10, P117, DOI 10.1016/j.tics.2006.01.011

Delis D.C., 2001, DELIS KAPLAN EXECUTI

Dewey D, 2002, HUM MOVEMENT SCI, V21, P905, DOI 10.1016/S0167-9457(02)00163-X

Diamond A, 2000, CHILD DEV, V71, P44, DOI 10.1111/1467-8624.00117

Diamond A, 2013, ANNU REV PSYCHOL, V64, P135, DOI 10.1146/annurev-psych-113011-143750

Elliot C.D., 2011, BRIT ABILITY SCALES, V3rd Edn

Field A., 2018, DISCOVERING STAT USI

Friedman NP, 2006, PSYCHOL SCI, V17, P172, DOI 10.1111/j.1467-9280.2006.01681.x

Geuze Reint H., 2005, Neural Plasticity, V12, P183, DOI 10.1155/NP.2005.183

Goodman R, 1997, J CHILD PSYCHOL PSYC, V38, P581, DOI 10.1111/j.1469-7610.1997.tb01545.x

Goodman R, 1999, J ABNORM CHILD PSYCH, V27, P17, DOI 10.1023/A:1022658222914

Henderson S., 2007, MOVEMENT ASSESSMENT

Henry LA, 2001, MEMORY, V9, P233, DOI 10.1080/09658210042000085

Henry LA, 2012, J CHILD PSYCHOL PSYC, V53, P37, DOI 10.1111/j.1469-7610.2011.02430.x

Henry LA, 2010, CHILD ADOL MENT H-UK, V15, P110, DOI 10.1111/j.1475-3588.2010.00557.x

Hill EL, 2004, TRENDS COGN SCI, V8, P26, DOI 10.1016/j.tics.2003.11.003

Isquith PK, 2005, MENT RETARD DEV D R, V11, P209, DOI 10.1002/mrdd.20075

Johnson MH, 2012, TRENDS COGN SCI, V16, P454, DOI 10.1016/j.tics.2012.07.001

Kadesjo B, 1999, J AM ACAD CHILD PSY, V38, P820, DOI 10.1097/00004583-199907000-00011

Kirby A, 2008, DYSLEXIA, V14, P197, DOI 10.1002/dys.367

Kirby A, 2005, BRIT J SPEC EDUC, V32, P122, DOI 10.1111/j.0952-3383.2005.00384.x

Lingam R, 2009, PEDIATRICS, V123, pE693, DOI 10.1542/peds.2008-1770

Luna B, 2008, BRAIN COGNITION, V68, P293, DOI 10.1016/j.bandc.2008.08.019

Mandich A, 2002, BRAIN COGNITION, V50, P150, DOI 10.1016/S0278-2626(02)00020-9

Michel E, 2011, CHILD NEUROPSYCHOL, V17, P151, DOI 10.1080/09297049.2010.525501

Miyake A, 2000, COGNITIVE PSYCHOL, V41, P49, DOI 10.1006/cogp.1999.0734

Pennington BF, 1996, J CHILD PSYCHOL PSYC, V37, P51, DOI 10.1111/j.1469-7610.1996.tb01380.x

Pickering S. J., 2001, WORKING MEMORY TEST

Piek JP, 2007, DEV MED CHILD NEUROL, V49, P678, DOI 10.1111/j.1469-8749.2007.00678.x

Piek JP, 2004, ARCH CLIN NEUROPSYCH, V19, P1063, DOI 10.1016/j.acn.2003.12.007

Pratt ML, 2014, RES DEV DISABIL, V35, P1579, DOI 10.1016/j.ridd.2014.04.008

Querne L, 2008, BRAIN RES, V1244, P89, DOI 10.1016/j.brainres.2008.07.066

Rubia K, 2006, HUM BRAIN MAPP, V27, P973, DOI 10.1002/hbm.20237

Semel E. M., 2006, CLIN EVALUATION LANG, V4th

St Clair-Thompson HL, 2006, Q J EXP PSYCHOL, V59, P745, DOI 10.1080/17470210500162854

TORGENSEN JK, 1999, TEST WORD READING EF

van Swieten LM, 2010, J EXP PSYCHOL HUMAN, V36, P493, DOI 10.1037/a0017177

Wilmut K, 2010, DEV MED CHILD NEUROL, V52, P229, DOI 10.1111/j.1469-8749.2009.03372.x

Wilson PH, 2013, DEV MED CHILD NEUROL, V55, P217, DOI 10.1111/j.1469-8749.2012.04436.x

Wilson PH, 1998, J CHILD PSYCHOL PSYC, V39, P829, DOI 10.1111/1469-7610.00384

Wisdom SN, 2007, EUR CHILD ADOLES PSY, V16, P178, DOI 10.1007/s00787-006-0586-8

Wuang YP, 2011, RES DEV DISABIL, V32, P1669, DOI 10.1016/j.ridd.2011.02.021

Zwicker JG, 2011, INT J DEV NEUROSCI, V29, P145, DOI 10.1016/j.ijdevneu.2010.12.002

NR 54

TC 55

Z9 57

U1 5

U2 40

PU ROUTLEDGE JOURNALS, TAYLOR & FRANCIS LTD

PI ABINGDON

PA 4 PARK SQUARE, MILTON PARK, ABINGDON OX14 4RN, OXFORDSHIRE, ENGLAND

SN 8756-5641

EI 1532-6942

J9 DEV NEUROPSYCHOL

JI Dev. Neuropsychol.

PD MAY 19

PY 2015

VL 40

IS 4

BP 201

EP 215

DI 10.1080/87565641.2014.997933

PG 15

WC Psychology, Developmental; Psychology; Psychology, Experimental

WE Science Citation Index Expanded (SCI-EXPANDED); Social Science Citation Index (SSCI)

SC Psychology

GA CN0WL

UT WOS:000358134600001

PM 26179487

OA Green Accepted

DA 2022-08-17

ER

PT J

AU Fliers, EA

de Hoog, MLA

Franke, B

Faraone, SV

Rommelse, NNJ

Buitelaar, JK

Nijhuis-van der Sanden, MWG

AF Fliers, Ellen A.

de Hoog, Marieke L. A.

Franke, Barbara

Faraone, Stephen V.

Rommelse, Nanda N. J.

Buitelaar, Jan K.

Nijhuis-van der Sanden, Maria W. G.

TI Actual Motor Performance and Self-Perceived Motor Competence in Children

With Attention-Deficit Hyperactivity Disorder Compared With Healthy

Siblings and Peers

SO JOURNAL OF DEVELOPMENTAL AND BEHAVIORAL PEDIATRICS

LA English

DT Article

DE ADHD; motor performance; MABC; self-perception; DCD

ID FUNDAMENTAL MOVEMENT SKILLS; PHYSICAL-ACTIVITY; ADHD; COORDINATION;

QUESTIONNAIRE; ADOLESCENTS; PERCEPTION; AGE; DCD

AB Objective: Children with attention-deficit hyperactivity disorder (ADHD) frequently experience comorbid motor problems, developmental coordination disorder. Also, children with ADHD are said to overestimate their abilities in the cognitive and social domain, the so-called "Positive illusory Bias." In this cross-sectional study, the relationship between actual motor performance and perceived motor competence was examined. Method: Motor performance was assessed using the Movement Assessment Battery for Children in 100 children and adolescents (age 6-17 years), including 32 children with ADHD combined type, 18 unaffected siblings, and 50 healthy control children. ADHD was diagnosed using Parent and Teacher questionnaires and a clinical interview. Perceived motor competence and interest in the motor domain were rated with the Dutch supplement scale to Harters' Self-Perception Profile for Children, especially focusing on the motor domain (m-CBSK). Results: Children with ADHD had poorer motor performance than unaffected siblings and control children, especially in the field of manual dexterity. However, no relationship was found between motor performance and perceived motor competence. Only children with the very lowest motor performance had a significantly lowered perception of their motor competence. Interest in the motor domain and motor self-perception was positively correlated. Conclusion: Children with ADHD performed poorer on the Movement Assessment Battery for Children, but generally overestimated their own motor competence.

C1 [Fliers, Ellen A.] Parnassia BAVO Grp, Lucertis, NL-3089 BD Rotterdam, Netherlands.

[Fliers, Ellen A.; Franke, Barbara; Buitelaar, Jan K.] Radboud Univ Nijmegen, Med Ctr, Dept Psychiat, Donders Inst Brain Cognit & Behav, NL-6525 ED Nijmegen, Netherlands.

[de Hoog, Marieke L. A.; Nijhuis-van der Sanden, Maria W. G.] Radboud Univ Nijmegen, Med Ctr, Dept Paediat Phys Therapy, NL-6525 ED Nijmegen, Netherlands.

[Franke, Barbara] Radboud Univ Nijmegen, Med Ctr, Dept Human Genet, NL-6525 ED Nijmegen, Netherlands.

[Faraone, Stephen V.] SUNY Upstate Med Univ, Dept Psychiat, Syracuse, NY USA.

[Faraone, Stephen V.] SUNY Upstate Med Univ, Dept Neurosci & Physiol, Syracuse, NY USA.

[Buitelaar, Jan K.] Karakter Child & Adolescent Univ Ctr, Nijmegen, Netherlands.

RP Fliers, EA (通讯作者)，Parnassia BAVO Grp, Lucertis, Twentestr 52, NL-3089 BD Rotterdam, Netherlands.

EM E.Fliers@psy.umcn.nl

RI Faraone, Stephen V/O-7239-2018; Rommelse, Nanda NJ/D-4872-2009; Franke,

Barbara/D-4836-2009; Buitelaar, Jan K/E-4584-2012; Faraone,

Stephen/AAE-7334-2019; Nijhuis-van der Sanden, Maria WG/B-3463-2012

OI Faraone, Stephen V/0000-0002-9217-3982; Rommelse, Nanda

NJ/0000-0002-1711-0359; Franke, Barbara/0000-0003-4375-6572; Buitelaar,

Jan K/0000-0001-8288-7757; Faraone, Stephen/0000-0002-9217-3982;

Nijhuis-van der Sanden, Maria WG/0000-0003-2637-6877

FU NATIONAL INSTITUTE OF MENTAL HEALTH [R01MH062873] Funding Source: NIH

RePORTER; NIMH NIH HHS [R01MH62873, R01 MH062873, R01 MH062873-01A1]

Funding Source: Medline

CR Barber Sara, 2005, J Pediatr Nurs, V20, P235

Berument SK, 1999, BRIT J PSYCHIAT, V175, P444, DOI 10.1192/bjp.175.5.444

Brookes K, 2006, MOL PSYCHIATR, V11, P934, DOI 10.1038/sj.mp.4001869

Conners C. K, 2003, CONNERS RATING SCALE

Fisher A, 2005, MED SCI SPORT EXER, V37, P684, DOI 10.1249/01.MSS.0000159138.48107.7D

Fliers E, 2008, J NEURAL TRANSM, V115, P211, DOI 10.1007/s00702-007-0827-0

Fliers E, 2009, J AM ACAD CHILD PSY, V48, P25, DOI 10.1097/CHI.0b013e31818b1ca2

Gillberg C, 2003, ARCH DIS CHILD, V88, P904, DOI 10.1136/adc.88.10.904

Goodman R, 1997, J CHILD PSYCHOL PSYC, V38, P581, DOI 10.1111/j.1469-7610.1997.tb01545.x

Harter S, 1985, MANUAL SELF PERCEPTI

Harvey WJ, 2007, J ABNORM CHILD PSYCH, V35, P871, DOI 10.1007/s10802-007-9140-5

HENDERSON SF, 1992, MANUAL MOVEMENT ASSE

Hoza B, 2004, J CONSULT CLIN PSYCH, V72, P382, DOI 10.1037/0022-006X.72.3.382

Hoza B, 2002, J ABNORM PSYCHOL, V111, P268, DOI 10.1037//0021-843X.111.2.268

Kadesjo B, 2001, J CHILD PSYCHOL PSYC, V42, P487, DOI 10.1111/1469-7610.00742

Kuntsi Jonna, 2006, Behav Brain Funct, V2, P27, DOI 10.1186/1744-9081-2-27

LOSSE A, 1991, DEV MED CHILD NEUROL, V33, P55, DOI 10.1111/j.1469-8749.1991.tb14785.x

Martin NC, 2006, HUM MOVEMENT SCI, V25, P110, DOI 10.1016/j.humov.2005.10.006

Nijhuis-van der Sanden RWG, 2000, DEV MED CHILD NEUROL, V42, P685, DOI 10.1017/S0012162200001262

Piek JP, 2004, ARCH CLIN NEUROPSYCH, V19, P1063, DOI 10.1016/j.acn.2003.12.007

Pitcher TM, 2003, DEV MED CHILD NEUROL, V45, P525, DOI 10.1111/j.1469-8749.2003.tb00952.x

Raudsepp L, 2002, PERCEPT MOTOR SKILL, V94, P1059, DOI 10.2466/PMS.94.4.1059-1070

Raustorp A, 2005, SCAND J MED SCI SPOR, V15, P126, DOI 10.1111/j.1600-0838.2004.406.x

ROSSUM JHA, 2000, MANUAL SUPPLELMENT C

Schoemaker MM, 2003, BRIT J EDUC PSYCHOL, V73, P425, DOI 10.1348/000709903322275911

Sergeant JA, 2006, HUM MOVEMENT SCI, V25, P76, DOI 10.1016/j.humov.2005.10.007

Skinner RA, 2001, HUM MOVEMENT SCI, V20, P73, DOI 10.1016/S0167-9457(01)00029-X

TAYLOR E, 1986, BRIT J PSYCHIAT, V149, P760, DOI 10.1192/bjp.149.6.760

NR 28

TC 33

Z9 35

U1 0

U2 40

PU LIPPINCOTT WILLIAMS & WILKINS

PI PHILADELPHIA

PA TWO COMMERCE SQ, 2001 MARKET ST, PHILADELPHIA, PA 19103 USA

SN 0196-206X

EI 1536-7312

J9 J DEV BEHAV PEDIATR

JI J. Dev. Behav. Pediatr.

PD JAN

PY 2010

VL 31

IS 1

BP 35

EP 40

DI 10.1097/DBP.0b013e3181c7227e

PG 6

WC Behavioral Sciences; Psychology, Developmental; Pediatrics

WE Science Citation Index Expanded (SCI-EXPANDED); Social Science Citation Index (SSCI)

SC Behavioral Sciences; Psychology; Pediatrics

GA 545QG

UT WOS:000273750800005

PM 20081434

OA Green Accepted

DA 2022-08-17

ER

PT J

AU Sartori, RF

Valentini, NC

Fonseca, RP

AF Sartori, Rodrigo Flores

Valentini, Nadia Cristina

Fonseca, Rochele Paz

TI Executive function in children with and without developmental

coordination disorder: A comparative study

SO CHILD CARE HEALTH AND DEVELOPMENT

LA English

DT Article

DE developmental coordination disorder; executive functions; motor

impairments

ID 5 DIGIT TEST; WORKING-MEMORY; MOTOR-PERFORMANCE; ATTENTION; DCD;

DIFFICULTIES; SEVERITY; MOVEMENT; LANGUAGE; DEFICITS

AB Background Children with motor impairments also show poor performance in some executive functions' components. However, there is no consensus on which specific executive subdomain is more impacted. Aim The objective of this study was to compare executive functions in children with developmental coordination disorder (DCD), at risk for DCD (r-DCD), and in typically developing (TD) children. Methods and Procedures A sample of 397 children was assessed using the MABC-2. Two groups of children were identified; DCD (n = 63) and at r-DCD (n = 31). A third matched group of children with TD (n = 63) was formed. The MABC-2 checklist and the WASI tests were used as screening tools. Measures of executive function including verbal and nonverbal tasks for working memory, inhibitory control, and cognitive flexibility were tested. Multivariate analysis of variance followed by analyses of variance and Bonferroni tests were used to verify group effects on executive functions. Results A significant group effects were found for Working Memory, ? = .78, F(4, 360) = 10.12, p <= .001, eta(2)(p) = .12; Inhibitory Control, ? = .59, F(16, 294) = 5.48, p <= .001, eta(2)(p) = .23; and Cognitive Flexibility and Inhibitory Control, ? = .60, F(22, 288) = 3.74, p <= .001, eta(2)(p) = .22, with moderate effect sizes. The DCD group showed lower scores compared with the TD group on the visuospatial and verbal working memory; inhibitory control and in tasks of cognitive flexibility; the r-DCD group showed lower scores compared with the TD group for visuospatial working memory and for cognitive flexibility. Conclusions and Implications Poor performance in several measures of executive functions in children with DCD emphasized the need of motor/executive task-specific interventions. Furthermore, children at r-DCD showed low scores in several executive functions; therefore, preventive services should also be provided for this subclinical group.

C1 [Sartori, Rodrigo Flores; Fonseca, Rochele Paz] Pontificia Univ Catolica Rio Grande do Sul, Sch Hlth & Life Sci, Porto Alegre, RS, Brazil.

[Sartori, Rodrigo Flores] Serra Gaucha Univ, Dept Phys Educ, Porto Alegre, RS, Brazil.

[Valentini, Nadia Cristina] Univ Fed Rio Grande do Sul, Dept Phys Educ, Sch Phys Educ Physiotherapy & Dance, Porto Alegre, RS, Brazil.

RP Sartori, RF (通讯作者)，Pontificia Univ Catolica Rio Grande do Sul, Sch Hlth & Life Sci, Porto Alegre PUCRS, 6681 Ipiranga Ave,Bldg 80, BR-90619900 Porto Alegre, RS, Brazil.

EM rodrigo.sartori@pucrs.br

RI valentini, nadia/AAY-9815-2020; Valentini, Nadia Cristina/I-5520-2013

OI Valentini, Nadia Cristina/0000-0001-6412-5206; Sartori,

Rodrigo/0000-0001-7843-0199

CR Alesi M, 2019, EUR J SPEC NEEDS EDU, V34, P285, DOI 10.1080/08856257.2018.1468635

Alloway TP, 2007, J EXP CHILD PSYCHOL, V96, P20, DOI 10.1016/j.jecp.2006.07.002

Alloway TP, 2007, APPL COGNITIVE PSYCH, V21, P473, DOI 10.1002/acp.1284

American Psychiatric Association, 2013, DIAGNOSTIC STAT MANU, DOI [10.1176/appi.books.9780890425596, DOI 10.1176/APPI.BOOKS.9780890425596]

Asonitou K, 2012, RES DEV DISABIL, V33, P996, DOI 10.1016/j.ridd.2012.01.008

Bernardi M, 2018, DEV MED CHILD NEUROL, V60, P306, DOI 10.1111/dmcn.13640

Best JR, 2010, DEV REV, V30, P331, DOI 10.1016/j.dr.2010.08.001

Brown-Lum M., 2015, CURR DEV DISORD REP, V2, P131, DOI [10.1007/s40474-015-0046-6, DOI 10.1007/S40474-015-0046-6]

Cairney J, 2013, DEV REV, V33, P224, DOI 10.1016/j.dr.2013.07.002

Chen WY, 2012, RES DEV DISABIL, V33, P1516, DOI 10.1016/j.ridd.2012.03.005

Cohen J., 1988, STAT POWER ANAL SOCI

Ramalho MHD, 2013, MOTRIZ, V19, P423

Paiva GCD, 2016, ARQ NEURO-PSIQUIAT, V74, P29, DOI 10.1590/0004-282X20150184

Diamond A, 2000, CHILD DEV, V71, P44, DOI 10.1111/1467-8624.00117

Diamond A., 2012, NEUROPSYCHOL REV, V4, P1, DOI [10.1146/annurev-psych-113011-143750, DOI 10.1146/ANNUREV-PSYCH-113011-143750]

Diamond A, 2007, SCIENCE, V318, P1387, DOI 10.1126/science.1151148

Diamond A, 2012, CURR DIR PSYCHOL SCI, V21, P335, DOI 10.1177/0963721412453722

Diamond A, 2013, ANNU REV PSYCHOL, V64, P135, DOI 10.1146/annurev-psych-113011-143750

Dyck M, 2010, INT J LANG COMM DIS, V45, P336, DOI 10.3109/13682820903009503

Fonseca R.P., 2010, AVALIA O PSICOL GICA, P337

Fonseca Rochele Paz, 2008, Psychol. Neurosci., V1, P55, DOI 10.1590/S1983-32882008000100009

Gomez A, 2015, RES DEV DISABIL, V43-44, P167, DOI 10.1016/j.ridd.2015.06.011

Henderson S. E., 2007, 2 EDITION MOVEMENT A

Henry LA, 2001, MEMORY, V9, P233, DOI 10.1080/09658210042000085

Holden C, 2004, SCIENCE, V303, P1316, DOI 10.1126/science.303.5662.1316

Houwen S, 2017, HUM MOVEMENT SCI, V53, P24, DOI 10.1016/j.humov.2016.12.009

Ito M, 2008, NAT REV NEUROSCI, V9, P304, DOI 10.1038/nrn2332

Karras HC, 2019, RES DEV DISABIL, V84, P85, DOI 10.1016/j.ridd.2018.05.012

Koziol LF, 2014, CEREBELLUM, V13, P151, DOI 10.1007/s12311-013-0511-x

Koziol LF, 2013, APPL NEUROPSYCH-CHIL, V2, P104, DOI 10.1080/21622965.2013.748386

Leonard H. C., 2015, CURR DEV DISORD REP, V2, P141, DOI [10.1007/s40474-015-0044-8, DOI 10.1007/S40474-015-0044-8]

Leonard HC, 2015, DEV NEUROPSYCHOL, V40, P201, DOI 10.1080/87565641.2014.997933

Livesey D, 2006, HUM MOVEMENT SCI, V25, P50, DOI 10.1016/j.humov.2005.10.008

Mandich A, 2003, BRAIN COGNITION, V51, P346, DOI 10.1016/S0278-2626(03)00039-3

Mandich A, 2002, BRAIN COGNITION, V50, P150, DOI 10.1016/S0278-2626(02)00020-9

Maroco J, 2014, ANALISE ESTATISTICA

Maroco J, 2010, ANALISE EQUACOES EST, V2<feminine

Michel E, 2011, CHILD NEUROPSYCHOL, V17, P151, DOI 10.1080/09297049.2010.525501

Missiuna C, 2014, RES DEV DISABIL, V35, P1198, DOI 10.1016/j.ridd.2014.01.007

Miyake A, 2012, CURR DIR PSYCHOL SCI, V21, P8, DOI 10.1177/0963721411429458

Montiel J. M., 2012, AVALIACAO NEUROPSICO

Pesce C, 2016, FRONT PSYCHOL, V7, DOI 10.3389/fpsyg.2016.00349

Piek J. P., 2007, WORKING MEMORY PROCE, P678

Piek JP, 2007, DEV MED CHILD NEUROL, V49, P678, DOI 10.1111/j.1469-8749.2007.00678.x

Piek JP, 2004, ARCH CLIN NEUROPSYCH, V19, P1063, DOI 10.1016/j.acn.2003.12.007

Pieters S, 2012, RES DEV DISABIL, V33, P1128, DOI 10.1016/j.ridd.2012.02.007

Querne L, 2008, BRAIN RES, V1244, P89, DOI 10.1016/j.brainres.2008.07.066

Sartori R. F., 2018, N AM SOC PSYCHOL SPO, V40, P60

Schott N, 2015, J INTELL DISABIL RES, V59, P860, DOI 10.1111/jir.12189

Sedo MA, 2004, REV NEUROLOGIA, V38, P824, DOI 10.33588/rn.3809.2003545

Siqueira Larissa de Souza, 2016, Trends Psychiatry Psychother., V38, P164, DOI 10.1590/2237-6089-2016-0019

Smits-Engelsman B, 2015, HUM MOVEMENT SCI, V42, P293, DOI 10.1016/j.humov.2015.03.010

Toussaint-Thorin M., 2013, Annals of Physical and Rehabilitation Medicine, V56, P268, DOI 10.1016/j.rehab.2013.02.006

Tsai CL, 2014, ARCH CLIN NEUROPSYCH, V29, P173, DOI 10.1093/arclin/act081

Tsai CL, 2012, BRAIN COGNITION, V79, P12, DOI 10.1016/j.bandc.2012.02.004

Tsai CL, 2010, RES DEV DISABIL, V31, P642, DOI 10.1016/j.ridd.2010.01.003

Tsai CL, 2009, BRAIN COGNITION, V71, P246, DOI 10.1016/j.bandc.2009.08.006

Tsai CL, 2009, RES DEV DISABIL, V30, P1268, DOI 10.1016/j.ridd.2009.05.001

Vaivre-Douret L, 2014, NEUROPHYSIOL CLIN, V44, P13, DOI 10.1016/j.neucli.2013.10.133

Valentini NC, 2015, CHILD CARE HLTH DEV, V41, P970, DOI 10.1111/cch.12219

Wechsler D., 2011, WECHSLER ABBREVIATED, VSecond, pP

Wilson PH, 2017, DEV MED CHILD NEUROL, V59, P1117, DOI 10.1111/dmcn.13530

Wuang YP, 2011, RES DEV DISABIL, V32, P1669, DOI 10.1016/j.ridd.2011.02.021

Xue G, 2008, CEREB CORTEX, V18, P1923, DOI 10.1093/cercor/bhm220

Zwicker JG, 2012, PEDIATR NEUROL, V46, P162, DOI 10.1016/j.pediatrneurol.2011.12.007

NR 65

TC 9

Z9 10

U1 7

U2 30

PU WILEY

PI HOBOKEN

PA 111 RIVER ST, HOBOKEN 07030-5774, NJ USA

SN 0305-1862

EI 1365-2214

J9 CHILD CARE HLTH DEV

JI Child Care Health Dev.

PD MAY

PY 2020

VL 46

IS 3

BP 294

EP 302

DI 10.1111/cch.12734

EA JAN 2020

PG 9

WC Psychology, Developmental; Pediatrics

WE Science Citation Index Expanded (SCI-EXPANDED); Social Science Citation Index (SSCI)

SC Psychology; Pediatrics

GA KY6EM

UT WOS:000509104200001

PM 31845379

DA 2022-08-17

ER

PT J

AU Michel, E

Kauer, M

Roebers, CM

AF Michel, Eva

Kauer, Marianne

Roebers, Claudia M.

TI Motor coordination impairments in childhood: The role of individual

differences in cognitive functions

SO KINDHEIT UND ENTWICKLUNG

LA German

DT Article

DE developmental coordination disorder; coordination; executive functions;

attention; working memory

ID SPECIAL-NEEDS CLASSES; WORKING-MEMORY; 6-YEAR-OLD CHILDREN; EXECUTIVE

FUNCTION; DISORDER DCD; PERFORMANCE; ABILITY; ADOLESCENCE; INHIBITION;

PREDICTORS

AB The aim of the present study was to explore cognitive functions in children with poor motor coordination and in children at risk for developmental coordination disorder. A total of 179 children aged 7 years were tested with a motor test battery (M-ABC-2), and completed several attention and working memory tasks with executive demands. Thirty-four children showed motor coordination performance below average, 8 children were identified as being at risk for developmental coordination disorder. Results revealed that children with motor coordination below average reacted slower but not less accurately in the attention tasks compared to children with normative motor coordination. In contrast, children at risk for developmental coordination disorder showed lower accuracy in attentional control tasks. Working memory was not associated with motor coordination. Results are discussed in terms of processes underlying cognitive problems related to developmental coordination disorder.

C1 [Michel, Eva] Univ Wurzburg, Lehrstuhl Psychol 4, D-97070 Wurzburg, Germany.

[Kauer, Marianne] PH Bern, Inst Vorschulstufe & Primarstufe, CH-3005 Bern, Switzerland.

[Roebers, Claudia M.] Univ Bern, Abt Entwicklungspsychol, Inst Psychol, CH-3000 Bern 9, Switzerland.

RP Michel, E (通讯作者)，Univ Wurzburg, Lehrstuhl Psychol 4, Rontgenring 10, D-97070 Wurzburg, Germany.

EM eva.michel@uni-wuerzburg.de

CR Achenbach T. M., 1991, MANUAL CHILD BEHAV C

Alloway TP, 2005, BRIT J DEV PSYCHOL, V23, P417, DOI 10.1348/026151005X26804

Alloway TP, 2007, J EXP CHILD PSYCHOL, V96, P20, DOI 10.1016/j.jecp.2006.07.002

Alloway TP, 2007, APPL COGNITIVE PSYCH, V21, P473, DOI 10.1002/acp.1284

Alloway TP, 2008, J LEARN DISABIL-US, V41, P251, DOI 10.1177/0022219408315815

Bull R, 2001, DEV NEUROPSYCHOL, V19, P273, DOI 10.1207/S15326942DN1903_3

Davidson MC, 2006, NEUROPSYCHOLOGIA, V44, P2037, DOI 10.1016/j.neuropsychologia.2006.02.006

Dewey D, 2002, HUM MOVEMENT SCI, V21, P905, DOI 10.1016/S0167-9457(02)00163-X

Diamond A, 2007, SCIENCE, V318, P1387, DOI 10.1126/science.1151148

ERIKSEN BA, 1974, PERCEPT PSYCHOPHYS, V16, P143, DOI 10.3758/BF03203267

Gathercole SE, 2006, J EXP CHILD PSYCHOL, V93, P265, DOI 10.1016/j.jecp.2005.08.003

Geary DC, 2001, APHASIOLOGY, V15, P635, DOI 10.1080/02687040143000113

Geuze RH, 2001, HUM MOVEMENT SCI, V20, P7, DOI 10.1016/S0167-9457(01)00027-6

Henderson S. E., 2007, M ABC 2 MOVEMENT ASS

Hughes C., 2002, CHILD ADOLESCENT MEN, V7, P131, DOI DOI 10.1111/1475-3588.00024

KAIL R, 1991, DEV PSYCHOL, V27, P259, DOI 10.1037/0012-1649.27.2.259

Kail R. V, 2007, CHILDREN THEIR DEV

Kaplan BJ, 1998, HUM MOVEMENT SCI, V17, P471, DOI 10.1016/S0167-9457(98)00010-4

Kastner J, 2009, PSYCHOL RUNDSCH, V60, P73, DOI 10.1026/0033-3042.60.2.73

Lamm C, 2006, NEUROPSYCHOLOGIA, V44, P2139, DOI 10.1016/j.neuropsychologia.2005.10.013

Lee K, 2004, J EXP CHILD PSYCHOL, V89, P140, DOI 10.1016/j.jecp.2004.07.001

Livesey D, 2006, HUM MOVEMENT SCI, V25, P50, DOI 10.1016/j.humov.2005.10.008

Mandich A, 2003, BRAIN COGNITION, V51, P346, DOI 10.1016/S0278-2626(03)00039-3

Mandich A, 2002, BRAIN COGNITION, V50, P150, DOI 10.1016/S0278-2626(02)00020-9

Michel E, 2008, SWISS J PSYCHOL, V67, P249, DOI 10.1024/1421-0185.67.4.249

Parker H. E., 2003, DEV MOVEMENT COORDIN, P107

Petermann F., 2008, MOVEMENT ASSESSMENT

Piek JP, 2004, ARCH CLIN NEUROPSYCH, V19, P1063, DOI 10.1016/j.acn.2003.12.007

Remschmidt H., 2006, MULTIAXIALES KLASSIF

Roebers CM, 2009, DEVELOPMENTAL SCI, V12, P175, DOI 10.1111/j.1467-7687.2008.00755.x

Rothlisberger M, 2009, PRAX KINDERPSYCHOL K, V58, P215

Schmid C, 2008, Z ENTWICKL PADAGOGIS, V40, P2, DOI 10.1026/0049-8637.40.1.2

Schott N., 2004, Z SPORTPSYCHOL, V11, P147, DOI DOI 10.1026/1612-5010.11.4.147

SIMON JR, 1969, J EXP PSYCHOL, V81, P174, DOI 10.1037/h0027448

Visser J, 2003, HUM MOVEMENT SCI, V22, P479, DOI 10.1016/j.humov.2003.09.005

Von Suchodeletz W., 2005, FRUHERKENNUNG ENTWIC, P45

Wassenberg R, 2005, CHILD DEV, V76, P1092, DOI 10.1111/j.1467-8624.2005.00899.x

Wilson PH, 1998, J CHILD PSYCHOL PSYC, V39, P829, DOI 10.1111/1469-7610.00384

Zoelch C, 2005, YOUNG CHILDREN'S COGNITIVE DEVELOPMENT: INTERRELATIONSHIPS AMONG EXECUTIVE FUNCTIONING, WORKING MEMORY, VERBAL ABILITY, AND THEORY OF MIND, P39

NR 39

TC 12

Z9 12

U1 3

U2 18

PU HOGREFE & HUBER PUBLISHERS

PI GOTTINGEN

PA ROHNSWEG 25, D-37085 GOTTINGEN, GERMANY

SN 0942-5403

J9 KINDH ENTWICKL

JI Kindh. Entwickl.

PY 2011

VL 20

IS 1

BP 49

EP 58

DI 10.1026/0942-5403/a000024

PG 10

WC Psychology, Developmental

WE Social Science Citation Index (SSCI)

SC Psychology

GA 687DA

UT WOS:000284753600007

DA 2022-08-17

ER

PT J

AU Rahimi-Golkhandan, S

Piek, JP

Steenbergen, B

Wilson, PH

AF Rahimi-Golkhandan, S.

Piek, J. P.

Steenbergen, B.

Wilson, P. H.

TI Hot executive function in children with Developmental Coordination

Disorder: Evidence for heightened sensitivity to immediate reward

SO COGNITIVE DEVELOPMENT

LA English

DT Article

DE Executive function; Cognitive control; Decision-making; Motor

coordination; Developmental Coordination Disorder (DCD); Motor

development

ID DEFICIT-HYPERACTIVITY DISORDER; DECISION-MAKING; MOTOR COORDINATION;

WORKING-MEMORY; BEHAVIORAL-PROBLEMS; PREFRONTAL CORTEX; ATTENTION;

PERFORMANCE; TASK; ADOLESCENTS

AB Deficits of cool executive function (EF) have been shown in children with motor problems (or Developmental Coordination Disorder - DCD), but little is known of hot EF in this group. Given some evidence of poor self-regulation in DCD, we predicted poorer performance on a measure of hot EF, the Hungry Donkey Task (HDT), relative to typically developing (TD) children. Participants were 14 children with DCD and 22 TD children aged between 6.5 and 12 years. The DCD group performed significantly worse than the TD group on a 100-trial version of the HDT, making more selections from disadvantageous options and less from advantageous ones. Within-group analyses showed that children with DCD had faster responses to disadvantageous options than to advantageous. These results suggest high sensitivity to immediate reward in DCD. This sensitivity may reflect a more generalized deficit in the ability to resist the rewarding aspects of emotionally significant stimuli. (C) 2014 Elsevier Inc. All rights reserved.

C1 [Rahimi-Golkhandan, S.; Steenbergen, B.; Wilson, P. H.] Australian Catholic Univ, Sch Psychol, Melbourne, Vic 3450, Australia.

[Piek, J. P.] Curtin Univ, Sch Psychol & Speech Pathol, Curtin Hlth Innovat Res Inst, Perth, WA 6845, Australia.

[Steenbergen, B.] Radboud Univ Nijmegen, Inst Behav Sci, NL-6525 ED Nijmegen, Netherlands.

RP Wilson, PH (通讯作者)，Australian Catholic Univ, Sch Psychol, 115 Victoria Parade, Melbourne, Vic 3450, Australia.

EM peterh.wilson@acu.edu.au

RI Wilson, Peter H./E-2881-2018

OI Wilson, Peter H./0000-0003-3747-0287; Piek, Jan/0000-0003-3838-6773;

Steenbergen, Bert/0000-0001-8863-2624

CR Alloway TP, 2007, J EXP CHILD PSYCHOL, V96, P20, DOI 10.1016/j.jecp.2006.07.002

American Psychiatric Association, 2013, DIAGN STAT MAN MENT, V5th edition, DOI [10.1176/appi.books.9780890425596, DOI 10.1176/APPI.BOOKS.9780890425596]

American Psychological Association, 2000, DIAGN STAT MAN MENT, DOI DOI 10.1176/APPI.BOOKS.9780890423349

Asonitou K, 2010, PROCD SOC BEHV, V5, P1702, DOI 10.1016/j.sbspro.2010.07.350

Barkley RA, 1997, PSYCHOL BULL, V121, P65, DOI 10.1037/0033-2909.121.1.65

Bechara A, 2004, BRAIN COGNITION, V55, P30, DOI 10.1016/j.bandc.2003.04.001

Bechara A, 2005, CURR OPIN NEUROL, V18, P734, DOI 10.1097/01.wco.0000194141.56429.3c

BECHARA A, 1994, COGNITION, V50, P7, DOI 10.1016/0010-0277(94)90018-3

Bouffard M, 1996, ADAPT PHYS ACT Q, V13, P61, DOI 10.1123/apaq.13.1.61

Cairney J, 2010, CURR OPIN PSYCHIATR, V23, P324, DOI 10.1097/YCO.0b013e32833aa0aa

Casey BJ, 2011, P NATL ACAD SCI USA, V108, P14998, DOI 10.1073/pnas.1108561108

Cassotti M, 2011, CHILD NEUROPSYCHOL, V17, P400, DOI 10.1080/09297049.2010.547463

Cauffman E, 2010, DEV PSYCHOL, V46, P193, DOI 10.1037/a0016128

Collie A, 2002, ANN THORAC SURG, V73, P2005, DOI 10.1016/S0003-4975(01)03375-6

Crone EA, 2005, CHILD NEUROPSYCHOL, V11, P245, DOI 10.1080/092970490911261

Crone EA, 2004, DEV NEUROPSYCHOL, V25, P251, DOI 10.1207/s15326942dn2503_2

Damasio H, 1994, DESCARTES ERROR

Dawson G, 1998, CHILD DEV, V69, P1276, DOI 10.2307/1132265

Dewey D, 2002, HUM MOVEMENT SCI, V21, P905, DOI 10.1016/S0167-9457(02)00163-X

Dinn WM, 2001, BRAIN COGNITION, V46, P114, DOI 10.1016/S0278-2626(01)80046-4

Dolan M, 2013, PSYCHOL MED, V43, P2427, DOI 10.1017/S0033291712003078

Dunn BD, 2006, NEUROSCI BIOBEHAV R, V30, P239, DOI 10.1016/j.neubiorev.2005.07.001

Ernst M, 2002, NEUROPSYCHOPHARMACOL, V26, P682, DOI 10.1016/S0893-133X(01)00414-6

Eslinger PJ, 2004, BRAIN COGNITION, V55, P84, DOI 10.1016/s0278-2626(03)00281-1

Geurts HM, 2006, J ABNORM CHILD PSYCH, V34, P813, DOI 10.1007/s10802-006-9059-2

Geuze RH, 2001, HUM MOVEMENT SCI, V20, P7, DOI 10.1016/S0167-9457(01)00027-6

GRANT DA, 1948, J EXP PSYCHOL, V38, P404, DOI 10.1037/h0059831

Green D, 2006, CHILD CARE HLTH DEV, V32, P741, DOI 10.1111/j.1365-2214.2006.00684.x

Hinson JM, 2002, COGN AFFECT BEHAV NE, V2, P341, DOI 10.3758/CABN.2.4.341

Huizenga HM, 2007, DEVELOPMENTAL SCI, V10, P814, DOI 10.1111/j.1467-7687.2007.00621.x

Johnson MH, 2005, TRENDS COGN SCI, V9, P152, DOI 10.1016/j.tics.2005.01.009

Jokic CS, 2011, EDUC PSYCHOL REV, V23, P75, DOI 10.1007/s10648-010-9148-1

Kanioglou A, 2005, PERCEPT MOTOR SKILL, V101, P163, DOI 10.2466/pms.101.1.163-173

Maruff P, 2009, ARCH CLIN NEUROPSYCH, V24, P165, DOI 10.1093/arclin/acp010

McCarron L. T., 1997, MAND MCCARRON ASSESS

MISCHEL W, 1989, SCIENCE, V244, P933, DOI 10.1126/science.2658056

Missiuna Cheryl, 2008, Can J Occup Ther, V75, P157

Must A, 2006, J AFFECT DISORDERS, V90, P209, DOI 10.1016/j.jad.2005.12.005

Pennequin V, 2010, BRAIN COGNITION, V74, P107, DOI 10.1016/j.bandc.2010.07.003

Piek JP, 2007, TWIN RES HUM GENET, V10, P587, DOI 10.1375/twin.10.4.587

Piek JP, 2007, DEV MED CHILD NEUROL, V49, P678, DOI 10.1111/j.1469-8749.2007.00678.x

Piek JP, 1999, J INT NEUROPSYCH SOC, V5, P320, DOI 10.1017/S1355617799544032

Poulsen AA, 2007, AM J OCCUP THER, V61, P451, DOI 10.5014/ajot.61.4.451

Prencipe A, 2011, J EXP CHILD PSYCHOL, V108, P621, DOI 10.1016/j.jecp.2010.09.008

Riggs NR, 2003, CHILD NEUROPSYCHOL, V9, P267, DOI 10.1076/chin.9.4.267.23513

Rigoli D, 2012, ARCH CLIN NEUROPSYCH, V27, P766, DOI 10.1093/arclin/acs061

Rigoli D, 2012, DEV MED CHILD NEUROL, V54, P1025, DOI 10.1111/j.1469-8749.2012.04403.x

Rigoli D, 2012, PEDIATRICS, V129, pE892, DOI 10.1542/peds.2011-1237

Schmahmann JD, 2006, BRAIN, V129, P290, DOI 10.1093/brain/awh729

Schutter DJLG, 2009, CEREBELLUM, V8, P28, DOI 10.1007/s12311-008-0056-6

Sergeant J, 2000, NEUROSCI BIOBEHAV R, V24, P7, DOI 10.1016/S0149-7634(99)00060-3

SIEGLER RS, 1981, MONOGR SOC RES CHILD, V46, P1, DOI 10.2307/1165995

Smith DG, 2012, DEV PSYCHOL, V48, P1180, DOI 10.1037/a0026342

Somerville LH, 2011, J COGNITIVE NEUROSCI, V23, P2123, DOI 10.1162/jocn.2010.21572

Tan SK, 2001, ADAPT PHYS ACT Q, V18, P168, DOI 10.1123/apaq.18.2.168

Toplak ME, 2005, BEHAV BRAIN FUNCT, V1, DOI 10.1186/1744-9081-1-8

Tseng MH, 2007, INT J REHABIL RES, V30, P327, DOI 10.1097/MRR.0b013e3282f144c7

van den Bos R, 2013, BEHAV BRAIN RES, V238, P95, DOI 10.1016/j.bbr.2012.10.002

Van Duijvenvoorde ACK, 2012, DEV PSYCHOL, V48, P192, DOI 10.1037/a0025601

van Duijvenvoorde ACK, 2010, DEV NEUROPSYCHOL, V35, P539, DOI 10.1080/87565641.2010.494749

Welsh M, 2014, J INT NEUROPSYCH SOC, V20, P152, DOI 10.1017/S1355617713001379

Williams J, 2006, CHILD CARE HLTH DEV, V32, P633, DOI 10.1111/j.1365-2214.2006.00688.x

Wilson PH, 2013, DEV MED CHILD NEUROL, V55, P217, DOI 10.1111/j.1469-8749.2012.04436.x

Wilson PH, 2004, DEV MED CHILD NEUROL, V46, P754, DOI 10.1017/S001216220400129X

Zelazo P. D., 2010, WILEY BLACKWELL HDB, P574, DOI DOI 10.1002/9781444325485.CH22

Zelazo P.D., 2002, HDB CHILDHOOD COGNIT, P445, DOI DOI 10.1002/9780470996652.CH20

Zelazo PD, 2012, CHILD DEV PERSPECT, V6, P354, DOI 10.1111/j.1750-8606.2012.00246.x

Zwicker JG, 2012, EUR J PAEDIATR NEURO, V16, P573, DOI 10.1016/j.ejpn.2012.05.005

NR 68

TC 10

Z9 11

U1 0

U2 26

PU ELSEVIER SCIENCE INC

PI NEW YORK

PA STE 800, 230 PARK AVE, NEW YORK, NY 10169 USA

SN 0885-2014

EI 1879-226X

J9 COGNITIVE DEV

JI Cogn. Dev.

PD OCT-DEC

PY 2014

VL 32

BP 23

EP 37

DI 10.1016/j.cogdev.2014.06.002

PG 15

WC Psychology, Developmental; Psychology, Experimental

WE Social Science Citation Index (SSCI)

SC Psychology

GA AY7WN

UT WOS:000347766800003

DA 2022-08-17

ER

PT J

AU Mountford, HS

Hill, A

Barnett, AL

Newbury, DF

AF Mountford, Hayley S.

Hill, Amanda

Barnett, Anna L.

Newbury, Dianne F.

TI Genome-Wide Association Study of Motor Coordination

SO FRONTIERS IN HUMAN NEUROSCIENCE

LA English

DT Article

DE coordination; development; dyspraxia; neurodevelopment; GWAS; ALSPAC;

developmental coordination disorder; motor coordination

ID COPY-NUMBER VARIATIONS; DISORDER; CHILDREN; CLUMSINESS; GENES; IMPACT;

ADHD; DCD

AB The ability to finely control our movement is key to achieving many of the educational milestones and life-skills we develop throughout our lives. Despite the centrality of coordination to early development, there is a vast gap in our understanding of the underlying biology. Like most complex traits, both genetics and environment influence motor coordination, however, the specific genes, early environmental risk factors and molecular pathways are unknown. Previous studies have shown that about 5% of school-age children experience unexplained difficulties with motor coordination. These children are said to have Developmental Coordination Disorder (DCD). For children with DCD, these motor coordination difficulties significantly impact their everyday life and learning. DCD is associated with poorer academic achievement, reduced quality of life, it can constrain career opportunities and increase the risk of mental health issues in adulthood. Despite the high prevalence of coordination difficulties, many children remain undiagnosed by healthcare professionals. Compounding under-diagnosis in the clinic, research into the etiology of DCD is severely underrepresented in the literature. Here we present the first genome-wide association study to examine the genetic basis of early motor coordination in the context of motor difficulties. Using data from the Avon Longitudinal Study of Parents and Children we generate a derived measure of motor coordination from four components of the Movement Assessment Battery for Children, providing an overall measure of coordination across the full range of ability. We perform the first genome-wide association analysis focused on motor coordination (N = 4542). No single nucleotide polymorphisms (SNPs) met the threshold for genome-wide significance, however, 59 SNPs showed suggestive associations. Three regions contained multiple suggestively associated SNPs, within five preliminary candidate genes: IQSEC1, LRCC1, SYNJ2B2, ADAM20, and ADAM21. Association to the gene IQSEC1 suggests a potential link to axon guidance and dendritic projection processes as a potential underlying mechanism of motor coordination difficulties. This represents an interesting potential mechanism, and whilst further validation is essential, it generates a direct window into the biology of motor coordination difficulties. This research has identified potential biological drivers of DCD, a first step towards understanding this common, yet neglected neurodevelopmental disorder.

C1 [Mountford, Hayley S.; Newbury, Dianne F.] Oxford Brookes Univ, Fac Hlth & Life Sci, Dept Biol & Med Sci, Oxford, England.

[Hill, Amanda] Univ Bristol, Bristol Med Sch, Populat Hlth Sci, Bristol, Avon, England.

[Barnett, Anna L.] Oxford Brookes Univ, Fac Hlth & Life Sci, Ctr Psychol Res, Oxford, England.

RP Mountford, HS (通讯作者)，Oxford Brookes Univ, Fac Hlth & Life Sci, Dept Biol & Med Sci, Oxford, England.

EM hmountford@brookes.ac.uk

FU UK Medical Research Council [217065/Z/19/Z]; Oxford Brookes University;

Leverhulme Trust; British Academy; Wellcome [217065/Z/19/Z]; Wellcome

Trust Centre for Human Genetics, Oxford [G1000569/1, 203141/Z/16/Z]

FX The UK Medical Research Council and Wellcome (Grant Ref: 217065/Z/19/Z)

and the University of Bristol provide core support for ALSPAC. This

publication is the work of the authors and DN will serve as guarantor

for the contents of this paper. A comprehensive list of grants funding

is available on the ALSPAC website

(http://www.bristol.ac.uk/alspac/external/documents/grant-acknowledgemen

ts.pdf).GWAS data was generated by Sample Logistics and Genotyping

Facilities at Wellcome Sanger Institute and LabCorp (Laboratory

Corporation of America) using support from 23 and Me. The work of the

Newbury Lab is currently funded by Oxford Brookes University, the

Leverhulme Trust and the British Academy. Access to the ALSPAC dataset

was, in part, completed while DN was at the Wellcome Trust Centre for

Human Genetics, Oxford as an MRC Career Development Fellow (G1000569/1),

and through the Chromosome Dynamics Core facility (Grant ref:

203141/Z/16/Z).

CR Anderson CA, 2010, NAT PROTOC, V5, P1564, DOI 10.1038/nprot.2010.116

Ansar M, 2019, AM J HUM GENET, V105, P907, DOI 10.1016/j.ajhg.2019.09.013

APA, 2013, DIAGNOSTIC STAT MANU

Barnett AL, 2021, CURR DEV DISORD REP, V8, P6, DOI 10.1007/s40474-020-00216-8

Becker N, 2017, DEV NEUROPSYCHOL, V42, P423, DOI 10.1080/87565641.2017.1374960

Bishop DVM, 2010, PLOS ONE, V5, DOI 10.1371/journal.pone.0015112

Blank R, 2019, DEV MED CHILD NEUROL, V61, P242, DOI 10.1111/dmcn.14132

Boyd A, 2013, INT J EPIDEMIOL, V42, P111, DOI 10.1093/ije/dys064

Brown-Lum M, 2020, JAMA NETW OPEN, V3, DOI 10.1001/jamanetworkopen.2020.1184

Cairney J, 2005, INT J OBESITY, V29, P369, DOI 10.1038/sj.ijo.0802893

CANTELL MH, 1994, ADAPT PHYS ACT Q, V11, P115, DOI 10.1123/apaq.11.2.115

Cerqua C, 2018, BBA-BIOENERGETICS, V1859, P244, DOI 10.1016/j.bbabio.2018.01.004

Chen EH, 2003, CELL, V114, P751, DOI 10.1016/S0092-8674(03)00720-7

Cleaton MAM, 2019, QUAL LIFE RES, V28, P925, DOI 10.1007/s11136-018-2075-1

Coe BP, 2014, NAT GENET, V46, P1063, DOI 10.1038/ng.3092

Cunningham AC, 2021, PSYCHOL MED, V51, P290, DOI 10.1017/S0033291719003210

D'Souza Ryan S, 2016, Small GTPases, V7, P257

Daulat AM, 2019, PROTEOMICS, V19, DOI 10.1002/pmic.201800487

Delaneau O, 2013, NAT METHODS, V10, P5, DOI 10.1038/nmeth.2307

Demontis D, 2019, NAT GENET, V51, P63, DOI 10.1038/s41588-018-0269-7

Fedorenko E, 2016, EUR J HUM GENET, V24, P302, DOI 10.1038/ejhg.2015.149

Fliers EA, 2012, WORLD J BIOL PSYCHIA, V13, P211, DOI 10.3109/15622975.2011.560279

Fraser A, 2013, INT J EPIDEMIOL, V42, P97, DOI 10.1093/ije/dys066

Gaines R, 2008, PAED CHILD HEALT-CAN, V13, P763, DOI 10.1093/pch/13.9.763

Harrowell I, 2018, RES DEV DISABIL, V72, P13, DOI 10.1016/j.ridd.2017.10.014

Hartmann C, 2020, BMC MOL CELL BIOL, V21, DOI 10.1186/s12860-020-00274-1

Henderson S., 2007, MOVEMENT ASSESSMENT

HENDERSON SE, 1992, MOVEMENT ASSESSMENT

Kalnak N, 2018, CLIN GENET, V94, P313, DOI 10.1111/cge.13389

Kirby A, 2011, RES DEV DISABIL, V32, P1351, DOI 10.1016/j.ridd.2011.01.041

Kirby A, 2008, J RES SPEC EDUC NEED, V8, P120, DOI 10.1111/j.1471-3802.2008.00111.x

Lai CSL, 2001, NATURE, V413, P519, DOI 10.1038/35097076

Lam M, 2019, AM J HUM GENET, V105, P334, DOI 10.1016/j.ajhg.2019.06.012

Larsen RF, 2013, DEV MED CHILD NEUROL, V55, P1016, DOI 10.1111/dmcn.12223

Licari MK, 2015, EXP BRAIN RES, V233, P1703, DOI 10.1007/s00221-015-4243-7

Lichtenstein P, 2010, AM J PSYCHIAT, V167, P1357, DOI 10.1176/appi.ajp.2010.10020223

Lingam R, 2010, PEDIATRICS, V126, pE1109, DOI 10.1542/peds.2009-2789

Lingam R, 2009, PEDIATRICS, V123, pE693, DOI 10.1542/peds.2008-1770

Lionel AC, 2011, SCI TRANSL MED, V3, DOI 10.1126/scitranslmed.3002464

LOSSE A, 1991, DEV MED CHILD NEUROL, V33, P55, DOI 10.1111/j.1469-8749.1991.tb14785.x

Martin NC, 2006, HUM MOVEMENT SCI, V25, P110, DOI 10.1016/j.humov.2005.10.006

Moruzzi S, 2010, HUM MOVEMENT SCI, V29, P326, DOI 10.1016/j.humov.2010.01.005

Mosca SJ, 2016, J MED GENET, V53, P812, DOI 10.1136/jmedgenet-2016-103818

Mountford HS, 2020, AM J MED GENET C, V184, P256, DOI 10.1002/ajmg.c.31791

Mountford HS, 2018, J LANG EVOL, V3, P49, DOI 10.1093/jole/lzx019

Newbury DF, 2013, EUR J HUM GENET, V21, P361, DOI 10.1038/ejhg.2012.166

Onel S, 2004, DEVELOPMENT, V131, P2587, DOI 10.1242/dev.01147

Patterson N, 2006, PLOS GENET, V2, P2074, DOI 10.1371/journal.pgen.0020190

Prasad A, 2012, G3-GENES GENOM GENET, V2, P1665, DOI 10.1534/g3.112.004689

Price AL, 2006, NAT GENET, V38, P904, DOI 10.1038/ng1847

Ripke S, 2014, NATURE, V511, P421, DOI 10.1038/nature13595

Sanders SJ, 2015, NEURON, V87, P1215, DOI 10.1016/j.neuron.2015.09.016

Simpson NH, 2015, EUR J HUM GENET, V23, P1370, DOI 10.1038/ejhg.2014.296

Artigas MS, 2020, MOL PSYCHIATR, V25, P2493, DOI 10.1038/s41380-018-0339-3

St Pourcain B, 2014, NAT COMMUN, V5, DOI 10.1038/ncomms5831

Stephenson EA, 2008, CHILD CARE HLTH DEV, V34, P335, DOI 10.1111/j.1365-2214.2007.00805.x

Summers J, 2008, HUM MOVEMENT SCI, V27, P215, DOI 10.1016/j.humov.2008.02.002

Tsiotra GD, 2006, J ADOLESCENT HEALTH, V39, P125, DOI 10.1016/j.jadohealth.2005.07.011

Turner SD, 2014, QQMAN R PACKAGE VISU, DOI 10.1101/005165

van Huijsduijnen RH, 1998, GENE, V206, P273, DOI 10.1016/S0378-1119(97)00597-0

Van Waelvelde H, 2004, HUM MOVEMENT SCI, V23, P49, DOI 10.1016/j.humov.2004.04.004

Wickham H, 2009, USE R, P1, DOI 10.1007/978-0-387-98141-3_1

Wright HC, 1996, DEV MED CHILD NEUROL, V38, P1099

NR 63

TC 0

Z9 0

U1 0

U2 2

PU FRONTIERS MEDIA SA

PI LAUSANNE

PA AVENUE DU TRIBUNAL FEDERAL 34, LAUSANNE, CH-1015, SWITZERLAND

SN 1662-5161

J9 FRONT HUM NEUROSCI

JI Front. Hum. Neurosci.

PD JUN 9

PY 2021

VL 15

AR 669902

DI 10.3389/fnhum.2021.669902

PG 13

WC Neurosciences; Psychology

WE Science Citation Index Expanded (SCI-EXPANDED)

SC Neurosciences & Neurology; Psychology

GA SW4TZ

UT WOS:000664510800001

PM 34177493

OA Green Published, gold

DA 2022-08-17

ER

PT J

AU Ke, L

Duan, W

Xue, Y

Wang, Y

AF Ke, Li

Duan, Wen

Xue, Ye

Wang, Yun

TI Developmental Coordination Disorder in Chinese Children Is Correlated

With Cognitive Deficits

SO FRONTIERS IN PSYCHIATRY

LA English

DT Article

DE developmental coordination disorder; movement development; cognitive

ability; cognitive deficits; execution function

ID WORKING-MEMORY; VISUOSPATIAL ATTENTION; EXECUTIVE FUNCTIONS; CLUMSY

CHILDREN; MOVEMENT; PERFORMANCE; ACTIVATION; PATTERNS; SKILLS

AB Cognitive deficits have been commonly observed in children with developmental coordination disorder (DCD), including memory, attention, and executive function difficulties. The present study evaluates the specific cognitive deficits in Chinese children with DCD, through a number of tests. A total of 401 children aged 7 to 10 years old from primary schools in Guangdong Province, China, participated in this study. Using the guidelines of the Movement Assessment Battery for Children ("Movement ABC-2"), a measurement tool of motor function ability, the children were divided into a DCD group, a group identified as being at risk of DCD, and a normal control group. The results of our analysis revealed that children's overall motor abilities could predict their overall cognitive ability, reaction time, memory, and attention. The performance of the DCD children was worse than that of the other two groups in terms of reaction time. The DCD group also returned lower scores for executive function than the normal control group did. A regression analysis showed that the cognitive deficits in children with DCD center mainly on poor executive function rather than attention and memory issues. These findings provide preliminary results regarding the cognitive deficits in Chinese children with DCD and have potential applications for the diagnosis and treatment of the disorder.

C1 [Ke, Li; Xue, Ye; Wang, Yun] Beijing Normal Univ, State Key Lab Cognit Neurosci & Learning, Beijing, Peoples R China.

[Duan, Wen] Collaborat Innovat Ctr Assessment Basic Educ Qual, Beijing, Peoples R China.

RP Wang, Y (通讯作者)，Beijing Normal Univ, State Key Lab Cognit Neurosci & Learning, Beijing, Peoples R China.

EM wangyun@bnu.edu.cn

OI Wang, Yun/0000-0002-2648-4467; Ke, Li/0000-0001-9279-4503

FU Fundamental Research Funds for the Central Universities [ZYGX2015J167]

FX This study was supported by a grant from the Fundamental Research Funds

for the Central Universities (grant number: ZYGX2015J167).

CR Alloway TP, 2007, J EXP CHILD PSYCHOL, V96, P20, DOI 10.1016/j.jecp.2006.07.002

Alloway TP, 2007, APPL COGNITIVE PSYCH, V21, P473, DOI 10.1002/acp.1284

Alloway TP, 2008, J LEARN DISABIL-US, V41, P251, DOI 10.1177/0022219408315815

American Psychiatric Association, 1987, DIAGNOSTIC STAT MANU, V3rd

APA, 2013, BMC MED, V17, P133

Bernardi M, 2018, DEV MED CHILD NEUROL, V60, P306, DOI 10.1111/dmcn.13640

DARE MT, 1970, DEV MED CHILD NEUROL, V12, P178

Davis CL, 2011, HEALTH PSYCHOL, V30, P91, DOI 10.1037/a0021766

Debrabant J, 2013, RES DEV DISABIL, V34, P1478, DOI 10.1016/j.ridd.2013.02.008

Dewey D, 2002, HUM MOVEMENT SCI, V21, P905, DOI 10.1016/S0167-9457(02)00163-X

Dewey Deborah, 2001, Physical and Occupational Therapy in Pediatrics, V20, P5, DOI 10.1300/J006v20n02_02

Duff SC, 1999, SCAND J PSYCHOL, V40, P251, DOI 10.1111/1467-9450.404124

Fong SSM, 2016, MEDICINE, V95, DOI 10.1097/MD.0000000000004935

GUBBAY SS, 1965, BRAIN, V88, P295, DOI 10.1093/brain/88.2.295

Henderson SE, 2007, EXAMINERS MANUAL, DOI [10.1037/t55281-000, DOI 10.1037/T55281-000]

Ke L., 2019, THESIS

Langaas T, 1998, VISION RES, V38, P1817, DOI 10.1016/S0042-6989(97)00399-4

Licari MK, 2015, EXP BRAIN RES, V233, P1703, DOI 10.1007/s00221-015-4243-7

Miyake A, 2000, COGNITIVE PSYCHOL, V41, P49, DOI 10.1006/cogp.1999.0734

Pangelinan MM, 2013, J NEUROPHYSIOL, V109, P3041, DOI 10.1152/jn.00532.2012

Piek JP, 2007, DEV MED CHILD NEUROL, V49, P678, DOI 10.1111/j.1469-8749.2007.00678.x

QUINN JG, 1994, Q J EXP PSYCHOL-A, V47, P465, DOI 10.1080/14640749408401120

QUINN JG, 1986, Q J EXP PSYCHOL-A, V38, P689, DOI 10.1080/14640748608401621

Raynor A J, 1998, Motor Control, V2, P114

Sigmundsson H., 2003, Neural Plasticity, V10, P27, DOI 10.1155/NP.2003.27

SMYTH MM, 1988, Q J EXP PSYCHOL-A, V40, P497, DOI 10.1080/02724988843000041

SMYTH TR, 1986, AUST J PSYCHOL, V38, P13, DOI 10.1080/00049538608256413

SMYTH TR, 1991, CHILD CARE HLTH DEV, V17, P283, DOI 10.1111/j.1365-2214.1991.tb00698.x

Vaessen W, 1990, DEVELOPMENTAL BIOPSY, P223

VANDELLEN T, 1988, J CHILD PSYCHOL PSYC, V29, P489

WALL AE, 1990, ADV PSYCHOL, V74, P283

Wilmut K, 2007, DISABIL REHABIL, V29, P47, DOI 10.1080/09638280600947765

Wilson PH, 2017, DEV MED CHILD NEUROL, V59, P1117, DOI 10.1111/dmcn.13530

Wilson PH, 2013, DEV MED CHILD NEUROL, V55, P217, DOI 10.1111/j.1469-8749.2012.04436.x

Wilson PH, 1997, DEV MED CHILD NEUROL, V39, P736

Wilson PH, 1999, HUM MOVEMENT SCI, V18, P421, DOI 10.1016/S0167-9457(99)00017-2

NR 36

TC 2

Z9 3

U1 2

U2 8

PU FRONTIERS MEDIA SA

PI LAUSANNE

PA AVENUE DU TRIBUNAL FEDERAL 34, LAUSANNE, CH-1015, SWITZERLAND

SN 1664-0640

J9 FRONT PSYCHIATRY

JI Front. Psychiatry

PD JUN 13

PY 2019

VL 10

AR 404

DI 10.3389/fpsyt.2019.00404

PG 7

WC Psychiatry

WE Science Citation Index Expanded (SCI-EXPANDED); Social Science Citation Index (SSCI)

SC Psychiatry

GA ID7UH

UT WOS:000471887800001

PM 31249536

OA Green Published, gold

DA 2022-08-17

ER

PT J

AU Licari, MK

Billington, J

Reid, SL

Wann, JP

Elliott, CM

Winsor, AM

Robins, E

Thornton, AL

Jones, R

Bynevelt, M

AF Licari, Melissa K.

Billington, Jac

Reid, Siobhan L.

Wann, John P.

Elliott, Catherine M.

Winsor, Anne M.

Robins, Erin

Thornton, Ashleigh L.

Jones, Randall

Bynevelt, Michael

TI Cortical functioning in children with developmental coordination

disorder: a motor overflow study

SO EXPERIMENTAL BRAIN RESEARCH

LA English

DT Article

DE Developmental coordination disorder; Functional magnetic resonance

imaging; Brain function; Motor overflow

ID ATTENTION-DEFICIT/HYPERACTIVITY DISORDER; DEFICIT-HYPERACTIVITY

DISORDER; NEUROLOGICAL SOFT SIGNS; NEUROMOTOR DEVELOPMENT; OBJECT

MANIPULATION; MOVEMENTS; SCHIZOPHRENIA; DIFFICULTIES; DYSFUNCTION;

ACTIVATION

AB This study examined brain activation in children with developmental coordination disorder (DCD) to reveal areas that may contribute to poor movement execution and/or abundant motor overflow. Using functional magnetic resonance imaging, 13 boys with DCD (mean age = 9.6 years +/- 0.8) and 13 typically developing controls (mean age = 9.3 years +/- 0.6) were scanned performing two tasks (finger sequencing and hand clenching) with their dominant hand, while a four-finger motion sensor recorded contralateral motor overflow on their non-dominant hand. Despite displaying increased motor overflow on both functional tasks during scanning, there were no obvious activation deficits in the DCD group to explain the abundant motor overflow seen. However, children with DCD were found to display decreased activation in the left superior frontal gyrus on the finger-sequencing task, an area which plays an integral role in executive and spatially oriented processing. Decreased activation was also seen in the left inferior frontal gyrus, an area typically active during the observation and imitation of hand movements. Finally, increased activation in the right postcentral gyrus was seen in children with DCD, which may reflect increased reliance on somatosensory information during the execution of complex fine motor tasks.

C1 [Licari, Melissa K.; Reid, Siobhan L.; Thornton, Ashleigh L.] Univ Western Australia, Sch Sport Sci Exercise & Hlth, Crawley, WA 6009, Australia.

[Billington, Jac] Univ Leeds, Inst Psychol Sci, Leeds, W Yorkshire, England.

[Wann, John P.] Univ London, Dept Psychol, Egham, Surrey, England.

[Elliott, Catherine M.] Univ Western Australia, Sch Paediat & Child Hlth, Perth, WA 6009, Australia.

[Winsor, Anne M.; Jones, Randall; Bynevelt, Michael] Sir Charles Gairdner Hosp, Neurol Intervent & Imaging Serv Western Australia, Nedlands, WA 6009, Australia.

[Robins, Erin; Bynevelt, Michael] Princess Margaret Hosp Children, Dept Diagnost Imaging, Subiaco, WA, Australia.

RP Licari, MK (通讯作者)，Univ Western Australia, Sch Sport Sci Exercise & Hlth, 35 Stirling Highway, Crawley, WA 6009, Australia.

EM melissa.licari@uwa.edu.au

RI ; Elliott, Catherine/B-6718-2014; Thornton, Ashleigh/H-5960-2014

OI Billington, Jac/0000-0003-0995-8875; Reid, Siobhan/0000-0002-2589-3576;

Elliott, Catherine/0000-0002-5324-8216; Licari,

Melissa/0000-0003-3705-5323; Thornton, Ashleigh/0000-0003-3712-3330

FU University of Western Australia

FX The authors would like to thank the radiology staff from Sir Charles

Gairdner Hospital and Princess Margaret hospital involved in this

project, and the children and parents for their time and participation.

We would also like to thank Mag Design and Engineering who created the

motion sensor glove. This project was funded by a Research Development

Award from the University of Western Australia.

CR American Psychiatric Association, 2013, DIAGN STAT MAN MENT, V5th edition, DOI [10.1176/appi.books.9780890425596, DOI 10.1176/APPI.BOOKS.9780890425596]

American Psychological Association, 2000, DIAGN STAT MAN MENT, DOI DOI 10.1176/APPI.BOOKS.9780890423349

Andreasen NC, 1996, P NATL ACAD SCI USA, V93, P9985, DOI 10.1073/pnas.93.18.9985

Binkofski F, 1999, EUR J NEUROSCI, V11, P3276, DOI 10.1046/j.1460-9568.1999.00753.x

Binkofski F, 1999, EXP BRAIN RES, V128, P210, DOI 10.1007/s002210050838

Blank R, 2012, DEV MED CHILD NEUROL, V54, P54, DOI 10.1111/j.1469-8749.2011.04171.x

Cairney J, 2010, CURR OPIN PSYCHIATR, V23, P324, DOI 10.1097/YCO.0b013e32833aa0aa

Dewey D, 2007, J INT NEUROPSYCH SOC, V13, P246, DOI 10.1017/S1355617707070270

du Boisgueheneuc F, 2006, BRAIN, V129, P3315, DOI 10.1093/brain/awl244

Elbasan B, 2012, ITAL J PEDIATR, V38, DOI 10.1186/1824-7288-38-14

Henderson S., 2007, MOVEMENT ASSESSMENT

Kashiwagi M, 2009, NEUROREPORT, V20, P1319, DOI 10.1097/WNR.0b013e32832f4d87

Kuhtz-Buschbeck JP, 2000, DEV MED CHILD NEUROL, V42, P728, DOI 10.1017/S0012162200001353

Largo RH, 2003, SWISS MED WKLY, V133, P193

Largo RH, 2001, DEV MED CHILD NEUROL, V43, P444, DOI 10.1017/S0012162201000822

Licari M, 2006, HUM MOVEMENT SCI, V25, P90, DOI 10.1016/j.humov.2005.10.012

Licari M, 2008, HUM MOVEMENT SCI, V27, P310, DOI 10.1016/j.humov.2008.02.013

MacNeil LK, 2011, NEUROLOGY, V76, P622, DOI 10.1212/WNL.0b013e31820c3052

Marien P, 2010, CEREBELLUM, V9, P405, DOI 10.1007/s12311-010-0177-6

Mostofsky SH, 2006, BIOL PSYCHIAT, V59, P48, DOI 10.1016/j.biopsych.2005.06.011

Mouchet-Mages S, 2011, ACTA PSYCHIAT SCAND, V123, P451, DOI 10.1111/j.1600-0447.2010.01667.x

OLDFIELD RC, 1971, NEUROPSYCHOLOGIA, V9, P97, DOI 10.1016/0028-3932(71)90067-4

Ozbic M, 2010, KINESIOLOGY, V42, P44

Piek JP, 2007, DEV MED CHILD NEUROL, V49, P678, DOI 10.1111/j.1469-8749.2007.00678.x

Querne L, 2008, BRAIN RES, V1244, P89, DOI 10.1016/j.brainres.2008.07.066

Venkatasubramanian G, 2008, PSYCHIAT RES-NEUROIM, V164, P215, DOI 10.1016/j.pscychresns.2007.12.021

Werner JM, 2012, J BEHAV BRAIN SCI, V2, P258, DOI [10.4236/jbbs.2012.22029, DOI 10.4236/JBBS.2012.22029]

WHO, 2012, ICD 10 CLASS MENT BE

Wolraich ML, 2013, VANDERBILT ADHD PARE

Zwicker JG, 2011, INT J DEV NEUROSCI, V29, P145, DOI 10.1016/j.ijdevneu.2010.12.002

Zwicker JG, 2010, PEDIATRICS, V126, pE678, DOI 10.1542/peds.2010-0059

Zwicker JG, 2009, J CHILD NEUROL, V24, P1273, DOI 10.1177/0883073809333537

NR 32

TC 42

Z9 42

U1 1

U2 25

PU SPRINGER

PI NEW YORK

PA 233 SPRING ST, NEW YORK, NY 10013 USA

SN 0014-4819

EI 1432-1106

J9 EXP BRAIN RES

JI Exp. Brain Res.

PD JUN

PY 2015

VL 233

IS 6

BP 1703

EP 1710

DI 10.1007/s00221-015-4243-7

PG 8

WC Neurosciences

WE Science Citation Index Expanded (SCI-EXPANDED)

SC Neurosciences & Neurology

GA CI4OR

UT WOS:000354731200003

PM 25757959

OA Green Accepted

DA 2022-08-17

ER

PT J

AU Salaj, S

Masnjak, M

AF Salaj, Sanja

Masnjak, Mia

TI Correlation of Motor Competence and Social-Emotional Wellbeing in

Preschool Children

SO FRONTIERS IN PSYCHOLOGY

LA English

DT Article

DE skill; development; association; proficiency; movement

ID PHYSICAL-ACTIVITY; SKILLS; PERFORMANCE; COORDINATION; CHILDHOOD; FITNESS

AB IntroductionThe relations of motor skills to different developmental domains, i.e., cognitive, emotional, and social domain, are well-documented in research on children with poor motor competence and children with disabilities. Less conclusive evidence on interaction of motor and social or emotional development can be seen in research on typically developing children. The purpose of this study was to determine a correlation between motor skills and social-emotional functioning in typically developing preschool children and to identify differences in social-emotional functioning in children with different levels of motor competence. MethodsA total of 125 preschool children (67 boys, 58 girls, average age 5.1 years) participated in this study. To assess children's motor skills, we used the Test of Gross Motor Development-Second Edition that measures locomotor and object-control skills. To screen child's social and emotional functioning, we used the Ages and Stages Questionnaire-Social Emotional: Second Edition. Spearman's correlation analysis was used to determine association between motor skills and social-emotional functioning. Difference in social-emotional functioning between groups of preschool children with High and Low motor competences was calculated using Mann-Whitney U-test. ResultsThe main result of this study is weak correlation of child's motor skills to social-emotional functioning. Furthermore, preschool children with High and Low motor competences do not differ in risk for social and emotional difficulties. ConclusionFurther research on typically developing children is needed to have more conclusive evidence on interaction of motor and social or emotional development.

C1 [Salaj, Sanja; Masnjak, Mia] Univ Zagreb, Fac Kinesiol, Motor Dev Lab, Zagreb, Croatia.

RP Salaj, S (通讯作者)，Univ Zagreb, Fac Kinesiol, Motor Dev Lab, Zagreb, Croatia.

EM sanja.salaj@kif.hr

RI Salaj, Sanja/V-5962-2018

OI Salaj, Sanja/0000-0002-8581-1958

FU Croatian Science Foundation [UIP-201409-5428]

FX This research was conducted within the national scientific research

project "Motor skills in preschool children" (UIP-201409-5428) funded by

the Croatian Science Foundation.

CR Adolph Karen E, 2020, Nestle Nutr Inst Workshop Ser, V95, P136, DOI 10.1159/000511511

Asonitou K, 2010, PROCD SOC BEHV, V5, P1702, DOI 10.1016/j.sbspro.2010.07.350

Bardid F, 2021, SCAND J MED SCI SPOR, V31, P5, DOI 10.1111/sms.13946

Barnett LM, 2009, J ADOLESCENT HEALTH, V44, P252, DOI 10.1016/j.jadohealth.2008.07.004

Bruininks R., 2005, BRUININKS OSERETSKY, DOI [10.1037/t14991-000, DOI 10.1037/T14991-000]

Cattuzzo MT, 2016, J SCI MED SPORT, V19, P123, DOI 10.1016/j.jsams.2014.12.004

Cheung WC, 2022, J DEV PHYS DISABIL, V34, P211, DOI 10.1007/s10882-021-09796-8

CICCHETTI D, 1995, DEV PSYCHOPATHOL, V7, P1, DOI 10.1017/S0954579400006301

Dadgar Hooshang, 2017, Iran J Psychiatry, V12, P236

Dancey Christine P, 2007, STAT MATHS PSYCHOL

Dapp LC, 2021, PSYCHOL SPORT EXERC, V54, DOI 10.1016/j.psychsport.2021.101916

Eather Narelle, 2018, Prev Med Rep, V11, P191, DOI 10.1016/j.pmedr.2018.06.005

Ecevit R., 2021, EUR J EDUC, V8, P3928, DOI [10.46827/ejes.v8i10.3928, DOI 10.46827/EJES.V8I10.3928]

Emck C, 2009, DEV MED CHILD NEUROL, V51, P501, DOI 10.1111/j.1469-8749.2009.03337.x

Farooq A, 2020, OBES REV, V21, DOI 10.1111/obr.12953

Freeman S, 2014, P NATL ACAD SCI USA, V111, P8410, DOI 10.1073/pnas.1319030111

Griffiths LJ, 2010, INT J BEHAV NUTR PHY, V7, DOI 10.1186/1479-5868-7-30

Henderson S., 2007, MOVEMENT ASSESSMENT

Hsu Hung-Chih, 2004, Chang Gung Med J, V27, P750

Izard CE, 2009, ANNU REV PSYCHOL, V60, P1, DOI 10.1146/annurev.psych.60.110707.163539

Kim H, 2016, RES DEV DISABIL, V53-54, P43, DOI 10.1016/j.ridd.2016.01.016

Kuzik N, 2020, PLOS ONE, V15, DOI 10.1371/journal.pone.0237945

LaFreniere Peter J., 2000, EMOTIONAL DEV BIOSOC

Lee K, 2020, INT J ENV RES PUB HE, V17, DOI 10.3390/ijerph17207362

Lima RA, 2017, J PHYS ACT HEALTH, V14, P440, DOI 10.1123/jpah.2016-0473

Loprinzi Paul D, 2015, Prev Med Rep, V2, P833, DOI 10.1016/j.pmedr.2015.09.015

MacDonald M, 2017, ADAPT PHYS ACT Q, V34, P179, DOI [10.1123/apaq.2015-0091, 10.1123/APAQ.2015-0091]

MacDonald M, 2013, ADAPT PHYS ACT Q, V30, P271, DOI 10.1123/apaq.30.3.271

Martoccio TL, 2014, INFANT YOUNG CHILD, V27, P193, DOI 10.1097/IYC.0000000000000014

Masnjak M., 2016, ASQ WORLD 2 INVITATI

Nix RL, 2013, EARLY EDUC DEV, V24, P1000, DOI 10.1080/10409289.2013.825565

Ohara R, 2020, EUR J INVEST HEALTH, V10, P276, DOI 10.3390/ejihpe10010022

Piek JP, 2015, HUM MOVEMENT SCI, V43, P155, DOI 10.1016/j.humov.2015.08.004

Piek JP, 2010, HUM MOVEMENT SCI, V29, P777, DOI 10.1016/j.humov.2010.03.006

Piek JP, 2008, INT J DISABIL DEV ED, V55, P143, DOI 10.1080/10349120802033592

Piek JP, 2006, HUM MOVEMENT SCI, V25, P65, DOI 10.1016/j.humov.2005.10.011

Pienaar AE, 2021, INT J ENV RES PUB HE, V18, DOI 10.3390/ijerph18041648

Rigoli D, 2012, PEDIATRICS, V129, pE892, DOI 10.1542/peds.2011-1237

Rodrigues LP, 2016, J SCI MED SPORT, V19, P87, DOI 10.1016/j.jsams.2015.01.002

Shonkoff J. P., 2000, NEURONS NEIGHBORHOOD, DOI [10.17226/9824, DOI 10.17226/9824]

Skinner RA, 2001, HUM MOVEMENT SCI, V20, P73, DOI 10.1016/S0167-9457(01)00029-X

Squires J., 2015, ASQSE 2 TM USERS GUI, V2nd

Starc B., 2004, CHARACTERISTICS PHYS

Stodden DF, 2008, QUEST, V60, P290, DOI 10.1080/00336297.2008.10483582

Ulrich D.A, 2000, TEST GROSS MOTOR DEV, V2nd ed

Vukelja M, 2022, MONTENEGRIN J SPORT, V11, P37, DOI 10.26773/mjssm.220304

Williams HG, 2008, OBESITY, V16, P1421, DOI 10.1038/oby.2008.214

NR 47

TC 0

Z9 0

U1 1

U2 1

PU FRONTIERS MEDIA SA

PI LAUSANNE

PA AVENUE DU TRIBUNAL FEDERAL 34, LAUSANNE, CH-1015, SWITZERLAND

SN 1664-1078

J9 FRONT PSYCHOL

JI Front. Psychol.

PD APR 6

PY 2022

VL 13

AR 846520

DI 10.3389/fpsyg.2022.846520

PG 7

WC Psychology, Multidisciplinary

WE Social Science Citation Index (SSCI)

SC Psychology

GA 1Z5RM

UT WOS:000808881500001

PM 35465487

OA gold, Green Published

DA 2022-08-17

ER

PT J

AU Omer, S

Leonard, HC

AF Omer, Serif

Leonard, Hayley C.

TI Internalising symptoms in Developmental Coordination Disorder: The

indirect effect of everyday executive function

SO RESEARCH IN DEVELOPMENTAL DISABILITIES

LA English

DT Article

DE Developmental Coordination Disorder; Dyspraxia; Motor development;

Executive function; Internalising symptom; Mental health

ID ENVIRONMENTAL-STRESS HYPOTHESIS; PERFORMANCE-BASED MEASURES; EMOTION

REGULATION; DEPRESSIVE SYMPTOMS; COGNITIVE CONTROL; FUNCTION DEFICITS;

DAILY-LIFE; CHILDREN; BEHAVIOR; RUMINATION

AB Background: Individuals with Developmental Coordination Disorder (DCD) report elevated executive function (EF) difficulties and internalising symptoms. Previous research suggests EF is important for wellbeing, yet no research has examined its role in internalising symptoms in DCD.

Aims: To explore an indirect relationship between DCD and internalising symptoms, through everyday EF difficulties.

Method and procedures: Thirty-two children with a DCD diagnosis and 51 typically-developing children (ages 8-15) participated. A cross-sectional survey was conducted to collect parent-reported EF and self-reported internalising symptoms.

Outcomes and results: Internalising symptoms and everyday EF difficulties were significantly higher in the DCD group. A bias-corrected, bootstrapped mediation analysis identified an indirect effect of everyday EF difficulties on the relationship between DCD diagnosis and internalising symptoms.

Conclusions and implications: This supports previous research indicating that individuals with DCD experience greater levels of internalising symptoms and EF difficulties than peers. It is the first to suggest an indirect effect of everyday EF difficulties in the pathway between DCD and internalising symptoms. This highlights hypotheses for future research into the role of EFs in understanding mental health in DCD. It suggests benefits from increased awareness, routine screening, and intervention for mental health and EF in people with poor motor skills.

C1 [Omer, Serif; Leonard, Hayley C.] Univ Surrey, Sch Psychol, Guildford GU2 7HX, Surrey, England.

RP Leonard, HC (通讯作者)，Univ Surrey, Sch Psychol, Guildford GU2 7HX, Surrey, England.

EM h.leonard@surrey.ac.uk

CR Agoston AM, 2016, J EARLY ADOLESCENCE, V36, P1070, DOI 10.1177/0272431615593176

Amani M, 2019, GAMES HEALTH J, V8, P213, DOI 10.1089/g4h.2018.0052

American Psychiatric Association, 2013, DIAGN STAT MAN

Barkley RA, 2011, DEV NEUROPSYCHOL, V36, P137, DOI 10.1080/87565641.2010.549877

Bernardi M, 2018, DEV MED CHILD NEUROL, V60, P306, DOI 10.1111/dmcn.13640

Bolin JH, 2014, J EDUC MEAS, V51, P335, DOI 10.1111/jedm.12050

Brosschot JF, 2006, J PSYCHOSOM RES, V60, P113, DOI 10.1016/j.jpsychores.2005.06.074

Burgess PW, 2006, J INT NEUROPSYCH SOC, V12, P194, DOI 10.1017/S1355617706060310

Cairney J, 2013, DEV REV, V33, P224, DOI 10.1016/j.dr.2013.07.002

Campbell WN, 2012, PSYCHOL SCHOOLS, V49, P328, DOI 10.1002/pits.21600

Clark CAC, 2013, CHILD DEV, V84, P662, DOI 10.1111/j.1467-8624.2012.01854.x

COHEN J, 1992, PSYCHOL BULL, V112, P155, DOI 10.1037/0033-2909.112.1.155

Cousins M, 2003, HUM MOVEMENT SCI, V22, P433, DOI 10.1016/j.humov.2003.09.003

Dawson EL, 2012, J CLIN EXP NEUROPSYC, V34, P297, DOI 10.1080/13803395.2011.639297

De Raedt R, 2010, COGN AFFECT BEHAV NE, V10, P50, DOI 10.3758/CABN.10.1.50

Demeyer I, 2012, BEHAV RES THER, V50, P292, DOI 10.1016/j.brat.2012.02.012

Diamond A, 2000, CHILD DEV, V71, P44, DOI 10.1111/1467-8624.00117

Diamond A, 2013, ANNU REV PSYCHOL, V64, P135, DOI 10.1146/annurev-psych-113011-143750

Ebesutani C, 2012, PSYCHOL ASSESSMENT, V24, P833, DOI 10.1037/a0027283

Ernst M, 2006, PSYCHOL MED, V36, P299, DOI 10.1017/S0033291705005891

Evans LD, 2016, J CLIN CHILD ADOLESC, V45, P6, DOI 10.1080/15374416.2014.982282

Fritz MS, 2007, PSYCHOL SCI, V18, P233, DOI 10.1111/j.1467-9280.2007.01882.x

GARBER J, 1995, DEV PSYCHOPATHOL, V7, P93, DOI 10.1017/S0954579400006362

Gardiner E, 2018, AUTISM RES, V11, P284, DOI 10.1002/aur.1877

Gilotty L, 2002, CHILD NEUROPSYCHOL, V8, P241, DOI 10.1076/chin.8.4.241.13504

Gioia GA, 2000, CHILD NEUROPSYCHOL, V6, P235, DOI 10.1076/chin.6.3.235.3152

Han G, 2016, J CLIN CHILD ADOLESC, V45, P44, DOI 10.1080/15374416.2015.1041592

Harrowell I, 2017, DEV MED CHILD NEUROL, V59, P973, DOI 10.1111/dmcn.13469

Johnson DR, 2009, EMOTION, V9, P681, DOI 10.1037/a0017095

Joormann J, 2010, COGNITION EMOTION, V24, P913, DOI 10.1080/02699931003784939

Joormann J, 2010, COGNITION EMOTION, V24, P281, DOI 10.1080/02699930903407948

Kertz SJ, 2016, J ABNORM CHILD PSYCH, V44, P1185, DOI 10.1007/s10802-015-0101-0

Kirby A, 2011, RES DEV DISABIL, V32, P1351, DOI 10.1016/j.ridd.2011.01.041

Knouse LE, 2013, J AFFECT DISORDERS, V145, P270, DOI 10.1016/j.jad.2012.05.064

Koenigs M, 2009, BEHAV BRAIN RES, V201, P239, DOI 10.1016/j.bbr.2009.03.004

Koole S, 2009, COGNITION EMOTION, V23, P4, DOI 10.1080/02699930802619031

Koster EHW, 2011, CLIN PSYCHOL REV, V31, P138, DOI 10.1016/j.cpr.2010.08.005

Kovacs M, 1998, J CHILD PSYCHOL PSYC, V39, P47, DOI 10.1017/S0021963097001765

Koziol LF, 2012, CEREBELLUM, V11, P505, DOI 10.1007/s12311-011-0321-y

Lengua LJ, 2006, DEV PSYCHOL, V42, P819, DOI 10.1037/0012-1649.42.5.819

Leonard H. C., 2015, CURR DEV DISORD REP, V2, P141, DOI [10.1007/s40474-015-0044-8, DOI 10.1007/S40474-015-0044-8]

Letkiewicz AM, 2014, COGNITIVE THER RES, V38, P612, DOI 10.1007/s10608-014-9629-5

Levens SM, 2009, J ABNORM PSYCHOL, V118, P757, DOI 10.1037/a0017206

Li YC, 2018, PSYCHOL SPORT EXERC, V37, P244, DOI 10.1016/j.psychsport.2017.11.001

Mancini VO, 2016, FRONT PSYCHOL, V7, DOI [10.3389/fpsyg.2016.00230, 10.3389/fpsyg.2016.00239]

Martel MM, 2007, DEV PSYCHOPATHOL, V19, P541, DOI 10.1017/S0954579407070265

Mezulis Amy H, 2011, Depress Res Treat, V2011, P487873, DOI 10.1155/2011/487873

Nelson TD, 2018, J ABNORM CHILD PSYCH, V46, P1509, DOI 10.1007/s10802-017-0395-1

Nigg JT, 1999, J ABNORM CHILD PSYCH, V27, P51, DOI 10.1023/A:1022614407893

Omer S, 2019, J CHILD PSYCHOL PSYC, V60, P606, DOI 10.1111/jcpp.13001

Piaget J., ORIGIN INTELLIGENCE

Piek JP, 2010, HUM MOVEMENT SCI, V29, P777, DOI 10.1016/j.humov.2010.03.006

Price JL, 2010, NEUROPSYCHOPHARMACOL, V35, P192, DOI 10.1038/npp.2009.104

Pugliese CE, 2015, J AUTISM DEV DISORD, V45, P1579, DOI 10.1007/s10803-014-2309-1

Riggs NR, 2006, PREV SCI, V7, P91, DOI 10.1007/s11121-005-0022-1

Riggs NR, 2003, CHILD NEUROPSYCHOL, V9, P267, DOI 10.1076/chin.9.4.267.23513

Rigoli D, 2012, PEDIATRICS, V129, pE892, DOI 10.1542/peds.2011-1237

Saban MT, 2014, RES DEV DISABIL, V35, P2644, DOI 10.1016/j.ridd.2014.07.002

Schmeichel BJ, 2015, CURR DIR PSYCHOL SCI, V24, P93, DOI 10.1177/0963721414555178

Silk JS, 2003, CHILD DEV, V74, P1869, DOI 10.1046/j.1467-8624.2003.00643.x

Snyder HR, 2016, CLIN PSYCHOL SCI, V4, P1047, DOI 10.1177/2167702616633157

Snyder HR, 2015, CLIN PSYCHOL SCI, V3, P301, DOI 10.1177/2167702614534210

Snyder HR, 2013, PSYCHOL BULL, V139, P81, DOI 10.1037/a0028727

Streiner DL, 2011, CHEST, V140, P16, DOI 10.1378/chest.11-0523

Ten Eycke KD, 2016, CHILD NEUROPSYCHOL, V22, P889, DOI 10.1080/09297049.2015.1065961

Toplak ME, 2013, J CHILD PSYCHOL PSYC, V54, P131, DOI 10.1111/jcpp.12001

Vaivre-Douret L, 2014, NEUROPHYSIOL CLIN, V44, P13, DOI 10.1016/j.neucli.2013.10.133

Wagner M, 2016, FRONT PSYCHOL, V7, DOI 10.3389/fpsyg.2016.01904

Wagner S, 2015, EUR CHILD ADOLES PSY, V24, P5, DOI 10.1007/s00787-014-0559-2

Wante L, 2017, CHILD NEUROPSYCHOL, V23, P935, DOI 10.1080/09297049.2016.1212986

White BA, 2013, J PSYCHOPATHOL BEHAV, V35, P1, DOI 10.1007/s10862-012-9310-9

Whitmer AJ, 2013, PSYCHOL BULL, V139, P1036, DOI 10.1037/a0030923

Wilson BN, 2009, PHYS OCCUP THER PEDI, V29, P182, DOI 10.1080/01942630902784761

Wilson PH, 2013, DEV MED CHILD NEUROL, V55, P217, DOI 10.1111/j.1469-8749.2012.04436.x

Zwicker JG, 2013, CHILD CARE HLTH DEV, V39, P562, DOI 10.1111/j.1365-2214.2012.01379.x

NR 75

TC 2

Z9 2

U1 3

U2 8

PU PERGAMON-ELSEVIER SCIENCE LTD

PI OXFORD

PA THE BOULEVARD, LANGFORD LANE, KIDLINGTON, OXFORD OX5 1GB, ENGLAND

SN 0891-4222

EI 1873-3379

J9 RES DEV DISABIL

JI Res. Dev. Disabil.

PD FEB

PY 2021

VL 109

AR 103831

DI 10.1016/j.ridd.2020.103831

PG 11

WC Education, Special; Rehabilitation

WE Social Science Citation Index (SSCI)

SC Education & Educational Research; Rehabilitation

GA QH9MO

UT WOS:000618598000002

PM 33360963

DA 2022-08-17

ER

PT J

AU Doney, R

Lucas, BR

Watkins, RE

Tsang, TW

Sauer, K

Howat, P

Latimer, J

Fitzpatrick, JP

Oscar, J

Carter, M

Elliott, EJ

AF Doney, Robyn

Lucas, Barbara R.

Watkins, Rochelle E.

Tsang, Tracey W.

Sauer, Kay

Howat, Peter

Latimer, Jane

Fitzpatrick, James P.

Oscar, June

Carter, Maureen

Elliott, Elizabeth J.

TI Visual-motor integration, visual perception, and fine motor coordination

in a population of children with high levels of Fetal Alcohol Spectrum

Disorder

SO RESEARCH IN DEVELOPMENTAL DISABILITIES

LA English

DT Article

DE Fetal alcohol spectrum disorders; Psychomotor performance; Motor skills;

Visual motor; Indigenous population

ID HANDWRITING SKILLS; PRENATAL TOBACCO; EXPOSURE; ABNORMALITIES;

KINDERGARTEN; INDIVIDUALS; ADOLESCENCE; PERFORMANCE; CONSUMPTION;

IMPAIRMENT

AB Background: Visual-motor integration (VMI) skills are essential for successful academic performance, but to date no studies have assessed these skills in a population-based cohort of Australian Aboriginal children who, like many children in other remote, disadvantaged communities, consistently underperform academically. Furthermore, many children in remote areas of Australia have prenatal alcohol exposure (PAE) and Fetal Alcohol Spectrum Disorder (FASD), which are often associated with VMI deficits.

Methods: VMI, visual perception, and fine motor coordination were assessed using The Beery-Buktenica Developmental Test of Visual-Motor Integration, including its associated subtests of Visual Perception and Fine Motor Coordination, in a cohort of predominantly Australian Aboriginal children (7.5-9.6 years, n = 108) in remote Western Australia to explore whether PAE adversely affected test performance. Cohort results were reported, and comparisons made between children i) without PAE; ii) with PAE (no FASD); and iii) FASD. The prevalence of moderate (<= 16th percentile) and severe (<= 2nd percentile) impairment was established.

Results: Mean VMI scores were 'below average' (M = 87.8 +/- 9.6), and visual perception scores were 'average' (M = 97.6 +/- 12.5), with no differences between groups. Few children had severe VMI impairment (1.9%), but moderate impairment rates were high (47.2%). Children with FASD had significantly lower fine motor coordination scores and higher moderate impairment rates (M = 87.9 +/- 12.5; 66.7%) than children without PAE (M = 95.1 +/- 10.7; 23.3%) and PAE (no FASD) (M = 96.1 +/- 10.9; 15.4%).

Conclusions: Aboriginal children living in remote Western Australia have poor VMI skills regardless of PAE or FASD. Children with FASD additionally had fine motor coordination problems. VMI and fine motor coordination should be assessed in children with PAE, and included in FASD diagnostic assessments. (c) 2016 Elsevier Ltd. All rights reserved.

C1 [Doney, Robyn; Sauer, Kay; Howat, Peter] Curtin Univ, Sch Publ Hlth, Perth, WA 6845, Australia.

[Lucas, Barbara R.; Tsang, Tracey W.; Fitzpatrick, James P.; Elliott, Elizabeth J.] Univ Sydney, Sydney Med Sch, Discipline Paediat & Child Hlth, Sydney, NSW 2006, Australia.

[Lucas, Barbara R.; Tsang, Tracey W.; Latimer, Jane; Fitzpatrick, James P.; Elliott, Elizabeth J.] Univ Sydney, Sydney Med Sch, George Inst Global Hlth, Sydney, NSW 2006, Australia.

[Lucas, Barbara R.] Univ Sydney, Sydney Med Sch, Poche Ctr Indigenous Hlth, Sydney, NSW 2006, Australia.

[Lucas, Barbara R.] Royal N Shore Hosp, Physiotherapy Dept, Sydney, NSW, Australia.

[Watkins, Rochelle E.; Fitzpatrick, James P.] Univ Western Australia, Telethon Kids Inst, Perth, WA 6009, Australia.

[Sauer, Kay; Howat, Peter] Curtin Univ, Ctr Behav Res Canc Control, Perth, WA 6845, Australia.

[Oscar, June] Marninwarntikura Womens Resource Ctr, Fitzroy Crossing, Australia.

[Oscar, June] Univ Notre Dame, Broome, Australia.

[Carter, Maureen] Nindilingarri Cultural Hlth Serv, Fitzroy Crossing, Australia.

[Elliott, Elizabeth J.] Sydney Childrens Hosp Network Westmead, Sydney, NSW, Australia.

RP Doney, R (通讯作者)，Curtin Univ, Sch Publ Hlth, Perth, WA 6845, Australia.

EM robyndoney@gmail.com; blucas@georgeinstitute.org.au;

rochelle.watkins@telethonkids.org.au; tracey.tsang@sydney.edu.au;

k.sauer@curtin.edu.au; p.howat@curtin.edu.au;

jlatimer@georgeinstitute.org.au; james.fitzpatrick@telethonkids.org.au;

ceo@mwrc.com.au; maureen.carter@nindilingarri.org.au;

elizabeth.elliott@health.nsw.gov.au

RI Tsang, Tracey W/J-4399-2012

OI Tsang, Tracey W/0000-0002-3753-5506; Watkins,

Rochelle/0000-0002-1372-4089; Elliott, Elizabeth/0000-0002-6501-5487

FU National Health and Medical Research Council of Australia [1024474,

1021480]; Australian Government Department of Health and Ageing (DOHA);

Australian Government Department of Families, Housing, Community

Services and Indigenous Affairs (FaHCSIA); Save the Children Australia;

Foundation for Alcohol Research and Education; Australian Postgraduate

Award; Curtin University Postgraduate Scholarship; Poche Centre for

Indigenous Health Fellowship, Sydney Medical School, The University of

Sydney; Australian Research Council [0130007]

FX The Lililwan Project was supported by the National Health and Medical

Research Council of Australia (Project Grant No. 1024474); the

Australian Government Department of Health and Ageing (DOHA); the

Australian Government Department of Families, Housing, Community

Services and Indigenous Affairs (FaHCSIA); Save the Children Australia;

and the Foundation for Alcohol Research and Education. Pro bono support

was provided by M&C Saatchi; Blake Dawson Solicitors; and the Australian

Human Rights Commission. Robyn Doney is supported by an Australian

Postgraduate Award, a Curtin University Postgraduate Scholarship and

Faculty Postgraduate Award. Barbara Lucas is supported by a Poche Centre

for Indigenous Health Fellowship, Sydney Medical School, The University

of Sydney. Professor Jane Latimer is supported by an Australian Research

Council Future Fellowship (No. 0130007). Professor Elizabeth Elliott was

supported by National Health and Medical Research Council of Australia

Practitioner Fellowships (No. 1021480).

CR Alvik A, 2006, ALCOHOL CLIN EXP RES, V30, P510, DOI 10.1111/j.1530-0277.2006.00055.x

Archibald SL, 2001, DEV MED CHILD NEUROL, V43, P148, DOI 10.1017/S0012162201000299

ARONSON M, 1985, ACTA PAEDIATR SCAND, V74, P27, DOI 10.1111/j.1651-2227.1985.tb10916.x

Astley SJ, 2000, ALCOHOL ALCOHOLISM, V35, P400, DOI 10.1093/alcalc/35.4.400

ASTLEY SJ, 2009, CAN J CLIN PHARM, V16, P178

Australian Curriculum Assessment and Reporting Authority, 2015, NAPLAN ACHIEVEMENT I

Australian Health Ministers' Advisory Council, 2012, AB TORR STRAIT ISL H

Avaria MD, 2004, J PEDIATR-US, V144, P338

Beery Keith E., 2010, BEERY BUKTENICA DEV, V6th

Benton A, 1993, CLIN NEUROPSYCHOLOGY, P165

Bracken B. A., 1998, UNIVERSAL NONVERBAL

Bush K, 1998, ARCH INTERN MED, V158, P1789, DOI 10.1001/archinte.158.16.1789

Case-Smith J, 2002, AM J OCCUP THER, V56, P17, DOI 10.5014/ajot.56.1.17

Case-Smith J, 2014, AM J OCCUP THER, V68, P690, DOI 10.5014/ajot.2014.011585

Centers for Disease Control and Prevention, 2005, FET ALC SPECTR DIS G

CHASE CI, 1986, J EDUC MEAS, V23, P33, DOI 10.1111/j.1745-3984.1986.tb00232.x

Chudley AE, 2005, CAN MED ASSOC J, V172, pS1, DOI 10.1503/cmaj.1040302

Cornelius MD, 2001, J DEV BEHAV PEDIATR, V22, P217, DOI 10.1097/00004703-200108000-00002

Daly CJ, 2003, AM J OCCUP THER, V57, P459, DOI 10.5014/ajot.57.4.459

David P, 2005, BIRTH DEFECTS RES A, V73, P897, DOI 10.1002/bdra.20190

Doney R, 2014, J DEV BEHAV PEDIATR, V35, P598, DOI 10.1097/DBP.0000000000000107

Doyle Lauren R, 2015, Curr Dev Disord Rep, V2, P175

Duval-White CJ, 2013, AM J OCCUP THER, V67, P534, DOI 10.5014/ajot.2013.008243

Fitzpatrick JP, 2015, DRUG ALCOHOL REV, V34, P329, DOI 10.1111/dar.12232

Fitzpatrick JP, 2013, BMC PEDIATR, V13, DOI 10.1186/1471-2431-13-33

Fitzpatrick JP, 2012, BMJ OPEN, V2, DOI 10.1136/bmjopen-2012-000968

JANZEN LA, 1995, NEUROTOXICOL TERATOL, V17, P273, DOI 10.1016/0892-0362(94)00063-J

Jones KL, 2010, AM J MED GENET A, V152A, P2731, DOI 10.1002/ajmg.a.33675

Korkman M, 2003, CHILD NEUROPSYCHOL, V9, P117, DOI 10.1076/chin.9.2.117.14503

Kulp MT, 1999, OPTOMETRY VISION SCI, V76, P159, DOI 10.1097/00006324-199903000-00015

Lucas BR, 2016, DRUG ALCOHOL REV

MARCUS JC, 1987, NEUROPEDIATRICS, V18, P158, DOI 10.1055/s-2008-1052471

Mattson SN, 2006, NEUROPSYCHOLOGY, V20, P361, DOI 10.1037/0894-4105.20.3.361

Mattson SN, 1998, NEUROPSYCHOLOGY, V12, P146, DOI 10.1037/0894-4105.12.1.146

May PA, 2014, PEDIATRICS, V134, P855, DOI 10.1542/peds.2013-3319

Milner A. D., 2006, VISUAL BRAIN ACTION

Morphy F, 2010, POPULATION PEOPLE PL

Portney LG., 2000, FDN CLIN RES APPL PR

Riley EP, 2011, NEUROPSYCHOL REV, V21, P73, DOI 10.1007/s11065-011-9166-x

Satz P., 1982, FLORIDA KINDERGARTEN

Schneck C. M., 2010, OCCUPATIONAL THERAPY, P373

Simmons RW, 2002, ALCOHOL CLIN EXP RES, V26, P1412, DOI 10.1097/01.ALC.0000030563.14827.29

Sowell ER, 2008, J NEUROSCI, V28, P1313, DOI 10.1523/JNEUROSCI.5067-07.2008

Sowell ER, 2002, CEREB CORTEX, V12, P856, DOI 10.1093/cercor/12.8.856

Streissguth AP, 2004, J DEV BEHAV PEDIATR, V25, P228, DOI 10.1097/00004703-200408000-00002

Stromland K, 2004, ADDICT BIOL, V9, P153, DOI 10.1080/13556210410001717024

Thorley M, 2011, AUST OCCUP THER J, V58, P3, DOI 10.1111/j.1440-1630.2010.00852.x

Tomchek S. D., 2006, HAND FUNCTION CHILD, P293

Uecker A, 1996, NEUROPSYCHOLOGIA, V34, P209, DOI 10.1016/0028-3932(95)00096-8

Volman MJM, 2006, AM J OCCUP THER, V60, P451, DOI 10.5014/ajot.60.4.451

WEIL MJ, 1994, AM J OCCUP THER, V48, P982, DOI 10.5014/ajot.48.11.982

Willford JA, 2010, NEUROTOXICOL TERATOL, V32, P580, DOI 10.1016/j.ntt.2010.06.004

NR 52

TC 12

Z9 13

U1 1

U2 28

PU PERGAMON-ELSEVIER SCIENCE LTD

PI OXFORD

PA THE BOULEVARD, LANGFORD LANE, KIDLINGTON, OXFORD OX5 1GB, ENGLAND

SN 0891-4222

J9 RES DEV DISABIL

JI Res. Dev. Disabil.

PD AUG

PY 2016

VL 55

BP 346

EP 357

DI 10.1016/j.ridd.2016.05.009

PG 12

WC Education, Special; Rehabilitation

WE Social Science Citation Index (SSCI)

SC Education & Educational Research; Rehabilitation

GA DP4GT

UT WOS:000378455100031

PM 27228005

DA 2022-08-17

ER

PT J

AU Nakagawa, A

Sukigara, M

Miyachi, T

Nakai, A

AF Nakagawa, Atsuko

Sukigara, Masune

Miyachi, Taishi

Nakai, Akio

TI Relations between Temperament, Sensory Processing, and Motor

Coordination in 3-Year-Old Children

SO FRONTIERS IN PSYCHOLOGY

LA English

DT Article

DE temperament; sensory processing; motor coordination; effortful control;

3-year-olds

ID SELF-REGULATION; SCHOOL-AGE; PERSONALITY; ATTENTION; BEHAVIOR;

QUESTIONNAIRE; DISORDERS; INFANTS

AB Poor motor skills and differences in sensory processing have been noted as behavioral markers of common neurodevelopmental disorders. A total of 171 healthy children (81 girls, 90 boys) were investigated at age 3 to examine relations between temperament, sensory processing, and motor coordination. Using the Japanese versions of the Children's Behavior Questionnaire (CBQ), the Sensory Profile (SP-J), and the Little Developmental Coordination Disorder Questionnaire (LDCDQ), this study examines an expanded model based on Rothbart's three-factor temperamental theory (surgency, negative affect, effortful control) through covariance structure analysis. The results indicate that effortful control affects both sensory processing and motor coordination. The subscale of the LDCDQ, control during movement, is also influenced by surgency, while temperamental negative affect and surgency each have an effect on subscales of the SP-J.

C1 [Nakagawa, Atsuko; Sukigara, Masune] Nagoya City Univ, Sch Humanities & Social Sci, Nagoya, Aichi, Japan.

[Miyachi, Taishi] Nagoya Western Care Ctr Disabled Children, Nagoya, Aichi, Japan.

[Nakai, Akio] Hyogo Childrens Sleep & Dev Med Res Ctr, Dept Pediat, Dept Pediat Neurol, Kobe, Hyogo, Japan.

RP Nakagawa, A (通讯作者)，Nagoya City Univ, Sch Humanities & Social Sci, Nagoya, Aichi, Japan.

EM nakagawa@hum.nagoya-cu.ac.jp

RI Nakai, Akio/AAE-1124-2020

FU Ministry of Education, Culture, Sports, Science, and Technology of Japan

[25285185, 16H03733]

FX this study was supported by a Grant-in-Aid (No. 25285185, 16H03733) for

Scientific Research from the Ministry of Education, Culture, Sports,

Science, and Technology of Japan. Special thanks go to all the infants

and families who took part in this project.

CR Anckarsater H, 2006, AM J PSYCHIAT, V163, P1239, DOI 10.1176/appi.ajp.163.7.1239

Bundy A., 2002, SENSORY INTEGRATION, V2

Case-Smith J, 1998, AM J OCCUP THER, V52, P547, DOI 10.5014/ajot.52.7.547

Colombo J, 2001, ANNU REV PSYCHOL, V52, P337, DOI 10.1146/annurev.psych.52.1.337

DeSantis A, 2011, INFANT BEHAV DEV, V34, P280, DOI 10.1016/j.infbeh.2011.01.003

Dunn W, 2001, AM J OCCUP THER, V55, P608, DOI 10.5014/ajot.55.6.608

Dunn W, 2006, SENSORY PROFILE SUPP

Dunn W., 1999, SENSORY PROFILE USER

Gibbs J, 2007, ARCH DIS CHILD, V92, P534, DOI 10.1136/adc.2005.088054

Gouze KR, 2012, INFANT MENT HEALTH J, V33, P620, DOI 10.1002/imhj.21363

Grist CL, 2010, INFANT CHILD DEV, V19, P264, DOI 10.1002/icd.663

Ito H., 2013, SEISHINIGAKU, V55, P537

Johnson MH, 2015, J CHILD PSYCHOL PSYC, V56, P228, DOI 10.1111/jcpp.12328

Johnson MH, 2012, TRENDS COGN SCI, V16, P454, DOI 10.1016/j.tics.2012.07.001

Kusanagi E., 1993, ANN REPORT RES CLIN, V15, P25

Misra G, 2015, CEREB CORTEX, V25, P1906, DOI 10.1093/cercor/bhu001

Moreira RS, 2014, J PEDIAT-BRAZIL, V90, P119, DOI 10.1016/j.jped.2013.05.010

Nakagawa A, 2015, BIENN M SOC RES CHIL

Nakagawa A, 2013, INFANT BEHAV DEV, V36, P517, DOI 10.1016/j.infbeh.2013.04.002

Nakai A., 2012, NO TO HATTATSU, V44, P256

Posner MI, 2000, DEV PSYCHOPATHOL, V12, P427, DOI 10.1017/S0954579400003096

Posner MI, 1998, PHILOS T R SOC B, V353, P1915, DOI 10.1098/rstb.1998.0344

Posner MI, 2012, DEV PSYCHOL, V48, P827, DOI 10.1037/a0025530

Rihtman T, 2011, RES DEV DISABIL, V32, P1378, DOI 10.1016/j.ridd.2010.12.040

Rothbart M. K., 2011, BECOMING WE ARE TEMP

Rothbart MK, 2001, CHILD DEV, V72, P1394, DOI 10.1111/1467-8624.00355

Rothbart MK, 2000, J PERS SOC PSYCHOL, V78, P122, DOI 10.1037/0022-3514.78.1.122

Rothbart MK, 2007, HDB CHILD PSYCHOL, V3, P99, DOI [10.1002/9780470147658.chpsy0303, DOI 10.1002/9780470147658.CHPSY0303]

Tofighi D, 2011, BEHAV RES METHODS, V43, P692, DOI 10.3758/s13428-011-0076-x

van Baar AL, 2009, PEDIATRICS, V124, P251, DOI 10.1542/peds.2008-2315

NR 30

TC 12

Z9 13

U1 0

U2 13

PU FRONTIERS MEDIA SA

PI LAUSANNE

PA PO BOX 110, EPFL INNOVATION PARK, BUILDING I, LAUSANNE, 1015,

SWITZERLAND

SN 1664-1078

J9 FRONT PSYCHOL

JI Front. Psychol.

PD APR 29

PY 2016

VL 7

AR 623

DI 10.3389/fpsyg.2016.00623

PG 7

WC Psychology, Multidisciplinary

WE Social Science Citation Index (SSCI)

SC Psychology

GA DK7MY

UT WOS:000375111000001

PM 27199852

OA Green Published, gold

DA 2022-08-17

ER

PT J

AU Bernardi, M

Leonard, HC

Hill, EL

Henry, LA

AF Bernardi, Marialivia

Leonard, Hayley C.

Hill, Elisabeth L.

Henry, Lucy A.

TI Brief report: Response inhibition and processing speed in children with

motor difficulties and developmental coordination disorder

SO CHILD NEUROPSYCHOLOGY

LA English

DT Article

DE Developmental Coordination Disorder; Response inhibition; Processing

speed; Motor difficulties; Executive functioning

ID LANGUAGE IMPAIRMENT; ABILITY

AB A previous study reported that children with poor motor skills, classified as having motor difficulties (MD) or Developmental Coordination Disorder (DCD), produced more errors in a motor response inhibition task compared to typically developing (TD) children but did not differ in verbal inhibition errors. The present study investigated whether these groups differed in the length of time they took to respond in order to achieve these levels of accuracy, and whether any differences in response speed could be explained by generally slow information processing in children with poor motor skills. Timing data from the Verbal Inhibition Motor Inhibition test were analyzed to identify differences in performance between the groups on verbal and motor inhibition, as well as on processing speed measures from standardized batteries. Although children with MD and DCD produced more errors in the motor inhibition task than TD children, the current analyses found that they did not take longer to complete the task. Children with DCD were slower at inhibiting verbal responses than TD children, while the MD group seemed to perform at an intermediate level between the other groups in terms of verbal inhibition speed. Slow processing speed did not account for these group differences. Results extended previous research into response inhibition in children with poor motor skills by explicitly comparing motor and verbal responses, and suggesting that slow performance, even when accurate, may be attributable to an inefficient way of inhibiting responses, rather than slow information processing speed per se.

C1 [Bernardi, Marialivia; Henry, Lucy A.] City Univ London, Div Language & Commun Sci, London EC1V 0HB, England.

[Leonard, Hayley C.; Hill, Elisabeth L.] Univ London, Dept Psychol, London WC1E 7HU, England.

RP Bernardi, M (通讯作者)，City Univ London, Div Language & Commun Sci, London EC1V 0HB, England.

EM Marialivia.Bernardi@city.ac.uk

OI Hill, Elisabeth/0000-0003-3130-1271; Bernardi,

Marialivia/0000-0002-6844-0358

FU Waterloo Foundation [1121/1555]

FX This work was supported by The Waterloo Foundation [Grant no.

1121/1555], awarded to L. Henry at London South Bank University.

CR American Psychiatric Association, 2013, DIAGN STAT MAN MENT, V5th edition, DOI [10.1176/appi.books.9780890425596, DOI 10.1176/APPI.BOOKS.9780890425596]

Bishop DVM, 2002, AM J MED GENET, V114, P56, DOI 10.1002/ajmg.1630

Cambridge Cognition, 2006, CAMBR NEUR TEST AUT

Delis D.C., 2001, DELIS KAPLAN EXECUTI

Elliot C.D., 2011, BRIT ABILITY SCALES, V3rd Edn

Goodman R, 1997, J CHILD PSYCHOL PSYC, V38, P581, DOI 10.1111/j.1469-7610.1997.tb01545.x

HENDERSON L, 1992, J CHILD PSYCHOL PSYC, V33, P895, DOI 10.1111/j.1469-7610.1992.tb01963.x

Henderson S., 2007, MOVEMENT ASSESSMENT

Henry LA, 2012, J CHILD PSYCHOL PSYC, V53, P37, DOI 10.1111/j.1469-7610.2011.02430.x

Ho AK, 2010, HUM MOVEMENT SCI, V29, P605, DOI 10.1016/j.humov.2010.01.007

Leonard HC, 2015, DEV NEUROPSYCHOL, V40, P201, DOI 10.1080/87565641.2014.997933

Lingam R, 2009, PEDIATRICS, V123, pE693, DOI 10.1542/peds.2008-1770

Mandich A, 2002, BRAIN COGNITION, V50, P150, DOI 10.1016/S0278-2626(02)00020-9

Nigg JT, 2000, PSYCHOL BULL, V126, P220, DOI 10.1037/0033-2909.126.2.220

Piek JP, 2007, DEV MED CHILD NEUROL, V49, P678, DOI 10.1111/j.1469-8749.2007.00678.x

Piek JP, 1999, J INT NEUROPSYCH SOC, V5, P320, DOI 10.1017/S1355617799544032

Pitcher TM, 2003, DEV MED CHILD NEUROL, V45, P525, DOI 10.1111/j.1469-8749.2003.tb00952.x

Querne L, 2008, BRAIN RES, V1244, P89, DOI 10.1016/j.brainres.2008.07.066

TORGENSEN JK, 1999, TEST WORD READING EF

Wilmut K, 2010, DEV MED CHILD NEUROL, V52, P229, DOI 10.1111/j.1469-8749.2009.03372.x

Wilson PH, 1998, J CHILD PSYCHOL PSYC, V39, P829, DOI 10.1111/1469-7610.00384

NR 21

TC 14

Z9 14

U1 1

U2 14

PU ROUTLEDGE JOURNALS, TAYLOR & FRANCIS LTD

PI ABINGDON

PA 4 PARK SQUARE, MILTON PARK, ABINGDON OX14 4RN, OXFORDSHIRE, ENGLAND

SN 0929-7049

EI 1744-4136

J9 CHILD NEUROPSYCHOL

JI Child Neuropsychol.

PD JUL 3

PY 2016

VL 22

IS 5

BP 627

EP 634

DI 10.1080/09297049.2015.1014898

PG 8

WC Clinical Neurology

WE Science Citation Index Expanded (SCI-EXPANDED); Social Science Citation Index (SSCI)

SC Neurosciences & Neurology

GA DJ0FC

UT WOS:000373876200008

PM 25761255

OA Green Accepted

DA 2022-08-17

ER

PT J

AU Lucas, BR

Elliott, EJ

Coggan, S

Pinto, RZ

Jirikowic, T

McCoy, SW

Latimer, J

AF Lucas, Barbara R.

Elliott, Elizabeth J.

Coggan, Sarah

Pinto, Rafael Z.

Jirikowic, Tracy

McCoy, Sarah Westcott

Latimer, Jane

TI Interventions to improve gross motor performance in children with

neurodevelopmental disorders: a meta- analysis

SO BMC PEDIATRICS

LA English

DT Article

DE Neurodevelopmental disorders; Motor skills disorders; Motor skills;

Child development; Physiotherapy; Cerebral palsy; Developmental

Coordination Disorder

ID DEVELOPMENTAL COORDINATION DISORDER; CEREBRAL-PALSY; PHYSICAL-THERAPY;

BALANCE CONTROL; PEDRO SCALE; QUALITY; RELIABILITY; STRENGTH; OUTCOMES;

TRIALS

AB Background: Gross motor skills are fundamental to childhood development. The effectiveness of current physical therapy options for children with mild to moderate gross motor disorders is unknown. The aim of this study was to systematically review the literature to investigate the effectiveness of conservative interventions to improve gross motor performance in children with a range of neurodevelopmental disorders.

Methods: A systematic review with meta-analysis was conducted. MEDLINE, EMBASE, AMED, CINAHL, PsycINFO, PEDro, Cochrane Collaboration, Google Scholar databases and clinical trial registries were searched. Published randomised controlled trials including children 3 to <= 18 years with (i) Developmental Coordination Disorder (DCD) or Cerebral Palsy (CP) (Gross Motor Function Classification System Level 1) or Developmental Delay or Minimal Acquired Brain Injury or Prematurity (<30 weeks gestational age) or Fetal Alcohol Spectrum Disorders; and (ii) receiving non-pharmacological or non-surgical interventions from a health professional and (iii) gross motor outcomes obtained using a standardised assessment tool. Meta-analysis was performed to determine the pooled effect of intervention on gross motor function. Methodological quality and strength of meta-analysis recommendations were evaluated using PEDro and the GRADE approach respectively.

Results: Of 2513 papers, 9 met inclusion criteria including children with CP (n = 2) or DCD (n = 7) receiving 11 different interventions. Only two of 9 trials showed an effect for treatment. Using the least conservative trial outcomes a large beneficial effect of intervention was shown (SMD:-0.8; 95% CI:-1.1 to -0.5) with "very low quality" GRADE ratings. Using the most conservative trial outcomes there is no treatment effect (SMD:-0.1; 95% CI:-0.3 to 0.2) with "low quality" GRADE ratings. Study limitations included the small number and poor quality of the available trials.

Conclusion: Although we found that some interventions with a task-orientated framework can improve gross motor outcomes in children with DCD or CP, these findings are limited by the very low quality of the available evidence. High quality intervention trials are urgently needed.

C1 [Lucas, Barbara R.; Elliott, Elizabeth J.] Univ Sydney, Childrens Hosp Westmead, Sch Clin, Discipline Paediat & Child Hlth, Locked Bag 4001, Sydney, NSW 2145, Australia.

[Lucas, Barbara R.; Elliott, Elizabeth J.; Coggan, Sarah; Latimer, Jane] Univ Sydney, Sydney Med Sch, George Inst Global Hlth, POB M201,Missenden Rd, Sydney, NSW 2050, Australia.

[Lucas, Barbara R.] Univ Sydney, Sydney Sch Publ Hlth, Poche Ctr Indigenous Hlth, Sydney, NSW 2006, Australia.

[Lucas, Barbara R.] Royal North Shore Hosp, Physiotherapy Dept, Sydney, NSW 2065, Australia.

[Elliott, Elizabeth J.] Sydney Childrens Hosp Networks Westmead, Locked Bag 4001, Sydney, NSW 2145, Australia.

[Coggan, Sarah] Curtin Univ, Sch Publ Hlth, GPO Box U1987, Perth, WA 6845, Australia.

[Pinto, Rafael Z.] Univ Sydney, Royal North Shore Hosp, Pain Management Res Inst, Sydney, NSW 2065, Australia.

[Pinto, Rafael Z.] UNESP Univ Estadual Paulista, Fac Ciencias & Tecnol, Dept Fisioterapia, BR-19060900 Prudente, SP, Brazil.

[Jirikowic, Tracy] Univ Washington, Dept Rehabil Med, Div Occupat Therapy, Seattle, WA 98195 USA.

[McCoy, Sarah Westcott] Univ Washington, Dept Rehabil Med, Div Phys Therapy, Seattle, WA 98195 USA.

RP Lucas, BR (通讯作者)，Univ Sydney, Childrens Hosp Westmead, Sch Clin, Discipline Paediat & Child Hlth, Locked Bag 4001, Sydney, NSW 2145, Australia.; Lucas, BR (通讯作者)，Univ Sydney, Sydney Med Sch, George Inst Global Hlth, POB M201,Missenden Rd, Sydney, NSW 2050, Australia.; Lucas, BR (通讯作者)，Univ Sydney, Sydney Sch Publ Hlth, Poche Ctr Indigenous Hlth, Sydney, NSW 2006, Australia.; Lucas, BR (通讯作者)，Royal North Shore Hosp, Physiotherapy Dept, Sydney, NSW 2065, Australia.

EM blucas@georgeinstitute.org.au

RI Pinto, Rafael Zambelli/I-2892-2012

OI Pinto, Rafael Zambelli/0000-0002-2775-860X; Elliott,

Elizabeth/0000-0002-6501-5487

FU Poche Centre for Indigenous Health, Sydney Medical School, The

University of Sydney, Sydney, Australia; National Health and Medical

Research Council of Australia [1021480]; Australian Federal Government

(Departments of Health and Ageing, and Families, Housing, Community

Services and Indigenous Affairs); National Institute on Alcohol Abuse

and Alcoholism [R21AA019579, R33AA019579-03]; Australian Research

Council [0130007]; NATIONAL INSTITUTE ON ALCOHOL ABUSE AND ALCOHOLISM

[R33AA019579, R21AA019579] Funding Source: NIH RePORTER

FX The authors have no financial relationships relevant to this article to

disclose. Funding support was received as follows: Ms BR Lucas:

supported by a Fellowship from the Poche Centre for Indigenous Health,

Sydney Medical School, The University of Sydney, Sydney, Australia.

Prof. EJ Elliott: supported by a National Health and Medical Research

Council of Australia Practitioner Fellowship (No 1021480). Dr RZ Pinto:

supported by Australian Federal Government (Departments of Health and

Ageing, and Families, Housing, Community Services and Indigenous

Affairs). Associate Prof T. Jirikowic: supported by grants from The

National Institute on Alcohol Abuse and Alcoholism (R21AA019579,

R33AA019579-03). Prof S.W. McCoy: supported by grants from The National

Institute on Alcohol Abuse and Alcoholism (R21AA019579, R33AA019579-03).

Prof. J Latimer: supported by an Australian Research Council Future

Fellowship (No 0130007).

CR American Psychiatric Association, 2013, DIAGNOSTIC STAT MANU, DOI [10.1176/appi.books.9780890425596, DOI 10.1176/APPI.BOOKS.9780890425596]

[Anonymous], 1997, MMWR Recomm Rep, V46, P1

Arciniegas David B, 2005, Neuropsychiatr Dis Treat, V1, P311

Australian Institute of Health and Welfare, 2012, PICT AUSTR CHILDR 20, P57

Balshem H, 2011, J CLIN EPIDEMIOL, V64, P401, DOI 10.1016/j.jclinepi.2010.07.015

Bland DC, 2011, BRAIN INJURY, V25, P664, DOI 10.3109/02699052.2011.576306

Blank R, 2012, DEV MED CHILD NEUROL, V54, P54, DOI 10.1111/j.1469-8749.2011.04171.x

CanChild Resources, GROSS MOT FUNCT CLAS

Chrysagis N, 2012, AM J PHYS MED REHAB, V91, P747, DOI 10.1097/PHM.0b013e3182643eba

Cochrane Collaboration, 2011, COCHR HDB SYST REV I

Cohen J., 1988, STAT POWER ANAL SOCI

de Morton NA, 2009, AUST J PHYSIOTHER, V55, P129, DOI 10.1016/S0004-9514(09)70043-1

Delamothe T, 2004, BMJ-BRIT MED J, V328, P1, DOI 10.1136/bmj.328.7430.1

Eccles MP, 2011, IMPLEMENT SCI, V6, DOI 10.1186/1748-5908-6-32

Egger M, 1997, BMJ-BRIT MED J, V315, P629, DOI 10.1136/bmj.315.7109.629

Fong SSM, 2013, RES DEV DISABIL, V34, P1446, DOI 10.1016/j.ridd.2013.01.025

Fong SSM, 2012, RES DEV DISABIL, V33, P85, DOI 10.1016/j.ridd.2011.08.023

Green D, 2011, RES DEV DISABIL, V32, P1332, DOI 10.1016/j.ridd.2011.01.040

Hamilton GF, 2012, ALCOHOL CLIN EXP RES, V36, P1196, DOI 10.1111/j.1530-0277.2011.01726.x

Hammond J, 2014, CHILD CARE HLTH DEV, V40, P165, DOI 10.1111/cch.12029

Henschke N, 2010, COCHRANE DB SYST REV, DOI 10.1002/14651858.CD002014.pub3

Higgins JPT, 2003, BRIT MED J, V327, P557, DOI 10.1136/bmj.327.7414.557

Hillier S, 2010, PHYS OCCUP THER PEDI, V30, P111, DOI 10.3109/01942630903543575

Klintsova AY, 2002, BRAIN RES, V937, P83, DOI 10.1016/S0006-8993(02)02492-7

Kolehmainen N, 2011, BMC PEDIATR, V11, DOI 10.1186/1471-2431-11-100

Larin HM, 2006, MOTOR LEARNING THEOR

Laufer Y, 2011, J PHYS THER ED, V25, P59, DOI DOI 10.1097/00001416-201110000-00011

Ledebt A, 2005, MOTOR CONTROL, V9, P459, DOI 10.1123/mcj.9.4.459

Lucas BR, 2014, PEDIATRICS, V134, pE192, DOI 10.1542/peds.2013-3733

Macedo LG, 2010, J CLIN EPIDEMIOL, V63, P920, DOI 10.1016/j.jclinepi.2009.10.005

Maher CG, 2003, PHYS THER, V83, P713, DOI 10.1093/ptj/83.8.713

Mandich Angela D., 2001, Physical and Occupational Therapy in Pediatrics, V20, P51, DOI 10.1300/J006v20n02_04

Moher David, 2009, Ann Intern Med, V151, P264, DOI 10.1136/bmj.b2535

Morgan C, 2015, BMC PEDIATR, V15, DOI 10.1186/s12887-015-0347-2

Morgan C, 2013, PEDIATRICS, V132, pE735, DOI 10.1542/peds.2012-3985

Moseley AM, 2002, AUST J PHYSIOTHER, V48, P43, DOI 10.1016/S0004-9514(14)60281-6

Niemeijer AS, 2007, DEV MED CHILD NEUROL, V49, P406, DOI 10.1111/j.1469-8749.2007.00406.x

Palisano R, 1997, DEV MED CHILD NEUROL, V39, P214, DOI 10.1111/j.1469-8749.1997.tb07414.x

Peens A, 2008, CHILD CARE HLTH DEV, V34, P316, DOI 10.1111/j.1365-2214.2007.00803.x

Piek JP, 2008, HUM MOVEMENT SCI, V27, P668, DOI 10.1016/j.humov.2007.11.002

Pless M, 2000, ADAPT PHYS ACT Q, V17, P381, DOI 10.1123/apaq.17.4.381

POLATAJKO HJ, 1995, DEV MED CHILD NEUROL, V37, P310

Roberts G, 2011, DEV MED CHILD NEUROL, V53, P55, DOI 10.1111/j.1469-8749.2010.03779.x

Sandlund M, 2009, DEV MED CHILD NEUROL, V51, P173, DOI 10.1111/j.1469-8749.2008.03184.x

Shaheed CA, 2014, J PAIN, V15, P2, DOI 10.1016/j.jpain.2013.09.016

Shonkoff J.P., 2000, NEURONS NEIGHBORHOOD

Smits-Engelsman BCM, 2013, DEV MED CHILD NEUROL, V55, P229, DOI 10.1111/dmcn.12008

Sterne JAC, 2011, COCHRANE COLLAB

Sterne JAC, 2011, BMJ-BRIT MED J, V343, DOI 10.1136/bmj.d4002

Tsai CL, 2009, RES DEV DISABIL, V30, P1268, DOI 10.1016/j.ridd.2009.05.001

Vaccarino FM, 2004, ARCH DIS CHILD-FETAL, V89, pF190, DOI 10.1136/adc.2003.043661

Wagner JL, 2013, ALCOHOL CLIN EXP RES, V37, P1561, DOI 10.1111/acer.12122

Wang TN, 2009, DEV MED CHILD NEUROL, V51, P817, DOI 10.1111/j.1469-8749.2009.03271.x

Weingarten MA, 2004, BMJ-BRIT MED J, V328, P1013, DOI 10.1136/bmj.328.7446.1013

Williams J, 2010, DEV MED CHILD NEUROL, V52, P232, DOI 10.1111/j.1469-8749.2009.03544.x

NR 55

TC 36

Z9 37

U1 2

U2 34

PU BMC

PI LONDON

PA CAMPUS, 4 CRINAN ST, LONDON N1 9XW, ENGLAND

EI 1471-2431

J9 BMC PEDIATR

JI BMC Pediatr.

PD NOV 29

PY 2016

VL 16

AR 193

DI 10.1186/s12887-016-0731-6

PG 16

WC Pediatrics

WE Science Citation Index Expanded (SCI-EXPANDED); Social Science Citation Index (SSCI)

SC Pediatrics

GA ED5QR

UT WOS:000388907400001

PM 27899082

OA Green Published, gold

DA 2022-08-17

ER

PT J

AU Dyck, M

Piek, J

AF Dyck, Murray

Piek, Jan

TI How to distinguish normal from disordered children with poor language or

motor skills

SO INTERNATIONAL JOURNAL OF LANGUAGE & COMMUNICATION DISORDERS

LA English

DT Article

DE discrepancy criterion; cognitive development; developmental disorder;

Developmental Coordination Disorder; Mixed Receptive-Expressive Language

Disorder; language impairment; motor skills

ID DEVELOPMENTAL COORDINATION DISORDER; DEFICIT-HYPERACTIVITY DISORDER;

READING-DISABILITY; LEARNING-DISABILITIES; COGNITIVE-DEVELOPMENT;

WORKING-MEMORY; FRONTAL-LOBE; IMPAIRMENT; ATTENTION; ABILITY

AB Methods & Procedures: Children with a diagnosis of Mixed Receptive Expressive Language Disorder (RELD; n = 21) were compared with children with no previously suspected disorder but low standard language scores ( < 80; n = 22) selected from a representative sample, and children with Developmental Coordination Disorder (DCD; n = 20) were compared with children with no previously suspected disorder but low standard motor skills scores (n = 28) selected from a representative sample.

Outcomes & Results: Children with diagnosed disorders were more pervasive underachievers. The RELD group obtained lower scores on measures of verbal comprehension, emotion understanding, theory of mind, working memory and response inhibition; the DCD group obtained lower scores on measures of perceptual organization, verbal comprehension, receptive and expressive language, and visual inspection time.

Conclusions & Implications: We conclude that relatively pervasive underachievement distinguishes disordered from normal low achievers.

C1 [Dyck, Murray] Griffith Univ, Sch Psychol, Gold Coast, Qld 4222, Australia.

[Piek, Jan] Curtin Univ Technol, Perth, WA, Australia.

RP Dyck, M (通讯作者)，Griffith Univ, Sch Psychol, Gold Coast, Qld 4222, Australia.

EM m.dyck@griffith.edu.au

OI Piek, Jan/0000-0003-3838-6773

CR ANDERSON M, 1988, BRIT J DEV PSYCHOL, V6, P43, DOI 10.1111/j.2044-835X.1988.tb01079.x

APA, 2000, DIAGNOSTIC STAT MANU, V4

Archibald LMD, 2006, INT J LANG COMM DIS, V41, P675, DOI 10.1080/13682820500442602

BARONCOHEN S, 1985, COGNITION, V21, P37, DOI 10.1016/0010-0277(85)90022-8

Botting N, 2005, J CHILD PSYCHOL PSYC, V46, P317, DOI 10.1111/j.1469-7610.2004.00355.x

Brookes RL, 2007, NEUROPSYCHOLOGIA, V45, P1921, DOI 10.1016/j.neuropsychologia.2006.11.019

Cummins A, 2005, DEV MED CHILD NEUROL, V47, P437, DOI 10.1017/S001216220500085X

Dewey D, 2002, HUM MOVEMENT SCI, V21, P905, DOI 10.1016/S0167-9457(02)00163-X

Diamond A, 2000, CHILD DEV, V71, P44, DOI 10.1111/1467-8624.00117

Duncan J, 1996, COGNITIVE PSYCHOL, V30, P257, DOI 10.1006/cogp.1996.0008

Dyck MJ, 2006, J CLIN CHILD ADOLESC, V35, P20, DOI 10.1207/s15374424jccp3501_3

Dyck MJ, 2004, J CHILD PSYCHOL PSYC, V45, P979, DOI 10.1111/j.1469-7610.2004.t01-1-00290.x

Dyck MJ, 2004, J CHILD PSYCHOL PSYC, V45, P789, DOI 10.1111/j.1469-7610.2004.00272.x

Dyck MJ, 2001, EUR CHILD ADOLES PSY, V10, P105, DOI 10.1007/s007870170033

Francis DJ, 1996, J EDUC PSYCHOL, V88, P3, DOI 10.1037/0022-0663.88.1.3

Gonzalez JEJ, 1999, LEARN DISABILITY Q, V22, P291, DOI 10.2307/1511263

Green D, 2002, J CHILD PSYCHOL PSYC, V43, P655, DOI 10.1111/1469-7610.00054

HAPPE FGE, 1994, J AUTISM DEV DISORD, V24, P129, DOI 10.1007/BF02172093

Harris P., 1989, COGNITION EMOTION, V3, P379, DOI DOI 10.1080/02699938908412713

HENDERSON SE, 1992, MOVEMENT ASSESSMENT

HILL E, 2001, INT J LANG COMM DIS, V2, P149

Hill EL, 1998, DEV MED CHILD NEUROL, V40, P388

Kadesjo B, 1999, J AM ACAD CHILD PSY, V38, P820, DOI 10.1097/00004583-199907000-00011

Kaplan BJ, 1998, HUM MOVEMENT SCI, V17, P471, DOI 10.1016/S0167-9457(98)00010-4

Karmiloff-Smith A, 2003, CORTEX, V39, P161, DOI 10.1016/S0010-9452(08)70091-1

Karmiloff-Smith A, 2007, DEVELOPMENTAL SCI, V10, P84, DOI 10.1111/j.1467-7687.2007.00568.x

LARSON VL, 1995, LANGUAGE DISORDERS O

Matsumoto D., 1995, JAPANESE CAUCASIAN F

MCCARRON LT, 1997, MCCARRON ASSESSMENT

PERNER J, 1989, CHILD DEV, V60, P689, DOI 10.2307/1130734

Piek JP, 2007, DEV MED CHILD NEUROL, V49, P678, DOI 10.1111/j.1469-8749.2007.00678.x

Piek JP, 1999, DEV MED CHILD NEUROL, V41, P159, DOI 10.1017/S0012162299000341

Pitcher TM, 2003, DEV MED CHILD NEUROL, V45, P525, DOI 10.1111/j.1469-8749.2003.tb00952.x

Rabbit P., 1997, METHODOLOGY FRONTAL

Ramus F, 2003, J CHILD PSYCHOL PSYC, V44, P712, DOI 10.1111/1469-7610.00157

Semel E., 2006, CLIN EVALUATION LANG

SHUE KL, 1992, BRAIN COGNITION, V20, P104, DOI 10.1016/0278-2626(92)90064-S

STANOVICH KE, 1997, ED PSYCHOL PRACTICE, V13, P3, DOI DOI 10.1080/0266736970130101

Sternberg RJ, 2002, J SCHOOL PSYCHOL, V40, P65, DOI 10.1016/S0022-4405(01)00094-2

Ullman MT, 2005, CORTEX, V41, P399, DOI 10.1016/S0010-9452(08)70276-4

Vellutino FR, 2000, J LEARN DISABIL-US, V33, P223, DOI 10.1177/002221940003300302

Vellutino FR, 2004, J CHILD PSYCHOL PSYC, V45, P2, DOI 10.1046/j.0021-9630.2003.00305.x

Visser J, 2003, HUM MOVEMENT SCI, V22, P479, DOI 10.1016/j.humov.2003.09.005

Wechsler D., 1992, WECHSLER INTELLIGENC

Wilson PH, 2005, J CHILD PSYCHOL PSYC, V46, P806, DOI 10.1111/j.1469-7610.2005.01409.x

WIMMER H, 1983, COGNITION, V13, P103, DOI 10.1016/0010-0277(83)90004-5

Wisdom SN, 2007, EUR CHILD ADOLES PSY, V16, P178, DOI 10.1007/s00787-006-0586-8

Zoia S, 2006, CHILD CARE HLTH DEV, V32, P613, DOI 10.1111/j.1365-2214.2006.00697.x

NR 48

TC 8

Z9 8

U1 0

U2 23

PU WILEY-BLACKWELL

PI MALDEN

PA COMMERCE PLACE, 350 MAIN ST, MALDEN 02148, MA USA

SN 1368-2822

J9 INT J LANG COMM DIS

JI Int. J. Lang. Commun. Disord.

PD MAY

PY 2010

VL 45

IS 3

BP 336

EP 344

DI 10.3109/13682820903009503

PG 9

WC Audiology & Speech-Language Pathology; Linguistics; Rehabilitation

WE Social Science Citation Index (SSCI)

SC Audiology & Speech-Language Pathology; Linguistics; Rehabilitation

GA 587QA

UT WOS:000277006300006

PM 20144011

OA Green Published

DA 2022-08-17

ER

PT J

AU Miyachi, T

Nakai, A

Tani, I

Ohnishi, M

Nakajima, S

Tsuchiya, KJ

Matsumoto, K

Tsujii, M

AF Miyachi, Taishi

Nakai, Akio

Tani, Iori

Ohnishi, Masafumi

Nakajima, Shunji

Tsuchiya, Kenji J.

Matsumoto, Kaori

Tsujii, Masatsugu

TI Evaluation of Motor Coordination in Boys with High-Functioning Pervasive

Developmental Disorder Using the Japanese Version of the Developmental

Coordination Disorder Questionnaire

SO JOURNAL OF DEVELOPMENTAL AND PHYSICAL DISABILITIES

LA English

DT Article

DE High-functioning pervasive developmental disorder (HFPDD); Developmental

coordination disorder (DCD); Developmental coordination disorder

questionnaire (DCDQ); Motor coordination dysfunction; Autism diagnostic

interview-revised (ADI-R); Questionnaire

ID AUTISM SPECTRUM DISORDERS; ASPERGER-SYNDROME; DIAGNOSTIC INTERVIEW;

CHILDREN; CLUMSINESS; METAANALYSIS; IMPAIRMENTS; ADOLESCENTS;

PERCEPTIONS; RELIABILITY

AB Children with high-functioning pervasive developmental disorder (HFPDD) often have motor coordination dysfunction. However, there is no assessment tool for screening developmental coordination disorder (DCD) in Japan, which makes it difficult to evaluate the actual motor impairments of children with HFPDD. We evaluated the motor coordination function of 54 school-age boys with HFPDD using the Japanese version of the Developmental Coordination Disorder Questionnaire (DCDQ-J). We subsequently assessed the relationship between DCDQ-J scores and the results of the Japanese version of the Autism Diagnostic Interview-Revised (ADI-R) of 48 boys. The total and subscale DCDQ-J scores of the boys with HFPDD were significantly lower than the population means in the same grade: 37.0 % were below 2 standard deviations for the total score, 38.9 % for control during movement, 26.0 % for fine motor/handwriting, and 37.0 % for general coordination. Furthermore, the scores of Qualitative Abnormalities in Communication in the ADI-R were negatively correlated with control during movement, fine motor/handwriting, and total scores in the DCDQ-J. This study is the first to show Japanese children with HFPDD frequently exhibit considerably poor motor coordination according to the DCDQ-J. The screening or assessment of motor dysfunction in HFPDD using assessment tools such as the DCDQ could aid the development of interventions for these underestimated problems in Japan.

C1 [Miyachi, Taishi] Nagoya Cent Rehabil Ctr, Dept Pediat, Nagoya, Aichi, Japan.

[Nakai, Akio] Hyogo Childrens Sleep & Dev Med Res Ctr, Nishi Ku, Kobe, Hyogo 6512181, Japan.

[Tani, Iori] Tokai Gakuen Univ, Sch Humanities, Nagoya, Aichi, Japan.

[Ohnishi, Masafumi] Univ Fukui, Fac Educ & Reg Studies, Fukui 910, Japan.

[Nakajima, Shunji; Tsuchiya, Kenji J.; Matsumoto, Kaori] Hamamatsu Univ Sch Med, Res Ctr Child Mental Dev, Hamamatsu, Shizuoka 4313192, Japan.

[Tsujii, Masatsugu] Chukyo Univ, Sch Contemporary Sociol, Nagoya, Aichi, Japan.

RP Nakai, A (通讯作者)，Hyogo Childrens Sleep & Dev Med Res Ctr, Nishi Ku, Akebono Cho 1070, Kobe, Hyogo 6512181, Japan.

EM anakai.kodomo@gmail.com

RI Tsuchiya, Kenji J./I-9123-2019; Nakai, Akio/AAE-1124-2020

OI Tsuchiya, Kenji J./0000-0002-1314-4199; TANI, Iori/0000-0003-2329-4518

CR Aman MG, 2009, RES DEV DISABIL, V30, P386, DOI 10.1016/j.ridd.2008.07.004

American Psychiatric Association, 2013, DIAGN STAT MAN MENT, V5th edition, DOI [10.1176/appi.books.9780890425596, DOI 10.1176/APPI.BOOKS.9780890425596]

American Psychological Association, 2000, DIAGN STAT MAN MENT, DOI DOI 10.1176/APPI.BOOKS.9780890423349

Attwood T., 1998, ASPERGERS SYNDROME G, P13

Bart O, 2013, RES DEV DISABIL, V34, P1922, DOI 10.1016/j.ridd.2013.03.015

Blank R, 2012, DEV MED CHILD NEUROL, V54, P54, DOI 10.1111/j.1469-8749.2011.04171.x

Braathen G, 1997, ACTA PAEDIATR, V86, P372, DOI 10.1111/j.1651-2227.1997.tb09025.x

Dziuk MA, 2007, DEV MED CHILD NEUROL, V49, P734, DOI 10.1111/j.1469-8749.2007.00734.x

Enticott PG, 2009, DEV MED CHILD NEUROL, V51, P787, DOI 10.1111/j.1469-8749.2009.03270.x

Farkas Z, 2010, SEIZURE-EUR J EPILEP, V19, P173, DOI 10.1016/j.seizure.2010.01.013

Fournier KA, 2010, J AUTISM DEV DISORD, V40, P1227, DOI 10.1007/s10803-010-0981-3

Ghaziuddin M, 1998, J INTELL DISABIL RES, V42, P43, DOI 10.1046/j.1365-2788.1998.00065.x

GILLBERG IC, 1989, J CHILD PSYCHOL PSYC, V30, P631, DOI 10.1111/j.1469-7610.1989.tb00275.x

Green D, 2009, DEV MED CHILD NEUROL, V51, P311, DOI 10.1111/j.1469-8749.2008.03242.x

Haswell CC, 2009, NAT NEUROSCI, V12, P970, DOI 10.1038/nn.2356

HENDERSON SE, 1992, MOVEMENT ASSESSMENT

Japanese WISC-III Publication Committee, 1998, NIH WISC 3 CHIN KENS

Kokubun M., 2014, INT J DEV D IN PRESS

Kopp S, 2010, RES DEV DISABIL, V31, P350, DOI 10.1016/j.ridd.2009.09.017

Kushki A, 2011, J AUTISM DEV DISORD, V41, P1706, DOI 10.1007/s10803-011-1206-0

LORD C, 1994, J AUTISM DEV DISORD, V24, P659, DOI 10.1007/BF02172145

Loubinoux I, 2005, NEUROIMAGE, V27, P299, DOI 10.1016/j.neuroimage.2004.12.023

Missiuna Cheryl, 2006, Can J Occup Ther, V73, P7

Murdoch BE, 2010, CORTEX, V46, P858, DOI 10.1016/j.cortex.2009.07.018

Nakai A, 2011, RES DEV DISABIL, V32, P1615, DOI 10.1016/j.ridd.2011.02.012

Piek JP, 2006, HUM MOVEMENT SCI, V25, P65, DOI 10.1016/j.humov.2005.10.011

Polatajko Helene J, 2005, Semin Pediatr Neurol, V12, P250

Rivard LM, 2007, BRIT J EDUC PSYCHOL, V77, P633, DOI 10.1348/000709906X159879

Skinner RA, 2001, HUM MOVEMENT SCI, V20, P73, DOI 10.1016/S0167-9457(01)00029-X

Stanfield AC, 2008, EUR PSYCHIAT, V23, P289, DOI 10.1016/j.eurpsy.2007.05.006

Stephenson EA, 2008, CHILD CARE HLTH DEV, V34, P335, DOI 10.1111/j.1365-2214.2007.00805.x

Sturm H, 2004, DEV MED CHILD NEUROL, V46, P444, DOI 10.1017/S0012162204000738

Tsuchiya KJ, 2013, J AUTISM DEV DISORD, V43, P643, DOI 10.1007/s10803-012-1606-9

Vakalopoulos C, 2013, CEREBELLUM, V12, P212, DOI 10.1007/s12311-012-0416-0

Wilson BN, 2000, AM J OCCUP THER, V54, P484, DOI 10.5014/ajot.54.5.484

Wilson BN, 2009, PHYS OCCUP THER PEDI, V29, P182, DOI 10.1080/01942630902784761

NR 36

TC 4

Z9 4

U1 1

U2 12

PU SPRINGER/PLENUM PUBLISHERS

PI NEW YORK

PA 233 SPRING ST, NEW YORK, NY 10013 USA

SN 1056-263X

EI 1573-3580

J9 J DEV PHYS DISABIL

JI J. Dev. Phys. Disabil.

PD AUG

PY 2014

VL 26

IS 4

BP 403

EP 413

DI 10.1007/s10882-014-9377-1

PG 11

WC Education, Special; Psychology, Developmental; Rehabilitation

WE Social Science Citation Index (SSCI)

SC Education & Educational Research; Psychology; Rehabilitation

GA AK9XZ

UT WOS:000338783200003

DA 2022-08-17

ER

PT J

AU Schott, N

Holfelder, B

AF Schott, N.

Holfelder, B.

TI Relationship between motor skill competency and executive function in

children with Down's syndrome

SO JOURNAL OF INTELLECTUAL DISABILITY RESEARCH

LA English

DT Article

DE Down's syndrome; executive function; intellectual disability; motor

skill competency; TGMD-2; TMT

ID FUNDAMENTAL MOVEMENT SKILLS; INDIVIDUAL-DIFFERENCES; POPULATION

PREVALENCE; COGNITIVE-DEVELOPMENT; PERFORMANCE; ADOLESCENTS;

COORDINATION; WILLIAMS; VALIDITY; BRAIN

AB BackgroundPrevious studies suggest that children with Down's syndrome (DS), a genetically based neurodevelopmental disorder, demonstrate motor problems and cognitive deficits. The first aim of this study was to examine motor skills and executive functions (EFs) in school-age children with DS. The second aim was to investigate the relationship between these two performance domains.

MethodsThe Test of Gross Motor Development (TGMD-2), the Movement Assessment Battery Children-2 checklist (MABC2-checklist) and the Trail-Making Test for young children (Trails-P) were used to assess motor and cognitive performances of 18 children (11 boys, 7 girls) with DS aged between 7 and 11 years (9.060.96) and an age- and sex-matched sample of 18 typically developing (TD) children (11 boys, 7 girls; 8.990.93).

ResultsIndividuals with DS showed the expected difficulties in attentional control, response suppression and distraction, as well as in locomotor and object control skills, as indicated by poorer performance than TD individuals. Motor performance (bottom-up as well as top-down measures) and EF correlated positively, with regard to the group with DS only though. In the most complex task (distraction), the children of the DS group achieving lower locomotor scores showed lower efficacy scores on the Trails-P. Additionally, strong relationships were found for the perspective of teachers on all sections of the MABC2-Checklist and EF.

ConclusionThe findings from this study suggest that children with DS are not only impaired in higher-order EF, but showing also deficits in locomotor and object control skills. This study stresses the importance of early interventions facilitating cognitive abilities and motor skills.

C1 [Schott, N.; Holfelder, B.] Univ Stuttgart, Dept Sport & Exercise Sci, D-70569 Stuttgart, Germany.

RP Schott, N (通讯作者)，Univ Stuttgart, Dept Sport & Exercise Sci, Allmandring 28, D-70569 Stuttgart, Germany.

EM nadja.schott@inspo.uni-stuttgart.de

OI Schott, Nadja/0000-0002-0922-1374

CR Barr M, 2011, J INTELL DISABIL RES, V55, P1020, DOI 10.1111/j.1365-2788.2011.01425.x

Best JR, 2010, CHILD DEV, V81, P1641, DOI 10.1111/j.1467-8624.2010.01499.x

Borella E, 2013, RES DEV DISABIL, V34, P65, DOI 10.1016/j.ridd.2012.07.017

Brown T, 2012, OCCUP THER HEALTH CA, V26, P283, DOI 10.3109/07380577.2012.722749

Burack JA, 2002, DEV PSYCHOPATHOL, V14, P225, DOI 10.1017/S095457940200202X

Capio CM, 2010, J EXERC SCI FIT, V8, P17, DOI 10.1016/S1728-869X(10)60003-2

Carducci F, 2013, FUNCT NEUROL, V28, P19, DOI 10.11138/FNeur/2013.28.1.019

Carney DPJ, 2013, RES DEV DISABIL, V34, P46, DOI 10.1016/j.ridd.2012.07.013

Carvalho RL, 2011, PRENATAL DIAGNOSIS S, P34

CONNOLLY BH, 1986, PHYS THER, V66, P344, DOI 10.1093/ptj/66.3.344

Costanzo F, 2013, RES DEV DISABIL, V34, P1770, DOI 10.1016/j.ridd.2013.01.024

de Campos AC, 2012, RES DEV DISABIL, V33, P2228, DOI 10.1016/j.ridd.2012.06.016

de Graaf G, 2011, J APPL RES INTELLECT, V24, P247, DOI 10.1111/j.1468-3148.2010.00593.x

Diamond A, 2000, CHILD DEV, V71, P44, DOI 10.1111/1467-8624.00117

Diamond A, 2013, ANNU REV PSYCHOL, V64, P135, DOI 10.1146/annurev-psych-113011-143750

Dierssen M, 2012, NAT REV NEUROSCI, V13, P844, DOI 10.1038/nrn3314

Espy K., 2004, CLIN NEUROPSYCHOL, V18, P1

Evaggelinou C, 2002, ADAPT PHYS ACT Q, V19, P483, DOI 10.1123/apaq.19.4.483

Friedman NP, 2008, J EXP PSYCHOL GEN, V137, P201, DOI 10.1037/0096-3445.137.2.201

Hartman E, 2010, J INTELL DISABIL RES, V54, P468, DOI 10.1111/j.1365-2788.2010.01284.x

Hasan H., 2012, HUM SCI ENG RES SHUS, P217, DOI [10.1109/SHUSER.2012.6268854, DOI 10.1109/SHUSER.2012.6268854]

Henderson S., 2007, MOVEMENT ASSESSMENT

Hickey Fran, 2012, Adv Pediatr, V59, P137, DOI 10.1016/j.yapd.2012.04.006

Horvat M, 2013, RES DEV DISABIL, V34, P3517, DOI 10.1016/j.ridd.2013.06.038

Jobling A, 1998, INT J DISABIL DEV ED, V45, P283, DOI DOI 10.1080/1034912980450304

Kennedy J, 2013, SCAND J OCCUP THER, V20, P45, DOI 10.3109/11038128.2012.693944

Kent RD, 2013, J SPEECH LANG HEAR R, V56, P178, DOI 10.1044/1092-4388(2012/12-0148)

Kourtessis K., 2003, EUR J PHYS ED, V8, P202, DOI 10.1080/1740898030080207

Koziol LF, 2014, CEREBELLUM, V13, P151, DOI 10.1007/s12311-013-0511-x

Koziol LF, 2013, APPL NEUROPSYCH-CHIL, V2, P104, DOI 10.1080/21622965.2013.748386

Koziol LF, 2012, CEREBELLUM, V11, P505, DOI 10.1007/s12311-011-0321-y

Lanfranchi S, 2010, J INTELL DISABIL RES, V54, P308, DOI 10.1111/j.1365-2788.2010.01262.x

Lanfranchi S, 2009, CHILD NEUROPSYCHOL, V15, P397, DOI 10.1080/09297040902740652

Loane M, 2013, EUR J HUM GENET, V21, P27, DOI 10.1038/ejhg.2012.94

Lott IT, 2010, LANCET NEUROL, V9, P623, DOI 10.1016/S1474-4422(10)70112-5

Miyake A, 2012, CURR DIR PSYCHOL SCI, V21, P8, DOI 10.1177/0963721411429458

Moreno S, 2011, PSYCHOL SCI, V22, P1425, DOI 10.1177/0956797611416999

Okely AD, 2004, RES Q EXERCISE SPORT, V75, P238, DOI 10.1080/02701367.2004.10609157

Palisano RJ, 2001, ARCH PHYS MED REHAB, V82, P494, DOI 10.1053/apmr.2001.21956

Patterson T, 2013, J INTELL DISABIL RES, V57, P306, DOI [10.1111/j.1365-2788.2012.01536.x, 10.1111/jir.12037]

Piek JP, 2004, ARCH CLIN NEUROPSYCH, V19, P1063, DOI 10.1016/j.acn.2003.12.007

Pitetti K, 2013, J SPORT HEALTH SCI, V2, P47, DOI 10.1016/j.jshs.2012.10.004

Preacher K. J., 2002, CALCULATION TEST DIF

Presson AP, 2013, J PEDIATR-US, V163, P1163, DOI 10.1016/j.jpeds.2013.06.013

Reinert KRS, 2013, J OBES, V2013, DOI 10.1155/2013/820956

Reitan RM, 1992, NEUROPSYCHOLOGICAL E

Rigoli D, 2012, DEV MED CHILD NEUROL, V54, P1025, DOI 10.1111/j.1469-8749.2012.04403.x

Rowe J, 2006, BRIT J CLIN PSYCHOL, V45, P5, DOI 10.1348/014466505X29594

Schott N, 2014, RES DEV DISABIL, V35, P3299, DOI 10.1016/j.ridd.2014.08.001

Silverman W, 2007, MENT RETARD DEV D R, V13, P228, DOI 10.1002/mrdd.20156

Simons J, 2008, ADAPT PHYS ACT Q, V25, P71, DOI 10.1123/apaq.25.1.71

Ulrich D.A, 2000, TEST GROSS MOTOR DEV, V2nd ed

van Gameren-Oosterom HBM, 2012, PEDIATRICS, V130, pE1520, DOI 10.1542/peds.2012-0886

Van Gamereri-Oosterom HBM, 2013, RES DEV DISABIL, V34, P4599, DOI 10.1016/j.ridd.2013.09.041

Volman MJM, 2007, DISABIL REHABIL, V29, P25, DOI 10.1080/09638280600947617

Wassenberg R, 2005, CHILD DEV, V76, P1092, DOI 10.1111/j.1467-8624.2005.00899.x

Wasserman T, 2013, APPL NEUROPSYCH-CHIL, V2, P88, DOI 10.1080/21622965.2013.748394

Westendorp M, 2011, RES DEV DISABIL, V32, P2773, DOI 10.1016/j.ridd.2011.05.032

Wu JH, 2013, EUR J HUM GENET, V21, P1016, DOI 10.1038/ejhg.2012.294

Zigman WB, 2007, MENT RETARD DEV D R, V13, P237, DOI 10.1002/mrdd.20163

NR 60

TC 44

Z9 50

U1 2

U2 93

PU WILEY

PI HOBOKEN

PA 111 RIVER ST, HOBOKEN 07030-5774, NJ USA

SN 0964-2633

EI 1365-2788

J9 J INTELL DISABIL RES

JI J. Intell. Disabil. Res.

PD SEP

PY 2015

VL 59

IS 9

BP 860

EP 872

DI 10.1111/jir.12189

PG 13

WC Education, Special; Genetics & Heredity; Clinical Neurology; Psychiatry;

Rehabilitation

WE Social Science Citation Index (SSCI)

SC Education & Educational Research; Genetics & Heredity; Neurosciences &

Neurology; Psychiatry; Rehabilitation

GA CP9KS

UT WOS:000360213600043

PM 25688672

DA 2022-08-17

ER

PT J

AU Fenollar-Cortes, J

Gallego-Martinez, A

Fuentes, LJ

AF Fenollar-Cortes, Javier

Gallego-Martinez, Ana

Fuentes, Luis J.

TI The role of inattention and hyperactivity/impulsivity in the fine motor

coordination in children with ADHD

SO RESEARCH IN DEVELOPMENTAL DISABILITIES

LA English

DT Article

DE Attention-deficit/hyperactivity disorder; Fine motor coordination;

Handwriting; Graphomotor skills; Non-pharmacological interventions

ID ATTENTION-DEFICIT/HYPERACTIVITY DISORDER; ELEMENTARY-SCHOOL;

PERFORMANCE; SKILLS; COMORBIDITY; ADOLESCENTS

AB Objective: Deficits in fine motor coordination have been suggested to be associated with Attention-Deficit/Hyperactivity Disorder (ADHD). However, despite the negative impact of poor fine motor skills on academic achievement, researchers have paid little attention to this problem. The aim of this study was to explore the relationship between ADHD dimensions and fine motor performance.

Method: Participants were 43 children with a diagnosis of ADHD aged between 7 and 14 years (M = 9.61; 81% male) and 42 typically developing (TP) children in the same age range (M = 10.76; 75.2% male).

Results: Children with ADHD performed worse than TP on all tasks (delta(Fine_motor_tasks,) -0.19 to -0.44). After controlling for age and ADHD-HY (hyperactivity/impulsivity), higher scores on ADHD-IN (inattentiveness) predicted a larger number of mistakes among all psychomotricity tasks and conditions (beta 0.39-0.58, ps < 0.05).

Conclusion: The ADHD group showed poorer fine motor performance than controls across all fine motor coordination tasks. However, lower performance (more mistakes), was related to the inattention dimension but not to the hyperactivity/impulsivity dimensions. Authors recommend including training and enhancement of the fine motor skills for more comprehensive ADHD treatment.

C1 [Fenollar-Cortes, Javier] Univ Murcia, Dept Psicol Evolut & Educ, Murcia, Spain.

[Gallego-Martinez, Ana] Ctr Desarrollo Infantil & Atenc Temprana HIGEA, Murcia, Spain.

[Fuentes, Luis J.] Univ Murcia, Dept Psicol Basica & Metodol, Murcia, Spain.

RP Fenollar-Cortes, J (通讯作者)，Univ Murcia, Dept Psicol Evolut & Educ, Fac Psicol, Campus Espinardo, E-30100 Murcia, Spain.

EM javier.fenollar@um.es

RI Fenollar-Cortés, Javier/C-6636-2019

OI Fenollar-Cortés, Javier/0000-0001-6154-8836; Gallego,

Ana/0000-0002-7302-7613

FU Spanish Ministry of Economy and Competitiveness [PSI2014-53427-P];

Fundacion Seneca [19267/PI/14]

FX This study was supported by the Spanish Ministry of Economy and

Competitiveness (project PSI2014-53427-P) and Fundacion Seneca (project

19267/PI/14).

CR Arnold L., 2015, J ATTENTION DISORDER

Barkley RA, 2015, ATTENTION-DEFICIT HYPERACTIVITY DISORDER IN ADULTS AND CHILDREN, P53

Bart O, 2010, RES DEV DISABIL, V31, P1443, DOI 10.1016/j.ridd.2010.06.014

Berninger VW, 2006, J SCHOOL PSYCHOL, V44, P3, DOI 10.1016/j.jsp.2005.12.003

Cohen J., 1988, STAT POWER ANAL BEHA

Czamara D, 2013, PLOS ONE, V8, DOI 10.1371/journal.pone.0063859

Dinehart L, 2013, EARLY EDUC DEV, V24, P138, DOI 10.1080/10409289.2011.636729

Dinehart LH, 2015, J EARLY CHILD LIT, V15, P97, DOI 10.1177/1468798414522825

DuPaul G.J., 1998, ADHD RATING SCALE 4

DuPaul GJ, 2016, PSYCHOL ASSESSMENT, V28, P214, DOI 10.1037/pas0000166

DuPaul GJ, 2014, CHILD ADOL PSYCH CL, V23, P687, DOI 10.1016/j.chc.2014.05.003

Feder KP, 2007, DEV MED CHILD NEUROL, V49, P312, DOI 10.1111/j.1469-8749.2007.00312.x

Fenollar-Cortes J, 2016, FRONT PSYCHOL, V7, DOI 10.3389/fpsyg.2016.00871

Fliers E, 2008, J NEURAL TRANSM, V115, P211, DOI 10.1007/s00702-007-0827-0

Fliers EA, 2010, CHILD ADOL MENT H-UK, V15, P85, DOI 10.1111/j.1475-3588.2009.00538.x

Goulardins JB, 2013, RES DEV DISABIL, V34, P40, DOI 10.1016/j.ridd.2012.07.014

Greifeneder R, 2010, SOC PSYCHOL PERS SCI, V1, P230, DOI 10.1177/1948550610368434

Grissmer D, 2010, DEV PSYCHOL, V46, P1008, DOI 10.1037/a0020104

Langmaid RA, 2016, J ATTEN DISORD, V20, P131, DOI 10.1177/1087054713493317

Luo Z, 2007, BRIT J DEV PSYCHOL, V25, P595, DOI 10.1348/026151007X185329

Martinussen R, 2005, J AM ACAD CHILD PSY, V44, P377, DOI 10.1097/01.chi.0000153228.72591.73

Mayes SD, 2007, CHILD NEUROPSYCHOL, V13, P469, DOI 10.1080/09297040601112773

MCHALE K, 1992, AM J OCCUP THER, V46, P898, DOI 10.5014/ajot.46.10.898

Molitor SJ, 2016, RES DEV DISABIL, V51-52, P49, DOI 10.1016/j.ridd.2016.01.005

Noda W, 2013, RES DEV DISABIL, V34, P2909, DOI 10.1016/j.ridd.2013.05.023

Pitchford NJ, 2016, FRONT PSYCHOL, V7, DOI 10.3389/fpsyg.2016.00783

Racine MB, 2008, J CHILD NEUROL, V23, P399, DOI 10.1177/0883073807309244

RESTA SP, 1994, PERCEPT MOTOR SKILL, V79, P1131, DOI 10.2466/pms.1994.79.3.1131

Romano J., 2006, ANN M SO ASS I RES C, V13, P1

Rosenblum S., 2009, PHYS OCCUPATIONAL TH, V28, P219

Sayal K, 2015, J AM ACAD CHILD PSY, V54, P360, DOI 10.1016/j.jaac.2015.02.007

Servera & Cardo, 2007, REV NEUROLOGIA, V45, P393

Shen IH, 2012, RES DEV DISABIL, V33, P1301, DOI 10.1016/j.ridd.2012.02.010

Son S., 2006, MERRILL PALMER Q, V52

Tucha O, 2001, J ABNORM CHILD PSYCH, V29, P351, DOI 10.1023/A:1010366014095

Wahlstedt C, 2009, J ABNORM CHILD PSYCH, V37, P551, DOI 10.1007/s10802-008-9286-9

Willcutt EG, 2005, BIOL PSYCHIAT, V57, P1336, DOI 10.1016/j.biopsych.2005.02.006

Willcutt EG, 2012, J ABNORM PSYCHOL, V121, P991, DOI 10.1037/a0027347

NR 38

TC 22

Z9 22

U1 0

U2 38

PU PERGAMON-ELSEVIER SCIENCE LTD

PI OXFORD

PA THE BOULEVARD, LANGFORD LANE, KIDLINGTON, OXFORD OX5 1GB, ENGLAND

SN 0891-4222

EI 1873-3379

J9 RES DEV DISABIL

JI Res. Dev. Disabil.

PD OCT

PY 2017

VL 69

BP 77

EP 84

DI 10.1016/j.ridd.2017.08.003

PG 8

WC Education, Special; Rehabilitation

WE Social Science Citation Index (SSCI)

SC Education & Educational Research; Rehabilitation

GA FH9MQ

UT WOS:000411536500008

PM 28829996

DA 2022-08-17

ER

PT J

AU Sumner, E

Pratt, ML

Hill, EL

AF Sumner, Emma

Pratt, Michelle L.

Hill, Elisabeth L.

TI Examining the cognitive profile of children with Developmental

Coordination Disorder

SO RESEARCH IN DEVELOPMENTAL DISABILITIES

LA English

DT Article

DE Cognitive ability; Developmental Coordination Disorder (DCD); Diagnosis;

Intelligence; Motor impairment

ID HIGH-FUNCTIONING AUTISM; WISC-IV; PROCESSING SPEED; WORKING-MEMORY;

ATTENTION; SKILLS; DCD

AB Background: While primarily a motor disorder, research considering the cognitive abilities in children with Developmental Coordination Disorder (DCD) is limited; even though these children often struggle academically.

Aims: The present study aimed to characterise the IQ profile of children with and without DCD, and to identify whether children with DCD exhibit specific cognitive weaknesses.

Methods and procedures: 104 children participated in the study. Fifty-two children (mean age, 9 years) with a diagnosis of DCD were matched to 52 typically-developing children by age and gender. Cognitive ability was assessed using the Wechsler Intelligence Scale for Children (WISC-IV).

Outcomes and results: Children with DCD performed poorer than their peers on processing speed and working memory measures. Individual analyses revealed varied performance in the DCD group across all cognitive indices, despite displaying Full-Scale IQs in the typical range. Discriminant function analyses show processing speed and working memory performance predicted only 23% of between-group variability.

Conclusions: Children with DCD present with a heterogeneous cognitive profile, lending support to individual case analyses in research and when designing educational assistance plans. The motorically-demanding nature of the WISC-IV processing speed tasks raises specific concerns about using this index of the IQ assessment in this population. Research and practical implications are raised. (C) 2016 Elsevier Ltd. All rights reserved.

C1 [Sumner, Emma; Hill, Elisabeth L.] Goldsmiths Univ London, Dept Psychol, London SE14 6NW, England.

[Pratt, Michelle L.] Chelsea & Westminister Hosp, Psychol Med Unit, London, England.

RP Hill, EL (通讯作者)，Goldsmiths Univ London, Dept Psychol, London SE14 6NW, England.

EM e.hill@gold.ac.uk

RI VARRECCHIA, TIWANA/AAJ-8712-2021

OI Sumner, Emma/0000-0002-9196-7713; Hill, Elisabeth/0000-0003-3130-1271

FU ESRC; Leverhulme Trust [RPG-2012-742]

FX This research was jointly facilitated by an ESRC PhD studentship awarded

to the second author and a Leverhulme Trust grant (RPG-2012-742) awarded

to the final author. Special thanks go to all the children, parents, and

schools that participated in the work described here. Thanks are also

due to Dr Laura Crane for providing comments on the first draft of the

manuscript.

CR Alloway TP, 2007, J EXP CHILD PSYCHOL, V96, P20, DOI 10.1016/j.jecp.2006.07.002

American Psychiatric Association, 2013, DIAGN STAT MAN MENT, V5th edition, DOI [10.1176/appi.books.9780890425596, DOI 10.1176/APPI.BOOKS.9780890425596]

Anderson VA, 2001, DEV NEUROPSYCHOL, V20, P385, DOI 10.1207/S15326942DN2001_5

Blank R, 2012, DEV MED CHILD NEUROL, V54, P54, DOI 10.1111/j.1469-8749.2011.04171.x

Castles A, 2014, PHILOS T R SOC B, V369, DOI 10.1098/rstb.2013.0407

Charman T, 2011, PSYCHOL MED, V41, P619, DOI 10.1017/S0033291710000991

Dewey D, 2002, HUM MOVEMENT SCI, V21, P905, DOI 10.1016/S0167-9457(02)00163-X

Geuze Reint H, 2015, Curr Dev Disord Rep, V2, P127

Gomez A, 2015, RES DEV DISABIL, V43-44, P167, DOI 10.1016/j.ridd.2015.06.011

Grant D., 2010, WAY I THINK DYSLEXIA

Hagberg BS, 2010, RES DEV DISABIL, V31, P1, DOI 10.1016/j.ridd.2009.07.012

Henderson S., 2007, MOVEMENT ASSESSMENT

Kaplan BJ, 1998, HUM MOVEMENT SCI, V17, P471, DOI 10.1016/S0167-9457(98)00010-4

Kirby A, 2007, J ROY SOC MED, V100, P182, DOI 10.1258/jrsm.100.4.182

Leonard H. C., 2015, CURR DEV DISORD REP, V2, P141, DOI [10.1007/s40474-015-0044-8, DOI 10.1007/S40474-015-0044-8]

Lingam R, 2009, PEDIATRICS, V123, pE693, DOI 10.1542/peds.2008-1770

Loh PR, 2011, RES DEV DISABIL, V32, P1260, DOI 10.1016/j.ridd.2011.02.008

Mayes SD, 2008, J AUTISM DEV DISORD, V38, P428, DOI 10.1007/s10803-007-0410-4

Moura O, 2014, DYSLEXIA, V20, P19, DOI 10.1002/dys.1468

Oliveras-Rentas RE, 2012, J AUTISM DEV DISORD, V42, P655, DOI 10.1007/s10803-011-1289-7

Parke E. M., 2015, J ATTENTION DISORDER, V1

Pfeiffer SI, 2000, SCHOOL PSYCHOL QUART, V15, P376, DOI 10.1037/h0088795

Piaget J., 1952, ORIGIN INTELLIGENCE, DOI [10.1037/11494-000, DOI 10.1037/11494-000]

Piek JP, 2007, DEV MED CHILD NEUROL, V49, P678, DOI 10.1111/j.1469-8749.2007.00678.x

Querne L, 2008, BRAIN RES, V1244, P89, DOI 10.1016/j.brainres.2008.07.066

Smits-Engelsman B. C. M., 2012, PEDIATRICS, V130, P2011

Smits-Engelsman B, 2015, HUM MOVEMENT SCI, V42, P293, DOI 10.1016/j.humov.2015.03.010

van der Fels IMJ, 2015, J SCI MED SPORT, V18, P697, DOI 10.1016/j.jsams.2014.09.007

WECHSLER David, 2003, WECHSLER INTELLIGENC, V4

Wilson PH, 1998, J CHILD PSYCHOL PSYC, V39, P829, DOI 10.1111/1469-7610.00384

NR 30

TC 21

Z9 24

U1 5

U2 45

PU PERGAMON-ELSEVIER SCIENCE LTD

PI OXFORD

PA THE BOULEVARD, LANGFORD LANE, KIDLINGTON, OXFORD OX5 1GB, ENGLAND

SN 0891-4222

J9 RES DEV DISABIL

JI Res. Dev. Disabil.

PD SEP

PY 2016

VL 56

BP 10

EP 17

DI 10.1016/j.ridd.2016.05.012

PG 8

WC Education, Special; Rehabilitation

WE Social Science Citation Index (SSCI)

SC Education & Educational Research; Rehabilitation

GA DQ7EP

UT WOS:000379370000002

PM 27258924

OA Green Accepted, Green Submitted

DA 2022-08-17

ER

PT J

AU Jokic, CAS

Whitebread, D

AF Jokic, Claire A. Sangster

Whitebread, David

TI Self-Regulatory Skill Among Children with and without Developmental

Coordination Disorder: An Exploratory Study

SO PHYSICAL & OCCUPATIONAL THERAPY IN PEDIATRICS

LA English

DT Article

DE Metacognition; motor learning; developmental coordination disorder;

self-regulation

ID METACOGNITION; EMULATION; DCD

AB Aim: Children with developmental coordination disorder (DCD) experience difficulty learning and performing everyday motor tasks due to poor motor coordination. Recent research applying a cognitive learning paradigm has argued that children with DCD have less effective cognitive and metacognitive skills with which to effectively acquire motor skills. However, there is currently limited research examining individual differences in children's use of self-regulatory and metacognitive skill during motor learning. This exploratory study aimed to compare the self-regulatory performance of children with and without DCD. Methods: Using a mixed methods approach, this study observed and compared the self-regulatory behavior of 15 children with and without DCD, aged between 7 and 9years, during socially mediated motor practice. Observation was conducted using a quantitative coding scheme and qualitative analysis of video-recorded sessions. This paper will focus on the results of quantitative analysis, while data arising from the qualitative analysis will be used to support quantitative findings. Results: In general, findings indicate that children with DCD exhibit less independent and more ineffective self-regulatory skill during motor learning than their typically developing peers. In addition, children with DCD rely more heavily on external support for effective regulation and are more likely to exhibit negative patterns of motivational regulation. Conclusions: These findings provide further support for the notion that children with DCD experience difficulty effectively self-regulating motor learning. Implications for practice and directions for future research are discussed.

C1 [Jokic, Claire A. Sangster; Whitebread, David] Univ Cambridge, Fac Educ, Cambridge, England.

RP Jokic, CAS (通讯作者)，Stipanciceva 21, Zagreb 10000, Croatia.

EM sangsterjokic@gmail.com

RI Jokić, Claire Sangster/ABD-9300-2021

OI Jokić, Claire Sangster/0000-0003-0306-9815

FU Cambridge Commonwealth Trusts in completing these studies

FX The author acknowledges the financial support provided by the Cambridge

Commonwealth Trusts in completing these studies.

CR *AM PSYCH ASS, 2000, DSM 4 TR DIAGN STAT, P53

Bakeman R., 1997, OBSERVING INTERACTIO, V2nd

Boekaerts M., 1999, INT J EDUC RES, V31, P445, DOI DOI 10.1016/S0883-0355(99)00014-2

BORKOWSKI JG, 1989, LEARN DISABILITY Q, V12, P57, DOI 10.2307/1510252

Butler DL, 2002, EDUC PSYCHOL-US, V37, P59, DOI 10.1207/00461520252828564

CHAMBERS ME, 2005, CHILDREN DEV COORDIN, P1

Cleary TJ, 2001, J APPL SPORT PSYCHOL, V13, P185, DOI 10.1080/104132001753149883

Cousins M, 2003, HUM MOVEMENT SCI, V22, P433, DOI 10.1016/j.humov.2003.09.003

Davidson JE, 1998, EDUC PSYCHO, P47

Ferrari M, 1996, DEV REV, V16, P203, DOI 10.1006/drev.1996.0008

HENDERSON SE, 1992, MOVEMENT ASSESSMENT

Jokic CS, 2013, ADAPT PHYS ACT Q, V30, P103, DOI 10.1123/apaq.30.2.103

Kitsantas A, 2000, J EDUC PSYCHOL, V92, P811, DOI 10.1037/0022-0663.92.4.811

Kitsantas A, 2002, J APPL SPORT PSYCHOL, V14, P91, DOI 10.1080/10413200252907761

Kolovelonis A, 2010, J APPL SPORT PSYCHOL, V22, P198, DOI 10.1080/10413201003664681

Lloyd M, 2006, ADAPT PHYS ACT Q, V23, P370, DOI 10.1123/apaq.23.4.370

Mandich A., 1997, THESIS

Mandich AD, 2004, PAEDIAT ACTIVITY CAR

Mandich Angela D., 2001, Physical and Occupational Therapy in Pediatrics, V20, P125, DOI 10.1300/J006v20n02_08

Martini R, 2004, ADAPT PHYS ACT Q, V21, P248, DOI 10.1123/apaq.21.3.248

Martini R, 2008, LEARN INDIVID DIFFER, V18, P237, DOI 10.1016/j.lindif.2007.08.004

Missiuna C., 1999, KEEPING CURRENT, V99, P1

Missiuna C., 1997, COGNITIVE TOP DOWN A

NEWELL KM, 1982, DEV MOVEMENT CONTROL, P175

Noldus Information Technology, 2008, THE OBS XT

Paris S.G., 1990, DIMENSIONS THINKING, P15

Perry NE, 2002, EDUC PSYCHOL, V37, P5, DOI 10.1207/S15326985EP3701_2

Pino Pasternak D, 2008, THESIS

Pintrich PR., 2000, HDB SELF REGULATION, P451, DOI [10.1016/B978-012109890-2/50043-3, DOI 10.1016/B978-012109890-2/50043-3]

Polatajko H., 2004, ENABLING OCCUPATION

Polatajko HJ, 1999, CLIN DEV MED, P119

Sangster C.A., 2005, THESIS

Sangster Claire A, 2005, Can J Occup Ther, V72, P67

Schunk DH, 2001, SELF-REGULATED LEARNING AND ACADEMIC ACHIEVEMENT, SECOND ED., P125

Sugden D A, 2006, COMMUNICATION

Sugden D. A., 1998, MOTOR COORDINATION D

Sugden D, 2007, DEV MED CHILD NEUROL, V49, P467, DOI 10.1111/j.1469-8749.2007.00467.x

Sugden D, 2008, INT J DISABIL DEV ED, V55, P173, DOI 10.1080/10349120802033691

Wall AE, 1985, ADAPTED PHYSICAL ACT, V2, P21

Whitebread D, 2007, J COGN EDUC PSYCHOL, V6, P433, DOI 10.1891/194589507787382043

Wilson PH, 2005, J CHILD PSYCHOL PSYC, V46, P806, DOI 10.1111/j.1469-7610.2005.01409.x

Winne P. H., 2000, HDB SELF REGULATION, P531, DOI [DOI 10.1016/B978-012109890-2/50045-7, 10.1016/B978-012109890-2/50045-7]

WONG BYL, 1985, METACOGNITION COGNIT, V2, P137

Zimmerman B.J., 2000, HDB SELF REGULATION, P13, DOI [10.1016/b978-012109890-2/ 50031-7, https://doi.org/10.1016/B978-012109890-2/50031-7]

Zimmerman BJ, 2006, CAMBRIDGE HANDBOOK OF EXPERTISE AND EXPERT PERFORMANCE, P705

Zimmerman BJ, 1997, J EDUC PSYCHOL, V89, P29, DOI 10.1037/0022-0663.89.1.29

Zimmerman BJ, 2002, THEOR PRACT, V41, P64, DOI 10.1207/s15430421tip4102_2

[No title captured]

NR 48

TC 4

Z9 4

U1 0

U2 10

PU TAYLOR & FRANCIS INC

PI PHILADELPHIA

PA 530 WALNUT STREET, STE 850, PHILADELPHIA, PA 19106 USA

SN 0194-2638

EI 1541-3144

J9 PHYS OCCUP THER PEDI

JI Phys. Occup. Ther. Pediatr.

PD NOV

PY 2016

VL 36

IS 4

BP 401

EP 421

DI 10.3109/01942638.2015.1135844

PG 21

WC Pediatrics; Rehabilitation

WE Science Citation Index Expanded (SCI-EXPANDED); Social Science Citation Index (SSCI)

SC Pediatrics; Rehabilitation

GA DZ0LK

UT WOS:000385530400006

PM 26939836

DA 2022-08-17

ER

PT J

AU Salawu, S

Lyoka, P

Goon, DI

AF Salawu, Seyide

Lyoka, Philemon

Goon, Daniel, I

TI Physical motor competences of severely mentally challenged children in

King William's Town, South Africa

SO MEDICINA DELLO SPORT

LA English

DT Article

DE Motor skills; Child development disorders, pervasive; Disabled Children

ID MILD INTELLECTUAL DISABILITY; RETARDATION; PERFORMANCE; TGMD-2

AB BACKGROUND: The purpose of the study was to assess the levels of physical motor competences among severely mentally challenged children (SMCC); and identify the physical motor difficulties they experienced.

METHODS: Participants were 25 SMCC (18 boys and 7 girls) aged 6-10 years, attending at King William Special School, East London, South Africa. The TGMD-2 Test battery was utilized to assess motor competency. Five locomotor subtests (run, hop, leap, horizontal jump and slide); and six object control subtests (striking stationary ball, stationary dribble, catch, kick, overhand throw and underhand roll) were measured.

RESULTS: The gross motor quotient were very poor and poor, respectively (ranging from GMQ<70 and 70-79). About 96% of the children had their gross motor competence below average. Generally, GMQ were below 70 in gross motor abilities. The object control competences of SMCC indicates that the highest score was in striking (10), stationary dribbling (8), kicking (8), overhand throw (8) underhand rolls (8) and catching (6). Overall, SMCC were competent in upper body coordination, especially in catching. The gross motor difficulties in the locomotor subtests among the SMCC were: running (superior category=38%; average performance=29%; very poor and poor=33%); hopping (very superior (38%) and superior (10%) performances. Performance was low (45%) among the rated very superior (18), superior (27) in horizontal jumping activity. The results from sliding ability was encouraging as SMCC had 57% performance score, obtained from very superior (22%), superior (35%) scores. The best cumulative locomotor performance was 62% in hop activity, followed by 57% in slide activity. Apart from the 54% in horizontal jump, SMCC scored below 50% in running. The performance score in striking was 40% (very superior - 12 and superior - 28). Critical challenges were observed in stationary dribbling, as only few had superior performance (12%), average (12%), and poor/very poor (76%). About 64% performed average and poor/very poor (36%) in catching activity; kicking (58% very superior). Majority of the SMCC were competent (59%), performed very superiorly (27) and superior (32) in overhead throws. Majority (60%) performed well in underhand rolling. None of the SMCC scored 20%, except, in kicking the ball (23%), while stationary dribble recorded the lowest (4%).

CONCLUSIONS: The highest motor competence of SMCC was above average in object control ability, kicking while locomotor ability, leaping was poor. The SMCC were competent in object control abilities. Hence, these results have implications for teaching and supporting children with mental disabilities.

C1 [Salawu, Seyide; Lyoka, Philemon] Univ Ft Hare, Fac Hlth Sci, Dept Human Movement, Alice, South Africa.

[Goon, Daniel, I] Univ Ft Hare, Fac Hlth Sci, Dept Nursing Sci, East London, South Africa.

RP Goon, DI (通讯作者)，Univ Ft Hare, Fac Hlth Sci, East London Campus,5 Oxford St, ZA-5201 East London, South Africa.

EM dgoon@ufh.ac.za

RI Goon, Daniel/C-2912-2019

OI Goon, Daniel/0000-0001-9070-7875

CR Ahmadi R, 2012, INT J SPORT STUDIES, V2, P496

American Association on Intellectual and Developmental Disabilities (AAIDD), 2007, DEF MENT RET

Carmeli E, 2008, DISABIL REHABIL, V30, P323, DOI 10.1080/09638280701265398

Cooper RA, 1999, J REHABIL RES DEV, V36, P1

Gallahue D.L., 2007, DEV PHYS ED ALL CHIL

Gallahue D.L., 2006, UNDERSTANDING MOTOR

Gentry D, 1977, ED SEVERELY PROFOUND

Hartman E, 2010, J INTELL DISABIL RES, V54, P468, DOI 10.1111/j.1365-2788.2010.01284.x

Heward WL, 2014, CHARACTERISTICS CHIL

Houwen S, 2010, ADAPT PHYS ACT Q, V27, P143, DOI 10.1123/apaq.27.2.143

Kangas S., 2012, INT J PLAY, V1, P37, DOI [10.1080/21594937.2012.656920, DOI 10.1080/21594937.2012.656920]

Kim Y, 2012, ADAPT PHYS ACT Q, V29, P346, DOI 10.1123/apaq.29.4.346

LACY AC, 2007, MEASUREMENT EVALUATI

Sadrossadat L, 2010, INT J PREVENTIVE MED, V1, P34

Sanghavi R., 2005, INDIAN J OCCUP THER, VXXXVII, P33, DOI DOI 10.1016/J.SBSPRO.2014.01.1201

Spano M, 1999, Eur J Paediatr Neurol, V3, P7, DOI 10.1053/ejpn.1999.0173

Stodden DF, 2008, QUEST, V60, P290, DOI 10.1080/00336297.2008.10483582

Tomporowski PD, 2011, PREV MED, V52, pS3, DOI 10.1016/j.ypmed.2011.01.028

Ulrich D.A, 2000, TEST GROSS MOTOR DEV, V2nd ed

Virji-Babul Naznin, 2006, Downs Syndr Res Pract, V10, P74, DOI 10.3104/reports.308

Vuijk PJ, 2010, J INTELL DISABIL RES, V54, P955, DOI 10.1111/j.1365-2788.2010.01318.x

Wehmeyer ML, 2003, EDUC TRAIN DEV DISAB, V38, P271

Wuest AD, 2009, FDN PHYS ED EXERCISE

Yukselen A, 2008, MIDDLE E J FAM MED, V6, P2

Yun J, 2004, ADAPT PHYS ACT Q, V21, P269, DOI 10.1123/apaq.21.3.269

NR 25

TC 0

Z9 0

U1 0

U2 8

PU EDIZIONI MINERVA MEDICA

PI TURIN

PA CORSO BRAMANTE 83-85 INT JOURNALS DEPT., 10126 TURIN, ITALY

SN 0025-7826

EI 1827-1863

J9 MED SPORT

JI Med. Sport

PD DEC

PY 2017

VL 70

IS 4

BP 521

EP 536

DI 10.23736/S0025-7826.17.02962-3

PG 16

WC Medicine, General & Internal; Sport Sciences

WE Science Citation Index Expanded (SCI-EXPANDED); Social Science Citation Index (SSCI)

SC General & Internal Medicine; Sport Sciences

GA GU3RK

UT WOS:000445196900012

DA 2022-08-17

ER

PT J

AU Fong, SSM

Chung, JWY

Cheng, YTY

Yam, TTT

Chiu, HC

Fong, DYT

Cheung, CY

Yuen, L

Yu, EYT

Hung, YS

Macfarlane, DJ

Ng, SSM

AF Fong, Shirley S. M.

Chung, Joanne W. Y.

Cheng, Yoyo T. Y.

Yam, Timothy T. T.

Chiu, Hsiu-Ching

Fong, Daniel Y. T.

Cheung, C. Y.

Yuen, Lily

Yu, Esther Y. T.

Hung, Yeung Sam

Macfarlane, Duncan J.

Ng, Shamay S. M.

TI Attention during functional tasks is associated with motor performance

in children with developmental coordination disorder: A cross-sectional

study

SO MEDICINE

LA English

DT Article

DE children; mental concentration; motor difficulty; rehabilitation

ID DEFICIT-HYPERACTIVITY DISORDER; SENSORY ORGANIZATION; PSYCHOSOCIAL

ADJUSTMENT; POSTURAL CONTROL; BALANCE CONTROL; CORTEX; EXERCISE; SKILLS;

FORCE; AREAS

AB This cross-sectional and exploratory study aimed to compare motor performance and electroencephalographic (EEG) attention levels in children with developmental coordination disorder (DCD) and those with typical development, and determine the relationship between motor performance and the real-time EEG attention level in children with DCD.Eighty-six children with DCD [DCD: n=57; DCD and attention deficit hyperactivity disorder (ADHD): n=29] and 99 children with typical development were recruited. Their motor performance was assessed with the Movement Assessment Battery for Children (MABC) and attention during the tasks of the MABC was evaluated by EEG.All children with DCD had higher MABC impairment scores and lower EEG attention scores than their peers (P<0.05). After accounting for age, sex, body mass index, and physical activity level, the attention index remained significantly associated with the MABC total impairment score and explained 14.1% of the variance in children who had DCD but not ADHD (P=0.009) and 17.5% of the variance in children with both DCD and ADHD (P=0.007). Children with DCD had poorer motor performance and were less attentive to movements than their peers. Their poor motor performance may be explained by inattention.

C1 [Fong, Shirley S. M.; Cheng, Yoyo T. Y.; Yam, Timothy T. T.] Univ Hong Kong, Sch Publ Hlth, Pokfulam, Hong Kong, Peoples R China.

[Chung, Joanne W. Y.] Educ Univ Hong Kong, Dept Hlth & Phys Educ, Tai Po, Hong Kong, Peoples R China.

[Chiu, Hsiu-Ching] I Shou Univ, Dept Phys Therapy, Kaohsiung, Taiwan.

[Fong, Daniel Y. T.] Univ Hong Kong, Sch Nursing, Pokfulam, Hong Kong, Peoples R China.

[Cheung, C. Y.] Univ Hong Kong, Dept Social Work & Social Adm, Pokfulam, Hong Kong, Peoples R China.

[Yuen, Lily] Heep Hong Soc, Ngau Tau Kok, Hong Kong, Peoples R China.

[Yu, Esther Y. T.] Univ Hong Kong, Dept Family Med & Primary Care, Pokfulam, Hong Kong, Peoples R China.

[Hung, Yeung Sam] Univ Hong Kong, Dept Elect & Elect Engn, Pokfulam, Hong Kong, Peoples R China.

[Macfarlane, Duncan J.] Univ Hong Kong, Ctr Sports & Exercise, Pokfulam, Hong Kong, Peoples R China.

[Ng, Shamay S. M.] Hong Kong Polytech Univ, Dept Rehabil Sci, Hong Kong, Hong Kong, Peoples R China.

RP Fong, SSM (通讯作者)，Univ Hong Kong, Sch Publ Hlth, Pokfulam, Hong Kong, Peoples R China.

EM smfong@hku.hk

RI Ng, Shamay/E-2731-2016; Fong, Daniel YT/C-4269-2009; Chiu,

Hsiu-Ching/AAR-7339-2021; Fong, Shirley S.M./J-4477-2012

OI Ng, Shamay/0000-0003-1660-0548; Fong, Daniel YT/0000-0001-7365-9146;

Fong, Shirley S.M./0000-0001-6410-7606; Yu, Yee Tak

Esther/0000-0001-7472-7083; Chung, Joanne/0000-0001-9884-9800

FU Research Grants Council of Hong Kong [27100614]

FX This research was partially supported by a grant (27100614) from the

Research Grants Council of Hong Kong.

CR Alesi Marianha, 2014, Muscles Ligaments Tendons J, V4, P114

Alesi M, 2016, PERCEPT MOTOR SKILL, V122, P27, DOI 10.1177/0031512515627527

Alesi M, 2015, FRONT PSYCHOL, V6, DOI 10.3389/fpsyg.2015.01627

Alesi M, 2014, NEUROPSYCH DIS TREAT, V10, P479, DOI 10.2147/NDT.S58455

American Psychiatric Association, 1994, DIAGNOSTIC STAT MANU

Barkley RA, 1990, ATTENTION DEFICIT HY, P3

Binkofski F, 2002, J NEUROPHYSIOL, V88, P514, DOI 10.1152/jn.2002.88.1.514

Bruininks R.H., 2005, BRUININKS OSERETSKY

Chaix Y, 2007, EUR J PAEDIATR NEURO, V11, P368, DOI 10.1016/j.ejpn.2007.03.006

Cherng RJ, 2009, GAIT POSTURE, V29, P204, DOI 10.1016/j.gaitpost.2008.08.003

Croce RV, 2001, PERCEPT MOTOR SKILL, V93, P275, DOI 10.2466/pms.2001.93.1.275

Dewey D, 2002, HUM MOVEMENT SCI, V21, P905, DOI 10.1016/S0167-9457(02)00163-X

FONG, 2015, J NOV PHYSIOTHER, V5

Fong SSM, 2016, SCI REP-UK, V6, DOI 10.1038/srep20945

Fong SSM, 2012, HUM MOVEMENT SCI, V31, P1317, DOI 10.1016/j.humov.2011.11.003

Fong SSM, 2011, RES DEV DISABIL, V32, P2376, DOI 10.1016/j.ridd.2011.07.025

Gabbard C, 2011, INT J NEUROSCI, V121, P113, DOI 10.3109/00207454.2010.535936

HENDERSON SE, 1992, MOVEMENT ASSESSMENT

Ivry Richard B., 2003, Neural Plasticity, V10, P141, DOI 10.1155/NP.2003.141

Johansen-Berg H, 2002, EXP BRAIN RES, V142, P13, DOI 10.1007/s00221-001-0905-8

Kristeva-Feige R, 2002, CLIN NEUROPHYSIOL, V113, P124, DOI 10.1016/S1388-2457(01)00722-2

Laufer Y, 2008, GAIT POSTURE, V27, P347, DOI 10.1016/j.gaitpost.2007.04.013

Leonard HC, 2015, DEV NEUROPSYCHOL, V40, P201, DOI 10.1080/87565641.2014.997933

Lohse KR, 2011, J MOTOR BEHAV, V43, P173, DOI 10.1080/00222895.2011.555436

Mandich Angela D., 2001, Physical and Occupational Therapy in Pediatrics, V20, P51, DOI 10.1300/J006v20n02_04

Marien P, 2010, CEREBELLUM, V9, P405, DOI 10.1007/s12311-010-0177-6

McLeod KR, 2014, NEUROIMAGE-CLIN, V4, P566, DOI 10.1016/j.nicl.2014.03.010

Muthukumaraswamy SD, 2004, CLIN NEUROPHYSIOL, V115, P1760, DOI 10.1016/j.clinph.2004.03.004

NeuroSky, 2012, MINDWAVE MOB US GUID

Pessoa L, 2003, J NEUROSCI, V23, P3990

Piek JP, 2004, HUM MOVEMENT SCI, V23, P475, DOI 10.1016/j.humov.2004.08.019

Piek JP, 2004, ARCH CLIN NEUROPSYCH, V19, P1063, DOI 10.1016/j.acn.2003.12.007

Piek JP, 1999, DEV MED CHILD NEUROL, V41, P159, DOI 10.1017/S0012162299000341

Rebolledo-Mendez G, 2009, LECT NOTES COMPUT SC, V5610, P149, DOI 10.1007/978-3-642-02574-7_17

Ridley K, 2008, INT J BEHAV NUTR PHY, V5, DOI 10.1186/1479-5868-5-45

Rowan A.J., 2003, PRIMER EEG MINIATLAS

Tseng MH, 2007, INT J REHABIL RES, V30, P327, DOI 10.1097/MRR.0b013e3282f144c7

van der Fels IMJ, 2015, J SCI MED SPORT, V18, P697, DOI 10.1016/j.jsams.2014.09.007

Visser J, 2003, HUM MOVEMENT SCI, V22, P479, DOI 10.1016/j.humov.2003.09.005

Wilmut K, 2007, DISABIL REHABIL, V29, P47, DOI 10.1080/09638280600947765

Wilson BN, 2009, PHYS OCCUP THER PEDI, V29, P182, DOI 10.1080/01942630902784761

Zwicker JG, 2009, J CHILD NEUROL, V24, P1273, DOI 10.1177/0883073809333537

NR 42

TC 11

Z9 11

U1 1

U2 29

PU LIPPINCOTT WILLIAMS & WILKINS

PI PHILADELPHIA

PA TWO COMMERCE SQ, 2001 MARKET ST, PHILADELPHIA, PA 19103 USA

SN 0025-7974

EI 1536-5964

J9 MEDICINE

JI Medicine (Baltimore)

PD SEP

PY 2016

VL 95

IS 37

AR e4935

DI 10.1097/MD.0000000000004935

PG 8

WC Medicine, General & Internal

WE Science Citation Index Expanded (SCI-EXPANDED); Social Science Citation Index (SSCI)

SC General & Internal Medicine

GA DX5ZO

UT WOS:000384461600074

PM 27631272

OA Green Published, gold

DA 2022-08-17

ER

PT J

AU Scharoun, SM

Bryden, PJ

Otipkova, Z

Musalek, M

Lejcarova, A

AF Scharoun, S. M.

Bryden, P. J.

Otipkova, Z.

Musalek, M.

Lejcarova, A.

TI Motor skills in Czech children with attention-deficit/hyperactivity

disorder and their neurotypical counterparts

SO RESEARCH IN DEVELOPMENTAL DISABILITIES

LA English

DT Article

DE Attention-deficit/hyperactivity disorder (ADHD); Fine motor skills;

Gross motor skills; Children

ID DEFICIT-HYPERACTIVITY DISORDER; HYPERKINETIC DISORDER;

GENDER-DIFFERENCES; SUBTLE SIGNS; ADHD; PERFORMANCE; COORDINATION;

PREFERENCE; HANDEDNESS; FINE

AB Attention-deficit/hyperactivity disorder (ADHD) is the most commonly diagnosed neurobehavioural disorder. Characterized by recurring problems with impulsiveness and inattention in combination with hyperactivity, motor impairments have also been well documented in the literature. The aim of this study was to compare the fine and gross motor skills of male and female children with ADHD and their neurotypical counterparts within seven skill assessments. This included three fine motor tasks: (1) spiral tracing, (2) dot filling, (3) tweezers and beads; and four gross motor tasks: (1) twistbox, (2) foot tapping, (3) small plate finger tapping, and (4) large plate finger tapping. It was hypothesized that children with ADHD would display poorer motor skills in comparison to neurotypical controls in both fine and gross motor assessments. However, statistically significant differences between the groups only emerged in four of the seven tasks (spiral tracing, dot filling, tweezers and beads and foot tapping). In line with previous findings, the complexity underlying upper limb tasks solidified the divide in performance between children with ADHD and their neurotypical counterparts. In light of similar research, impairments in lower limb motor skill were also observed. Future research is required to further delineate trends in motor difficulties in ADHD, while further investigating the underlying mechanisms of impairment. (C) 2013 Elsevier Ltd. All rights reserved.

C1 [Scharoun, S. M.] Univ Waterloo, Dept Kinesiol, Waterloo, ON N2L 3G1, Canada.

[Bryden, P. J.] Wilfrid Laurier Univ, Dept Kinesiol & Phys Educ, Waterloo, ON N2L 3C5, Canada.

[Otipkova, Z.; Musalek, M.; Lejcarova, A.] Charles Univ Prague, Fac Phys Educ & Sport, Prague 16252, Czech Republic.

RP Scharoun, SM (通讯作者)，Univ Waterloo, Dept Kinesiol, 200 Univ Ave West, Waterloo, ON N2L 3G1, Canada.

EM sscharou@uwaterloo.ca; pbryden@wlu.ca; z.otipkova@seznam.cz;

musalek.martin@seznam.cz; lejcarova@ftvs.cuni.cz

RI Bryden, Pam/AAF-6706-2020; Musalek, Martin/M-9802-2017; Benson, Sara

Scharoun/AAG-5946-2020

OI Musalek, Martin/0000-0003-1070-8304; Benson, Sara

Scharoun/0000-0002-3329-6392; Bryden, Pamela/0000-0001-8862-3402

CR American Psychiatric Association, 2000, DIAGN STAT MAN MENT

ANNETT M, 1970, BRIT J PSYCHOL, V61, P545, DOI 10.1111/j.2044-8295.1970.tb01274.x

Barkley R. A., 1998, ATTENTION DEFICIT HY

Barkley RA, 2003, BRAIN DEV-JPN, V25, P77, DOI 10.1016/S0387-7604(02)00152-3

BARKLEY RA, 1990, J CONSULT CLIN PSYCH, V58, P775, DOI 10.1037/0022-006X.58.6.775

BARNSLEY RH, 1970, PERCEPT MOTOR SKILL, V30, P343, DOI 10.2466/pms.1970.30.2.343

Biederman J, 2002, AM J PSYCHIAT, V159, P36, DOI 10.1176/appi.ajp.159.1.36

Biederman J, 2005, LANCET, V366, P237, DOI 10.1016/S0140-6736(05)66915-2

Biederman J, 2006, BIOL PSYCHIAT, V60, P1098, DOI 10.1016/j.biopsych.2006.02.031

Brown R. T., 2008, CHILDHOOD MENTAL HLT

Cole WR, 2008, NEUROLOGY, V71, P1514, DOI 10.1212/01.wnl.0000334275.57734.5f

Cortese Samuele, 2012, Curr Top Behav Neurosci, V9, P199, DOI 10.1007/7854_2011_154

Cummins A, 2005, DEV MED CHILD NEUROL, V47, P437, DOI 10.1017/S001216220500085X

Das Banerjee T, 2007, ACTA PAEDIATR, V96, P1269, DOI 10.1111/j.1651-2227.2007.00430.x

DENCKLA MB, 1978, ANN NEUROL, V3, P231, DOI 10.1002/ana.410030308

DENCKLA MB, 1985, PSYCHOPHARMACOL BULL, V21, P773

Dopfner M, 2008, EUR CHILD ADOLES PSY, V17, P59, DOI 10.1007/s00787-008-1007-y

Flapper BCT, 2006, DEV MED CHILD NEUROL, V48, P165, DOI 10.1017/S0012162206000375

Fliers E, 2008, J NEURAL TRANSM, V115, P211, DOI 10.1007/s00702-007-0827-0

Fliers EA, 2010, CHILD ADOL MENT H-UK, V15, P85, DOI 10.1111/j.1475-3588.2009.00538.x

Gaub M, 1997, J AM ACAD CHILD PSY, V36, P1036, DOI 10.1097/00004583-199708000-00011

Gershon J, 2002, J Atten Disord, V5, P143, DOI 10.1177/108705470200500302

Gillberg C, 2004, EUR CHILD ADOLES PSY, V13, P80, DOI 10.1007/s00787-004-1008-4

Goldstein ML, 2011, CHRONIC DISORDERS IN CHILDREN AND ADOLESCENTS, P99, DOI 10.1007/978-1-4419-9764-7_5

Goodman R, 1997, J CHILD PSYCHOL PSYC, V38, P581, DOI 10.1111/j.1469-7610.1997.tb01545.x

Goulardins JB, 2013, RES DEV DISABIL, V34, P40, DOI 10.1016/j.ridd.2012.07.014

HARTSOUGH CS, 1985, AM J ORTHOPSYCHIAT, V55, P190, DOI 10.1111/j.1939-0025.1985.tb03433.x

Harvey WJ, 2007, J ABNORM CHILD PSYCH, V35, P871, DOI 10.1007/s10802-007-9140-5

Harvey WJ, 2003, ADAPT PHYS ACT Q, V20, P1, DOI 10.1123/apaq.20.1.1

Harvey WJ, 1997, ADAPT PHYS ACT Q, V14, P189, DOI 10.1123/apaq.14.3.189

Havluj J., 2002, WECHSLER INTELLIGENC

HENDERSON SE, 1992, MOVEMENT ASSESSMENT

Hinshaw S. P., 1993, ANN M INT SOC RES CH

Jancke J, 1998, COGNITIVE BRAIN RES, V6, P279

Johansen EB, 2002, BEHAV BRAIN RES, V130, P37, DOI 10.1016/S0166-4328(01)00434-X

Kadesjo B, 2001, J CHILD PSYCHOL PSYC, V42, P487, DOI 10.1111/1469-7610.00742

Kalff AC, 2003, J CHILD PSYCHOL PSYC, V44, P1049, DOI 10.1111/1469-7610.00189

Karatekin C, 2003, PERCEPT MOTOR SKILL, V97, P1267, DOI 10.2466/pms.2003.97.3f.1267

Lange KW, 2010, ADHD-ATTEND DEFICIT, V2, P241, DOI 10.1007/s12402-010-0045-8

Lavasani NM, 2011, OCCUP THER INT, V18, P106, DOI 10.1002/oti.306

Leung PWL, 1998, DEV MED CHILD NEUROL, V40, P600

Melnick SM, 2000, J ABNORM CHILD PSYCH, V28, P73, DOI 10.1023/A:1005174102794

Miyahara M, 2006, HUM MOVEMENT SCI, V25, P100, DOI 10.1016/j.humov.2005.11.004

Morano M, 2011, SCAND J MED SCI SPOR, V21, P465, DOI 10.1111/j.1600-0838.2009.01068.x

Musalek M., 2012, THESIS CHARLES U PRA

Niederhofer H, 2005, PERCEPT MOTOR SKILL, V101, P808, DOI 10.2466/pms.101.3.808-810

Ohan JL, 2005, CHILD PSYCHIAT HUM D, V35, P359, DOI 10.1007/s10578-005-2694-y

Pan CY, 2009, J AUTISM DEV DISORD, V39, P1694, DOI 10.1007/s10803-009-0813-5

Pedersen Scott J, 2007, J Atten Disord, V10, P343, DOI 10.1177/1087054706292112

PETERS M, 1990, Neuropsychology Review, V1, P165, DOI 10.1007/BF01108716

PETERS M, 1988, PSYCHOL BULL, V103, P179, DOI 10.1037/0033-2909.103.2.179

Piek JP, 1999, DEV MED CHILD NEUROL, V41, P159, DOI 10.1017/S0012162299000341

Pitcher TM, 2003, DEV MED CHILD NEUROL, V45, P525, DOI 10.1111/j.1469-8749.2003.tb00952.x

Polanczyk G, 2007, AM J PSYCHIAT, V164, P942, DOI 10.1176/appi.ajp.164.6.942

Racine MB, 2008, J CHILD NEUROL, V23, P399, DOI 10.1177/0883073807309244

RIGAL RA, 1992, PERCEPT MOTOR SKILL, V75, P851, DOI 10.2466/pms.1992.75.3.851

Riverin M, 2009, INT J OBESITY, V33, P945, DOI 10.1038/ijo.2009.125

Rodriguez A, 2010, PEDIATRICS, V125, pE340, DOI 10.1542/peds.2009-1165

Rohde Luis A., 2004, J. Pediatr. (Rio J.), V80, P61, DOI 10.1590/S0021-75572004000300009

Rommelse NNJ, 2007, J CHILD PSYCHOL PSYC, V48, P1071, DOI 10.1111/j.1469-7610.2007.01781.x

Rosa Neto F., 2002, MANUAL AVALIACAO MOT

Rucklidge JJ, 2010, PSYCHIAT CLIN N AM, V33, P357, DOI 10.1016/j.psc.2010.01.006

Sagvolden T, 2005, BEHAV BRAIN SCI, V28, P397, DOI 10.1017/S0140525X05000075

Sagvolden T., 2006, BEHAV BRAIN FUNCT, V2, P33, DOI [DOI 10.1186/1744-9081-2-33, 10.1186/1744-9081-2-33]

Schlee G, 2012, RES DEV DISABIL, V33, P1957, DOI 10.1016/j.ridd.2012.05.020

Schuerholz LJ, 1997, J CHILD NEUROL, V12, P438, DOI 10.1177/088307389701200705

Seidman LJ, 1997, J CONSULT CLIN PSYCH, V65, P150, DOI 10.1037/0022-006X.65.1.150

Seidman LJ, 2005, DEV NEUROPSYCHOL, V27, P79, DOI 10.1207/s15326942dn2701_4

Sergeant JA, 2006, HUM MOVEMENT SCI, V25, P76, DOI 10.1016/j.humov.2005.10.007

Shaw P, 2007, P NATL ACAD SCI USA, V104, P19649, DOI 10.1073/pnas.0707741104

Shum SBM, 2009, J PEDIATR-US, V155, P245, DOI 10.1016/j.jpeds.2009.02.032

Skinner RA, 2001, HUM MOVEMENT SCI, V20, P73, DOI 10.1016/S0167-9457(01)00029-X

Springer S. P., 1998, LEFT BRAIN RIGHT BRA

TAPLEY SM, 1985, NEUROPSYCHOLOGIA, V23, P215, DOI 10.1016/0028-3932(85)90105-8

Taylor E, 2004, EUR CHILD ADOLES PSY, V13, P7, DOI 10.1007/s00787-004-1002-x

Tervo RC, 2002, DEV MED CHILD NEUROL, V44, P383

TIFFIN J, 1968, PURDUE PEGBOARD EXAM

Tiffin J, 1948, J APPL PSYCHOL, V32, P234, DOI 10.1037/h0061266

Tseng MH, 2004, DEV MED CHILD NEUROL, V46, P381, DOI 10.1017/S0012162204000623

Ulrich D.A, 2000, TEST GROSS MOTOR DEV, V2nd ed

Ulrich DA, 1985, TEST GROSS MOTOR DEV

Visser J, 2003, HUM MOVEMENT SCI, V22, P479, DOI 10.1016/j.humov.2003.09.005

WADE MG, 1976, J LEARN DISABIL, V9, P443, DOI 10.1177/002221947600900711

Wang Juan, 2003, Beijing Da Xue Xue Bao Yi Xue Ban, V35, P280

World Health Organization (WHO), 1993, INT STAT CLASS DIS R, V10

Yahagi S, 1999, NEUROSCI LETT, V276, P185, DOI 10.1016/S0304-3940(99)00823-X

Zang Y, 2002, CHIN J CLIN REHABIL, V6, P1372

NR 87

TC 23

Z9 25

U1 0

U2 18

PU PERGAMON-ELSEVIER SCIENCE LTD

PI OXFORD

PA THE BOULEVARD, LANGFORD LANE, KIDLINGTON, OXFORD OX5 1GB, ENGLAND

SN 0891-4222

J9 RES DEV DISABIL

JI Res. Dev. Disabil.

PD NOV

PY 2013

VL 34

IS 11

BP 4142

EP 4153

DI 10.1016/j.ridd.2013.08.011

PG 12

WC Education, Special; Rehabilitation

WE Social Science Citation Index (SSCI)

SC Education & Educational Research; Rehabilitation

GA 251BD

UT WOS:000326901900053

PM 24060728

DA 2022-08-17

ER

PT J

AU Montes-Montes, R

Delgado-Lobete, L

Rodriguez-Seoane, S

AF Montes-Montes, Rebeca

Delgado-Lobete, Laura

Rodriguez-Seoane, Sara

TI Developmental Coordination Disorder, Motor Performance, and Daily

Participation in Children with Attention Deficit and Hyperactivity

Disorder

SO CHILDREN-BASEL

LA English

DT Article

DE attention deficit hyperactivity disorder; developmental coordination

disorder; comorbidity; motor performance; daily participation;

activities of daily living; occupational therapy

AB Children with Attention Deficit and Hyperactivity Disorder (ADHD) often present with Developmental Coordination Disorder (DCD) or motor coordination problems that further impact their daily functioning. However, little is known about the prevalence of co-occurring DCD and ADHD in the Spanish context, and research about the impact of ADHD on performance and participation in motor-based activities of daily living (ADL) is scarce. The aims of this study were to explore the prevalence of co-occurring DCD in children with ADHD, and to examine differences in performance and participation in motor-based ADL between children with ADHD and typically developing children. We conducted a case-control study including 20 children with ADHD and 40 typically developing controls randomly matched for exact age and sex (males = 80%; mean age = 8, 9 (2, 3) years). Presence of probable DCD (p-DCD) was confirmed with the Developmental Coordination Disorder Questionnaire (DCDQ). The DCDDaily-Q was administered to assess performance and participation in ADL. A 75% prevalence of p-DCD was found in the ADHD group (OR = 27; p < 0.001). Children with ADHD showed poorer motor performance and less participation in ADL (p < 0.01; d = 0.9-1.4). These findings contribute to understand the functional consequences of ADHD in motor-based ADL and its relationship with DCD.

C1 [Montes-Montes, Rebeca] Univ A Coruna, Ctr Informat & Commun Technol Res CITIC, TALIONIS Res Grp, Res Ctr,Galician Univ Syst, La Coruna 15008, Spain.

[Delgado-Lobete, Laura] Univ A Coruna, Hlth Integrat & Promot Res Unit INTEGRA SAUDE, Fac Hlth Sci, La Coruna 15011, Spain.

[Rodriguez-Seoane, Sara] Spanish Cerebral Palsy Assoc ASPACE, Gijon 33394, Spain.

RP Delgado-Lobete, L (通讯作者)，Univ A Coruna, Hlth Integrat & Promot Res Unit INTEGRA SAUDE, Fac Hlth Sci, La Coruna 15011, Spain.

EM rebeca.montes@udc.es; l.delgado@udc.es; sarose79@gmail.com

RI Montes-Montes, Rebeca/AAK-8141-2021; Montes-Montes, Rebeca/C-1021-2017

OI Montes-Montes, Rebeca/0000-0003-3156-2308; Delgado-Lobete,

Laura/0000-0002-6460-1548

FU European Social Fund; Xunta de Galicia [ED481A-2018/150]

FX This research was partially funded by the European Social Fund 2014-2020

and Xunta de Galicia, grant number ED481A-2018/150.

CR Amador-Ruiz S, 2018, J SCHOOL HEALTH, V88, P538, DOI 10.1111/josh.12639

[Anonymous], 2020, Am J Occup Ther, V74, p7412410010p1, DOI 10.5014/ajot.2020.74S2001

[Anonymous], 2013, DIAGN STAT MAN MENT, V5th

Blanco-Martinez N, 2020, CHILDREN-BASEL, V7, DOI 10.3390/children7100157

Blank R, 2019, DEV MED CHILD NEUROL, V61, P242, DOI 10.1111/dmcn.14132

Marino MC, 2018, AN PEDIATR, V89, P153, DOI 10.1016/j.anpedi.2017.10.007

Catala-Lopez F, 2012, BMC PSYCHIATRY, V12, DOI 10.1186/1471-244X-12-168

Cleaton MAM., 2018, J CHILDHOOD DEV DISO, DOI [10.4172/2472-1786.100073, DOI 10.4172/2472-1786.1000]

Dawson PMT, 2020, S AFR J CHILD HEALTH, V14, P180, DOI 10.7196/SAJCH.2020.v14i4.1676

de Schipper E, 2015, EUR CHILD ADOLES PSY, V24, P859, DOI 10.1007/s00787-015-0727-z

Delgado-Lobete L, 2020, INT J ENV RES PUB HE, V17, DOI 10.3390/ijerph17134802

Delgado-Lobete L, 2020, INT J ENV RES PUB HE, V17, DOI 10.3390/ijerph17051705

Delgado-Lobete L, 2020, RES DEV DISABIL, V100, DOI 10.1016/j.ridd.2020.103608

Delgado-Lobete L, 2019, RES DEV DISABIL, V86, P31, DOI 10.1016/j.ridd.2019.01.004

Ferguson CJ, 2009, PROF PSYCHOL-RES PR, V40, P532, DOI 10.1037/a0015808

Fliers E, 2008, J NEURAL TRANSM, V115, P211, DOI 10.1007/s00702-007-0827-0

Glascoe FP, 2000, CHILD CARE HLTH DEV, V26, P137, DOI 10.1046/j.1365-2214.2000.00173.x

Goulardins JB, 2017, PERCEPT MOTOR SKILL, V124, P425, DOI 10.1177/0031512517690607

Goulardins JB, 2018, J ATTEN DISORD, V22, P796, DOI 10.1177/1087054715580394

Goulardins JB, 2015, BEHAV BRAIN RES, V292, P484, DOI 10.1016/j.bbr.2015.07.009

Gravetter F.D., 2014, ESSENTIALS STAT BEHA, V8th ed.

Hennessy S, 1999, AM J EPIDEMIOL, V149, P195

Izadi-Najafabadi S, 2019, RES DEV DISABIL, V84, P75, DOI 10.1016/j.ridd.2018.05.011

Kadesjo B, 2001, J CHILD PSYCHOL PSYC, V42, P487, DOI 10.1111/1469-7610.00742

Kaplan B, 2006, CHILD CARE HLTH DEV, V32, P723, DOI 10.1111/j.1365-2214.2006.00689.x

Magalhaes LC, 2011, RES DEV DISABIL, V32, P1309, DOI 10.1016/j.ridd.2011.01.029

McLeod KR, 2016, NEUROIMAGE-CLIN, V12, P157, DOI 10.1016/j.nicl.2016.06.019

Montes-Montes R, 2020, AM J OCCUP THER, V74, DOI 10.5014/ajot.2020.038315

Montes-Montes R, 2020, INT J ENV RES PUB HE, V17, DOI 10.3390/ijerph17072425

Montes-Montes R, 2020, INT J ENV RES PUB HE, V17, DOI 10.3390/ijerph17020555

Polanczyk GV, 2014, INT J EPIDEMIOL, V43, P434, DOI 10.1093/ije/dyt261

Rasmussen P, 2000, J AM ACAD CHILD PSY, V39, P1424, DOI 10.1097/00004583-200011000-00017

Ruiz L.M., 2018, J PHYS ED, V8, P32

Shaw M, 2012, BMC MED, V10, DOI 10.1186/1741-7015-10-99

Smits-Engelsman B, 2015, HUM MOVEMENT SCI, V42, P293, DOI 10.1016/j.humov.2015.03.010

Soref B, 2012, CHILD CARE HLTH DEV, V38, P561, DOI 10.1111/j.1365-2214.2011.01295.x

Suazo D.M., 2019, CUAD PISCOL DEPORTE, V19, P37

van der Linde BW, 2015, CHILD CARE HLTH DEV, V41, P23, DOI 10.1111/cch.12124

Van der Linde BW, 2015, PHYS THER, V95, P1496, DOI 10.2522/ptj.20140211

van der Linde BW, 2014, RES DEV DISABIL, V35, P1711, DOI 10.1016/j.ridd.2014.03.008

Villa M., 2019, RETOS, V36, P625

Villa M., 2020, CUAD PISCOL DEPORTE, V20, P47

Wall AET, 2004, ADAPT PHYS ACT Q, V21, P197, DOI 10.1123/apaq.21.3.197

Williams W., 2015, DYSPRAXIA FOUND PROF, V13, P21

Wilson BN, 2009, PHYS OCCUP THER PEDI, V29, P182, DOI 10.1080/01942630902784761

World Health Organization, 2007, PLOS ONE

NR 46

TC 2

Z9 2

U1 5

U2 12

PU MDPI

PI BASEL

PA ST ALBAN-ANLAGE 66, CH-4052 BASEL, SWITZERLAND

EI 2227-9067

J9 CHILDREN-BASEL

JI Children-Basel

PD MAR

PY 2021

VL 8

IS 3

AR 187

DI 10.3390/children8030187

PG 10

WC Pediatrics

WE Science Citation Index Expanded (SCI-EXPANDED); Social Science Citation Index (SSCI)

SC Pediatrics

GA RD4YT

UT WOS:000633486200001

PM 33804502

OA gold, Green Published

DA 2022-08-17

ER

PT J

AU Goyen, TA

Lui, K

Hummell, J

AF Goyen, Traci-Anne

Lui, Kei

Hummell, Jill

TI Sensorimotor skills associated with motor dysfunction in children born

extremely preterm

SO EARLY HUMAN DEVELOPMENT

LA English

DT Article

DE Preterm infants; Developmental Coordination Disorder

ID LOW-BIRTH-WEIGHT; DEVELOPMENTAL COORDINATION DISORDER;

COGNITIVE-ABILITIES; REGIONAL COHORT; VISUAL-MOTOR; SCHOOL-AGE;

FOLLOW-UP; IMPAIRMENT; GESTATION; INFANTS

AB Background: Children born prematurely, despite being free of intellectual and sensorineural deficits, are at risk of motor dysfunction.

Aim: To investigate the association of sensorimotor processing skills and Developmental Coordination Disorder (DCD) in "apparently normal" extreme preterm children.

Study design: In a matched case-control study, 50 preterm children born less than 29 weeks or birthweight < 1000g. with an IQ > 85 and no identified sensorineural disability, were assessed at 8 years of age along with 50 gender and birth date matched classroom controls born at full term. A battery of sensorimotor tests was administered, which examined visual-motor, visual perception, tactile perception, kinaesthesia, and praxis.

Results: For preterm children with DCD (n = 21), significantly lower scores were found for the visual processing and praxis tests, with the exception of verbal command, in comparison to those 29 preterm children without DCD and term controls (median visual perception scores were 92,96 and 108 respectively; design copying was 0.07, 0.46 and 0.95; constructive praxis was 0.09, 0.27 and 0.63; and sequencing praxis was 0.14, 0.73 and 0.96). There were no difference on the tactile sensitivity and kinaesthetic processing tests.

Conclusions: Preterm children with DCD have difficulty with visual processing tasks. Praxis or motor planning poses a particular challenge for them. Motor dysfunction in extremely preterm children was related to poorer visual processing and motor planning and may relate to a cognitive processing problem. Crown Copyright (c) 2011 Published by Elsevier Ireland Ltd. All rights reserved.

C1 [Goyen, Traci-Anne] Westmead Hosp, Ctr Newborn Care, Westmead, NSW 2145, Australia.

[Goyen, Traci-Anne; Lui, Kei] Univ New S Wales, Sch Womens & Childrens Hlth, Sydney, NSW, Australia.

[Hummell, Jill] Westmead Hosp, Brain Injury Rehabil Serv, Westmead, NSW 2145, Australia.

RP Goyen, TA (通讯作者)，Westmead Hosp, Ctr Newborn Care, Westmead, NSW 2145, Australia.

EM tagoyen@optushome.com.au

RI Lui, Kei/F-3710-2015

OI Lui, Kei/0000-0001-9884-3521

CR Ayres A.J., 1989, SENSORY INTEGRATION, V1st

Berry K., 1997, BEERY BUKTENICA DEVE

BOWEN JR, 1993, J PAEDIATR CHILD H, V29, P276, DOI 10.1111/j.1440-1754.1993.tb00511.x

BURNS YR, 1999, PHYSIOTHERAPY, P7

Cooke RWI, 2004, ARCH DIS CHILD-FETAL, V89, pF249, DOI 10.1136/adc.2002.023374

Dammann O, 1996, DEV MED CHILD NEUROL, V38, P97

Davis NM, 2007, DEV MED CHILD NEUROL, V49, P325, DOI 10.1111/j.1469-8749.2007.00325.x

de Kieviet JF, 2009, JAMA-J AM MED ASSOC, V302, P2235, DOI 10.1001/jama.2009.1708

DWYER C, 1994, ADAPT PHYS ACT Q, V11, P179

Foreman N, 1997, J EXP CHILD PSYCHOL, V64, P27, DOI 10.1006/jecp.1996.2326

Foulder-Hughes LA, 2003, DEV MED CHILD NEUROL, V45, P97, DOI 10.1017/S0012162203000197

Goyen TA, 2009, ARCH DIS CHILD, V94, P298, DOI 10.1136/adc.2007.134692

Goyen TA, 1998, DEV MED CHILD NEUROL, V40, P76, DOI 10.1111/j.1469-8749.1998.tb15365.x

Gray PH, 2004, J PAEDIATR CHILD H, V40, P114, DOI 10.1111/j.1440-1754.2004.00310.x

Hammill D. D., 1996, MOTOR FREE VISUAL PE

Hard AL, 2000, DEV MED CHILD NEUROL, V42, P100, DOI 10.1017/S0012162200000207

Holsti L, 2002, J DEV BEHAV PEDIATR, V23, P9, DOI 10.1097/00004703-200202000-00002

Jakobson LS, 2001, J PEDIATR PSYCHOL, V26, P503, DOI 10.1093/jpepsy/26.8.503

JONGMANS MJ, 1998, HUM MOVEMENT SCI, V17, P699

Korkman M, 1996, J CLIN EXP NEUROPSYC, V18, P220, DOI 10.1080/01688639608408277

Langaas T, 1998, VISION RES, V38, P1817, DOI 10.1016/S0042-6989(97)00399-4

LASZLO JI, 1985, TEST KINAESTHETIC SE

LORD R, 1987, DEV MED CHILD NEUROL, V29, P720

Luoma L, 1998, DEV MED CHILD NEUROL, V40, P21, DOI 10.1111/j.1469-8749.1998.tb15352.x

Missiuna Cheryl, 2001, Physical and Occupational Therapy in Pediatrics, V20, P69, DOI 10.1300/J006v20n02_05

MONWILLIAMS MA, 1994, ADAPT PHYS ACT Q, V11, P170

Olsen P, 1998, PEDIATRICS, V102, P329, DOI 10.1542/peds.102.2.329

POWLS A, 1995, ARCH DIS CHILD-FETAL, V73, pF62, DOI 10.1136/fn.73.2.F62

SAIGAL S, 1991, J PEDIATR-US, V118, P751, DOI 10.1016/S0022-3476(05)80043-5

Schoemaker MM, 2003, BRIT J EDUC PSYCHOL, V73, P425, DOI 10.1348/000709903322275911

SEMMES J, 1960, SEMMESWEINSTEIN AEST

THIBAULT A, 1994, DEV MED CHILD NEUROL, V36, P796

Whitfield MF, 2000, CLIN PERINATOL, V27, P363, DOI 10.1016/S0095-5108(05)70026-9

Williams J, 2010, DEV MED CHILD NEUROL, V52, P232, DOI 10.1111/j.1469-8749.2009.03544.x

Wilson PH, 1997, DEV MED CHILD NEUROL, V39, P736

NR 35

TC 28

Z9 30

U1 1

U2 21

PU ELSEVIER IRELAND LTD

PI CLARE

PA ELSEVIER HOUSE, BROOKVALE PLAZA, EAST PARK SHANNON, CO, CLARE, 00000,

IRELAND

SN 0378-3782

EI 1872-6232

J9 EARLY HUM DEV

JI Early Hum. Dev.

PD JUL

PY 2011

VL 87

IS 7

BP 489

EP 493

DI 10.1016/j.earlhumdev.2011.04.002

PG 5

WC Obstetrics & Gynecology; Pediatrics

WE Science Citation Index Expanded (SCI-EXPANDED)

SC Obstetrics & Gynecology; Pediatrics

GA 791MQ

UT WOS:000292669700006

PM 21549531

DA 2022-08-17

ER

PT J

AU Yu, JJ

Capio, CM

Abernethy, B

Sit, CHP

AF Yu, Jane Jie

Capio, Catherine M.

Abernethy, Bruce

Sit, Cindy Hui Ping

TI Moderate-to-vigorous physical activity and sedentary behavior in

children with and without developmental coordination disorder:

Associations with fundamental movement skills

SO RESEARCH IN DEVELOPMENTAL DISABILITIES

LA English

DT Article

DE Physical activity; Motor skills; DCD; Object control skills; Ball

skills; Motor skill disorders

ID MOTOR PROFICIENCY; REPORT CARD; INTERVENTION; PERFORMANCE; COMPETENCE

AB Background: There is a pandemic of physical inactivity in children. Compared to children with typical development (TD), those with developmental coordination disorder (DCD) are less physically active. Mastery of movement skills such as fundamental movement skills (FMS) are potential correlates of physical activity. Aims: To examine the associations of FMS with moderate-to-vigorous physical activity (MVPA) and sedentary behavior (SB) in children with DCD and TD. Methods and procedures: This cross-sectional study included 172 children with DCD (n = 73; boys = 49) or TD (n = 99; boys = 48) aged 6-10 years. Five components of FMS (running, jumping, throwing, catching, kicking) were assessed using process- and product- oriented approaches using the Test of Gross Motor Development-second edition. The time spent in MVPA and SB was measured using accelerometers. Outcomes and results: Children with DCD had significantly poorer performance in FMS proficiency in terms of specific movement patterns (jumping and catching) and outcomes (running, jumping, catching, and kicking) when compared to children with TD. MVPA and SB were significantly associated with certain process-oriented FMS assessments in children, which was moderated by motor coordination status and sex. Movement patterns of catching (odds ratio = 1.686, p < .05) was a significant predictor of children's attainment of the MVPA guideline. Conclusions and implications: It is important to develop FMS patterns and ball skills in early childhood, particularly for children with DCD, to combat physical inactivity and its related health problems.

C1 [Yu, Jane Jie] Zhejiang Univ, Dept Sport & Exercise Sci, Hangzhou, Peoples R China.

[Capio, Catherine M.] Educ Univ Hong Kong, Dept Early Childhood Educ, Hong Kong, Peoples R China.

[Abernethy, Bruce] Univ Queensland, Fac Hlth & Behav Sci, Brisbane, Qld, Australia.

[Sit, Cindy Hui Ping] Chinese Univ Hong Kong, Dept Sports Sci & Phys Educ, Hong Kong, Peoples R China.

RP Sit, CHP (通讯作者)，Chinese Univ Hong Kong, Shatin, Room G06,Kwok Sports Bldg, Hong Kong, Peoples R China.

EM sithp@cuhk.edu.hk

OI Capio, Catherine M./0000-0003-1698-5740

FU Health and Medical Research Fund from the Food and Health Bureau of the

Government of the Hong Kong Special Administrative Region, China

[11120781]; Fundamental Research Funds for the Central Universities,

China; "Double First-Class" University Construction Fund, China

FX This study was funded by the Health and Medical Research Fund from the

Food and Health Bureau of the Government of the Hong Kong Special

Administrative Region, China (reference number: 11120781).; Jane Jie Yu

was supported by the Fundamental Research Funds for the Central

Universities, China and "Double First-Class" University Construction

Fund, China.

CR American Psychiatric Association (APA), 2013, DIAGNOSTIC STAT MANU, V5th, DOI [10.1176/appi.books.9780890425596, DOI 10.1176/APPI.BOOKS.9780890425596]

Andersen LB, 2016, LANCET, V388, P1255, DOI 10.1016/S0140-6736(16)30960-6

Atkin AJ, 2012, INT J EPIDEMIOL, V41, P1460, DOI 10.1093/ije/dys118

Aubert S, 2018, J PHYS ACT HEALTH, V15, pS251, DOI 10.1123/jpah.2018-0472

Barnes J, 2012, APPL PHYSIOL NUTR ME, V37, P540, DOI [10.1139/h2012-024, 10.1139/H2012-024]

Barnett LM, 2019, J SPORT SCI, V37, P492, DOI 10.1080/02640414.2018.1508399

Barnett LM, 2009, J ADOLESCENT HEALTH, V44, P252, DOI 10.1016/j.jadohealth.2008.07.004

Basterfield L, 2011, J PHYS ACT HEALTH, V8, P543, DOI 10.1123/jpah.8.4.543

Basterfield L, 2011, PEDIATRICS, V127, pE24, DOI 10.1542/peds.2010-1935

Batey CA, 2014, HUM MOVEMENT SCI, V36, P258, DOI 10.1016/j.humov.2013.10.003

Blank R, 2012, DEV MED CHILD NEUROL, V54, P54, DOI 10.1111/j.1469-8749.2011.04171.x

Capio CM, 2012, RES DEV DISABIL, V33, P1235, DOI 10.1016/j.ridd.2012.02.020

Choi L, 2011, MED SCI SPORT EXER, V43, P357, DOI 10.1249/MSS.0b013e3181ed61a3

De Meester A, 2018, J SCI MED SPORT, V21, P58, DOI 10.1016/j.jsams.2017.05.007

Engel AC, 2018, SPORTS MED, V48, P1845, DOI 10.1007/s40279-018-0923-3

Evenson KR, 2008, J SPORT SCI, V26, P1557, DOI 10.1080/02640410802334196

Faigenbaum AD, 2009, J STRENGTH COND RES, V23, pS60, DOI 10.1519/JSC.0b013e31819df407

Green D, 2011, RES DEV DISABIL, V32, P1332, DOI 10.1016/j.ridd.2011.01.040

Haga M, 2009, PHYS THER, V89, P1089, DOI 10.2522/ptj.20090052

Hinkley T, 2014, PREV MED, V62, P182, DOI 10.1016/j.ypmed.2014.02.007

Huang WY, 2019, J EXERC SCI FIT, V17, P14, DOI 10.1016/j.jesf.2018.10.003

Kelly L, 2019, J SPORT SCI, V37, P1055, DOI 10.1080/02640414.2018.1543833

Kim CI, 2014, RES DEV DISABIL, V35, P800, DOI 10.1016/j.ridd.2014.01.019

Kwan MYW, 2016, HUM MOVEMENT SCI, V47, P159, DOI 10.1016/j.humov.2016.03.004

Logan S. W., 2015, KINESIOL REV, V4, P416, DOI DOI 10.1123/KR.2013-0012

Logan SW, 2017, J SPORT SCI, V35, P634, DOI 10.1080/02640414.2016.1183803

Lopes L, 2012, AM J HUM BIOL, V24, P746, DOI 10.1002/ajhb.22310

Lubans DR, 2010, SPORTS MED, V40, P1019, DOI 10.2165/11536850-000000000-00000

Pang AWY, 2009, RES SPORTS MED, V17, P125, DOI 10.1080/15438620902897516

Pau M, 2021, MEDICINE, V100, DOI 10.1097/MD.0000000000024931

Poitras VJ, 2016, APPL PHYSIOL NUTR ME, V41, pS197, DOI 10.1139/apnm-2015-0663

Robinson LE, 2015, SPORTS MED, V45, P1273, DOI 10.1007/s40279-015-0351-6

Sallis JF, 2016, LANCET, V388, P1325, DOI 10.1016/S0140-6736(16)30581-5

Sit CHP, 2019, RES DEV DISABIL, V89, P1, DOI 10.1016/j.ridd.2019.03.004

Slykerman S, 2016, J SCI MED SPORT, V19, P488, DOI 10.1016/j.jsams.2015.07.002

Ulrich D.A, 2000, TEST GROSS MOTOR DEV, V2nd ed

WHO, 2010, SCREENING DONATED BLOOD FOR TRANSFUSION: TRANSMISSIBLE INFECTIONS, P1

Wilson PH, 2013, DEV MED CHILD NEUROL, V55, P217, DOI 10.1111/j.1469-8749.2012.04436.x

Wright KE, 2019, RES DEV DISABIL, V84, P66, DOI 10.1016/j.ridd.2018.05.013

Wrotniak BH, 2006, PEDIATRICS, V118, pE1758, DOI 10.1542/peds.2006-0742

Yu J, 2016, ADAPT PHYS ACT Q, V33, P134, DOI 10.1123/APAQ.2015-0008

Yu J, 2016, DISABIL REHABIL, V38, P45, DOI 10.3109/09638288.2015.1014067

NR 42

TC 2

Z9 2

U1 9

U2 17

PU PERGAMON-ELSEVIER SCIENCE LTD

PI OXFORD

PA THE BOULEVARD, LANGFORD LANE, KIDLINGTON, OXFORD OX5 1GB, ENGLAND

SN 0891-4222

EI 1873-3379

J9 RES DEV DISABIL

JI Res. Dev. Disabil.

PD NOV

PY 2021

VL 118

AR 104070

DI 10.1016/j.ridd.2021.104070

EA AUG 2021

PG 10

WC Education, Special; Rehabilitation

WE Social Science Citation Index (SSCI)

SC Education & Educational Research; Rehabilitation

GA WA5BW

UT WOS:000702901400010

PM 34438197

DA 2022-08-17

ER

PT J

AU Fulceri, F

Grossi, E

Contaldo, A

Narzisi, A

Apicella, F

Parrini, I

Tancredi, R

Calderoni, S

Muratori, F

AF Fulceri, Francesca

Grossi, Enzo

Contaldo, Annarita

Narzisi, Antonio

Apicella, Fabio

Parrini, Ilaria

Tancredi, Raffaella

Calderoni, Sara

Muratori, Filippo

TI Motor Skills as Moderators of Core Symptoms in Autism Spectrum

Disorders: Preliminary Data From an Exploratory Analysis With Artificial

Neural Networks

SO FRONTIERS IN PSYCHOLOGY

LA English

DT Article

DE autism spectrum disorders; motor impairments; motor skills; repetitive

behaviors; artificial neural network; Peabody Developmental Motor Scale;

preschoolers

ID DEVELOPMENTAL COORDINATION DISORDER; REPETITIVE BEHAVIORS;

PRESCHOOL-CHILDREN; PHYSICAL-ACTIVITY; YOUNG-CHILDREN; FINE MOTOR;

INTERVENTION; IMPAIRMENT; LANGUAGE; MOVEMENT

AB Motor disturbances have been widely observed in children with autism spectrum disorder (ASD), and motor problems are currently reported as associated features supporting the diagnosis of ASD in the current Diagnostic and Statistical Manual of Mental Disorders (DSM-5). Studies on this issue reported disturbances in different motor domains, including both gross and fine motor areas as well as coordination, postural control, and standing balance. However, they failed to clearly state whether motor impairments are related to demographical and developmental features of ASD. Both the different methodological approaches assessing motor skills and the heterogeneity in clinical features of participants analyzed have been implicated as contributors to variance in findings. However, the non-linearity of the relationships between variables may account for the inability of the traditional analysis to grasp the core problem suggesting that the "single symptom approach analysis" should be overcome. Artificial neural networks (ANNs) are computational adaptive systems inspired by the functioning processes of the human brain particularly adapted to solving non-linear problems. This study aimed to apply the ANNs to reveal the entire spectrum of the relationship between motor skills and clinical variables. Thirty-two male children with ASD [mean age: 48.5 months (SD: 8.8); age range: 30-60 months] were recruited in a tertiary care university hospital. A multidisciplinary comprehensive diagnostic evaluation was associated with a standardized assessment battery for motor skills, the Peabody Developmental Motor Scale-Second Edition. Exploratory analyses were performed through the ANNs. The findings revealed that poor motor skills were a common clinical feature of preschoolers with ASD, relating both to the high level of repetitive behaviors and to the low level of expressive language. Moreover, unobvious trends among motor, cognitive and social skills have been detected. In conclusion, motor abnormalities in preschoolers with ASD were widespread, and the degree of impairment may inform clinicians about the severity of ASD core symptoms. Understanding motor disturbances in children with ASD may be relevant to clarify neurobiological basis and ultimately to guide the development of tailored treatments.

C1 [Fulceri, Francesca] Ist Super Sanita, Res Coordinat & Support Serv, Rome, Italy.

[Grossi, Enzo] Villa Santa Maria Inst, Autism Res Unit, Tavernerio, Italy.

[Contaldo, Annarita; Narzisi, Antonio; Apicella, Fabio; Parrini, Ilaria; Tancredi, Raffaella; Calderoni, Sara; Muratori, Filippo] IRCCS Fdn Stella Maris, Pisa, Italy.

[Calderoni, Sara; Muratori, Filippo] Univ Pisa, Dept Clin & Expt Med, Pisa, Italy.

RP Calderoni, S (通讯作者)，IRCCS Fdn Stella Maris, Pisa, Italy.; Calderoni, S (通讯作者)，Univ Pisa, Dept Clin & Expt Med, Pisa, Italy.

EM sara.calderoni@fsm.unipi.it

RI narzisi, antonio/K-3962-2016; Tancredi, Raffaella/C-3868-2018; Bargagna,

Stefania/AAB-7603-2019; Contaldo, Annarita/AAB-2000-2022; Fulceri,

Francesca/AAE-6412-2022; Contaldo, Annarita/AAC-1164-2021; Apicella,

Fabio/ABF-4304-2020; Calderoni, Sara/J-2279-2018; Muratori,

Filippo/K-3187-2018; Fulceri, Francesca/AAL-1582-2021; Grossi,

Enzo/AAF-7765-2020; Contaldo, Annarita/AAB-9165-2021

OI narzisi, antonio/0000-0003-3996-3827; Tancredi,

Raffaella/0000-0003-3517-4919; Bargagna, Stefania/0000-0001-6430-9494;

Fulceri, Francesca/0000-0002-6757-9585; Apicella,

Fabio/0000-0001-7390-3241; Calderoni, Sara/0000-0002-6250-5739;

Muratori, Filippo/0000-0001-9598-0096; Contaldo,

Annarita/0000-0002-3812-5031

FU European Community's Horizon 2020 Program [642996]; Italian Ministry of

Health Network Project 'Italian Autism Spectrum Disorders Network:

filling the gaps in the National Health System Care' [NET

2013-02355263]; IRCCS Fondazione StellaMaris (Ricerca Corrente); IRCCS

Fondazione StellaMaris ("5 x 1000" voluntary contributions, Italian

Ministry of Health)

FX This work has been partially supported by the European Community's

Horizon 2020 Program under the grant agreement n. 642996 (BRAINVIEW), by

the Italian Ministry of Health Network Project 'Italian Autism Spectrum

Disorders Network: filling the gaps in the National Health System Care'

(NET 2013-02355263), and by grant from the IRCCS Fondazione StellaMaris

(Ricerca Corrente, and the "5 x 1000" voluntary contributions, Italian

Ministry of Health).

CR American Psychiatric Association APA, 2013, DIAGNOSTIC STAT MANU, Vfifth, P947

APA, 2000, DIAGNOSTIC STAT MANU, V4

Ayer T, 2013, COMPUT MATH METHOD M, V2013, DOI 10.1155/2013/832509

Bal VH, 2015, AUTISM RES, V8, P583, DOI 10.1002/aur.1474

Bedford R, 2016, AUTISM RES, V9, P993, DOI 10.1002/aur.1587

Benjamini Y, 2001, ANN STAT, V29, P1165

Bhat AN, 2011, PHYS THER, V91, P1116, DOI 10.2522/ptj.20100294

Biancotto M, 2017, PDMS 2 PEABODY DEV M

Bishop JC, 2018, RES DEV DISABIL, V74, P14, DOI 10.1016/j.ridd.2017.11.002

Bishop SL, 2015, J AUTISM DEV DISORD, V45, P966, DOI 10.1007/s10803-014-2250-3

Brand S, 2015, NEUROPSYCH DIS TREAT, V11, P1911, DOI 10.2147/NDT.S85650

Bremer E, 2015, AUTISM, V19, P980, DOI 10.1177/1362361314557548

Buscema M, 2008, CURR ALZHEIMER RES, V5, P481, DOI 10.2174/156720508785908928

Buscema M, 2017, BEHAV BRAIN SCI, V40, DOI 10.1017/S0140525X1700005X

Buscema M, 2015, PLOS ONE, V10, DOI 10.1371/journal.pone.0126020

Buscema M, 2008, INT J DATA MIN BIOIN, V2, P362, DOI 10.1504/IJDMB.2008.022159

Campione GC, 2016, J AUTISM DEV DISORD, V46, P1985, DOI 10.1007/s10803-016-2732-6

COCCHI M, 2008, BMC PSYCHIATRY S1, V8

Dewey D, 2007, J INT NEUROPSYCH SOC, V13, P246, DOI 10.1017/S1355617707070270

Djemal R, 2017, BIOMED RES INT, V2017, DOI 10.1155/2017/9816591

Dowell LR, 2009, NEUROPSYCHOLOGY, V23, P563, DOI 10.1037/a0015640

Dziuk MA, 2007, DEV MED CHILD NEUROL, V49, P734, DOI 10.1111/j.1469-8749.2007.00734.x

Eggleston JD, 2017, GAIT POSTURE, V55, P162, DOI 10.1016/j.gaitpost.2017.04.026

Esposito G, 2008, PERCEPT MOTOR SKILL, V106, P259, DOI 10.2466/PMS.106.1.259-269

Esposito G, 2011, BRAIN DEV-JPN, V33, P367, DOI 10.1016/j.braindev.2010.07.006

Esposito G, 2009, BRAIN DEV-JPN, V31, P131, DOI 10.1016/j.braindev.2008.04.005

Folio M, 2000, PEABODY DEV MOTOR SC

Fournier KA, 2010, J AUTISM DEV DISORD, V40, P1227, DOI 10.1007/s10803-010-0981-3

Fredman M. L., 1990, Proceedings. 31st Annual Symposium on Foundations of Computer Science (Cat. No.90CH2925-6), P719, DOI 10.1109/FSCS.1990.89594

Fulceri F, 2015, CLIN NEUROPSYCHIATR, V12, P94

Gallese V, 2013, DEV MED CHILD NEUROL, V55, P15, DOI 10.1111/j.1469-8749.2012.04398.x

Garrido D, 2017, AUTISM RES, V10, P1737, DOI 10.1002/aur.1829

Gernsbacher MA, 2008, J CHILD PSYCHOL PSYC, V49, P43, DOI 10.1111/j.1469-7610.2007.01820.x

Gima H, 2018, EXP BRAIN RES, V236, P1139, DOI 10.1007/s00221-018-5202-x

Gironi M, 2013, IMMUN AGEING, V10, DOI 10.1186/1742-4933-10-1

Glazebrook CM, 2006, MOTOR CONTROL, V10, P244, DOI 10.1123/mcj.10.3.244

Gotham K, 2009, J AUTISM DEV DISORD, V39, P693, DOI 10.1007/s10803-008-0674-3

Green D, 2009, DEV MED CHILD NEUROL, V51, P311, DOI 10.1111/j.1469-8749.2008.03242.x

Grossi E, 2018, J DEV ORIG HLTH DIS, V9, P442, DOI [10.1017/S2040174418000211, 10.1017/s2040174418000211]

Grossi E, 2017, COMPUT METH PROG BIO, V142, P73, DOI 10.1016/j.cmpb.2017.02.002

Hedgecock JB, 2018, PHYS THER, V98, P251, DOI 10.1093/ptj/pzy006

Hilton CL, 2012, AUTISM, V16, P430, DOI 10.1177/1362361311423018

Iverson Jana M, 2010, Enfance, V2010, P257

Jasmin E, 2009, J AUTISM DEV DISORD, V39, P231, DOI 10.1007/s10803-008-0617-z

Ketcheson L, 2017, AUTISM, V21, P481, DOI 10.1177/1362361316650611

Kim HU, 2008, PERCEPT MOTOR SKILL, V107, P403, DOI 10.2466/PMS.107.2.403-406

Kopp S, 2010, RES DEV DISABIL, V31, P350, DOI 10.1016/j.ridd.2009.09.017

Krogh A, 2008, NAT BIOTECHNOL, V26, P195, DOI 10.1038/nbt1386

Kruskal J., 1956, P AM MATH SOC, V7, P48, DOI 10.1090/S0002-9939-1956-0078686-7

Lam KSL, 2007, J AUTISM DEV DISORD, V37, P855, DOI 10.1007/s10803-006-0213-z

Landa R, 2006, J CHILD PSYCHOL PSYC, V47, P629, DOI 10.1111/j.1469-7610.2006.01531.x

Landa RJ, 2013, CHILD DEV, V84, P429, DOI 10.1111/j.1467-8624.2012.01870.x

LeBarton ES, 2013, DEVELOPMENTAL SCI, V16, P815, DOI 10.1111/desc.12069

Leekam SR, 2011, PSYCHOL BULL, V137, P562, DOI 10.1037/a0023341

Libertus K, 2016, FRONT PSYCHOL, V7, DOI 10.3389/fpsyg.2016.00475

Libertus K, 2014, CHILD DEV, V85, P2218, DOI 10.1111/cdev.12262

Lloyd M, 2013, AUTISM, V17, P133, DOI 10.1177/1362361311402230

Longuet S, 2012, J AUTISM DEV DISORD, V42, P1446, DOI 10.1007/s10803-011-1383-x

Lopez BR, 2005, J AUTISM DEV DISORD, V35, P445, DOI 10.1007/s10803-005-5035-x

Lord C, 2000, J AUTISM DEV DISORD, V30, P205, DOI 10.1023/A:1005592401947

Luiz D. M., 2006, GRIFFITHS MENTAL DEV

MacDonald M, 2013, ADAPT PHYS ACT Q, V30, P271, DOI 10.1123/apaq.30.3.271

Manning T, 2014, BIOENGINEERED, V5, P80, DOI 10.4161/bioe.26997

Mari M, 2003, PHILOS T ROY SOC B, V358, P393, DOI 10.1098/rstb.2002.1205

May T, 2016, AUST PSYCHOL, V51, P296, DOI 10.1111/ap.12225

Miller M, 2014, BEHAV BRAIN RES, V269, P95, DOI 10.1016/j.bbr.2014.04.011

Minshew NJ, 2004, NEUROLOGY, V63, P2056, DOI 10.1212/01.WNL.0000145771.98657.62

Moseley RL, 2018, CORTEX, V100, P149, DOI 10.1016/j.cortex.2017.11.019

Najafabadi MG, 2018, PEDIATR NEONATOL, V59, P481, DOI 10.1016/j.pedneo.2017.12.005

Narzisi A, 2015, NEUROPSYCH DIS TREAT, V11, P1587, DOI 10.2147/NDT.S81233

Ozonoff S, 2008, J AUTISM DEV DISORD, V38, P644, DOI 10.1007/s10803-007-0430-0

Pan CY, 2017, AUTISM, V21, P190, DOI 10.1177/1362361316633562

Peixoto LA, 2015, GENET MOL RES, V14, P6796, DOI 10.4238/2015.June.18.22

Phagava H, 2008, Georgian Med News, P100

Provost B, 2007, J AUTISM DEV DISORD, V37, P321, DOI 10.1007/s10803-006-0170-6

Purpura G, 2016, MINERVA PEDIATR, V12

Radonovich KJ, 2013, FRONT INTEGR NEUROSC, V7, DOI 10.3389/fnint.2013.00028

Ravizza SM, 2013, DEV PSYCHOPATHOL, V25, P773, DOI 10.1017/S0954579413000163

Roid G.H., 1997, LEITER INT PERFORMAN

Sacrey LAR, 2014, FRONT NEUROL, V5, DOI 10.3389/fneur.2014.00006

Soska KC, 2010, DEV PSYCHOL, V46, P129, DOI 10.1037/a0014618

Srinivasan SM, 2016, INFANT BEHAV DEV, V42, P128, DOI 10.1016/j.infbeh.2015.12.003

Staples KL, 2010, J AUTISM DEV DISORD, V40, P209, DOI 10.1007/s10803-009-0854-9

Stins JF, 2015, GAIT POSTURE, V42, P199, DOI 10.1016/j.gaitpost.2015.05.010

Street ME, 2008, BMC PEDIATR, V8, DOI 10.1186/1471-2431-8-24

Toscano M, 2017, BMC MICROBIOL, V17, DOI 10.1186/s12866-017-1109-0

Travers BG, 2013, J AUTISM DEV DISORD, V43, P1568, DOI 10.1007/s10803-012-1702-x

Uljarevic M, 2017, AUTISM RES, V10, P1163, DOI 10.1002/aur.1763

Vanvuchelen M, 2007, AUTISM, V11, P225, DOI 10.1177/1362361307076846

Wechsler D., 1974, MANUAL WECHSLER SCAL

Whyatt CP, 2012, J AUTISM DEV DISORD, V42, P1799, DOI 10.1007/s10803-011-1421-8

Wilson RB, 2018, CURR OPIN NEUROL, V31, P134, DOI 10.1097/WCO.0000000000000541

Zachor DA, 2010, RES AUTISM SPECT DIS, V4, P438, DOI 10.1016/j.rasd.2009.10.016

Zappella M, 2015, EARLY HUM DEV, V91, P569, DOI 10.1016/j.earlhumdev.2015.07.006

NR 94

TC 20

Z9 22

U1 6

U2 20

PU FRONTIERS MEDIA SA

PI LAUSANNE

PA AVENUE DU TRIBUNAL FEDERAL 34, LAUSANNE, CH-1015, SWITZERLAND

SN 1664-1078

J9 FRONT PSYCHOL

JI Front. Psychol.

PD JAN 9

PY 2019

VL 9

AR 2683

DI 10.3389/fpsyg.2018.02683

PG 12

WC Psychology, Multidisciplinary

WE Social Science Citation Index (SSCI)

SC Psychology

GA HG8YR

UT WOS:000455293400001

PM 30687159

OA Green Published, gold

DA 2022-08-17

ER

PT J

AU Mancini, VO

Althorpe, KE

Chen, W

AF Mancini, Vincent O.

Althorpe, Kathryn E.

Chen, Wai

TI Does motor coordination and sleep difficulties predict peer functioning

in children and adolescents with attention-deficit and hyperactivity

disorder after accounting for existing ADHD symptomology?

SO BRITISH JOURNAL OF DEVELOPMENTAL PSYCHOLOGY

LA English

DT Article

DE attention-deficit and hyperactivity disorder; motor coordination; motor

skills; peer problems; peer relationships

ID DEFICIT/HYPERACTIVITY DISORDER; VICTIMIZATION; PERFORMANCE;

METAANALYSIS; FRIENDSHIPS; AGGRESSION; IMPAIRMENT; COMPETENCE;

CHILDHOOD; REJECTION

AB Children with attention-deficit and hyperactivity disorder (ADHD) are more likely to experience peer problems compared to their non-ADHD peers, though ADHD-specific symptoms only partially explain this association. This study examined whether sleep difficulties and motor coordination problems are additional predictors of peer problems in an ADHD population. An ADHD sample of 72 participants aged 6-14 years (M = 9.86 years,SD = 1.79 years) was evaluated for an association of peer problems with measures of motor coordination, sleep difficulties as well as ADHD and comorbidity symptoms. Hierarchical multiple regression analysis (HMRA) was used to test the current study aims. Motor coordination, but not sleep difficulties, predicted additional variance in peer problems after controlling for inattention, hyperactivity/impulsivity, internalizing problems, oppositionality, and conduct problems. Poor motor coordination predicts peer problems beyond ADHD symptoms. Clinicians seeking to improve peer functioning in children with ADHD should also consider motor coordination difficulties in addition to existing treatment strategies. Statement of contribution What is already known Children with attention-deficit and hyperactivity disorder (ADHD) experience greater peer problems (i.e., making friends, being victimized, participating in play) than their typically developing peers. Previous studies have attributed this association between ADHD and peer problems to the symptoms of ADHD (i.e., inattention and/or hyperactivity) disrupting the typical trajectory of social development. However, quantitative studies have identified that symptoms of ADHD predict only portion of the variance in a child's peer problems - highlighting that there may be other unique factors that contribute to the higher incidence of peer problems typically observed in this population. What this study adds This study tested whether additional theoretically relevant factors could predict levels of peer problems in children with ADHD beyond the primary symptoms of the disorder. Internalizing symptomatology, conduct problems, oppositionality, motor coordination, and sleep difficulties were added to a regression model already including inattention and hyperactivity symptoms. These factors explained 51% of the variability in peer problems. In this sample of 72 ADHD children, the results of the final model highlighted that only motor coordination and conduct problems remained significant predictors of peer problems - highlighting two potentially important target areas for screening and intervention.

C1 [Mancini, Vincent O.; Althorpe, Kathryn E.; Chen, Wai] Complex Attent & Hyperact Disorders CAHDS, Child & Adolescent Mental Hlth Serv, Dept Hlth, Perth, WA USA.

[Mancini, Vincent O.] Australian Coll Appl Psychol, Discipline Psychol Sci, Sydney, NSW, Australia.

[Chen, Wai] Univ Western Australia, Fac Hlth & Med Sci, Paediat, Perth, WA USA.

RP Mancini, VO (通讯作者)，255 Elizabeth St, Sydney, NSW 2000, Australia.

EM vincent.mancini@curtin.edu.au

RI Mancini, Vincent Oreste/AIC-2797-2022

OI Mancini, Vincent/0000-0002-4845-8104

CR Abikoff H, 2004, J AM ACAD CHILD PSY, V43, P820, DOI 10.1097/01.chi.0000128797.91601.1a

American Psychiatric Association, 2013, DIAGN STAT MAN MENT, V5th edition, DOI [10.1176/appi.books.9780890425596, DOI 10.1176/APPI.BOOKS.9780890425596]

Becker SP, 2015, J YOUTH ADOLESCENCE, V44, P239, DOI 10.1007/s10964-014-0248-y

Forner CB, 2017, PSICOTHEMA, V29, P514, DOI 10.7334/psicothema2016.376

Brown T.E., 2009, CURR PSYCHIAT REP, V10, P407, DOI [10.1007/s11920-008-0065, DOI 10.1007/S11920-008-0065]

Bruininks R.H., 2005, BRUININKS OSERETSKY, V2nd

Bruni O, 1996, J SLEEP RES, V5, P251, DOI 10.1111/j.1365-2869.1996.00251.x

Cairney J, 2013, DEV REV, V33, P224, DOI 10.1016/j.dr.2013.07.002

Campbell WN, 2012, PSYCHOL SCHOOLS, V49, P328, DOI 10.1002/pits.21600

Cardoos SL, 2011, J ABNORM CHILD PSYCH, V39, P1035, DOI 10.1007/s10802-011-9517-3

COHEN J, 1992, PSYCHOL BULL, V112, P155, DOI 10.1037/0033-2909.112.1.155

COIE JD, 1991, CHILD DEV, V62, P812, DOI 10.2307/1131179

Conners K.C., 2008, J PSYCHOEDUC ASSESS, DOI DOI 10.1177/0734282909360011

Cordier R, 2009, AUST OCCUP THER J, V56, P332, DOI 10.1111/j.1440-1630.2009.00796.x

Cortese S, 2009, J AM ACAD CHILD PSY, V48, P894, DOI 10.1097/CHI.0b013e3181ac09c9

CRICK NR, 1994, PSYCHOL BULL, V115, P74, DOI 10.1037/0033-2909.115.1.74

Dodge KA, 2003, CHILD DEV, V74, P374, DOI 10.1111/1467-8624.7402004

Fliers EA, 2010, J DEV BEHAV PEDIATR, V31, P35, DOI 10.1097/DBP.0b013e3181c7227e

Fogleman ND, 2018, ADHD-ATTEND DEFICIT, V10, P209, DOI 10.1007/s12402-018-0248-y

Gaub M, 1997, J AM ACAD CHILD PSY, V36, P1036, DOI 10.1097/00004583-199708000-00011

Goulardins JB, 2017, PERCEPT MOTOR SKILL, V124, P425, DOI 10.1177/0031512517690607

Goulardins JB, 2018, J ATTEN DISORD, V22, P796, DOI 10.1177/1087054715580394

Hoza B, 2007, J PEDIATR PSYCHOL, V32, P655, DOI 10.1093/jpepsy/jsm024

Humphrey Jamie L, 2007, J Child Health Care, V11, P248, DOI 10.1177/1367493507079571

Jordan JV, 2013, HANDBOOK OF RESILIENCE IN CHILDREN, SECOND EDITION, P73, DOI 10.1007/978-1-4614-3661-4_5

Kaiser ML, 2015, RES DEV DISABIL, V36, P338, DOI 10.1016/j.ridd.2014.09.023

Lange S.M., 2018, CONT SCH PSYCHOL, V22, P30, DOI [10.1016/j.cmi.2018.11.010.-Available, DOI 10.1007/S40688-017-0122-5, https://doi.org/10.1007/s40688-017-0122-5]

Livesey D, 2011, CHILD CARE HLTH DEV, V37, P581, DOI 10.1111/j.1365-2214.2010.01183.x

Lockwood PL, 2013, CURR BIOL, V23, P901, DOI 10.1016/j.cub.2013.04.018

Lycett K, 2016, J DEV BEHAV PEDIATR, V37, P405, DOI 10.1097/DBP.0000000000000276

Mackenzie G., 2018, JAC, V1, P14, DOI 10.1521/adhd.2018.26.3.1

Mancini V, 2018, INFANT CHILD DEV, V27, DOI 10.1002/icd.2073

Mancini Vincent O, 2019, Sleep Med X, V1, P100006, DOI 10.1016/j.sleepx.2019.100006

Mayes SD, 2009, J PEDIATR PSYCHOL, V34, P328, DOI 10.1093/jpepsy/jsn083

McQuade JD, 2008, DEV DISABIL RES REV, V14, P320, DOI 10.1002/ddrr.35

MERTON RK, 1968, SCIENCE, V159, P56, DOI 10.1126/science.159.3810.56

Missiuna C, 2014, RES DEV DISABIL, V35, P1198, DOI 10.1016/j.ridd.2014.01.007

Mrug S, 2012, J ABNORM CHILD PSYCH, V40, P1013, DOI 10.1007/s10802-012-9610-2

Paavonen EJ, 2010, SLEEP MED, V11, P386, DOI 10.1016/j.sleep.2009.09.009

Papadopoulos N, 2019, BEHAV SLEEP MED, V17, P646, DOI 10.1080/15402002.2018.1443455

PARKER JG, 1987, PSYCHOL BULL, V102, P357, DOI 10.1037/0033-2909.102.3.357

Pelham WE, 1982, ADV LEARN BEHAV DISA, V1, P365

Polanczyk G, 2007, AM J PSYCHIAT, V164, P942, DOI 10.1176/appi.ajp.164.6.942

Rigoli D, 2012, PEDIATRICS, V129, pE892, DOI 10.1542/peds.2011-1237

Rosen PJ, 2014, SOC DEV, V23, P288, DOI 10.1111/sode.12046

Rubin KH., 2007, HDB CHILD PSYCHOL, V1, P3

Sanchez ZE, 2011, J ATTEN DISORD, V70, P180

Schei J, 2015, BMC PSYCHIATRY, V15, DOI 10.1186/s12888-015-0491-0

Skrzypiec G, 2012, EMOT BEHAV DIFFIC, V17, P259, DOI 10.1080/13632752.2012.704312

Tan SK, 2001, ADAPT PHYS ACT Q, V18, P168, DOI 10.1123/apaq.18.2.168

Uchida M, 2018, J ATTEN DISORD, V22, P523, DOI 10.1177/1087054715604360

Vaughn BE, 2015, BEHAV SLEEP MED, V13, P92, DOI 10.1080/15402002.2013.845778

Vincon S, 2017, HUM MOVEMENT SCI, V53, P45, DOI 10.1016/j.humov.2016.10.005

Waschbusch DA, 2008, AGGRESSIVE BEHAV, V34, P139, DOI 10.1002/ab.20224

WIATER AH, 2005, SOMNOLOGIE, V9, P210, DOI DOI 10.1111/J.1439-054X.2005.00073.X

Wiener J, 2009, PSYCHOL SCHOOLS, V46, P116, DOI 10.1002/pits.20358

Wiggs L, 2016, FRONT PEDIATR, V4, DOI 10.3389/fped.2016.00081

Williams KE, 2016, BRIT J EDUC PSYCHOL, V86, P331, DOI 10.1111/bjep.12109

NR 58

TC 2

Z9 2

U1 0

U2 10

PU WILEY

PI HOBOKEN

PA 111 RIVER ST, HOBOKEN 07030-5774, NJ USA

SN 0261-510X

EI 2044-835X

J9 BRIT J DEV PSYCHOL

JI Br. J. Dev. Psychol.

PD SEP

PY 2020

VL 38

IS 3

BP 442

EP 457

DI 10.1111/bjdp.12327

EA MAR 2020

PG 16

WC Psychology, Developmental

WE Social Science Citation Index (SSCI)

SC Psychology

GA NB6BQ

UT WOS:000563143000001

PM 32167193

DA 2022-08-17

ER

PT J

AU Reynolds, JE

Kerrigan, S

Elliott, C

Lay, BS

Licari, MK

AF Reynolds, Jess E.

Kerrigan, Sophie

Elliott, Catherine

Lay, Brendan S.

Licari, Melissa K.

TI Poor Imitative Performance of Unlearned Gestures in Children with

Probable Developmental Coordination Disorder

SO JOURNAL OF MOTOR BEHAVIOR

LA English

DT Article

DE developmental coordination disorder; DCD; imitation; mirror neuron

system; praxis; sensory integration and praxis tests; SIPT

ID MIRROR NEURON SYSTEM; MOTOR IMAGERY; INTERNAL REPRESENTATION; LANGUAGE

IMPAIRMENT; FEMALE ADVANTAGE; WORKING-MEMORY; DEFICIT; SKILLS; MOVEMENT;

PRAXIS

AB It has been hypothesized that deficits in imitation, linked to abnormal functioning of the mirror neuron system (MNS), may contribute to the motor impairments associated with developmental coordination disorder (DCD). The authors aimed to examine imitation of complex novel postures and sequences of gestures in children with and without probable DCD (pDCD), using the postural praxis and sequencing praxis subtests of the Sensory Integration and Praxis Tests (Ayres, 1989). Participants were 29 boys with pDCD between 6.08 and 13.33years old, and 29 group age-matched typically developing boys between 6.08 and 13.83years old. Responses of children with pDCD on both imitation tasks were less accurate than controls, with group differences more apparent with increasing task complexity. Furthermore, as a group, children with pDCD were slower and had a higher number of non-mirror-imitated responses. There was considerable variability within the pDCD group, with some children displaying imitation scores within the normative range. Given the importance of imitation and visual learning for motor development, the difficulties in imitation displayed by some children with pDCD have the potential to impact on movement acquisition. Interventions to target imitation may be beneficial for these children. The results show that children with pDCD had difficulty imitating complex novel postures, children with pDCD had difficulty imitating gesture sequences, children with pDCD had slower responses than controls, group differences in imitation performance increased with task complexity, and not all children with pDCD displayed imitation deficits.

C1 [Reynolds, Jess E.; Kerrigan, Sophie; Lay, Brendan S.; Licari, Melissa K.] Univ Western Australia, Sch Sport Sci Exercise & Hlth, M408,35 Stirling Highway, Crawley, WA 6009, Australia.

[Elliott, Catherine] Curtin Univ, Sch Occupat Therapy & Social Work, Perth, WA, Australia.

[Elliott, Catherine] Child & Adolescent Hlth Serv, Perth, WA, Australia.

RP Reynolds, JE (通讯作者)，Univ Western Australia, Sch Sport Sci Exercise & Hlth, M408,35 Stirling Highway, Crawley, WA 6009, Australia.

EM jessica.reynolds@research.uwa.edu.au

RI ; Elliott, Catherine/B-6718-2014

OI Lay, Brendan/0000-0002-8888-5987; Elliott,

Catherine/0000-0002-5324-8216; Licari, Melissa/0000-0003-3705-5323

CR Alloway TP, 2007, J EXP CHILD PSYCHOL, V96, P20, DOI 10.1016/j.jecp.2006.07.002

American Psychiatric Association, 2013, DIAGN STAT MAN MENT, V5th edition, DOI [10.1176/appi.books.9780890425596, DOI 10.1176/APPI.BOOKS.9780890425596]

Arbib MA, 2000, NEURAL NETWORKS, V13, P975, DOI 10.1016/S0893-6080(00)00070-8

Ayres A.J., 1989, SENSORY INTEGRATION, V1st

AYRES AJ, 1965, PERCEPT MOTOR SKILL, V20, P335, DOI 10.2466/pms.1965.20.2.335

Barbarulo AM, 2008, NEUROCASE, V14, P293, DOI 10.1080/13554790802363688

Berges J., 1965, CLIN DEV MED

Brown-Lum M., 2015, CURR DEV DISORD REP, V2, P131, DOI [10.1007/s40474-015-0046-6, DOI 10.1007/S40474-015-0046-6]

Buccino G, 2006, COGN BEHAV NEUROL, V19, P55, DOI 10.1097/00146965-200603000-00007

Buccino G, 2012, DEV MED CHILD NEUROL, V54, P822, DOI 10.1111/j.1469-8749.2012.04334.x

Bussing R, 2008, ASSESSMENT, V15, P317, DOI 10.1177/1073191107313888

Cacola P, 2014, J CLIN EXP NEUROPSYC, V36, P596, DOI 10.1080/13803395.2014.918092

Chipman K, 2007, DEV NEUROPSYCHOL, V31, P137, DOI 10.1080/87565640701190692

Chipman K, 2006, NEUROPSYCHOLOGIA, V44, P2315, DOI 10.1016/j.neuropsychologia.2006.05.002

DEWEY D, 1991, DEV NEUROPSYCHOL, V7, P197, DOI 10.1080/87565649109540487

DEWEY D, 1992, DEV NEUROPSYCHOL, V8, P367, DOI 10.1080/87565649209540532

DEWEY D, 1993, BRAIN COGNITION, V23, P203, DOI 10.1006/brcg.1993.1055

Dewey D, 2007, J INT NEUROPSYCH SOC, V13, P246, DOI 10.1017/S1355617707070270

Elbasan B, 2012, ITAL J PEDIATR, V38, DOI 10.1186/1824-7288-38-14

Filipcic T, 2008, ACTA GYMNICA, V38, P25

Gomez A, 2015, NEUROPSYCHOLOGIA, V79, P272, DOI 10.1016/j.neuropsychologia.2015.09.032

Goyen TA, 2011, EARLY HUM DEV, V87, P489, DOI 10.1016/j.earlhumdev.2011.04.002

Green D, 2002, J CHILD PSYCHOL PSYC, V43, P655, DOI 10.1111/1469-7610.00054

Hayes SJ, 2010, EXP BRAIN RES, V204, P199, DOI 10.1007/s00221-010-2303-6

Heiser M, 2003, EUR J NEUROSCI, V17, P1123, DOI 10.1046/j.1460-9568.2003.02530.x

HENDERSON L, 1992, J CHILD PSYCHOL PSYC, V33, P895, DOI 10.1111/j.1469-7610.1992.tb01963.x

Henderson S., 2007, MOVEMENT ASSESSMENT

Hill EL, 1998, HUM MOVEMENT SCI, V17, P655, DOI 10.1016/S0167-9457(98)00017-7

Hill EL, 1998, DEV MED CHILD NEUROL, V40, P388

Iacoboni M, 2005, CURR OPIN NEUROBIOL, V15, P632, DOI 10.1016/j.conb.2005.10.010

Iacoboni M, 2006, NAT REV NEUROSCI, V7, P942, DOI 10.1038/nrn2024

Kagerer FA, 2006, HUM MOVEMENT SCI, V25, P622, DOI 10.1016/j.humov.2006.06.003

Kagerer FA, 2004, MOTOR CONTROL, V8, P450, DOI 10.1123/mcj.8.4.450

Kammers MPM, 2009, J COGNITIVE NEUROSCI, V21, P1311, DOI 10.1162/jocn.2009.21095

Laszlo J. I., 1989, BRIT J DEV PSYCHOL, V7, P251

Lloyd-Fox S, 2015, CEREB CORTEX, V25, P289, DOI 10.1093/cercor/bht207

Mehta UM, 2015, ASIAN J PSYCHIATR, V17, P71, DOI 10.1016/j.ajp.2015.06.014

MELTZOFF AN, 1977, SCIENCE, V198, P75, DOI 10.1126/science.198.4312.75

Meltzoff AN, 2013, ACTION SCIENCE: FOUNDATIONS OF AN EMERGING DISCIPLINE, P281

Noten M, 2014, RES DEV DISABIL, V35, P1152, DOI 10.1016/j.ridd.2014.01.026

Ozbic M, 2010, KINESIOLOGY, V42, P44

Piek JP, 2007, DEV MED CHILD NEUROL, V49, P678, DOI 10.1111/j.1469-8749.2007.00678.x

Power E, 2010, J CLIN EXP NEUROPSYC, V32, P1, DOI 10.1080/13803390902791646

Reynolds JE, 2015, INT J DEV NEUROSCI, V47, P309, DOI 10.1016/j.ijdevneu.2015.10.003

Reynolds JE, 2015, RES DEV DISABIL, V47, P234, DOI 10.1016/j.ridd.2015.09.015

Reynolds JE, 2015, HUM MOVEMENT SCI, V44, P287, DOI 10.1016/j.humov.2015.09.012

Rizzolatti G, 2004, ANNU REV NEUROSCI, V27, P169, DOI 10.1146/annurev.neuro.27.070203.144230

Schopler E., 1988, CHILDHOOD AUTISM RAT

Sigmundsson H, 1999, BEHAV BRAIN RES, V102, P129, DOI 10.1016/S0166-4328(99)00009-1

Sinani C, 2011, RES DEV DISABIL, V32, P1270, DOI 10.1016/j.ridd.2011.01.030

SMYTH TR, 1994, CHILD CARE HLTH DEV, V20, P27, DOI 10.1111/j.1365-2214.1994.tb00372.x

Steinman KJ, 2010, J CHILD NEUROL, V25, P71, DOI 10.1177/0883073809342591

Tomasello M., 2005, PERSPECTIVES IMITATI, V2, P133, DOI DOI 10.7551/MITPRESS/5331.003.0007

Tsai CL, 2012, DEV MED CHILD NEUROL, V54, P1114, DOI [10.1111/j.1469-8749.2012.04427.x, 10.1111/j.1469-8749.2012.04408.x]

Uddin LQ, 2006, SOC COGN AFFECT NEUR, V1, P65, DOI 10.1093/scan/nsl003

Werner JM, 2012, J BEHAV BRAIN SCI, V2, P258, DOI [10.4236/jbbs.2012.22029, DOI 10.4236/JBBS.2012.22029]

Williams JHG, 2001, NEUROSCI BIOBEHAV R, V25, P287, DOI 10.1016/S0149-7634(01)00014-8

Wilmut K, 2007, DISABIL REHABIL, V29, P47, DOI 10.1080/09638280600947765

Wilson PH, 2013, DEV MED CHILD NEUROL, V55, P217, DOI 10.1111/j.1469-8749.2012.04436.x

Wilson PH, 2004, DEV MED CHILD NEUROL, V46, P754, DOI 10.1017/S001216220400129X

World Health Organization (WHO), 2010, INT STAT CLASS DIS R

Zoia S, 2005, DEV NEUROPSYCHOL, V27, P257, DOI 10.1207/s15326942dn2702_4

Zoia S, 2002, DEV MED CHILD NEUROL, V44, P699

NR 63

TC 12

Z9 12

U1 0

U2 11

PU ROUTLEDGE JOURNALS, TAYLOR & FRANCIS LTD

PI ABINGDON

PA 2-4 PARK SQUARE, MILTON PARK, ABINGDON OX14 4RN, OXON, ENGLAND

SN 0022-2895

EI 1940-1027

J9 J MOTOR BEHAV

JI J. Mot. Behav.

PY 2017

VL 49

IS 4

BP 378

EP 387

DI 10.1080/00222895.2016.1219305

PG 10

WC Neurosciences; Psychology; Psychology, Experimental; Sport Sciences

WE Science Citation Index Expanded (SCI-EXPANDED); Social Science Citation Index (SSCI)

SC Neurosciences & Neurology; Psychology; Sport Sciences

GA EZ6DT

UT WOS:000404809300003

PM 27726691

DA 2022-08-17

ER

PT J

AU Jascenoka, J

Walter, F

AF Jascenoka, Julia

Walter, Franziska

TI Cognitive Profiles of Children with Low Motor Performance: A

Contribution to the Validation of the WPPSI-IV

SO CHILDREN-BASEL

LA English

DT Article

DE movement abilities; Developmental Coordination Disorder (DCD); cognitive

profiles; WPPSI-IV; LoMo 3-6; intelligence

ID DEVELOPMENTAL COORDINATION DISORDER; PRESCHOOL-CHILDREN; SKILLS;

PREVALENCE; KNOWLEDGE; DIFFICULTIES; IMPAIRMENTS

AB (1) Background: Developmental Coordination Disorder (DCD) is a common developmental disorder of preschool age. Children often show cognitive deficits in addition to motor problems. Various studies point in particular to problems in visual perception, working memory and processing speed. In this context, it is investigated whether the Wechsler Preschool and Primary Scale-IV (WPPSI-IV) is a suitable instrument for mapping these deficits in a valid and economical way. (2) Methods: The WPPSI-IV profiles of children with DCD (n = 12), below-average motor performance (n = 22) and a control group (n = 32) were compared. (3) Results: Children with DCD achieved significantly poorer test performance in the primary indices Verbal Comprehension, Visual Spatial, Processing Speed and Full Scale compared to a control group. Children with below-average motor skills, on the other hand, do not differ from the children in the control group. (4) Conclusions: The WPPSI-IV is a suitable instrument for diagnosing cognitive deficits in the context of DCD. The Fluid Reasoning and Verbal Comprehension indices should be used as a supplement to assess cognitive performance levels.

C1 [Jascenoka, Julia] Helmut Schmidt Univ, Univ Fed Armed Forces, Dept Educ Psychol, D-22043 Hamburg, Germany.

[Walter, Franziska] Med Sch Hamburg, Dept Med, D-20457 Hamburg, Germany.

RP Jascenoka, J (通讯作者)，Helmut Schmidt Univ, Univ Fed Armed Forces, Dept Educ Psychol, D-22043 Hamburg, Germany.

EM jascenoka@hsu-hh.de; franziska.walter@medicalschool-hamburg.de

OI Walter, Franziska/0000-0002-5022-1122

CR American Psychiatric Association, 2013, DIAGN STAT MAN MENT, V5, DOI 10.1176/appi.books.9780890425596

Asonitou K, 2012, RES DEV DISABIL, V33, P996, DOI 10.1016/j.ridd.2012.01.008

Blank R, 2019, DEV MED CHILD NEUROL, V61, P242, DOI 10.1111/dmcn.14132

Caçola Priscila, 2019, Motriz: rev. educ. fis., V25, pe101923, DOI 10.1590/s1980-6574201900020001

Cacola P, 2014, LEARN DISABIL-MULTI, V20, P98

Chen IC, 2013, RES DEV DISABIL, V34, P687, DOI 10.1016/j.ridd.2012.09.012

Cheng CH, 2014, RES DEV DISABIL, V35, P2172, DOI 10.1016/j.ridd.2014.05.009

COHEN J, 1992, PSYCHOL BULL, V112, P155, DOI 10.1037/0033-2909.112.1.155

Cohen J., 1977, STAT POWER ANAL BEHA, DOI DOI 10.4324/9780203771587

Daseking M, 2018, KINDH ENTWICKL, V27, P127, DOI 10.1026/0942-5403/a000252

De Milander M, 2016, S AFR J RES SPORT PH, V38, P49

Delgado-Lobete L, 2019, RES DEV DISABIL, V86, P31, DOI 10.1016/j.ridd.2019.01.004

Dewey D, 2016, CURR DEV DISORD REP, V3, P161, DOI [10.1007/s40474-016-0086-6, DOI 10.1007/S40474-016-0086-6]

Eickhorst A, 2016, BUNDESGESUNDHEITSBLA, V59, P1271, DOI 10.1007/s00103-016-2422-8

Henderson S. E., 2007, MOVEMENT ASSESSMENT

Hua J, 2022, FRONT PSYCHIATRY, V13, DOI 10.3389/fpsyt.2022.809181

Jascenoka J., 2018, LEISTUNGSINVENTAR OB

Jascenoka J, 2018, KINDH ENTWICKL, V27, P142, DOI 10.1026/0942-5403/a000254

Jascenoka J, 2018, KINDH ENTWICKL, V27, P14, DOI 10.1026/0942-5403/a000241

Jascenoka J, 2015, PRAX KINDERPSYCHOL K, V64, P117, DOI 10.13109/prkk.2015.64.2.117

Kastner J, 2011, KINDH ENTWICKL, V20, P173, DOI 10.1026/0942-5403/a000054

Leonard HC, 2015, DEV NEUROPSYCHOL, V40, P201, DOI 10.1080/87565641.2014.997933

Lingam R, 2009, PEDIATRICS, V123, pE693, DOI 10.1542/peds.2008-1770

Michel E, 2011, KINDH ENTWICKL, V20, P49, DOI 10.1026/0942-5403/a000024

Petermann F., 2011, WECHSLER INTELLIGENC

Prunty M, 2016, HUM MOVEMENT SCI, V49, P54, DOI 10.1016/j.humov.2016.06.003

Schroeder M A, 1990, West J Nurs Res, V12, P175, DOI 10.1177/019394599001200204

Smith M, 2019, J FOOT ANKLE RES, V12, DOI 10.1186/s13047-019-0353-y

Stollhoff K., 2018, P DIATRIE, V30, P56, DOI [10.1007/s15014-018-1538-8, DOI 10.1007/S15014-018-1538-8]

Alagesan J, 2020, INDIAN J PEDIATR, V87, P454, DOI 10.1007/s12098-020-03191-5

Sumner E, 2016, RES DEV DISABIL, V56, P10, DOI 10.1016/j.ridd.2016.05.012

Tsai CL, 2008, HUM MOVEMENT SCI, V27, P649, DOI 10.1016/j.humov.2007.10.002

van der Fels IMJ, 2015, J SCI MED SPORT, V18, P697, DOI 10.1016/j.jsams.2014.09.007

Wechsel D., 2018, WECHSLER PRESCHOOL P

Wechsler D., 2012, WECHSLER PRESCHOOL P, Vfourth

Wechsler D., 2002, WECHSLER PRESCHOOL P

Wilson BN, 2013, CHILD CARE HLTH DEV, V39, P296, DOI 10.1111/j.1365-2214.2012.01403.x

Zwicker JG, 2012, EUR J PAEDIATR NEURO, V16, P573, DOI 10.1016/j.ejpn.2012.05.005

NR 38

TC 0

Z9 0

U1 0

U2 0

PU MDPI

PI BASEL

PA ST ALBAN-ANLAGE 66, CH-4052 BASEL, SWITZERLAND

EI 2227-9067

J9 CHILDREN-BASEL

JI Children-Basel

PD MAY

PY 2022

VL 9

IS 5

AR 619

DI 10.3390/children9050619

PG 15

WC Pediatrics

WE Science Citation Index Expanded (SCI-EXPANDED)

SC Pediatrics

GA 1O9YG

UT WOS:000801677200001

PM 35626796

OA gold, Green Published

DA 2022-08-17

ER

PT J

AU Brown-Lum, M

Zwicker, JG

AF Brown-Lum, Meisan

Zwicker, Jill G.

TI Neuroimaging and Occupational Therapy: Bridging the Gap to Advance

Rehabilitation in Developmental Coordination Disorder

SO JOURNAL OF MOTOR BEHAVIOR

LA English

DT Review

DE developmental coordination disorder; magnetic resonance imaging;

occupational therapy; rehabilitation

ID LOW-BIRTH-WEIGHT; WHITE-MATTER; CEREBRAL-PALSY; MOTOR CONTROL;

BRAIN-DEVELOPMENT; NEONATAL PAIN; CHILDREN; DIFFUSION; CONNECTIVITY;

ATTENTION

AB Developmental coordination disorder (DCD) is a neurodevelopmental disorder characterized by poor motor skills that interfere with a child's ability to perform everyday activities. Little is known about the neural mechanisms that implicate DCD, making it difficult to understand why children with DCD struggle to learn motor skills and selecting the best intervention to optimize function. Neuroimaging studies that utilize magnetic resonance imaging techniques have the capacity to play a critical role in helping to guide clinicians to optimize functional outcomes of children with DCD using evidence-based rehabilitation interventions. The authors' goal is to describe how neuroimaging research can be applied to occupational therapy and rehabilitation sciences by highlighting projects that are at the forefront of the field and elucidate future directions.

C1 [Brown-Lum, Meisan; Zwicker, Jill G.] Univ British Columbia, Dept Occupat Sci & Occupat Therapy, Vancouver, BC, Canada.

[Brown-Lum, Meisan; Zwicker, Jill G.] BC Childrens Hosp, Res Inst, K3-180,4480 Oak St, Vancouver, BC V6H 3V4, Canada.

[Zwicker, Jill G.] Univ British Columbia, Dept Pediat, Vancouver, BC, Canada.

[Zwicker, Jill G.] Sunny Hill Hlth Ctr Children, Vancouver, BC, Canada.

[Zwicker, Jill G.] CanChild Ctr Childhood Disabil Res, Hamilton, ON, Canada.

RP Zwicker, JG (通讯作者)，BC Childrens Hosp, Res Inst, K3-180,4480 Oak St, Vancouver, BC V6H 3V4, Canada.

EM jill.zwicker@ubc.ca

OI Zwicker, Jill/0000-0002-2986-6938

FU BC Children's Hospital Research Institute; Michael Smith Foundation for

Health Research; Canadian Child Health Clinician Scientist Program;

Sunny Hill Foundation; Canadian Institutes of Health Research

FX Meisan Brown-Lum is funded by the BC Children's Hospital Research

Institute. Dr. Zwicker is funded by the Michael Smith Foundation for

Health Research, Canadian Child Health Clinician Scientist Program,

Sunny Hill Foundation, BC Children's Hospital Research Institute, and

Canadian Institutes of Health Research.

CR Adams ILJ, 2014, NEUROSCI BIOBEHAV R, V47, P225, DOI 10.1016/j.neubiorev.2014.08.011

American Psychiatric Association, 2013, DIAGN STAT MAN MENT, V5th edition, DOI [10.1176/appi.books.9780890425596, DOI 10.1176/APPI.BOOKS.9780890425596]

[Anonymous], 2011, ENCY CHILD BEHAV DEV, DOI DOI 10.1007/978-0-387-79061-9_834

Armstrong D, 2012, BRIT J OCCUP THER, V75, P532, DOI 10.4276/030802212X13548955545413

Barkovich AJ, 2011, PEDIAT NEUROIMAGING

Beaulieu C, 2005, NEUROIMAGE, V25, P1266, DOI 10.1016/j.neuroimage.2004.12.053

Bleyenheuft Y, 2015, RES DEV DISABIL, V43-44, P136, DOI 10.1016/j.ridd.2015.06.014

Blumenfeld-Katzir T, 2011, PLOS ONE, V6, DOI 10.1371/journal.pone.0020678

Boyd LA, 2007, PHYS THER, V87, P684, DOI 10.2522/ptj.20060164

Brown-Lum M., 2015, CURR DEV DISORD REP, V2, P131, DOI [10.1007/s40474-015-0046-6, DOI 10.1007/S40474-015-0046-6]

Brummelte S, 2012, ANN NEUROL, V71, P385, DOI 10.1002/ana.22267

Caeyenberghs K, 2016, DEVELOPMENTAL SCI, V19, P599, DOI 10.1111/desc.12424

Cantell MH, 2003, HUM MOVEMENT SCI, V22, P413, DOI 10.1016/j.humov.2003.09.002

Cascio CJ, 2007, J AM ACAD CHILD PSY, V46, P213, DOI 10.1097/01.chi.0000246064.93200.e8

Chavhan G. B., 2009, RADIOGRAPHICS, V29, P1422

Ciccarelli O, 2008, LANCET NEUROL, V7, P715, DOI 10.1016/S1474-4422(08)70163-7

Cousins M, 2003, HUM MOVEMENT SCI, V22, P433, DOI 10.1016/j.humov.2003.09.003

Debrabant J, 2016, J PEDIATR-US, V169, P21, DOI 10.1016/j.jpeds.2015.09.069

Debrabant J, 2013, RES DEV DISABIL, V34, P1478, DOI 10.1016/j.ridd.2013.02.008

Dewey D., 2002, J INT NEUROPSYCH SOC, V13, P246

Dubois J, 2014, NEUROSCIENCE, V276, P48, DOI 10.1016/j.neuroscience.2013.12.044

Edwards J, 2011, J DEV BEHAV PEDIATR, V32, P678, DOI 10.1097/DBP.0b013e31822a396a

Englander ZA, 2015, NEUROIMAGE-CLIN, V7, P315, DOI 10.1016/j.nicl.2015.01.002

Gebauer D, 2012, BRAIN STRUCT FUNCT, V217, P747, DOI 10.1007/s00429-011-0371-4

Goulardins JB, 2015, BEHAV BRAIN RES, V292, P484, DOI 10.1016/j.bbr.2015.07.009

Grunau RE, 2009, PAIN, V143, P138, DOI 10.1016/j.pain.2009.02.014

Harris SR, 2015, CAN MED ASSOC J, V187, P659, DOI 10.1503/cmaj.140994

Heeger DJ, 2002, NAT REV NEUROSCI, V3, P142, DOI 10.1038/nrn730

HELLGREN L, 1993, DEV MED CHILD NEUROL, V35, P881, DOI 10.1111/j.1469-8749.1993.tb11565.x

Henderson S., 2007, MOVEMENT ASSESSMENT

Inguaggiato E, 2013, NEURAL PLAST, V2013, DOI 10.1155/2013/356275

Jones DK, 2013, NEUROIMAGE, V73, P239, DOI 10.1016/j.neuroimage.2012.06.081

Kadesjo B, 1998, DEV MED CHILD NEUROL, V40, P796

Kaplan B. J., 1997, J INT NEUROPSYCH SOC, V3, P54

Kaplan BJ, 1998, HUM MOVEMENT SCI, V17, P471, DOI 10.1016/S0167-9457(98)00010-4

Kashiwagi M, 2009, NEUROREPORT, V20, P1319, DOI 10.1097/WNR.0b013e32832f4d87

Kawato M, 1999, CURR OPIN NEUROBIOL, V9, P718, DOI 10.1016/S0959-4388(99)00028-8

Keller TA, 2009, NEURON, V64, P624, DOI 10.1016/j.neuron.2009.10.018

Kilner James M, 2007, Cogn Process, V8, P159, DOI 10.1007/s10339-007-0170-2

Kim JH, 2015, NEURAL REGEN RES, V10, P624, DOI 10.4103/1673-5374.155438

Kimberley TJ, 2007, PHYS THER, V87, P670, DOI 10.2522/ptj.20060149

Krafft CE, 2014, OBESITY, V22, P232, DOI 10.1002/oby.20518

Langevin LM, 2015, DEV MED CHILD NEUROL, V57, P257, DOI 10.1111/dmcn.12561

Langevin LM, 2014, J PEDIATR-US, V164, P1157, DOI 10.1016/j.jpeds.2014.01.018

Lebel C, 2013, BRAIN LANG, V125, P215, DOI 10.1016/j.bandl.2012.10.009

Licari MK, 2015, EXP BRAIN RES, V233, P1703, DOI 10.1007/s00221-015-4243-7

Lloyd W., 2010, P INT SOC MAGNETIC M, P18

Marien P, 2010, CEREBELLUM, V9, P405, DOI 10.1007/s12311-010-0177-6

Mattay V., 1999, EUROPEAN J RADIOLOGY, V30

McCarron L. T., 1997, MAND MCCARRON ASSESS

McLeod KR, 2014, NEUROIMAGE-CLIN, V4, P566, DOI 10.1016/j.nicl.2014.03.010

Miall RC, 2003, NEUROREPORT, V14, P2135, DOI 10.1097/00001756-200312020-00001

Miller LT, 2001, HUM MOVEMENT SCI, V20, P183, DOI 10.1016/S0167-9457(01)00034-3

Minshew NJ, 2007, ARCH NEUROL-CHICAGO, V64, P945, DOI 10.1001/archneur.64.7.945

Missiuna C, 2006, CAN MED ASSOC J, V175, P471, DOI 10.1503/cmaj.051202

Missiuna Cheryl, 2003, Pediatr Phys Ther, V15, P32, DOI 10.1097/01.PEP.0000051695.47004.BF

Mukherjee P, 2002, AM J NEURORADIOL, V23, P1445

Nicolson RI, 1999, LANCET, V353, P1662, DOI 10.1016/S0140-6736(98)09165-X

Partridge SC, 2004, NEUROIMAGE, V22, P1302, DOI 10.1016/j.neuroimage.2004.02.038

Pitcher TM, 2003, DEV MED CHILD NEUROL, V45, P525, DOI 10.1111/j.1469-8749.2003.tb00952.x

POLATAJKO H. J., 2006, SEMINARS PEDIAT NEUR, V12, P250

Polatajko Helene J., 2001, Physical and Occupational Therapy in Pediatrics, V20, P83, DOI 10.1300/J006v20n02_06

Polatajko Helene J., 2001, Physical and Occupational Therapy in Pediatrics, V20, P107, DOI 10.1300/J006v20n02_07

Polatajko HJ, 1999, CLIN DEV MED, P119

Prunty MM, 2014, RES DEV DISABIL, V35, P2894, DOI 10.1016/j.ridd.2014.07.033

Querne L, 2008, BRAIN RES, V1244, P89, DOI 10.1016/j.brainres.2008.07.066

Ranger M, 2015, J PEDIATR-US, V167, P292, DOI 10.1016/j.jpeds.2015.04.055

Reynolds JE, 2015, INT J DEV NEUROSCI, V47, P309, DOI 10.1016/j.ijdevneu.2015.10.003

Salat DH, 2005, NEUROBIOL AGING, V26, P1215, DOI 10.1016/j.neurobiolaging.2004.09.017

Saling LL, 2007, BRAIN RES BULL, V73, P1, DOI 10.1016/j.brainresbull.2007.02.009

Scheck SM, 2012, DEV MED CHILD NEUROL, V54, P684, DOI 10.1111/j.1469-8749.2012.04332.x

Smith GC, 2011, ANN NEUROL, V70, P541, DOI 10.1002/ana.22545

Smits-Engelsman BCM, 2013, DEV MED CHILD NEUROL, V55, P229, DOI 10.1111/dmcn.12008

Song SK, 2002, NEUROIMAGE, V17, P1429, DOI 10.1006/nimg.2002.1267

Sparks BF, 2002, NEUROLOGY, V59, P184, DOI 10.1212/WNL.59.2.184

Sterling C, 2013, PEDIATRICS, V131, pE1664, DOI 10.1542/peds.2012-2051

Tam E. W., 2011, SCI TRANSL MED, V19, P245

Trivedi R, 2008, PEDIATR NEUROL, V39, P341, DOI 10.1016/j.pediatrneurol.2008.07.012

Tsiotra GD, 2006, J ADOLESCENT HEALTH, V39, P125, DOI 10.1016/j.jadohealth.2005.07.011

Ugurbil K, 1999, PHILOS T R SOC B, V354, P1195, DOI 10.1098/rstb.1999.0474

Visser J, 2003, HUM MOVEMENT SCI, V22, P479, DOI 10.1016/j.humov.2003.09.005

Werner JM, 2012, J BEHAV BRAIN SCI, V2, P258, DOI [10.4236/jbbs.2012.22029, DOI 10.4236/JBBS.2012.22029]

Williams DL, 2007, NEUROIMAG CLIN N AM, V17, P495, DOI 10.1016/j.nic.2007.07.007

Wilson PH, 2013, DEV MED CHILD NEUROL, V55, P217, DOI 10.1111/j.1469-8749.2012.04436.x

Wilson PH, 2002, J CHILD NEUROL, V17, P491, DOI 10.1177/088307380201700704

Yoshida S, 2010, DEV MED CHILD NEUROL, V52, P935, DOI 10.1111/j.1469-8749.2010.03669.x

Zatorre RJ, 2012, NAT NEUROSCI, V15, P528, DOI 10.1038/nn.3045

Zwicker JG, 2013, CHILD CARE HLTH DEV, V39, P562, DOI 10.1111/j.1365-2214.2012.01379.x

Zwicker JG, 2016, J PEDIATR-US, V172, P81, DOI 10.1016/j.jpeds.2015.12.024

Zwicker JG, 2015, PHYS OCCUP THER PEDI, V35, P163, DOI 10.3109/01942638.2014.957431

Zwicker JG, 2013, PEDIATR NEUROL, V48, P123, DOI 10.1016/j.pediatrneurol.2012.10.016

Zwicker JG, 2012, EUR J PAEDIATR NEURO, V16, P573, DOI 10.1016/j.ejpn.2012.05.005

Zwicker JG, 2012, PEDIATR NEUROL, V46, P162, DOI 10.1016/j.pediatrneurol.2011.12.007

Zwicker JG, 2011, INT J DEV NEUROSCI, V29, P145, DOI 10.1016/j.ijdevneu.2010.12.002

Zwicker JG, 2010, PEDIATRICS, V126, pE678, DOI 10.1542/peds.2010-0059

Zwicker JG, 2009, J CHILD NEUROL, V24, P1273, DOI 10.1177/0883073809333537

Zwicker JG, 2013, ARCH DIS CHILD, V98, P118, DOI 10.1136/archdischild-2012-302268

NR 97

TC 7

Z9 7

U1 1

U2 18

PU ROUTLEDGE JOURNALS, TAYLOR & FRANCIS LTD

PI ABINGDON

PA 2-4 PARK SQUARE, MILTON PARK, ABINGDON OX14 4RN, OXON, ENGLAND

SN 0022-2895

EI 1940-1027

J9 J MOTOR BEHAV

JI J. Mot. Behav.

PD JAN-FEB

PY 2017

VL 49

IS 1

BP 98

EP 110

DI 10.1080/00222895.2016.1271295

PG 13

WC Neurosciences; Psychology; Psychology, Experimental; Sport Sciences

WE Science Citation Index Expanded (SCI-EXPANDED); Social Science Citation Index (SSCI)

SC Neurosciences & Neurology; Psychology; Sport Sciences

GA EN5EN

UT WOS:000396028500011

PM 28166485

DA 2022-08-17

ER

PT J

AU Alesi, M

Pecoraro, D

Pepi, A

AF Alesi, Marianna

Pecoraro, Donatella

Pepi, Annamaria

TI Executive functions in kindergarten children at risk for developmental

coordination disorder

SO EUROPEAN JOURNAL OF SPECIAL NEEDS EDUCATION

LA English

DT Article

DE Developmental Coordination Disorder; executive functioning; working

memory; fluency; inhibitory Control; kindergarten

ID MOTOR COORDINATION; PHYSICAL-ACTIVITY; VERBAL FLUENCY; YOUNG-CHILDREN;

PERFORMANCE; DEFICITS; ATTENTION; DYSLEXIA; SKILLS; DIFFICULTIES

AB Executive functioning (EF) is a key cognitive process for development. Little is known about EF in Kindergarten children at risk for developmental coordination disorder (DCD), despite this age being one of the most critical and intensive period of motor and cognitive development. In our investigation we compared EF in kindergarten children at risk for DCD with Typically Developing (TD) children. Participants were 36 Italian children, 18 at risk for DCD (9 boys and 9 girls) who had a mean age of 4.6 years and 18 TD (9 boys and 9 girls) who had a mean age of 4.6. Executive functions were measured by tasks targeting cold executive functioning (working memory, fluency, inhibitory control) and two assessments of hot executive functioning (Snack Delay and Gift Wrap). Significant differences were found between children at risk for DCD and TD children on cold EF tasks of visuo-spatial working memory abilities, fluency and inhibitory control, but not on hot EF tasks. The findings suggest that it is advisable to implement preschool cognitively challenging physical activities programmes.

C1 [Alesi, Marianna; Pecoraro, Donatella; Pepi, Annamaria] Univ Palermo, Dipartimento Sci Psicol Pedag & Formaz, Palermo, Italy.

RP Alesi, M (通讯作者)，Univ Palermo, Dipartimento Sci Psicol Pedag & Formaz, Palermo, Italy.

EM marianna.alesi@unipa.it

CR Abrahams S, 2000, NEUROPSYCHOLOGIA, V38, P734, DOI 10.1016/S0028-3932(99)00146-3

Alesi M, 2017, PROGRAMMA MOTORIO AR

Alesi M, 2015, FRONT PSYCHOL, V6, DOI 10.3389/fpsyg.2015.01627

American Psychiatric Association, 2013, DIAGNOSTIC STAT MANU, DOI DOI 10.1176/APPI.BOOKS.9780890425596

Batey CA, 2014, HUM MOVEMENT SCI, V36, P258, DOI 10.1016/j.humov.2013.10.003

Biotteau M, 2017, EUR J PAEDIATR NEURO, V21, P286, DOI 10.1016/j.ejpn.2016.07.025

Bisiacchi P. S., 2005, BATTERIA VALUTAZIONE

Blank R, 2012, DEV MED CHILD NEUROL, V54, P54, DOI 10.1111/j.1469-8749.2011.04171.x

Cairney J, 2010, DEV MED CHILD NEUROL, V52, pE67, DOI 10.1111/j.1469-8749.2009.03520.x

Camden C, 2016, DISABIL HEALTH J, V9, P406, DOI 10.1016/j.dhjo.2016.04.002

Campbell WN, 2012, PSYCHOL SCHOOLS, V49, P328, DOI 10.1002/pits.21600

Carlson SA, 2005, DEV NEUROPSYCHOL, V28, P595, DOI 10.1207/s15326942dn2802_3

Carson V, 2016, J SCI MED SPORT, V19, P573, DOI 10.1016/j.jsams.2015.07.011

Castellanos FX, 2006, TRENDS COGN SCI, V10, P117, DOI 10.1016/j.tics.2006.01.011

Chaix Y, 2007, EUR J PAEDIATR NEURO, V11, P368, DOI 10.1016/j.ejpn.2007.03.006

Coleman R, 2001, HUM MOVEMENT SCI, V20, P95, DOI 10.1016/S0167-9457(01)00030-6

Diamond Adele, 2015, Ann Sports Med Res, V2, P1011

Diamond A, 2013, ANNU REV PSYCHOL, V64, P135, DOI 10.1146/annurev-psych-113011-143750

Gaines R, 2008, BMC HEALTH SERV RES, V8, DOI 10.1186/1472-6963-8-21

Gernsbacher MA, 2008, J CHILD PSYCHOL PSYC, V49, P43, DOI 10.1111/j.1469-7610.2007.01820.x

Haslum MN, 2007, DYSLEXIA, V13, P257, DOI 10.1002/dys.350

Henderson S., 2007, MOVEMENT ASSESSMENT

Houwen S, 2017, HUM MOVEMENT SCI, V53, P24, DOI 10.1016/j.humov.2016.12.009

Kirby A, 2005, BRIT J SPEC EDUC, V32, P122, DOI 10.1111/j.0952-3383.2005.00384.x

Kochanska G, 1996, CHILD DEV, V67, P490, DOI 10.2307/1131828

Krueger RF, 1996, J PERS, V64, P107, DOI 10.1111/j.1467-6494.1996.tb00816.x

Lanfranchi S, 2004, AM J MENT RETARD, V109, P456, DOI 10.1352/0895-8017(2004)109<456:VAVWMD>2.0.CO;2

Leonard HC, 2015, DEV NEUROPSYCHOL, V40, P201, DOI 10.1080/87565641.2014.997933

Livesey D, 2006, HUM MOVEMENT SCI, V25, P50, DOI 10.1016/j.humov.2005.10.008

Michel E, 2018, CHILD NEUROPSYCHOL, V24, P20, DOI 10.1080/09297049.2016.1223282

Michel E, 2011, CHILD NEUROPSYCHOL, V17, P151, DOI 10.1080/09297049.2010.525501

MILLER E, 1984, BRIT J CLIN PSYCHOL, V23, P53, DOI 10.1111/j.2044-8260.1984.tb00626.x

Missiuna C, 2014, RES DEV DISABIL, V35, P1198, DOI 10.1016/j.ridd.2014.01.007

Miyake A, 2012, CURR DIR PSYCHOL SCI, V21, P8, DOI 10.1177/0963721411429458

Moffitt TE, 2011, P NATL ACAD SCI USA, V108, P2693, DOI 10.1073/pnas.1010076108

Nicolson RI, 2011, CORTEX, V47, P117, DOI 10.1016/j.cortex.2009.08.016

Pearsall-Jones JG, 2011, RES DEV DISABIL, V32, P1245, DOI 10.1016/j.ridd.2011.01.042

Piek JP, 2004, ARCH CLIN NEUROPSYCH, V19, P1063, DOI 10.1016/j.acn.2003.12.007

Prunty M, 2016, HUM MOVEMENT SCI, V49, P54, DOI 10.1016/j.humov.2016.06.003

Rahimi-Golkhandan S, 2016, BRAIN COGNITION, V106, P55, DOI 10.1016/j.bandc.2016.04.010

Rahimi-Golkhandan S, 2015, HUM MOVEMENT SCI, V42, P352, DOI 10.1016/j.humov.2015.06.004

Ruddock S, 2016, BRAIN COGNITION, V109, P84, DOI 10.1016/j.bandc.2016.08.001

Schoemaker K, 2012, J CHILD PSYCHOL PSYC, V53, P111, DOI 10.1111/j.1469-7610.2011.02468.x

Summers J, 2008, HUM MOVEMENT SCI, V27, P215, DOI 10.1016/j.humov.2008.02.002

Vaivre-Douret L, 2016, FRONT PSYCHOL, V7, DOI 10.3389/fpsyg.2016.00502

Vaivre-Douret L, 2011, DEV NEUROPSYCHOL, V36, P614, DOI 10.1080/87565641.2011.560696

van der Fels IMJ, 2015, J SCI MED SPORT, V18, P697, DOI 10.1016/j.jsams.2014.09.007

Wang TN, 2009, DEV MED CHILD NEUROL, V51, P817, DOI 10.1111/j.1469-8749.2009.03271.x

Wilson PH, 2013, DEV MED CHILD NEUROL, V55, P217, DOI 10.1111/j.1469-8749.2012.04436.x

Wright I, 2003, J CHILD PSYCHOL PSYC, V44, P561, DOI 10.1111/1469-7610.00145

Zwicker JG, 2011, INT J DEV NEUROSCI, V29, P145, DOI 10.1016/j.ijdevneu.2010.12.002

NR 51

TC 11

Z9 12

U1 2

U2 11

PU ROUTLEDGE JOURNALS, TAYLOR & FRANCIS LTD

PI ABINGDON

PA 2-4 PARK SQUARE, MILTON PARK, ABINGDON OX14 4RN, OXON, ENGLAND

SN 0885-6257

EI 1469-591X

J9 EUR J SPEC NEEDS EDU

JI Eur. J. Spec. Needs Educ.

PD MAY 27

PY 2019

VL 34

IS 3

BP 285

EP 296

DI 10.1080/08856257.2018.1468635

PG 12

WC Education, Special

WE Social Science Citation Index (SSCI)

SC Education & Educational Research

GA HZ7AN

UT WOS:000469004000003

OA Green Submitted

DA 2022-08-17

ER

PT J

AU Molitor, S

Michel, E

Schneider, W

AF Molitor, Sabine

Michel, Eva

Schneider, Wolfgang

TI Executive Functions in Children with Motor Coordination Impairments

SO KINDHEIT UND ENTWICKLUNG

LA German

DT Article

DE developmental coordination disorder; executive functions; motor

coordination; attention; working memory

ID SCHOOL-AGED CHILDREN; COGNITIVE CONTROL; WORKING-MEMORY; DISORDER;

SUBTYPES; SKILLS; DCD; IDENTIFICATION; PRESCHOOLERS; ATTENTION

AB Links between motor coordination impairments and the development of executive functions have been explored in several recent investigations. In the current study, cognitive and motor coordination skills of kindergarten children (N = 96) aged 5 and 6 years with and without motor coordination impairments were compared. The matched sample consisted of n = 48 control children and n = 48 children with motor coordination impairments. Children with motor coordination impairments showed a marked deficit in the development of executive functions, particularly in inhibition processes. However, a subsample of children with motor coordination impairments showed no deficits in executive functions. These children outperformed the rest of the impairment group in manual dexterity. Underlying processes and implications for interventions are discussed.

C1 [Molitor, Sabine; Michel, Eva; Schneider, Wolfgang] Univ Wurzburg, Inst Psychol, D-97070 Wurzburg, Germany.

RP Molitor, S (通讯作者)，Univ Wurzburg, Lehrstuhl Psychol 4, Rontgenring 10, D-97070 Wurzburg, Germany.

EM sabine.molitor@uni-wuerzburg.de

CR Alloway TP, 2007, J EXP CHILD PSYCHOL, V96, P20, DOI 10.1016/j.jecp.2006.07.002

Alloway TP, 2007, APPL COGNITIVE PSYCH, V21, P473, DOI 10.1002/acp.1284

Bos K, 1987, HDB SPORTMOTORISCHER

CATTELL RB, 1997, GRUNDINTELLIGENZTEST

Cummins A, 2005, DEV MED CHILD NEUROL, V47, P437, DOI 10.1017/S001216220500085X

Davidson MC, 2006, NEUROPSYCHOLOGIA, V44, P2037, DOI 10.1016/j.neuropsychologia.2006.02.006

Dewey D, 2002, HUM MOVEMENT SCI, V21, P905, DOI 10.1016/S0167-9457(02)00163-X

DEWEY D, 1994, DEV NEUROPSYCHOL, V10, P265, DOI 10.1080/87565649409540583

Diamond A, 2007, SCIENCE, V318, P1387, DOI 10.1126/science.1151148

ERIKSEN BA, 1974, PERCEPT PSYCHOPHYS, V16, P143, DOI 10.3758/BF03203267

Garon N, 2008, PSYCHOL BULL, V134, P31, DOI 10.1037/0033-2909.134.1.31

Geuze RH, 2001, HUM MOVEMENT SCI, V20, P7, DOI 10.1016/S0167-9457(01)00027-6

Hasselhorn M., 2012, ARBEITSGEDACHTNISTES

Henderson S. E., 2007, MOVEMENT ABC MOVEMEN

Hill EL, 2001, INT J LANG COMM DIS, V36, P149, DOI 10.1080/13682820010019874

Hoare D., 1994, ADAPT PHYS ACT Q, V11, P158, DOI DOI 10.1123/APAQ.11.2.158

Kaplan BJ, 1998, HUM MOVEMENT SCI, V17, P471, DOI 10.1016/S0167-9457(98)00010-4

Kastner J, 2009, PSYCHOL RUNDSCH, V60, P73, DOI 10.1026/0033-3042.60.2.73

Kaufmann M., 2008, NEUROPSYCHOLOGISCHES

Kiphard E. J, 1974, KORPERKOORDINATIONST

Livesey D, 2006, HUM MOVEMENT SCI, V25, P50, DOI 10.1016/j.humov.2005.10.008

Macnab JJ, 2001, HUM MOVEMENT SCI, V20, P49, DOI 10.1016/S0167-9457(01)00028-8

Mandich A, 2002, BRAIN COGNITION, V50, P150, DOI 10.1016/S0278-2626(02)00020-9

Matthews JS, 2009, J EDUC PSYCHOL, V101, P689, DOI 10.1037/a0014240

McClelland MM, 2007, DEV PSYCHOL, V43, P947, DOI 10.1037/0012-1649.43.4.947

Michel E, 2011, CHILD NEUROPSYCHOL, V17, P151, DOI 10.1080/09297049.2010.525501

Michel E, 2011, KINDH ENTWICKL, V20, P49, DOI 10.1026/0942-5403/a000024

Miyake A, 2000, COGNITIVE PSYCHOL, V41, P49, DOI 10.1006/cogp.1999.0734

Petermann F., 2011, MOVEMENT ASSESSMENT

Piek JP, 2004, ARCH CLIN NEUROPSYCH, V19, P1063, DOI 10.1016/j.acn.2003.12.007

Visser J, 2003, HUM MOVEMENT SCI, V22, P479, DOI 10.1016/j.humov.2003.09.005

Wright HC, 1996, DEV MED CHILD NEUROL, V38, P1099

Zwicker J, 2013, Z SPORTPSYCHOL, V20, P5, DOI 10.1026/1612-5010/a000087

NR 33

TC 12

Z9 12

U1 7

U2 27

PU HOGREFE & HUBER PUBLISHERS

PI GOTTINGEN

PA ROHNSWEG 25, D-37085 GOTTINGEN, GERMANY

SN 0942-5403

EI 2190-6246

J9 KINDH ENTWICKL

JI Kindh. Entwickl.

PY 2015

VL 24

IS 3

BP 181

EP 188

DI 10.1026/0942-5403/a000174

PG 8

WC Psychology, Developmental

WE Social Science Citation Index (SSCI)

SC Psychology

GA CL9GC

UT WOS:000357283900006

DA 2022-08-17

ER

PT J

AU Bernardi, M

Leonard, HC

Hill, EL

Botting, N

Henry, LA

AF Bernardi, Marialivia

Leonard, Hayley C.

Hill, Elisabeth L.

Botting, Nicola

Henry, Lucy A.

TI Executive functions in children with developmental coordination

disorder: a 2-year follow-up study

SO DEVELOPMENTAL MEDICINE AND CHILD NEUROLOGY

LA English

DT Article

ID MEMORY; DIFFICULTIES; ATTENTION; AGE

AB AimExecutive function impairments have been identified in children with poor motor skills, with and without a diagnosis of developmental coordination disorder (DCD). However, most studies are cross-sectional. This study investigates the development of executive function in children with poor motor skills over 2 years.

MethodChildren aged 7 to 11 years (n=51) were assessed twice, 2 years apart, on verbal and nonverbal measures of executive functions: executive-loaded working memory (ELWM); fluency; response inhibition; planning; and cognitive flexibility. Typically developing children (n=17) were compared with those with a clinical diagnosis of DCD (n=17) and those with identified motor difficulties (n=17) but no formal diagnosis of DCD.

ResultsDevelopmental gains in executive function were similar between groups, although a gap between children with poor motor skills and typically developing children on nonverbal executive functions persisted. Specifically, children with DCD performed significantly more poorly than typically developing children on all nonverbal executive function tasks and verbal fluency tasks at both time points; and children with motor difficulties but no diagnosis of DCD showed persistent executive function problems in nonverbal tasks of ELWM and fluency.

InterpretationChildren with DCD and motor difficulties demonstrated executive function difficulties over 2 years, which may affect activities of daily living and academic achievement, in addition to their motor deficit.

C1 [Bernardi, Marialivia; Botting, Nicola; Henry, Lucy A.] City Univ London, Div Language & Commun Sci, London EC1V 0HB, England.

[Leonard, Hayley C.] Univ Surrey, Sch Psychol, Guildford, Surrey, England.

[Hill, Elisabeth L.] Goldsmiths Univ London, Dept Psychol, London, England.

RP Bernardi, M (通讯作者)，City Univ London, Div Language & Commun Sci, London EC1V 0HB, England.

EM Marialivia.Bernardi@city.ac.uk

OI Botting, Nicola/0000-0003-1082-9501; Bernardi,

Marialivia/0000-0002-6844-0358; Hill, Elisabeth/0000-0003-3130-1271

FU Waterloo Foundation [920-2318]; City, University of London PhD

studentship

FX This work was supported by The Waterloo Foundation under grant 920-2318,

and by a City, University of London PhD studentship to MB. We thank all

of the children, parents, teachers, headteachers, and classroom

assistants who assisted with this project. The authors have stated that

they had no interests that might be perceived as posing a conflict or

bias.

CR American Psychiatric Association, 2013, DIAGNOSTIC STAT MANU, DOI DOI 10.1176/APPI.BOOKS.9780890425596

Cambridge Cognition, 2006, CAMBR NEUR TEST AUT

Davidson MC, 2006, NEUROPSYCHOLOGIA, V44, P2037, DOI 10.1016/j.neuropsychologia.2006.02.006

Debrabant J, 2013, RES DEV DISABIL, V34, P1478, DOI 10.1016/j.ridd.2013.02.008

Delis D.C., 2001, DELIS KAPLAN EXECUTI

Diamond A, 2013, ANNU REV PSYCHOL, V64, P135, DOI 10.1146/annurev-psych-113011-143750

Elliot C.D., 2011, BRIT ABILITY SCALES, V3rd Edn

Gathercole SE, 2004, APPL COGNITIVE PSYCH, V18, P1, DOI 10.1002/acp.934

Henderson S., 2007, MOVEMENT ASSESSMENT

Henry LA, 2001, MEMORY, V9, P233, DOI 10.1080/09658210042000085

Henry LA, 2012, J CHILD PSYCHOL PSYC, V53, P37, DOI 10.1111/j.1469-7610.2011.02430.x

Huizinga M, 2006, NEUROPSYCHOLOGIA, V44, P2017, DOI 10.1016/j.neuropsychologia.2006.01.010

Kirby A, 2005, BRIT J SPEC EDUC, V32, P122, DOI 10.1111/j.0952-3383.2005.00384.x

Klenberg L, 2001, DEV NEUROPSYCHOL, V20, P407, DOI 10.1207/S15326942DN2001_6

Leonard H. C., 2015, CURR DEV DISORD REP, V2, P141, DOI [10.1007/s40474-015-0044-8, DOI 10.1007/S40474-015-0044-8]

Leonard HC, 2015, DEV NEUROPSYCHOL, V40, P201, DOI 10.1080/87565641.2014.997933

Levin HS, 1996, DEV NEUROPSYCHOL, V12, P17, DOI 10.1080/87565649609540638

Michel E, 2018, CHILD NEUROPSYCHOL, V24, P20, DOI 10.1080/09297049.2016.1223282

Michel E, 2011, CHILD NEUROPSYCHOL, V17, P151, DOI 10.1080/09297049.2010.525501

Miyake A, 2012, CURR DIR PSYCHOL SCI, V21, P8, DOI 10.1177/0963721411429458

Moffitt TE, 2011, P NATL ACAD SCI USA, V108, P2693, DOI 10.1073/pnas.1010076108

Pennington BF, 1996, J CHILD PSYCHOL PSYC, V37, P51, DOI 10.1111/j.1469-7610.1996.tb01380.x

Pickering S. J., 2001, WORKING MEMORY TEST

Ruddock S, 2016, BRAIN COGNITION, V109, P84, DOI 10.1016/j.bandc.2016.08.001

Saban MT, 2014, RES DEV DISABIL, V35, P2644, DOI 10.1016/j.ridd.2014.07.002

Wilson PH, 2017, DEV MED CHILD NEUROL, V59, P1117, DOI 10.1111/dmcn.13530

Zelazo PD, 2013, MONOGR SOC RES CHILD, V78, P16, DOI 10.1111/mono.12032

NR 27

TC 29

Z9 29

U1 9

U2 26

PU WILEY

PI HOBOKEN

PA 111 RIVER ST, HOBOKEN 07030-5774, NJ USA

SN 0012-1622

EI 1469-8749

J9 DEV MED CHILD NEUROL

JI Dev. Med. Child Neurol.

PD MAR

PY 2018

VL 60

IS 3

BP 306

EP 313

DI 10.1111/dmcn.13640

PG 8

WC Clinical Neurology; Pediatrics

WE Science Citation Index Expanded (SCI-EXPANDED); Social Science Citation Index (SSCI)

SC Neurosciences & Neurology; Pediatrics

GA FW9BA

UT WOS:000425629500021

PM 29238952

OA Green Accepted, Green Submitted, Bronze

DA 2022-08-17

ER

PT J

AU Schurink, J

Hartman, E

Scherder, EJA

Houwen, S

Visscher, C

AF Schurink, J.

Hartman, E.

Scherder, E. J. A.

Houwen, S.

Visscher, C.

TI Relationship between motor and executive functioning in school-age

children with pervasive developmental disorder not otherwise specified

SO RESEARCH IN AUTISM SPECTRUM DISORDERS

LA English

DT Article

DE PDD-NOS; Children; Motor skills; Movement ABC; Executive functioning;

Tower of London

ID SOCIAL-BEHAVIOR QUESTIONNAIRE; MOVEMENT ASSESSMENT BATTERY; AUTISM

SPECTRUM DISORDERS; COORDINATION DISORDER; ASPERGER-SYNDROME; LANGUAGE

IMPAIRMENT; PERFORMANCE; ATTENTION; TOWER; VALIDITY

AB This study examines the motor skills and executive functioning (EF) of 28 children diagnosed with pervasive developmental disorder-not otherwise specified (PDD-NOS; mean age: 10 years 6 months, range: 7-12 years; 19 boys, 9 girls) in comparison with age- and gender-matched typically developing children. The potential relationship between motor performance and EF in children with PDD-NOS is investigated as well. The children's motor skills were evaluated with the Movement ABC. EF, in terms of planning ability, strategic decision making, and problem solving, was gauged with the Tower of London (TOL) task. Compared with their typically developing peers, the children with PDD-NOS scored poor on the Movement ABC (p < 0.01) and the TOL (p < 0.05). They had significantly more definite motor problems than the normative sample of the Movement ABC: 43% (manual dexterity, p < 0.001), 25% (ball skills, p < 0.001), and 25% (balance skills, p < 0.001). There were significant inverse relationships between manual dexterity and the TOL score (r = -0.46, p < 0.01), and balance and the TOL score (r = -0.41, p < 0.05), indicating that children with a better performance on the manual dexterity subtest and the balance subtest had a better TOL score than children with a worse performance. Children with PDD-NOS have inferior motor skills, and these deficits are interrelated with planning ability, strategic decision making, and problem solving. (c) 2011 Elsevier Ltd. All rights reserved.

C1 [Schurink, J.; Hartman, E.; Houwen, S.; Visscher, C.] Univ Groningen, Univ Med Ctr Groningen, Ctr Human Movement Sci, NL-9700 AD Groningen, Netherlands.

[Scherder, E. J. A.] Vrije Univ Amsterdam, Dept Clin Neuropsychol, Amsterdam, Netherlands.

RP Hartman, E (通讯作者)，Univ Groningen, Univ Med Ctr Groningen, Ctr Human Movement Sci, Sect F,POB 196, NL-9700 AD Groningen, Netherlands.

EM e.hartman@med.umcg.nl

CR American Psychiatric Association, 2000, DIAGNOSTIC STAT MANU, V4th

Anderson P, 2002, CHILD NEUROPSYCHOL, V8, P71, DOI 10.1076/chin.8.2.71.8724

Anderson P, 1996, CLIN NEUROPSYCHOL, V10, P54, DOI 10.1080/13854049608406663

Anderson VA, 2001, DEV NEUROPSYCHOL, V20, P385, DOI 10.1207/S15326942DN2001_5

Belmonte MK, 2004, MOL PSYCHIATR, V9, P646, DOI 10.1038/sj.mp.4001499

Cohen J., 1988, STAT POWER ANAL SOCI

Courchesne E, 2004, MENT RETARD DEV D R, V10, P106, DOI 10.1002/mrdd.20020

Croce RV, 2001, PERCEPT MOTOR SKILL, V93, P275, DOI 10.2466/pms.2001.93.1.275

Dawson G, 2004, DEV PSYCHOL, V40, P271, DOI 10.1037/0012-1649.40.2.271

Dewey D, 2007, J INT NEUROPSYCH SOC, V13, P246, DOI 10.1017/S1355617707070270

Diamond A, 2000, CHILD DEV, V71, P44, DOI 10.1111/1467-8624.00117

Fabbri-Destro M, 2009, EXP BRAIN RES, V192, P521, DOI 10.1007/s00221-008-1578-3

Fombonne E, 2005, J APPL RES INTELLECT, V18, P281, DOI 10.1111/j.1468-3148.2005.00266.x

Fuentes CT, 2009, NEUROLOGY, V73, P1532, DOI 10.1212/WNL.0b013e3181c0d48c

Ghaziuddin M, 1998, J INTELL DISABIL RES, V42, P43, DOI 10.1046/j.1365-2788.1998.00065.x

Gioia GA, 2001, PSYCHOLOGICAL AND DEVELOPMENTAL ASSESSMENT, P317

Green D, 2009, DEV MED CHILD NEUROL, V51, P311, DOI 10.1111/j.1469-8749.2008.03242.x

Hartman CA, 2006, J AUTISM DEV DISORD, V36, P325, DOI 10.1007/s10803-005-0072-z

Hartman E, 2010, J INTELL DISABIL RES, V54, P468, DOI 10.1111/j.1365-2788.2010.01284.x

HENDERSON SE, 1992, MOVEMENT ASSESSMENT

Junaid Kathryn A, 2006, Phys Occup Ther Pediatr, V26, P5

Kopp S, 2010, RES DEV DISABIL, V31, P350, DOI 10.1016/j.ridd.2009.09.017

KRIKORIAN R, 1994, J CLIN EXP NEUROPSYC, V16, P840, DOI 10.1080/01688639408402697

Leary MR, 1996, MENT RETARD, V34, P39

Lecavalier L, 2009, J CHILD PSYCHOL PSYC, V50, P1246, DOI 10.1111/j.1469-7610.2009.02104.x

Lezak M. D., 1995, NEUROPSYCHOLOGICAL A

Lindgren KA, 2009, AUTISM RES, V2, P22, DOI 10.1002/aur.63

Luciana M, 2002, DEV NEUROPSYCHOL, V22, P595, DOI 10.1207/S15326942DN2203_3

Luteijn EEF, 1998, J AUTISM DEV DISORD, V28, P559, DOI 10.1023/A:1026060330122

MANJIVIONA J, 1995, J AUTISM DEV DISORD, V25, P23, DOI 10.1007/BF02178165

Matson JL, 2010, RES AUTISM SPECT DIS, V4, P444, DOI 10.1016/j.rasd.2009.10.018

Matson JL, 2009, DEV NEUROREHABIL, V12, P122, DOI 10.1080/17518420902936730

McCrimmon A. W., 2011, RES AUTISM SPECTRUM

Ming X, 2007, BRAIN DEV-JPN, V29, P565, DOI 10.1016/j.braindev.2007.03.002

Miyahara M, 1997, J AUTISM DEV DISORD, V27, P595, DOI 10.1023/A:1025834211548

Pennington BF, 1996, J CHILD PSYCHOL PSYC, V37, P51, DOI 10.1111/j.1469-7610.1996.tb01380.x

Ridler K, 2006, P NATL ACAD SCI USA, V103, P15651, DOI 10.1073/pnas.0602639103

Russo N, 2007, BRAIN COGNITION, V65, P77, DOI 10.1016/j.bandc.2006.04.007

Schall U, 2003, NEUROIMAGE, V20, P1154, DOI 10.1016/S1053-8119(03)00338-0

SEYHAN S, 1993, CLIN REHABIL, V7, P119

Smits-Engelsman BC, 1998, MOVEMENT ASSESSMENT

Smits-Engelsman BCM, 1998, HUM MOVEMENT SCI, V17, P699, DOI 10.1016/S0167-9457(98)00019-0

Smyth MM, 1997, J CHILD PSYCHOL PSYC, V38, P1023, DOI 10.1111/j.1469-7610.1997.tb01619.x

Sonuga-Barke EJS, 2002, DEV NEUROPSYCHOL, V21, P255, DOI 10.1207/S15326942DN2103_3

Tsai CL, 2011, RES AUTISM SPECT DIS, V5, P1092, DOI 10.1016/j.rasd.2010.12.004

Vanvuchelen M, 2007, AUTISM, V11, P225, DOI 10.1177/1362361307076846

Verte S, 2006, J AUTISM DEV DISORD, V36, P351, DOI 10.1007/s10803-006-0074-5

Vuijk PJ, 2010, J INTELL DISABIL RES, V54, P955, DOI 10.1111/j.1365-2788.2010.01318.x

Vuijk PJ, 2011, J LEARN DISABIL-US, V44, P276, DOI 10.1177/0022219410378446

Wassenberg R, 2005, CHILD DEV, V76, P1092, DOI 10.1111/j.1467-8624.2005.00899.x

Westendorp M, 2011, RES DEV DISABIL, V32, P1147, DOI 10.1016/j.ridd.2011.01.009

Williams D, 2008, PSYCHOL BULL, V134, P944, DOI 10.1037/a0013743

Zwicker JG, 2010, PEDIATRICS, V126, pE678, DOI 10.1542/peds.2010-0059

NR 53

TC 14

Z9 16

U1 1

U2 44

PU ELSEVIER SCI LTD

PI OXFORD

PA THE BOULEVARD, LANGFORD LANE, KIDLINGTON, OXFORD OX5 1GB, OXON, ENGLAND

SN 1750-9467

EI 1878-0237

J9 RES AUTISM SPECT DIS

JI Res. Autism Spectr. Disord.

PD APR-JUN

PY 2012

VL 6

IS 2

BP 726

EP 732

DI 10.1016/j.rasd.2011.10.013

PG 7

WC Education, Special; Psychology, Developmental; Psychiatry;

Rehabilitation

WE Social Science Citation Index (SSCI)

SC Education & Educational Research; Psychology; Psychiatry; Rehabilitation

GA 908DH

UT WOS:000301470100017

DA 2022-08-17

ER

PT J

AU Fears, NE

Palmer, SA

Miller, HL

AF Fears, Nicholas E.

Palmer, Stephanie A.

Miller, Haylie L.

TI Motor skills predict adaptive behavior in autistic children and

adolescents

SO AUTISM RESEARCH

LA English

DT Article

DE adaptive behavior; autism spectrum disorder; intelligence; motor skills;

movement disorder

ID DEVELOPMENTAL COORDINATION DISORDER; SPECTRUM DISORDER; POSTURAL

CONTROL; YOUNG-CHILDREN; IMITATION; DYSPRAXIA; SEVERITY; MOVEMENT;

ADULTS; PLAY

AB It is well-documented that intelligence quotient (IQ) is a poor predictor of adaptive behavior scores in autism, with autistic children having lower adaptive behavior scores than would be predicted based on their IQ scores. Differences in motor skills may explain the variability in their adaptive behavior scores. The current study examined how motor skills might explain autistic individuals' low adaptive behavior scores and which individual components of IQ (i.e., verbal comprehension and perceptual reasoning) and motor skills (i.e., manual dexterity, aiming and catching, and balance) may drive this effect. We examined the associations between IQ, motor skills, calibrated severity, and adaptive behavior scores in 45 autistic children and adolescents. Using a t-test, we found a significant difference (p <0.001) between full-scale IQ and adaptive behavior scores, indicating that our participants' adaptive behavior scores were lower than would be expected given their full-scale IQ. Using a linear regression, we investigated whether motor skills predicted adaptive behavior in autistic children and adolescents and found that motor skills scores were associated with adaptive behavior scores (p = 0.022). To further investigate these associations, we used another linear regression to examine how individual components of IQ and motor skills predicted adaptive behavior scores in autistic children and adolescents. Our results indicated that manual dexterity scores were associated with adaptive behavior scores (p = 0.036). These findings clearly illustrate the need for further understanding of autistic individuals' difficulties with adaptive behavior and the potential role of motor skill difficulties that may underlie these difficulties. Lay Summary Autistic children have lower adaptive behavior scores (e.g., daily living skills, social skills, communication) than intelligence scores (e.g., verbal and perceptual skills) along with difficulties with motor skills. Motor skills may explain the gap between adaptive behavior and intelligence. We found motor skills were associated with adaptive behavior in autistic children and adolescents. In particular, hand coordination was associated with adaptive behavior. We need to better understand how autistic individuals' motor skills impact their adaptive behavior to provide effective supports.

C1 [Fears, Nicholas E.; Miller, Haylie L.] Univ North Texas, Dept Phys Therapy, Hlth Sci Ctr, Ft Worth, TX USA.

[Fears, Nicholas E.; Palmer, Stephanie A.; Miller, Haylie L.] Univ Michigan, Sch Kinesiol, 830 N Univ Ave, Ann Arbor, MI 48170 USA.

RP Fears, NE (通讯作者)，Univ Michigan, Sch Kinesiol, 830 N Univ Ave, Ann Arbor, MI 48170 USA.

EM nfears@umich.edu

RI Fears, Nicholas/AAS-7984-2020

OI Fears, Nicholas/0000-0001-7081-0015

FU National Institute of Mental Health [K01-MH107774]; National Institutes

of Health

FX National Institute of Mental Health, Grant/Award Number: K01-MH107774;

National Institutes of Health

CR Adolph KE, 2019, ANNU REV PSYCHOL, V70, P141, DOI 10.1146/annurev-psych-010418-102836

Alvares GA, 2020, AUTISM, V24, P221, DOI 10.1177/1362361319852831

Bhat AN, 2021, AUTISM RES, V14, P202, DOI 10.1002/aur.2453

Bojanek EK, 2020, J NEURODEV DISORD, V12, DOI 10.1186/s11689-019-9305-x

Cameron CE, 2016, CHILD DEV PERSPECT, V10, P93, DOI 10.1111/cdep.12168

Carment L, 2020, AUTISM RES, V13, P885, DOI 10.1002/aur.2287

Chen LC, 2019, PHYS THER, V99, P1231, DOI 10.1093/ptj/pzz084

Curtin C, 2010, BMC PEDIATR, V10, DOI 10.1186/1471-2431-10-11

Delgado-Lobete L, 2022, DEV MED CHILD NEUROL, V64, P220, DOI 10.1111/dmcn.15036

Duncan AW, 2015, AUTISM, V19, P64, DOI 10.1177/1362361313510068

Dziuk MA, 2007, DEV MED CHILD NEUROL, V49, P734, DOI 10.1111/j.1469-8749.2007.00734.x

Glazebrook CM, 2006, MOTOR CONTROL, V10, P244, DOI 10.1123/mcj.10.3.244

Green D, 2009, DEV MED CHILD NEUROL, V51, P311, DOI 10.1111/j.1469-8749.2008.03242.x

Henderson S., 2007, MOVEMENT ASSESSMENT

Houwen S, 2016, RES DEV DISABIL, V53-54, P19, DOI 10.1016/j.ridd.2016.01.012

Hus V, 2014, J AUTISM DEV DISORD, V44, P1996, DOI 10.1007/s10803-014-2080-3

Karras HC, 2019, RES DEV DISABIL, V84, P85, DOI 10.1016/j.ridd.2018.05.012

Kraper CK, 2017, J AUTISM DEV DISORD, V47, P3007, DOI 10.1007/s10803-017-3213-2

Libertus K, 2016, FRONT PSYCHOL, V7, DOI 10.3389/fpsyg.2016.00475

Lidstone DE, 2021, PEDIATR NEUROL, V122, P98, DOI 10.1016/j.pediatrneurol.2021.06.010

Lim YH, 2017, J AUTISM DEV DISORD, V47, P2238, DOI 10.1007/s10803-017-3144-y

Logan SW, 2012, CHILD CARE HLTH DEV, V38, P305, DOI 10.1111/j.1365-2214.2011.01307.x

Lord C., 2014, ADOS 2 MANUAL I

MacDonald M, 2017, ADAPT PHYS ACT Q, V34, P19, DOI 10.1123/APAQ.2016-0028

MacDonald M, 2014, ADAPT PHYS ACT Q, V31, P95, DOI 10.1123/apaq.2013-0068

Macdonald Megan, 2011, BMC Res Notes, V4, P422, DOI 10.1186/1756-0500-4-422

Magalhaes LC, 2011, RES DEV DISABIL, V32, P1309, DOI 10.1016/j.ridd.2011.01.029

McCormick CEB, 2020, AUTISM RES, V13, P474, DOI 10.1002/aur.2261

McCrimmon AW, 2013, J PSYCHOEDUC ASSESS, V31, P337, DOI 10.1177/0734282912467756

Miller HL, 2021, J AUTISM DEV DISORD, V51, P3443, DOI 10.1007/s10803-020-04766-z

Miller HL, 2019, GAIT POSTURE, V67, P9, DOI 10.1016/j.gaitpost.2018.08.038

Mosconi MW, 2015, J NEUROSCI, V35, P2015, DOI 10.1523/JNEUROSCI.2731-14.2015

Mostofsky SH, 2006, J INT NEUROPSYCH SOC, V12, P314, DOI 10.1017/S1355617706060437

Perry A, 2009, J AUTISM DEV DISORD, V39, P1066, DOI 10.1007/s10803-009-0704-9

R Core Team, 2018, R LANG ENV STAT COMP

Redondo-Tebar A, 2021, RES DEV DISABIL, V119, DOI 10.1016/j.ridd.2021.104087

RESTALL G, 1994, AM J OCCUP THER, V48, P113, DOI 10.5014/ajot.48.2.113

Robinson LE, 2012, EARLY CHILD EDUC J, V40, P79, DOI 10.1007/s10643-011-0496-3

Smith LE, 2012, J AM ACAD CHILD PSY, V51, P622, DOI 10.1016/j.jaac.2012.03.001

Soska KC, 2010, DEV PSYCHOL, V46, P129, DOI 10.1037/a0014618

Sparrow S.S., 2016, VINELAND ADAPTIVE BE

Tamplain P, 2021, CURR DEV DISORD REP, V8, P24, DOI 10.1007/s40474-020-00209-7

Taverna EC, 2021, RES AUTISM SPECT DIS, V86, DOI 10.1016/j.rasd.2021.101824

Travers BG, 2017, DEVELOPMENTAL SCI, V20, DOI 10.1111/desc.12401

Tyler Kiley, 2014, Autism Res Treat, V2014, P312163, DOI 10.1155/2014/312163

Wang ZH, 2016, J CHEMINFORMATICS, V8, DOI 10.1186/s13321-016-0130-x

West KL, 2019, CHILD DEV, V90, pE356, DOI 10.1111/cdev.12980

Wolfers T, 2019, NEUROSCI BIOBEHAV R, V104, P240, DOI 10.1016/j.neubiorev.2019.07.010

Yu C, 2017, COGNITIVE SCI, V41, P5, DOI 10.1111/cogs.12366

Zukerman G, 2021, J AUTISM DEV DISORD, V51, P1466, DOI 10.1007/s10803-020-04632-y

Zwicker JG, 2018, BRIT J OCCUP THER, V81, P65, DOI 10.1177/0308022617735046

NR 51

TC 1

Z9 1

U1 7

U2 7

PU WILEY

PI HOBOKEN

PA 111 RIVER ST, HOBOKEN 07030-5774, NJ USA

SN 1939-3792

EI 1939-3806

J9 AUTISM RES

JI Autism Res.

PD JUN

PY 2022

VL 15

IS 6

BP 1083

EP 1089

DI 10.1002/aur.2708

EA MAR 2022

PG 7

WC Behavioral Sciences; Psychology, Developmental

WE Science Citation Index Expanded (SCI-EXPANDED); Social Science Citation Index (SSCI)

SC Behavioral Sciences; Psychology

GA 1T4VQ

UT WOS:000772231200001

PM 35322578

OA Green Published

DA 2022-08-17

ER

PT J

AU Verbecque, E

Johnson, C

Rameckers, E

Thijs, A

van der Veer, I

Meyns, P

Smits-Engelsman, B

Klingels, K

AF Verbecque, Evi

Johnson, Charlotte

Rameckers, Eugene

Thijs, Angelina

van der Veer, Ingrid

Meyns, Pieter

Smits-Engelsman, Bouwien

Klingels, Katrijn

TI Balance control in individuals with developmental coordination disorder:

A systematic review and meta-analysis

SO GAIT & POSTURE

LA English

DT Review

DE Postural balance; Balance control; Developmental coordination disorder;

Motor skills disorders

ID POSTURAL CONTROL; STATIC BALANCE; LIFTING TASK; MINI-BESTEST; CHILDREN;

PERFORMANCE; WALKING; STABILITY; MOVEMENT; MOTOR

AB Background: Although it is recognized that the majority of children with developmental coordination disorder (DCD) have balance deficits, comprehensive insights into which balance domains are affected, are still lacking in literature.

Research question: To what extent is balance control deficient in individuals with DCD compared to controls?

Methods: Pubmed, Scopus and Web of Science were systematically searched. Risk of bias was assessed with the Scottish Intercollegiate Guidelines Network checklist for case-control studies. Mean and standard deviations characterizing balance control were extracted to calculate standardized mean differences (SMD) and pooled, if possible, using Review Manager.

Results: The results of 31 studies (1152 individuals with DCD, 1103 typically developing (TD) peers, mean age 10.4 years old) were extracted of which 17 were used for meta-analysis. The mean SMD for the balance subscale of the Movement Assessment Battery for Children was 1.63 (pooled 95 %CI =[1.30;1.97]), indicating children with DCD to perform significantly poorer than their TD peers. Force plate studies also revealed that children with DCD present with a larger sway path during bipedal stance with eyes closed (pooled mean SMD = 0.55; 95 %CI = [0.32;0.78]). Children with DCD tend to have direction-specific limited stability limits and task-independent delayed onset of anticipatory postural adjustments.

Interpretation: Children with DCD perform poorer on different domains of balance compared to TD peers. Future research should focus on comprehensive balance assessment in these children, preferably using a longitudinal design.

C1 [Verbecque, Evi; Rameckers, Eugene; van der Veer, Ingrid; Meyns, Pieter; Klingels, Katrijn] Hasselt Univ, Rehabil Res Ctr REVAL, Rehabil Sci & Physiotherapy, Agoralaan Bldg A, B-3590 Diepenbeek, Belgium.

[Verbecque, Evi; Johnson, Charlotte] Univ Antwerp, Fac Med & Hlth Sci, Dept Rehabil Sci & Physiotherapy Movant, Antwerp, Belgium.

[Rameckers, Eugene] Maastricht Univ, Dept Rehabil Med, Maastricht, Netherlands.

[Rameckers, Eugene] Ctr Expertise Adelante Rehabil, Valkenburg, Netherlands.

[Rameckers, Eugene; van der Veer, Ingrid] Univ Profess, AVANSplus, Breda, Netherlands.

[Thijs, Angelina] Ctr Dev Adv & Pediat Neurorehabil Wildermeth Fdn, Biel, Switzerland.

[Smits-Engelsman, Bouwien] Univ Cape Town, Fac Hlth Sci, Dept Hlth & Rehabil Sci, Div Physiotherapy, Cape Town, South Africa.

RP Verbecque, E (通讯作者)，Hasselt Univ, Agoralaan Bldg A, B-3590 Diepenbeek, Belgium.

EM evi.verbecque@uhasselt.be

RI Verbecque, Evi/AAE-2526-2021

OI Verbecque, Evi/0000-0001-8116-1620; rameckers,

eugene/0000-0001-6661-6500; Meyns, Pieter/0000-0003-2218-187X; Klingels,

Katrijn/0000-0001-5853-9680; Johnson, Charlotte/0000-0002-7540-1437

CR [Anonymous], 2020, SCOTTISH INTERCOLLEG

APA, 2013, DIAGNS STAT MAN MENT

Asonitou K, 2012, RES DEV DISABIL, V33, P996, DOI 10.1016/j.ridd.2012.01.008

Bair WN, 2012, PLOS ONE, V7, DOI 10.1371/journal.pone.0040932

Bair WN, 2011, GAIT POSTURE, V34, P183, DOI 10.1016/j.gaitpost.2011.04.007

Blank R, 2019, DEV MED CHILD NEUROL, V61, P242, DOI 10.1111/dmcn.14132

Chen FC, 2014, EXP BRAIN RES, V232, P2155, DOI 10.1007/s00221-014-3906-0

Chen FC, 2011, RES DEV DISABIL, V32, P1948, DOI 10.1016/j.ridd.2011.03.027

Chen FC, 2016, GAIT POSTURE, V45, P193, DOI 10.1016/j.gaitpost.2016.01.029

Cheng YTY, 2018, GAIT POSTURE, V62, P20, DOI 10.1016/j.gaitpost.2018.02.025

Cherng RJ, 2007, HUM MOVEMENT SCI, V26, P913, DOI 10.1016/j.humov.2007.05.006

Deconinck FJA, 2006, CHILD CARE HLTH DEV, V32, P711, DOI 10.1111/j.1365-2214.2006.00685.x

Deconinck FJA, 2010, GAIT POSTURE, V32, P327, DOI 10.1016/j.gaitpost.2010.05.018

Deconinck FJA, 2006, MOTOR CONTROL, V10, P125, DOI 10.1123/mcj.10.2.125

Deconinck FJA, 2008, ADAPT PHYS ACT Q, V25, P17, DOI 10.1123/apaq.25.1.17

Dewar R, 2017, GAIT POSTURE, V55, P68, DOI 10.1016/j.gaitpost.2017.04.010

Dewar R, 2019, ARCH PHYS MED REHAB, V100, P695, DOI 10.1016/j.apmr.2018.12.021

Di Carlo S, 2016, INT J REHABIL RES, V39, P97, DOI 10.1097/MRR.0000000000000153

Dijkstra BW, 2020, NEUROSCI BIOBEHAV R, V115, P351, DOI 10.1016/j.neubiorev.2020.04.028

Du WC, 2015, HUM MOVEMENT SCI, V43, P9, DOI 10.1016/j.humov.2015.06.010

Fong S.S., 2011, RES DEV DISABIL, V32

Fong S.S., 2013, RES DEV DISABIL, V34

Fong S.S.M., 2016, MEDICINE, V95

Fong SSM, 2016, GAIT POSTURE, V43, P60, DOI 10.1016/j.gaitpost.2015.10.026

Fong SSM, 2015, MEDICINE, V94, DOI 10.1097/MD.0000000000001785

Fong SSM, 2012, HUM MOVEMENT SCI, V31, P1317, DOI 10.1016/j.humov.2011.11.003

Gentle J, 2016, HUM MOVEMENT SCI, V49, P346, DOI 10.1016/j.humov.2016.08.010

Geuze R.H., 2005, NEURAL PLAST, V12, P263

Geuze Reint H., 2005, Neural Plasticity, V12, P183, DOI 10.1155/NP.2005.183

Geuze RH, 2003, HUM MOVEMENT SCI, V22, P527, DOI 10.1016/j.humov.2003.09.008

Grove CR, 2007, HUM MOVEMENT SCI, V26, P457, DOI 10.1016/j.humov.2007.01.014

Haddad JM, 2013, EXERC SPORT SCI REV, V41, P123, DOI 10.1097/JES.0b013e3182877cc8

Haddad JM, 2010, GAIT POSTURE, V32, P592, DOI 10.1016/j.gaitpost.2010.08.008

Higgins JPT, 2002, STAT MED, V21, P1539, DOI 10.1002/sim.1186

Hillier S, 2010, INT J STROKE, V5, P178, DOI 10.1111/j.1747-4949.2010.00427.x

Hootman JM, 2011, RES SYNTH METHODS, V2, P110, DOI 10.1002/jrsm.41

Horak FB, 2009, PHYS THER, V89, P484, DOI 10.2522/ptj.20080071

Jacobs JV, 2007, J NEURAL TRANSM, V114, P1339, DOI 10.1007/s00702-007-0657-0

Johnston LM, 2002, HUM MOVEMENT SCI, V21, P583, DOI 10.1016/S0167-9457(02)00153-7

Jover M, 2010, DEV MED CHILD NEUROL, V52, P850, DOI 10.1111/j.1469-8749.2009.03611.x

Jucaite A, 2003, DEV MED CHILD NEUROL, V45, P731, DOI 10.1017/S0012162203001373

Kane K, 2014, PHYS OCCUP THER PEDI, V34, P75, DOI 10.3109/01942638.2012.757574

Kane K, 2012, HUM MOVEMENT SCI, V31, P707, DOI 10.1016/j.humov.2011.08.004

Laufer Y, 2008, GAIT POSTURE, V27, P347, DOI 10.1016/j.gaitpost.2007.04.013

Lee DK, 2016, KOREAN J ANESTHESIOL, V69, P555, DOI 10.4097/kjae.2016.69.6.555

Macnab JJ, 2001, HUM MOVEMENT SCI, V20, P49, DOI 10.1016/S0167-9457(01)00028-8

Miller HL, 2019, GAIT POSTURE, V67, P9, DOI 10.1016/j.gaitpost.2018.08.038

Moher David, 2009, Ann Intern Med, V151, P264, DOI 10.1136/bmj.b2535

Przysucha EP, 2004, ADAPT PHYS ACT Q, V21, P19, DOI 10.1123/apaq.21.1.19

Przysucha EP, 2008, ADAPT PHYS ACT Q, V25, P1

Sankar U. Ganapathy, 2019, IND J PUBL HLTH RES, V10, P67, DOI DOI 10.5958/0976-5506.2019.01538.9

Shumway-Cook W.M., 2012, MOTOR CONTROL TRANSL

Sibley KM, 2017, ARCH PHYS MED REHAB, V98, P2066, DOI 10.1016/j.apmr.2017.02.032

Speedtsberg MB, 2018, GAIT POSTURE, V59, P99, DOI 10.1016/j.gaitpost.2017.09.035

Speedtsberg MB, 2017, GAIT POSTURE, V51, P1, DOI 10.1016/j.gaitpost.2016.09.019

Sugden DA, 2007, CHILD CARE HLTH DEV, V33, P520, DOI 10.1111/j.1365-2214.2006.00707.x

Tsai CL, 2009, CHILD CARE HLTH DEV, V35, P551, DOI 10.1111/j.1365-2214.2009.00974.x

Tsai CL, 2008, HUM MOVEMENT SCI, V27, P142, DOI 10.1016/j.humov.2007.08.002

Tsai CL, 2008, PERCEPT MOTOR SKILL, V107, P457, DOI 10.2466/PMS.107.2.457-472

Tsang WWN, 2012, RES DEV DISABIL, V33, P1898, DOI 10.1016/j.ridd.2012.05.015

Wagner MO, 2011, RES DEV DISABIL, V32, P1970, DOI 10.1016/j.ridd.2011.04.004

Williams H, 1997, AUSTR ED DEV PSYCHOL, V14, P43

Wilmut K, 2017, EXP BRAIN RES, V235, P1531, DOI 10.1007/s00221-017-4901-z

Wilmut K, 2016, EXP BRAIN RES, V234, P1747, DOI 10.1007/s00221-016-4592-x

Wilson PH, 2017, DEV MED CHILD NEUROL, V59, P1117, DOI 10.1111/dmcn.13530

Yam TTT, 2019, J MOTOR BEHAV, V51, P385, DOI 10.1080/00222895.2018.1485011

NR 66

TC 4

Z9 4

U1 5

U2 12

PU ELSEVIER IRELAND LTD

PI CLARE

PA ELSEVIER HOUSE, BROOKVALE PLAZA, EAST PARK SHANNON, CO, CLARE, 00000,

IRELAND

SN 0966-6362

EI 1879-2219

J9 GAIT POSTURE

JI Gait Posture

PD JAN

PY 2021

VL 83

BP 268

EP 279

DI 10.1016/j.gaitpost.2020.10.009

PG 12

WC Neurosciences; Orthopedics; Sport Sciences

WE Science Citation Index Expanded (SCI-EXPANDED); Social Science Citation Index (SSCI)

SC Neurosciences & Neurology; Orthopedics; Sport Sciences

GA PN3SQ

UT WOS:000604402600040

PM 33227605

OA Green Published, Green Submitted

DA 2022-08-17

ER

PT J

AU Miller, M

Chukoskie, L

Zinni, M

Townsend, J

Trauner, D

AF Miller, M.

Chukoskie, L.

Zinni, M.

Townsend, J.

Trauner, D.

TI Dyspraxia, motor function and visual-motor integration in autism

SO BEHAVIOURAL BRAIN RESEARCH

LA English

DT Article

DE Autism; Dyspraxia; Motor; Visual-motor integration; Eye movement;

Cerebellum

ID DEVELOPMENTAL COORDINATION DISORDER; SPECTRUM DISORDERS;

ASPERGER-SYNDROME; INFANTILE-AUTISM; YOUNG-CHILDREN; BASAL GANGLIA;

EXECUTIVE FUNCTION; INTERNAL-MODELS; EYE-MOVEMENTS; CEREBELLUM

AB This project assessed dyspraxia in high-functioning school aged children with autism with a focus on Ideational Praxis. We examined the association of specific underlying motor function including eye movement with ideational dyspraxia (sequences of skilled movements) as well as the possible role of visual-motor integration in dyspraxia. We found that compared to IQ-, sex- and age-matched typically developing children, the children with autism performed significantly worse on: Ideational and Buccofacial praxis; a broad range of motor tests, including measures of simple motor skill, timing and accuracy of saccadic eye movements and motor coordination; and tests of visual-motor integration. Impairments in individual children with autism were heterogeneous in nature, although when we examined the praxis data as a function of a qualitative measure representing motor timing, we found that children with poor motor timing performed worse on all praxis categories and had slower and less accurate eye movements while those with regular timing performed as well as typical children on those same tasks. Our data provide evidence that both motor function and visual-motor integration contribute to dyspraxia. We suggest that dyspraxia in autism involves cerebellar mechanisms of movement control and the integration of these mechanisms with cortical networks implicated in praxis. (C) 2014 Elsevier B.V. All rights reserved.

C1 [Miller, M.] Univ Calif San Diego, Sch Med, La Jolla, CA 92093 USA.

[Chukoskie, L.] Univ Calif San Diego, Inst Neural Computat, La Jolla, CA 92093 USA.

[Zinni, M.; Townsend, J.; Trauner, D.] Univ Calif San Diego, Dept Neurosci, La Jolla, CA 92093 USA.

RP Townsend, J (通讯作者)，Univ Calif San Diego, Dept Neurosci, 9500 Gilman Dr,MC-0959, La Jolla, CA 92093 USA.

EM jtownsend@ucsd.edu

OI Chukoskie, Leanne/0000-0003-4041-3646

FU NIH [2 T35 HL 7491-31]; NINDS [P50-NS22343, R21-NS070296]; NSF [SMA

1041755]; NATIONAL HEART, LUNG, AND BLOOD INSTITUTE [T35HL007491]

Funding Source: NIH RePORTER; NATIONAL INSTITUTE OF NEUROLOGICAL

DISORDERS AND STROKE [P50NS022343, R21NS070296] Funding Source: NIH

RePORTER

FX This study was funded by the NIH 2 T35 HL 7491-31 (MM), NINDS

P50-NS22343 (DT), NINDS R21-NS070296 (JT) and NSF SMA 1041755 TDLC

Science of Learning Center (LC). Tyler Brocklehurst (Institute for

Neural Computation, UCSD) and Carin Rojas (School of Medicine,

Northwestern University) who served as trained raters scoring praxis

videos.

CR Abell F, 1999, NEUROREPORT, V10, P1647, DOI 10.1097/00001756-199906030-00005

Bair WN, 2012, PLOS ONE, V7, DOI 10.1371/journal.pone.0040932

Baranek GT, 1999, J AUTISM DEV DISORD, V29, P213, DOI 10.1023/A:1023080005650

Barnea-Goraly N, 2004, BIOL PSYCHIAT, V55, P323, DOI 10.1016/j.biopsych.2003.10.022

Baron-Cohen S, 2005, ANNU REV NEUROSCI, V28, P109, DOI 10.1146/annurev.neuro.27.070203.144137

Bauman ML, 1997, INT REV NEUROBIOL, V41, P367, DOI 10.1016/S0074-7742(08)60360-8

BAUMAN ML, 1991, PEDIATRICS, V87, P791

Beery K, 2006, HE BEERY BUKTENICA D

Belmonte MK, 2004, J NEUROSCI, V24, P9228, DOI 10.1523/JNEUROSCI.3340-04.2004

Bhanpuri NH, 2013, J NEUROSCI, V33, P14301, DOI 10.1523/JNEUROSCI.0784-13.2013

Bhat AN, 2012, INFANT BEHAV DEV, V35, P838, DOI 10.1016/j.infbeh.2012.07.019

Bhat AN, 2011, PHYS THER, V91, P1116, DOI 10.2522/ptj.20100294

Bostan AC, 2013, TRENDS COGN SCI, V17, P241, DOI 10.1016/j.tics.2013.03.003

Bruininks R.H., 2005, BRUININKS OSERETSKY

Chukoskie L, 2013, INT REV NEUROBIOL, V113, P207, DOI 10.1016/B978-0-12-418700-9.00007-1

Constantino JohnN., 2005, SOCIAL RESPONSIVENES

Courchesne E, 1999, NEUROLOGY, V52, P1106, DOI 10.1212/WNL.52.5.1106

COURCHESNE E, 1994, LANCET, V343, P63, DOI 10.1016/S0140-6736(94)90923-7

Crippa A, 2013, J AUTISM DEV DISORD, V43, P841, DOI 10.1007/s10803-012-1623-8

DAMASIO AR, 1978, ARCH NEUROL-CHICAGO, V35, P777, DOI 10.1001/archneur.1978.00500360001001

DEMYER MK, 1972, J AUTISM CHILD SCHIZ, V2, P264, DOI 10.1007/BF01537618

DENCKLA MB, 1985, PSYCHOPHARMACOL BULL, V21, P773

DEWEY D, 1993, BRAIN COGNITION, V23, P203, DOI 10.1006/brcg.1993.1055

Dewey D, 2007, J INT NEUROPSYCH SOC, V13, P246, DOI 10.1017/S1355617707070270

DIXON WJ, 1990, BMDP STAT SOFTWARE M

Dowd AM, 2012, J AUTISM DEV DISORD, V42, P1539, DOI 10.1007/s10803-011-1385-8

Dowell LR, 2009, NEUROPSYCHOLOGY, V23, P563, DOI 10.1037/a0015640

Downey R, 2012, PEDIATR PHYS THER, V24, P2, DOI 10.1097/PEP.0b013e31823db95f

Dziuk MA, 2007, DEV MED CHILD NEUROL, V49, P734, DOI 10.1111/j.1469-8749.2007.00734.x

Esposito G, 2009, BRAIN DEV-JPN, V31, P131, DOI 10.1016/j.braindev.2008.04.005

Fatemi SH, 2012, CEREBELLUM, V11, P777, DOI 10.1007/s12311-012-0355-9

Gernsbacher MA, 2008, J CHILD PSYCHOL PSYC, V49, P43, DOI 10.1111/j.1469-7610.2007.01820.x

GHAZIUDDIN M, 1994, J INTELL DISABIL RES, V38, P519

Gibbs J, 2007, ARCH DIS CHILD, V92, P534, DOI 10.1136/adc.2005.088054

Goldberg MC, 2002, NEUROPSYCHOLOGIA, V40, P2039, DOI 10.1016/S0028-3932(02)00059-3

Haas RH, 1996, J CHILD NEUROL, V11, P84, DOI 10.1177/088307389601100204

HASHIMOTO T, 1995, J AUTISM DEV DISORD, V25, P1, DOI 10.1007/BF02178163

Haswell CC, 2009, NAT NEUROSCI, V12, P970, DOI 10.1038/nn.2356

HENDERSON SE, 1992, MOVEMENT ASSESSMENT

Hughes C, 1996, J AUTISM DEV DISORD, V26, P99, DOI 10.1007/BF02276237

Ivry Richard B., 2003, Neural Plasticity, V10, P141, DOI 10.1155/NP.2003.141

Izawa J, 2012, AUTISM RES, V5, P124, DOI 10.1002/aur.1222

Jansiewicz EM, 2006, J AUTISM DEV DISORD, V36, P613, DOI 10.1007/s10803-006-0109-y

Johnson BP, 2012, FRONT INTEGR NEUROSC, V6, DOI 10.3389/fnint.2012.00099

Just MA, 2007, CEREB CORTEX, V17, P951, DOI 10.1093/cercor/bhl006

Kanner L, 1943, NERV CHILD, V2, P217

Keehn B, 2013, HUM BRAIN MAPP, V34, P2524, DOI 10.1002/hbm.22084

Kourkoulou A, 2013, AUTISM RES, V6, P177, DOI 10.1002/aur.1274

Kuhn G, 2010, PSYCHOL SCI, V21, P1487, DOI 10.1177/0956797610383435

Landa R, 2006, J CHILD PSYCHOL PSYC, V47, P629, DOI 10.1111/j.1469-7610.2006.01531.x

Landry R, 2004, J CHILD PSYCHOL PSYC, V45, P1115, DOI 10.1111/j.1469-7610.2004.00304.x

Lane A, 2012, PEDIATR PHYS THER, V24, P21, DOI 10.1097/PEP.0b013e31823e071a

Lewis JD, 2013, FRONT HUM NEUROSCI, V7, DOI 10.3389/fnhum.2013.00845

Lewis JD, 2013, HUM BRAIN MAPP, V34, P1685, DOI 10.1002/hbm.22018

LINCOLN AJ, 1988, J AUTISM DEV DISORD, V18, P505, DOI 10.1007/BF02211870

Lloyd M, 2013, AUTISM, V17, P133, DOI 10.1177/1362361311402230

Luna B, 2007, BIOL PSYCHIAT, V61, P474, DOI 10.1016/j.biopsych.2006.02.030

MacNeil LK, 2012, NEUROPSYCHOLOGY, V26, P165, DOI 10.1037/a0026955

Manto M, 2012, CEREBELLUM, V11, P457, DOI 10.1007/s12311-011-0331-9

Mari M, 2003, PHILOS T ROY SOC B, V358, P393, DOI 10.1098/rstb.2002.1205

Maski KP, 2011, CURR OPIN PEDIATR, V23, P609, DOI 10.1097/MOP.0b013e32834c9282

Ming X, 2007, BRAIN DEV-JPN, V29, P565, DOI 10.1016/j.braindev.2007.03.002

Minshew NJ, 1999, NEUROLOGY, V52, P917, DOI 10.1212/WNL.52.5.917

Miyahara M, 1997, J AUTISM DEV DISORD, V27, P595, DOI 10.1023/A:1025834211548

Mosconi MW, 2009, PSYCHOL MED, V39, P1559, DOI 10.1017/S0033291708004984

Mosconi MW, 2013, PLOS ONE, V8, DOI 10.1371/journal.pone.0063709

Mostofsky SH, 2006, J INT NEUROPSYCH SOC, V12, P314, DOI 10.1017/S1355617706060437

Mostofsky SH, 2011, NEUROSCIENTIST, V17, P437, DOI 10.1177/1073858410392381

Murias M, 2007, BIOL PSYCHIAT, V62, P270, DOI 10.1016/j.biopsych.2006.11.012

Price KJ, 2012, RES AUTISM SPECT DIS, V6, P857, DOI 10.1016/j.rasd.2011.11.007

Rinehart NJ, 2006, DEV MED CHILD NEUROL, V48, P819, DOI 10.1017/S0012162206001769

Rogers SJ, 2003, J CHILD PSYCHOL PSYC, V44, P763, DOI 10.1111/1469-7610.00162

Rogers SJ, 1996, CHILD DEV, V67, P2060, DOI 10.2307/1131609

Rommelse NNJ, 2008, BRAIN COGNITION, V68, P391, DOI 10.1016/j.bandc.2008.08.025

ROSENHALL U, 1988, J LARYNGOL OTOL, V102, P435, DOI 10.1017/S0022215100105286

Schlerf JE, 2007, CEREBELLUM, V6, P221, DOI 10.1080/14734220701370643

Schmahmann JD, 2004, J COMP NEUROL, V478, P248, DOI 10.1002/cne.20286

Schmahmann JD, 2008, CORTEX, V44, P1037, DOI 10.1016/j.cortex.2008.04.004

Smith IM, 2007, COGN NEUROPSYCHOL, V24, P679, DOI 10.1080/02643290701669703

Steinman KJ, 2010, J CHILD NEUROL, V25, P71, DOI 10.1177/0883073809342591

Stoodley CJ, 2012, NEUROIMAGE, V59, P1560, DOI 10.1016/j.neuroimage.2011.08.065

Sutera S, 2007, J AUTISM DEV DISORD, V37, P98, DOI 10.1007/s10803-006-0340-6

Takarae Y, 2004, J NEUROL NEUROSUR PS, V75, P1359, DOI 10.1136/jnnp.2003.022491

Teitelbaum P, 1998, P NATL ACAD SCI USA, V95, P13982, DOI 10.1073/pnas.95.23.13982

Townsend J, 2010, HANDBOOK OF MEDICAL NEUROPSYCHOLOGY: APPLICATIONS OF COGNITIVE NEUROSCIENCE, P165, DOI 10.1007/978-1-4419-1364-7_10

van der Geest JN, 2001, BIOL PSYCHIAT, V50, P614, DOI 10.1016/S0006-3223(01)01070-8

VILENSKY JA, 1981, ARCH NEUROL-CHICAGO, V38, P646, DOI 10.1001/archneur.1981.00510100074013

Wechsler D., 2011, WECHSLER ABBREVIATED, VSecond, pP

Weimer AK, 2001, J DEV BEHAV PEDIATR, V22, P92, DOI 10.1097/00004703-200104000-00002

Weiss MJ, 2013, FRONT INTEGR NEUROSC, V7, DOI 10.3389/fnint.2013.00033

Whyatt CP, 2012, J AUTISM DEV DISORD, V42, P1799, DOI 10.1007/s10803-011-1421-8

Williams JHG, 2004, J AUTISM DEV DISORD, V34, P285, DOI 10.1023/B:JADD.0000029551.56735.3a

Williams JHG, 2001, NEUROSCI BIOBEHAV R, V25, P287, DOI 10.1016/S0149-7634(01)00014-8

WOLPERT DM, 1995, SCIENCE, V269, P1880, DOI 10.1126/science.7569931

Wolpert DM, 1998, TRENDS COGN SCI, V2, P338, DOI 10.1016/S1364-6613(98)01221-2

NR 95

TC 48

Z9 49

U1 5

U2 69

PU ELSEVIER SCIENCE BV

PI AMSTERDAM

PA PO BOX 211, 1000 AE AMSTERDAM, NETHERLANDS

SN 0166-4328

EI 1872-7549

J9 BEHAV BRAIN RES

JI Behav. Brain Res.

PD AUG 1

PY 2014

VL 269

BP 95

EP 102

DI 10.1016/j.bbr.2014.04.011

PG 8

WC Behavioral Sciences; Neurosciences

WE Science Citation Index Expanded (SCI-EXPANDED); Social Science Citation Index (SSCI)

SC Behavioral Sciences; Neurosciences & Neurology

GA AJ7GY

UT WOS:000337866400014

PM 24742861

OA Green Accepted, Green Submitted

DA 2022-08-17

ER

PT J

AU Michel, E

Molitor, S

Schneider, W

AF Michel, Eva

Molitor, Sabine

Schneider, Wolfgang

TI Differential changes in the development of motor coordination and

executive functions in children with motor coordination impairments

SO CHILD NEUROPSYCHOLOGY

LA English

DT Article

DE Developmental coordination disorder; Motor coordination; Executive

functions; Cognitive development; Motor development

ID BEHAVIORAL SELF-REGULATION; WORKING-MEMORY; COGNITIVE-DEVELOPMENT;

PREADOLESCENT CHILDREN; INDIVIDUAL-DIFFERENCES; POSTURAL INTERFERENCE;

PRESCHOOL-CHILDREN; AEROBIC FITNESS; ACADEMIC SKILLS; YOUNG-CHILDREN

AB Cognitive and motor coordination skills of children with and without motor coordination impairments were examined with a one-year follow-up investigation. Initially, children were between 4 and 6years old. Age-appropriate tests of executive functions (updating, switching, inhibition, interference control), motor coordination (the Movement Assessment Battery for Children-2) and fitness (the Korperkoordinations-Test fur Kinder) were administered in two consecutive years. Several background variables (age, socioeconomic status, medical support, clinical interventions, leisure activities) and potential moderators (nonverbal intelligence, reaction time, visual perception) were controlled. The matched sample consisted of 48 control children and 48 children with motor coordination impairments. The children's executive functions dramatically improved during the one-year period. With regard to motor coordination performance, half of the impaired children caught up to the control children's level (remission group), while the remaining half showed no improvement (persisting group). Compared to the persisting group, the children in the remission group showed markedly better interference control at both measurement points. The correlation between executive functions and motor coordination is significant in the persisting group, but not in the remission group. The results of the study are discussed in the light of the role of executive functions, especially inhibition processes, for the automatization of motor coordination tasks.

C1 [Michel, Eva; Molitor, Sabine; Schneider, Wolfgang] Univ Wurzburg, Dept Psychol, Wurzburg, Germany.

RP Michel, E (通讯作者)，Univ Wurzburg, Dept Psychol 4, Roentgenring 10, D-97070 Wurzburg, Germany.

EM eva.michel@uni-wuerzburg.de

FU German Research Foundation [MI 1717/1-1]

FX This work was supported by the German Research Foundation [grant number

MI 1717/1-1].

CR Alloway TP, 2007, J EXP CHILD PSYCHOL, V96, P20, DOI 10.1016/j.jecp.2006.07.002

Alloway TP, 2007, APPL COGNITIVE PSYCH, V21, P473, DOI 10.1002/acp.1284

Alloway TP, 2009, J LEARN DISABIL-US, V42, P372, DOI 10.1177/0022219409335214

Alloway TP, 2008, J LEARN DISABIL-US, V41, P251, DOI 10.1177/0022219408315815

American Psychiatric Association, 2013, DIAGNOSTIC STAT MANU, DOI DOI 10.1176/APPI.BOOKS.9780890425596

Blair C, 2007, CHILD DEV, V78, P647, DOI 10.1111/j.1467-8624.2007.01019.x

Buttner G., 2008, FEW2 FROSTIGS ENTWIC

Cairney J, 2007, PEDIATR EXERC SCI, V19, P20, DOI 10.1123/pes.19.1.20

CANTELL MH, 1994, ADAPT PHYS ACT Q, V11, P115, DOI 10.1123/apaq.11.2.115

Cantell MH, 2003, HUM MOVEMENT SCI, V22, P413, DOI 10.1016/j.humov.2003.09.002

CATTELL RB, 1997, GRUNDINTELLIGENZTEST

Chaddock L, 2010, DEV NEUROSCI-BASEL, V32, P249, DOI 10.1159/000316648

CHAMBERS ME, 2005, CHILDREN DEV COORDIN, P1

Cousins M, 2003, HUM MOVEMENT SCI, V22, P433, DOI 10.1016/j.humov.2003.09.003

Cummins A, 2005, DEV MED CHILD NEUROL, V47, P437, DOI 10.1017/S001216220500085X

Dewey D, 2002, HUM MOVEMENT SCI, V21, P905, DOI 10.1016/S0167-9457(02)00163-X

DEWEY D, 1994, DEV NEUROPSYCHOL, V10, P265, DOI 10.1080/87565649409540583

Diamond A, 2000, CHILD DEV, V71, P44, DOI 10.1111/1467-8624.00117

Diamond A, 2007, SCIENCE, V318, P1387, DOI 10.1126/science.1151148

Diamond A, 2013, ANNU REV PSYCHOL, V64, P135, DOI 10.1146/annurev-psych-113011-143750

ERIKSEN BA, 1974, PERCEPT PSYCHOPHYS, V16, P143, DOI 10.3758/BF03203267

Fliers EA, 2012, WORLD J BIOL PSYCHIA, V13, P211, DOI 10.3109/15622975.2011.560279

Fuster JM, 2002, J NEUROCYTOL, V31, P373, DOI 10.1023/A:1024190429920

Garon N, 2008, PSYCHOL BULL, V134, P31, DOI 10.1037/0033-2909.134.1.31

Hands B, 2006, EUR J SPEC NEEDS EDU, V21, P447, DOI DOI 10.1080/08856250600956410

Harnishfeger K, 1995, INTERFERERENCE INHIB, P175

Hartmann-Tews I., 2003, ERSTER DTSCH KINDER, P85

Hasselhorn M., 2012, ARBEITSGEDACHTNISTES

Henderson S., 2007, MOVEMENT ASSESSMENT

Hillman CH, 2013, Z SPORTPSYCHOL, V20, P33, DOI 10.1026/1612-5010/a000085

Hillman CH, 2009, DEV PSYCHOL, V45, P114, DOI 10.1037/a0014437

Hoare D., 1994, ADAPT PHYS ACT Q, V11, P158, DOI DOI 10.1123/APAQ.11.2.158

Iivonen S., 2015, EUR J ADAPT PHYS ACT, V8, P18, DOI [10.5507/euj.2015.006, DOI 10.5507/EUJ.2015.006]

KAIL R, 1991, PSYCHOL BULL, V109, P490, DOI 10.1037/0033-2909.109.3.490

Kanioglou A, 2005, PERCEPT MOTOR SKILL, V101, P163, DOI 10.2466/pms.101.1.163-173

Kaplan BJ, 1998, HUM MOVEMENT SCI, V17, P471, DOI 10.1016/S0167-9457(98)00010-4

Kauer M, 2011, PSYCHOL ERZ UNTERR, V58, P173, DOI 10.2378/peu2010.art28d

Kiphard EJ., 2007, KORPERKOORDINATIONST

Koziol LF, 2012, CEREBELLUM, V11, P505, DOI 10.1007/s12311-011-0321-y

Krombholz H., 2012, PSYCHOL ERZIEHUNG UN, V1, P1

Livesey D, 2006, HUM MOVEMENT SCI, V25, P50, DOI 10.1016/j.humov.2005.10.008

Lubans DR, 2010, SPORTS MED, V40, P1019, DOI 10.2165/11536850-000000000-00000

Macnab JJ, 2001, HUM MOVEMENT SCI, V20, P49, DOI 10.1016/S0167-9457(01)00028-8

Mandich A, 2003, BRAIN COGNITION, V51, P346, DOI 10.1016/S0278-2626(03)00039-3

Mandich A, 2002, BRAIN COGNITION, V50, P150, DOI 10.1016/S0278-2626(02)00020-9

Michel E, 2011, CHILD NEUROPSYCHOL, V17, P151, DOI 10.1080/09297049.2010.525501

Michel E, 2011, KINDH ENTWICKL, V20, P49, DOI 10.1026/0942-5403/a000024

Miyake A, 2000, COGNITIVE PSYCHOL, V41, P49, DOI 10.1006/cogp.1999.0734

Miyake A, 2012, CURR DIR PSYCHOL SCI, V21, P8, DOI 10.1177/0963721411429458

Moffitt TE, 2011, P NATL ACAD SCI USA, V108, P2693, DOI 10.1073/pnas.1010076108

Molitor S, 2015, KINDH ENTWICKL, V24, P181, DOI 10.1026/0942-5403/a000174

NICHOLLS JG, 1978, CHILD DEV, V49, P800, DOI 10.1111/j.1467-8624.1978.tb02383.x

Olivier I, 2007, NEUROREPORT, V18, P817, DOI 10.1097/WNR.0b013e3280e129e1

Parker H. E., 2003, DEV MOVEMENT COORDIN, P107

Pennequin V, 2010, BRAIN COGNITION, V74, P107, DOI 10.1016/j.bandc.2010.07.003

Petermann F., 2011, MOVEMENT ASSESSMENT

Piek JP, 2007, DEV MED CHILD NEUROL, V49, P678, DOI 10.1111/j.1469-8749.2007.00678.x

Pless M, 2002, ACTA PAEDIATR, V91, P521, DOI 10.1080/080352502753711632

Polatajko Helene J., 2001, Physical and Occupational Therapy in Pediatrics, V20, P83, DOI 10.1300/J006v20n02_06

Ponitz CC, 2009, DEV PSYCHOL, V45, P605, DOI 10.1037/a0015365

Querne L, 2008, BRAIN RES, V1244, P89, DOI 10.1016/j.brainres.2008.07.066

Remschmidt H., 2006, MULTIAXIALES KLASSIF, V5

Ridler K, 2006, P NATL ACAD SCI USA, V103, P15651, DOI 10.1073/pnas.0602639103

Rigoli D, 2013, HUM MOVEMENT SCI, V32, P1116, DOI 10.1016/j.humov.2013.07.014

Roebers CM, 2014, HUM MOVEMENT SCI, V33, P284, DOI 10.1016/j.humov.2013.08.011

Roebers CM, 2011, EUR J DEV PSYCHOL, V8, P526, DOI 10.1080/17405629.2011.571841

Rothlisberger M, 2010, Z ENTWICKL PADAGOGIS, V42, P99, DOI 10.1026/0049-8637/a000010

Saban MT, 2014, RES DEV DISABIL, V35, P2644, DOI 10.1016/j.ridd.2014.07.002

Schott N., 2004, Z SPORTPSYCHOL, V11, P147, DOI DOI 10.1026/1612-5010.11.4.147

Schott N, 2007, RES Q EXERCISE SPORT, V78, P438

Sergeant JA, 2006, HUM MOVEMENT SCI, V25, P76, DOI 10.1016/j.humov.2005.10.007

Skinner RA, 2001, HUM MOVEMENT SCI, V20, P73, DOI 10.1016/S0167-9457(01)00029-X

Stodden D, 2013, Z SPORTPSYCHOL, V20, P10, DOI 10.1026/1612-5010/a000088

Taylor S, 2007, OTJR-OCCUP PART HEAL, V27, P124, DOI 10.1177/153944920702700402

Tomporowski PD, 2008, EDUC PSYCHOL REV, V20, P111, DOI 10.1007/s10648-007-9057-0

Tsai CL, 2009, CHILD CARE HLTH DEV, V35, P551, DOI 10.1111/j.1365-2214.2009.00974.x

Tsai CL, 2008, HUM MOVEMENT SCI, V27, P649, DOI 10.1016/j.humov.2007.10.002

Vaivre-Douret L, 2011, DEV NEUROPSYCHOL, V36, P614, DOI 10.1080/87565641.2011.560696

Van Waelvelde H, 2004, DEV MED CHILD NEUROL, V46, P661, DOI 10.1017/s0012162204001112

Van Waelvelde H, 2010, DEV MED CHILD NEUROL, V52, pe174, DOI 10.1111/j.1469-8749.2009.03606.x

Visser J, 2003, HUM MOVEMENT SCI, V22, P479, DOI 10.1016/j.humov.2003.09.005

von Suchodoletz A, 2013, EARLY CHILD RES Q, V28, P62, DOI 10.1016/j.ecresq.2012.05.003

Wagner MO, 2011, DIAGNOSTICA, V57, P225, DOI 10.1026/0012-1924/a000051

Wegener B., 1988, KRITIK DES PRESTIGES

Wilson PH, 2013, DEV MED CHILD NEUROL, V55, P217, DOI 10.1111/j.1469-8749.2012.04436.x

Wilson PH, 1998, J CHILD PSYCHOL PSYC, V39, P829, DOI 10.1111/1469-7610.00384

Woollacott M, 2002, GAIT POSTURE, V16, P1, DOI 10.1016/S0966-6362(01)00156-4

Zwicker J, 2013, Z SPORTPSYCHOL, V20, P5, DOI 10.1026/1612-5010/a000087

NR 88

TC 17

Z9 18

U1 7

U2 47

PU ROUTLEDGE JOURNALS, TAYLOR & FRANCIS LTD

PI ABINGDON

PA 2-4 PARK SQUARE, MILTON PARK, ABINGDON OX14 4RN, OXON, ENGLAND

SN 0929-7049

EI 1744-4136

J9 CHILD NEUROPSYCHOL

JI Child Neuropsychol.

PY 2018

VL 24

IS 1

BP 20

EP 45

DI 10.1080/09297049.2016.1223282

PG 26

WC Clinical Neurology

WE Science Citation Index Expanded (SCI-EXPANDED); Social Science Citation Index (SSCI)

SC Neurosciences & Neurology

GA FN1FT

UT WOS:000415733400002

PM 27623087

DA 2022-08-17

ER

PT J

AU Poole, KL

Schmidt, LA

Missiuna, C

Saigal, S

Boyle, MH

Van Lieshout, RJ

AF Poole, Kristie L.

Schmidt, Louis A.

Missiuna, Cheryl

Saigal, Saroj

Boyle, Michael H.

Van Lieshout, Ryan J.

TI Motor coordination and mental health in extremely low birth weight

survivors during the first four decades of life

SO RESEARCH IN DEVELOPMENTAL DISABILITIES

LA English

DT Article

DE Infant; Extremely low birth weight; Premature; Motor skills disorders;

Motor coordination; Attention-deficit/hyperactivity disorder;

Depression; Anxiety; Internalizing; Mental disorders

ID DEFICIT HYPERACTIVITY DISORDER; PRETERM CHILDREN; REGIONAL COHORT;

ATTENTION; AGE; PSYCHOPATHOLOGY; INTERVENTION

AB The co-morbidity of motor coordination and mental health problems is an increasing concern. While links between poor motor coordination and mental health have been examined extensively in individuals born at normal birth weight (NBW; >2500 g), relatively little research has examined these associations in special populations, particularly those born at extremely low birth weight (ELBW; <1000 g). In this study, we examined whether birth weight status (ELBW vs. NBW) moderated associations between motor coordination problems and levels of mental health problems from childhood into the fourth decade of life. The present study utilized the oldest known prospectively followed, population-based cohort of ELBW survivors (n = 151). This group was born between 1977 and 1982 in Ontario, Canada and was compared to a matched group of NBW controls (n = 145). Mental health problems were measured at age 8 using parent and teacher reports, and at age 22-26 and 29-36 using self-reports. Childhood motor coordination was retrospectively reported at age 29-36. In both ELBW and NBW groups, childhood coordination problems were associated with elevated levels of inattention and symptoms of anxiety and depression. However, we observed stronger associations between childhood motor coordination problems and mental health problems in NBW controls at 22-26 and 29-36 years of age than in ELBW survivors. Our findings highlight the importance of recognizing and screening for motor coordination problems not only in vulnerable, at-risk children, but in all children, as motor difficulties appear to be associated with mental health problems well into adult life. (C) 2015 Elsevier Ltd. All rights reserved.

C1 [Poole, Kristie L.] McMaster Univ, Dept Clin Epidemiol & Biostat, Hamilton, ON L8S 4K1, Canada.

[Poole, Kristie L.; Schmidt, Louis A.] McMaster Univ, Dept Psychol Neurosci & Behav, Hamilton, ON L8S 4K1, Canada.

[Missiuna, Cheryl] McMaster Univ, Dept Rehabil Sci, Hamilton, ON L8S 4K1, Canada.

[Saigal, Saroj] McMaster Univ, Dept Pediat, Hamilton, ON L8S 4K1, Canada.

[Boyle, Michael H.; Van Lieshout, Ryan J.] McMaster Univ, Dept Psychiat & Behav Neurosci, Hamilton, ON L8S 4K1, Canada.

RP Poole, KL (通讯作者)，McMaster Univ, Dept Clin Epidemiol & Biostat, 1280 Main St W, Hamilton, ON L8S 4K1, Canada.

EM poolekl@mcmaster.ca

RI Van Lieshout, Ryan/AAI-1316-2021

FU Canadian Institutes of Health Research [2009H00529, MOP42536]; National

Institute of Child Health and Human Development [1-RO1HD40219]; Hospital

for Sick Children Foundation, Toronto, Ontario [ESPM85-201]; EUNICE

KENNEDY SHRIVER NATIONAL INSTITUTE OF CHILD HEALTH &HUMAN DEVELOPMENT

[R01HD040219] Funding Source: NIH RePORTER

FX We thank the many participants and their families for their continued

participation in the study. This research was supported by a Canadian

Institutes of Health Research Team Grant (2009H00529) awarded to LAS, by

operating grants MOP42536 from the Canadian Institutes of Health

Research and 1-RO1HD40219 from the National Institute of Child Health

and Human Development awarded to Dr. Saigal and grant ESPM85-201,

Hospital for Sick Children Foundation, Toronto, Ontario.

CR Achenbach T., 1997, MANUAL YOUNG ADULT S

Achenbach T. M., 1983, MANUAL CHILD BEHAV C

Barkley R. A., 1998, ATTENTION DEFICIT HY

Blondis TA, 1999, PEDIATR CLIN N AM, V46, P899, DOI 10.1016/S0031-3955(05)70162-0

Boyle MH, 2011, PSYCHOL MED, V41, P1763, DOI 10.1017/S0033291710002357

Cairney J, 2013, DEV REV, V33, P224, DOI 10.1016/j.dr.2013.07.002

Cairney J, 2010, CURR OPIN PSYCHIATR, V23, P324, DOI 10.1097/YCO.0b013e32833aa0aa

Davis NM, 2007, DEV MED CHILD NEUROL, V49, P325, DOI 10.1111/j.1469-8749.2007.00325.x

de Kieviet JF, 2009, JAMA-J AM MED ASSOC, V302, P2235, DOI 10.1001/jama.2009.1708

Dewey D, 2002, HUM MOVEMENT SCI, V21, P905, DOI 10.1016/S0167-9457(02)00163-X

Fliers EA, 2010, J DEV BEHAV PEDIATR, V31, P35, DOI 10.1097/DBP.0b013e3181c7227e

Foulder-Hughes LA, 2003, DEV MED CHILD NEUROL, V45, P97, DOI 10.1017/S0012162203000197

Green D, 2006, CHILD CARE HLTH DEV, V32, P741, DOI 10.1111/j.1365-2214.2006.00684.x

Grunau RE, 2004, PEDIATRICS, V114, pE725, DOI 10.1542/peds.2004-0932

Hack M, 2009, J DEV BEHAV PEDIATR, V30, P122, DOI 10.1097/DBP.0b013e31819e6a16

Holsti L, 2002, J DEV BEHAV PEDIATR, V23, P9, DOI 10.1097/00004703-200202000-00002

Indredavik MS, 2004, ARCH DIS CHILD-FETAL, V89, pF445, DOI 10.1136/adc.2003.038943

Johnson S, 2010, J AM ACAD CHILD PSY, V49, P453, DOI 10.1016/j.jaac.2010.02.002

Kirby A, 2010, RES DEV DISABIL, V31, P131, DOI 10.1016/j.ridd.2009.08.010

Lahat A, 2014, FRONT PSYCHOL, V5, DOI 10.3389/fpsyg.2014.00446

Missiuna C, 2014, RES DEV DISABIL, V35, P1198, DOI 10.1016/j.ridd.2014.01.007

Parry T., 1996, AUSTR ED DEV PSYCHOL, V13, P56

Peens A, 2008, CHILD CARE HLTH DEV, V34, P316, DOI 10.1111/j.1365-2214.2007.00803.x

Piek JP, 2007, TWIN RES HUM GENET, V10, P587, DOI 10.1375/twin.10.4.587

Pless M, 2000, ADAPT PHYS ACT Q, V17, P381, DOI 10.1123/apaq.17.4.381

Pyhala R, 2011, J PEDIATR-US, V158, P251, DOI 10.1016/j.jpeds.2010.07.059

Roberts G, 2011, DEV MED CHILD NEUROL, V53, P55, DOI 10.1111/j.1469-8749.2010.03779.x

Saigal S, 2003, PEDIATRICS, V111, P969, DOI 10.1542/peds.111.5.969

SAIGAL S, 1991, J DEV BEHAV PEDIATR, V12, P294

Schmidt LA, 2008, PEDIATRICS, V122, pE181, DOI 10.1542/peds.2007-3747

Sigurdsson E, 2002, AM J PSYCHIAT, V159, P1044, DOI 10.1176/appi.ajp.159.6.1044

Skinner RA, 2001, HUM MOVEMENT SCI, V20, P73, DOI 10.1016/S0167-9457(01)00029-X

Strang-Karlsson S, 2008, AM J PSYCHIAT, V165, P1345, DOI 10.1176/appi.ajp.2008.08010085

SZATMARI P, 1993, DEV PSYCHOPATHOL, V5, P345, DOI 10.1017/S0954579400004454

Van Lieshout RJ, 2015, PEDIATRICS, V135, P452, DOI 10.1542/peds.2014-3143

Volpe JJ, 2009, LANCET NEUROL, V8, P110, DOI 10.1016/S1474-4422(08)70294-1

Waxman J., 2013, PERSONALITY INDIVIDU, V55, P967

Williams J, 2010, DEV MED CHILD NEUROL, V52, P232, DOI 10.1111/j.1469-8749.2009.03544.x

Wocadlo C, 2008, EARLY HUM DEV, V84, P769, DOI 10.1016/j.earlhumdev.2008.06.001

NR 39

TC 16

Z9 16

U1 1

U2 19

PU PERGAMON-ELSEVIER SCIENCE LTD

PI OXFORD

PA THE BOULEVARD, LANGFORD LANE, KIDLINGTON, OXFORD OX5 1GB, ENGLAND

SN 0891-4222

J9 RES DEV DISABIL

JI Res. Dev. Disabil.

PD AUG-SEP

PY 2015

VL 43-44

BP 87

EP 96

DI 10.1016/j.ridd.2015.06.004

PG 10

WC Education, Special; Rehabilitation

WE Social Science Citation Index (SSCI)

SC Education & Educational Research; Rehabilitation

GA CP4TS

UT WOS:000359876100010

PM 26163481

DA 2022-08-17

ER

PT J

AU Di Brina, C

Averna, R

Rampoldi, P

Rossetti, S

Penge, R

AF Di Brina, Carlo

Averna, Roberto

Rampoldi, Paola

Rossetti, Serena

Penge, Roberta

TI Reading and Writing Skills in Children With Specific Learning

Disabilities With and Without Developmental Coordination Disorder

SO MOTOR CONTROL

LA English

DT Article

DE DCD; learning disabilities; movement; poor handwriting; reading

ID MOVEMENT ASSESSMENT BATTERY; FINE MOTOR DEFICIENCIES; AUTOMATIZATION

PROCESS; DYSLEXIA; DCD; PERFORMANCE; DYSGRAPHIA; PATTERNS

AB This pilot study is to investigate the influence of a developmental coordination disorder (DCD) comorbidity in a group of children with learning disability (LD). Reading and writing were assessed to investigate if the coexistence of a motor impairment can worsen writing quality, speed, and reading accuracy. A sample of 33 LD children (aged 7-11 years) was divided in two subgroups, on the base of their scores on the Movement Assessment Battery for Children: LD-only (n = 14) and LD with a comorbidity for DCD (LD-DCD, n = 19). No differences were found in handwriting speed, but significant differences were found in handwriting quality: LD-DCD children showed a worst performance. Reading words and nonwords accuracy was more impaired in LD-only children than in LD-DCD children. Group differences suggest a poorer phonological decoding of the LD-only sample, whereas worst cursive handwriting legibility scores are typical of the motor-impaired subgroup.

C1 [Di Brina, Carlo; Averna, Roberto; Rampoldi, Paola; Penge, Roberta] Sapienza Univ Rome, Dept Pediat & Child Neuropsychiat, Rome, Italy.

[Averna, Roberto] IRCCS, Bambino Gestu Childrens Hosp, Child Neuropsychiat Unit, Rome, Italy.

[Rossetti, Serena] Sapienza Univ Rome, Dept Clin Psychol, Rome, Italy.

RP Di Brina, C (通讯作者)，Sapienza Univ Rome, Dept Pediat & Child Neuropsychiat, Rome, Italy.

EM cdibrina@tiscali.it

RI Averna, Roberto/AAA-6090-2020

OI Di Brina, Carlo/0000-0003-0985-6086

CR American Psychiatric Association, 2013, DIAGNOSTIC STAT MANU, DOI [10.1176/appi.books.9780890425596, DOI 10.1176/APPI.BOOKS.9780890425596]

[Anonymous], [No title captured]

Beery K.E., 2004, BEERY VMI BEERY BUKT

Berninger VW, 2008, J SCHOOL PSYCHOL, V46, P1, DOI 10.1016/j.jsp.2006.11.008

Biotteau M, 2017, EUR J PAEDIATR NEURO, V21, P286, DOI 10.1016/j.ejpn.2016.07.025

Biotteau M, 2015, HUM MOVEMENT SCI, V43, P78, DOI 10.1016/j.humov.2015.07.005

Capellini Simone Aparecida, 2010, Pró-Fono R. Atual. Cient., V22, P201, DOI 10.1590/S0104-56872010000300008

Chow SMK, 2003, AM J OCCUP THER, V57, P574, DOI 10.5014/ajot.57.5.574

Coltheart M, 2001, PSYCHOL REV, V108, P204, DOI 10.1037//0033-295X.108.1.204

Cornoldi C, 1981, PROVE DI LETTUR

D'Angiulli A, 2003, J LEARN DISABIL-US, V36, P48, DOI 10.1177/00222194030360010601

Das T, 2011, NEUROIMAGE, V54, P1476, DOI 10.1016/j.neuroimage.2010.09.022

Dohla D, 2016, FRONT PSYCHOL, V6, DOI 10.3389/fpsyg.2015.02045

Drummond CR, 2005, ARCH CLIN NEUROPSYCH, V20, P171, DOI 10.1016/j.acn.2004.05.001

Fortes IS, 2016, EUR CHILD ADOLES PSY, V25, P195, DOI 10.1007/s00787-015-0708-2

Geertsen SS, 2016, PLOS ONE, V11, DOI 10.1371/journal.pone.0161960

Getchell N, 2007, PERCEPT MOTOR SKILL, V105, P207, DOI 10.2466/PMS.105.1.207-214

Gori S, 2014, FRONT HUM NEUROSCI, V8, DOI 10.3389/fnhum.2014.00460

Grissmer D, 2010, DEV PSYCHOL, V46, P1008, DOI 10.1037/a0020104

Hamstra-Bletz E, 1987, BEKNOPTEBEOORDELINGS

Haslum MN, 2007, DYSLEXIA, V13, P257, DOI 10.1002/dys.350

HENDERSON SE, 1992, MOVEMENT ASSESSMENT

Jongmans MJ, 2003, J LEARN DISABIL-US, V36, P528, DOI 10.1177/00222194030360060401

Kadesjo B, 1998, DEV MED CHILD NEUROL, V40, P796

Kaplan BJ, 1998, HUM MOVEMENT SCI, V17, P471, DOI 10.1016/S0167-9457(98)00010-4

Lam SST, 2011, RES DEV DISABIL, V32, P1745, DOI 10.1016/j.ridd.2011.03.001

LAMME LL, 1979, YOUNG CHILDREN, V35, P20

LYYTINEN H, 1989, LEARNING DISABILITIE, V1, P35

Mammarella IC, 2014, CHILD NEUROPSYCHOL, V20, P255, DOI 10.1080/09297049.2013.796920

Nicolson RI, 2011, CORTEX, V47, P117, DOI 10.1016/j.cortex.2009.08.016

Pagliarini E, 2015, HUM MOVEMENT SCI, V42, P161, DOI 10.1016/j.humov.2015.04.012

Pape-Neumann J, 2015, ACTA NEUROBIOL EXP, V75, P80

Peters LHJ, 2013, DEV MED CHILD NEUROL, V55, P59, DOI 10.1111/dmcn.12309

Pitchford NJ, 2016, FRONT PSYCHOL, V7, DOI 10.3389/fpsyg.2016.00783

REGEHR SM, 1988, PEDIATRICS, V82, P204

Ruffino M, 2014, FRONT HUM NEUROSCI, V8, DOI 10.3389/fnhum.2014.00331

Sartori G, 2007, DDE2 BATTERIA VALUTA

SILVA P A, 1982, Journal of Human Movement Studies, V8, P187

Smits-Engelsman BCM, 2003, HUM MOVEMENT SCI, V22, P495, DOI 10.1016/j.humov.2003.09.006

Smits-Engelsman BCM, 2001, HUM MOVEMENT SCI, V20, P161, DOI 10.1016/S0167-9457(01)00033-1

Stoodley CJ, 2011, CORTEX, V47, P101, DOI 10.1016/j.cortex.2009.10.005

Tseng MH, 2000, AM J OCCUP THER, V54, P83, DOI 10.5014/ajot.54.1.83

TSENG MH, 1993, AM J OCCUP THER, V47, P919, DOI 10.5014/ajot.47.10.919

Wilson PH, 2005, J CHILD PSYCHOL PSYC, V46, P806, DOI 10.1111/j.1469-7610.2005.01409.x

Wimmer H, 2010, DYSLEXIA, V16, P283, DOI 10.1002/dys.411

Wolff PH, 1995, AM J MED GENET, V60, P494, DOI 10.1002/ajmg.1320600604

NR 46

TC 8

Z9 8

U1 3

U2 28

PU HUMAN KINETICS PUBL INC

PI CHAMPAIGN

PA 1607 N MARKET ST, PO BOX 5076, CHAMPAIGN, IL 61820-2200 USA

SN 1087-1640

EI 1543-2696

J9 MOTOR CONTROL

JI Motor Control

PD OCT

PY 2018

VL 22

IS 4

BP 391

EP 405

DI 10.1123/mc.2016-0006

PG 15

WC Neurosciences; Sport Sciences

WE Science Citation Index Expanded (SCI-EXPANDED); Social Science Citation Index (SSCI)

SC Neurosciences & Neurology; Sport Sciences

GA HE5CR

UT WOS:000453389700002

PM 29488824

DA 2022-08-17

ER

PT J

AU Blanchet, M

Assaiante, C

AF Blanchet, Marieve

Assaiante, Christine

TI Specific Learning Disorder in Children and Adolescents, a Scoping Review

on Motor Impairments and Their Potential Impacts

SO CHILDREN-BASEL

LA English

DT Review

DE learning disabilities; motor development; sensorimotor representations;

locomotion; posture; gross motor skills; fine motor skills; children and

adolescents

ID DEVELOPMENTAL COORDINATION DISORDER; PHYSICAL-ACTIVITY; POSTURAL

CONTROL; CARDIORESPIRATORY FITNESS; ACADEMIC-ACHIEVEMENT; EXECUTIVE

FUNCTIONS; VISUAL INFORMATION; DYSLEXIC-CHILDREN; INTERNAL-MODELS;

SKILLS

AB Mastering motor skills is important for children to achieve functional mobility and participate in daily activities. Some studies have identified that students with specific learning disorders (SLD) could have impaired motor skills; however, this postulate and the potential impacts remain unclear. The purpose of the scoping review was to evaluate if SLD children have motor impairments and examine the possible factors that could interfere with this assumption. The sub-objective was to investigate the state of knowledge on the lifestyle behavior and physical fitness of participants with SLD and to discuss possible links with their motor skills. Our scoping review included preregistration numbers and the redaction conformed with the PRISMA guidelines. A total of 34 studies published between 1990 and 2022 were identified. The results of our scoping review reflected that students with SLD have poorer motor skills than their peers. These motor impairments are exacerbated by the complexity of the motor activities and the presence of comorbidities. These results support our sub-objective and highlight the link between motor impairments and the sedentary lifestyle behavior of SLDs. This could lead to deteriorating health and motor skills due to a lack of motor experience, meaning that this is not necessarily a comorbidity. This evidence emphasizes the importance of systematic clinical motor assessments and physical activity adaptations.

C1 [Blanchet, Marieve] Univ Quebec Montreal, Dept Sci Activ Phys, Lab Rech Motr Enfant, 141 Av President Kennedy, Montreal, PQ H2X 1Y4, Canada.

[Assaiante, Christine] AMU, CNRS, UMR 7291, Federat 3C,LNC,Ctr St Charles, Pole 3C,Case C,3 Pl Victor Hugo, F-13331 Marseille, France.

RP Blanchet, M (通讯作者)，Univ Quebec Montreal, Dept Sci Activ Phys, Lab Rech Motr Enfant, 141 Av President Kennedy, Montreal, PQ H2X 1Y4, Canada.

EM blanchet.marieve@uqam.ca; christine.assaiante@univ-amu.fr

FU Fonds de Recherche du Quebec -Sante [296315]; "Table pour un mode de vie

physiquement actif" (TMVPA); Direction generale de sante publique du

ministere de la Sante et des Services sociaux du Quebec

FX This research was funded by the "Direction generale de sante publique du

ministere de la Sante et des Services sociaux du Quebec: N/A; Fonds de

Recherche du Quebec -Sante: 296315" and was supported by the "Table pour

un mode de vie physiquement actif" (TMVPA).

CR ADOLPH KE, 1993, CHILD DEV, V64, P1158, DOI 10.2307/1131332

American Psychiatric Association, 1994, DIAGNOSTIC STAT MANU

Assaiante C, 2014, NEUROPHYSIOL CLIN, V44, P3, DOI 10.1016/j.neucli.2013.10.125

Assaiante C., 2020, ANAE, V168, P535

Baharudin NS, 2020, MALAYS J MED SCI, V27, P21, DOI 10.21315/mjms2020.27.2.3

Barela JA, 2020, NEUROSCI LETT, V725, DOI 10.1016/j.neulet.2020.134890

Barela JA, 2011, RES DEV DISABIL, V32, P1814, DOI 10.1016/j.ridd.2011.03.011

Barnett LM, 2016, SPORTS MED, V46, P1663, DOI 10.1007/s40279-016-0495-z

Barnett LM, 2009, J ADOLESCENT HEALTH, V44, P252, DOI 10.1016/j.jadohealth.2008.07.004

Beaton AA, 2002, CORTEX, V38, P479, DOI 10.1016/S0010-9452(08)70017-0

Blanchet M, 2022, LIMITS STABILITY POS

Blanchet M., 2022, J PEDIAT EXERC SCI

Bluechardt M., 1996, BMC SPORTS SCI MED R, V7, P133, DOI [10.1080/15438629609512077, DOI 10.1080/15438629609512077]

Boutros G.H., 2020, PEDIATR NEONATOL, V9, P1

Brookes RL, 2010, DYSLEXIA, V16, P358, DOI 10.1002/dys.420

BRUININKS VL, 1977, PERCEPT MOTOR SKILL, V44, P1131, DOI 10.2466/pms.1977.44.3c.1131

Brymer E., 2010, AUSTR J OUTDOOR ED, V14, P33, DOI DOI 10.1007/BF03400903

Bucci MP, 2017, INT J DEV NEUROSCI, V61, P51, DOI 10.1016/j.ijdevneu.2017.06.010

Burton N, 2016, LEARN DISABIL ELF

Cairney J, 2007, PEDIATR EXERC SCI, V19, P20, DOI 10.1123/pes.19.1.20

Cairney J, 2017, J SCI MED SPORT, V20, P380, DOI 10.1016/j.jsams.2016.08.025

Canadian Attention Deficit Hyperactivity Disorder Resource Alliance (CADDRA), 2011, CAN ADHD PRACT GUID

Cantell M, 2008, HUM MOVEMENT SCI, V27, P344, DOI 10.1016/j.humov.2008.02.007

Capellini Simone Aparecida, 2010, Pró-Fono R. Atual. Cient., V22, P201, DOI 10.1590/S0104-56872010000300008

Cignetti F., 2020, CEREB CORTEX, V1, DOI [10.1093/texcom/tgaa011, DOI 10.1093/TEXCOM/TGAA011]

Cignetti F, 2018, RES DEV DISABIL, V76, P25, DOI 10.1016/j.ridd.2018.03.001

Cook BG, 2015, J LEARN DISABIL-US, V48, P563, DOI 10.1177/0022219413518582

Craity B.J., 1972, PHYS EXPRESSIONS INT

Eckert MA, 2003, BRAIN, V126, P482, DOI 10.1093/brain/awg026

Emerson E, 2011, TIZARD LEARN DISABIL, V16, P42, DOI 10.5042/tldr.2011.0008

Ensrud-Skraastad OK, 2020, SPORTS, V8, DOI 10.3390/sports8090120

Fawcett AJ, 1996, ANN DYSLEXIA, V46, P259, DOI 10.1007/BF02648179

FILIPEK PA, 1995, J CHILD NEUROL, V10, pS62, DOI 10.1177/08830738950100S113

Fontan A, 2017, DEV COGN NEUROS-NETH, V24, P118, DOI 10.1016/j.dcn.2017.02.010

Foweather L, 2015, J SCI MED SPORT, V18, P691, DOI 10.1016/j.jsams.2014.09.014

Galli M, 2011, RES DEV DISABIL, V32, P1004, DOI 10.1016/j.ridd.2011.01.051

Gashaj V, 2019, J EXP CHILD PSYCHOL, V182, P187, DOI 10.1016/j.jecp.2019.01.021

Getchell N, 2007, PERCEPT MOTOR SKILL, V105, P207, DOI 10.2466/PMS.105.1.207-214

Gooch D, 2014, J CHILD PSYCHOL PSYC, V55, P237, DOI 10.1111/jcpp.12139

Gouleme N, 2017, GAIT POSTURE, V56, P141, DOI 10.1016/j.gaitpost.2017.04.039

Gouleme N, 2015, CLIN NEUROPHYSIOL, V126, P1370, DOI 10.1016/j.clinph.2014.10.016

Grigorenko EL, 2020, AM PSYCHOL, V75, P37, DOI 10.1037/amp0000452

Haoues M.M.M., 2021, REV PODOL, V17, P18, DOI [10.1016/j.revpod.2021.02.005, DOI 10.1016/J.REVPOD.2021.02.005]

Haubenstricker J.L., 1982, J PHYS EDUC RECREAT, V53, P41, DOI [10.1080/07303084.1982.10629384, DOI 10.1080/07303084.1982.10629384]

Hazzaa N., 2021, AIN SHAMS MED J, V72, P97, DOI [10.21608/asmj.2021.167357, DOI 10.21608/ASMJ.2021.167357]

Hollins S, 2013, BMJ-BRIT MED J, V346, DOI 10.1136/bmj.f3421

Humphrey N., 2002, J RES SP C ED NEEDS, P2, DOI DOI 10.1111/J.1471-3802.2002.00163.X

Hussein Z.A., 2020, DRUG INVENT TODAY, V14, P303

HYND GW, 1989, PSYCHOL BULL, V106, P447, DOI 10.1037/0033-2909.106.3.447

Iivonen KS, 2013, PERCEPT MOTOR SKILL, V117, P627, DOI 10.2466/10.06.PMS.117x22z7

Ismail FY, 2017, EUR J PAEDIATR NEURO, V21, P23, DOI 10.1016/j.ejpn.2016.07.007

Iversen S, 2005, DYSLEXIA, V11, P217, DOI 10.1002/dys.297

Johnston MV, 2009, DEV DISABIL RES REV, V15, P94, DOI 10.1002/ddrr.64

Jongmans MJ, 2003, J LEARN DISABIL-US, V36, P528, DOI 10.1177/00222194030360060401

Jover M., 2013, ENFANCE, V4, P323, DOI DOI 10.4074/S0013754513004023

Kalogeraki E, 2017, PLOS ONE, V12, DOI 10.1371/journal.pone.0186999

Kaplan BJ, 1998, HUM MOVEMENT SCI, V17, P471, DOI 10.1016/S0167-9457(98)00010-4

Kapoula Z, 2007, J NEUROL, V254, P1174, DOI 10.1007/s00415-006-0460-0

Katartzi ES, 2011, RES DEV DISABIL, V32, P2674, DOI 10.1016/j.ridd.2011.06.005

Kawato M, 1998, NOVART FDN SYMP, V218, P291

Kirby A, 2008, J RES SPEC EDUC NEED, V8, P120, DOI 10.1111/j.1471-3802.2008.00111.x

KOPPITZ EM, 1958, J CLIN PSYCHOL, V14, P292, DOI 10.1002/1097-4679(195807)14:3<292::AID-JCLP2270140321>3.0.CO;2-O

Kulkarni Madhuri, 2001, Indian Journal of Pediatrics, V68, P539, DOI 10.1007/BF02723250

Kulkarni SK, 2001, DRUG TODAY, V37, P97, DOI 10.1358/dot.2001.37.2.834327

Kumari P., 2016, CLIN EXP PSYCHOL, V2, P1, DOI [10.4172/2471-2701.1000118, DOI 10.4172/2471-2701.1000118]

Lam SST, 2011, RES DEV DISABIL, V32, P1745, DOI 10.1016/j.ridd.2011.03.001

Laprevotte J, 2021, SCI REP-UK, V11, DOI 10.1038/s41598-020-79612-4

LeGear M, 2012, INT J BEHAV NUTR PHY, V9, DOI 10.1186/1479-5868-9-29

Legrand A, 2012, PLOS ONE, V7, DOI 10.1371/journal.pone.0035301

Leonard CM, 2002, J COMMUN DISORD, V35, P501, DOI 10.1016/S0021-9924(02)00120-X

Lipowska M, 2011, MED SCI MONITOR, V17, pCR216, DOI 10.12659/MSM.881718

Lopes L, 2013, HUM MOVEMENT SCI, V32, P9, DOI 10.1016/j.humov.2012.05.005

Lubans DR, 2010, SPORTS MED, V40, P1019, DOI 10.2165/11536850-000000000-00000

Lukasova K, 2016, FRONT PSYCHOL, V7, DOI 10.3389/fpsyg.2016.00987

Luo Z, 2007, BRIT J DEV PSYCHOL, V25, P595, DOI 10.1348/026151007X185329

Macdonald K, 2018, INT J ENV RES PUB HE, V15, DOI 10.3390/ijerph15081603

Magistro D., 2015, COGN BRAIN BEHAV, V19, P149

MALOY CF, 1979, J SCHOOL PSYCHOL, V17, P213, DOI 10.1016/0022-4405(79)90002-5

Mancini VO, 2018, BRIT J EDUC PSYCHOL, V88, P363, DOI 10.1111/bjep.12187

Marchand-Krynski ME, 2017, PLOS ONE, V12, DOI 10.1371/journal.pone.0177490

Marchetti R., 2021, ANAE, V33, P689

Margari L, 2013, BMC NEUROL, V13, DOI 10.1186/1471-2377-13-198

McGuigan SM, 1995, J INTELL DISABIL RES, V39, P527, DOI 10.1111/j.1365-2788.1995.tb00573.x

McPhillips M, 2004, DYSLEXIA, V10, P316, DOI 10.1002/dys.282

MELTZOFF AN, 1977, SCIENCE, V198, P75, DOI 10.1126/science.198.4312.75

Miall RC, 1996, NEURAL NETWORKS, V9, P1265, DOI 10.1016/S0893-6080(96)00035-4

Moe-Nilssen R, 2003, EXP BRAIN RES, V150, P237, DOI 10.1007/s00221-003-1450-4

Morasso PG, 1999, J NEUROPHYSIOL, V82, P1622, DOI 10.1152/jn.1999.82.3.1622

Naz S, 2019, PSYCHIAT CLIN PSYCH, V29, P674, DOI 10.1080/24750573.2019.1589174

Nicolson RI, 1999, LANCET, V353, P1662, DOI 10.1016/S0140-6736(98)09165-X

Nicolson RI, 2001, TRENDS NEUROSCI, V24, P508, DOI 10.1016/S0166-2236(00)01896-8

NICOLSON RI, 1990, COGNITION, V35, P159, DOI 10.1016/0010-0277(90)90013-A

Niechwiej-Szwedo E, 2017, HUM MOVEMENT SCI, V56, P1, DOI 10.1016/j.humov.2017.10.014

Okuda P.M.M., 2014, PSYCHOL RES-PSYCH FO, V4, P31

Okuda PMM, 2015, PROCD SOC BEHV, V174, P1330, DOI 10.1016/j.sbspro.2015.01.755

Okuda Paola Matiko Martins, 2011, J. Soc. Bras. Fonoaudiol., V23, P351, DOI 10.1590/S2179-64912011000400010

Paillard T, 2017, NEUROSCI BIOBEHAV R, V72, P129, DOI 10.1016/j.neubiorev.2016.11.015

Pieters S, 2012, LEARN INDIVID DIFFER, V22, P498, DOI 10.1016/j.lindif.2012.03.014

Poblano A, 2002, ARCH MED RES, V33, P485, DOI 10.1016/S0188-4409(02)00397-1

Pope D.H., 2003, PSYCHOL P BRIT PSYCH

Pozzo T, 2006, NEUROSCI LETT, V403, P211, DOI 10.1016/j.neulet.2006.03.049

PYFER JL, 1972, PERCEPT MOTOR SKILL, V35, P291, DOI 10.2466/pms.1972.35.1.291

Quercia P, 2005, J FR OPHTALMOL, V28, P713, DOI 10.1016/S0181-5512(05)80983-0

Rae C, 2002, NEUROPSYCHOLOGIA, V40, P1285, DOI 10.1016/S0028-3932(01)00216-0

Ramus F, 2003, J CHILD PSYCHOL PSYC, V44, P712, DOI 10.1111/1469-7610.00157

Razuk M, 2020, DYSLEXIA, V26, P52, DOI 10.1002/dys.1641

Razuk M, 2018, PLOS ONE, V13, DOI 10.1371/journal.pone.0198001

Razuk M, 2014, RES DEV DISABIL, V35, P1988, DOI 10.1016/j.ridd.2014.03.045

Reiter A, 2005, DYSLEXIA, V11, P116, DOI 10.1002/dys.289

Renshaw I, 2019, PHYS EDUC SPORT PEDA, V24, P103, DOI 10.1080/17408989.2018.1552676

Rintala P, 2003, PERCEPT MOTOR SKILL, V97, P755, DOI 10.2466/PMS.97.7.755-762

Rivilis I, 2011, RES DEV DISABIL, V32, P894, DOI 10.1016/j.ridd.2011.01.017

Rizzolatti G, 2001, NAT REV NEUROSCI, V2, P661, DOI 10.1038/35090060

Robinson LE, 2015, SPORTS MED, V45, P1273, DOI 10.1007/s40279-015-0351-6

Robinson LE, 2012, RES Q EXERCISE SPORT, V83, P20, DOI 10.1080/02701367.2012.10599821

Rochelle KSH, 2006, J CHILD PSYCHOL PSYC, V47, P1159, DOI 10.1111/j.1469-7610.2006.01641.x

Soares Daniela Bento, 2015, Rev. CEFAC, V17, P1132, DOI 10.1590/1982-0216201517420014

Sommeijer JP, 2017, NAT NEUROSCI, V20, P1715, DOI 10.1038/s41593-017-0002-3

Son SH, 2006, MERRILL PALMER QUART, V52, P755, DOI 10.1353/mpq.2006.0033

Steenbergen-Hu S, 2020, GIFTED CHILD QUART, V64, P132, DOI 10.1177/0016986220908601

Stein J, 2001, Dyslexia, V7, P12, DOI 10.1002/dys.186

Stodden DF, 2008, QUEST, V60, P290, DOI 10.1080/00336297.2008.10483582

Stoodley CJ, 2000, NEUROSCI LETT, V295, P13, DOI 10.1016/S0304-3940(00)01574-3

Suggate S, 2018, J RES READ, V41, P1, DOI 10.1111/1467-9817.12081

Suhaili I, 2019, Med J Malaysia, V74, P34

Taube W, 2008, ACTA PHYSIOL, V193, P101, DOI 10.1111/j.1748-1716.2008.01850.x

Ulrich DA, 1985, TEST GROSS MOTOR DEV

Vaivre-Douret L., 2021, NEUROPSYCHIATR LENFA, V69, P311, DOI [10.1016/j.neurenf.2021.07.002, DOI 10.1016/J.NEURENF.2021.07.002]

Van Hecke R, 2019, J AUTISM DEV DISORD, V49, P3328, DOI 10.1007/s10803-019-04059-0

Viana AR, 2013, PLOS ONE, V8, DOI 10.1371/journal.pone.0072719

Vuijk PJ, 2011, J LEARN DISABIL-US, V44, P276, DOI 10.1177/0022219410378446

Westendorp M, 2014, RES DEV DISABIL, V35, P357, DOI 10.1016/j.ridd.2013.11.018

Westendorp M, 2011, RES DEV DISABIL, V32, P2773, DOI 10.1016/j.ridd.2011.05.032

Wilson M, 2005, PSYCHOL BULL, V131, P460, DOI 10.1037/0033-2909.131.3.460

WOLFF PH, 1990, DEV PSYCHOL, V26, P349, DOI 10.1037/0012-1649.26.3.349

Wolpert DM, 1998, TRENDS COGN SCI, V2, P338, DOI 10.1016/S1364-6613(98)01221-2

World Health Organization (WHO), US

Wright BA, 2000, CURR OPIN NEUROBIOL, V10, P482, DOI 10.1016/S0959-4388(00)00119-7

Zadina JN, 2006, J CHILD NEUROL, V21, P922, DOI 10.1177/08830738060210110201

Zoia S, 2006, CHILD CARE HLTH DEV, V32, P613, DOI 10.1111/j.1365-2214.2006.00697.x

NR 140

TC 0

Z9 0

U1 3

U2 3

PU MDPI

PI BASEL

PA ST ALBAN-ANLAGE 66, CH-4052 BASEL, SWITZERLAND

EI 2227-9067

J9 CHILDREN-BASEL

JI Children-Basel

PD JUN

PY 2022

VL 9

IS 6

AR 892

DI 10.3390/children9060892

PG 29

WC Pediatrics

WE Science Citation Index Expanded (SCI-EXPANDED)

SC Pediatrics

GA 2M2SG

UT WOS:000817555300001

PM 35740829

OA gold, Green Published

DA 2022-08-17

ER

PT J

AU Mukherjee, S

Jamie, LCT

Fong, LH

AF Mukherjee, Swarup

Jamie, Lye Ching Ting

Fong, Leong Hin

TI Fundamental Motor Skill Proficiency of 6-to 9-Year-Old Singaporean

Children

SO PERCEPTUAL AND MOTOR SKILLS

LA English

DT Article

DE children; fundamental movement skills; motor readiness; lower primary

school level; Singapore

ID PRIMARY-SCHOOL CHILDREN; MOVEMENT SKILLS; PHYSICAL-ACTIVITY; PLAYGROUND

GAMES; PERFORMANCE; ADOLESCENTS; FITNESS; MASTERY; PROGRAM

AB Fundamental movement proficiency (FMS) is most successfully acquired during early school years. This cross-sectional study assessed FMS proficiency in Singaporean children at the start of and following 2.5 years of primary school physical education (PE). Participants were 244 children from Primary 1 and 3 levels. Fundamental movement skills (FMS) were assessed with the Test of Gross Motor Development-Second Edition (TGMD-2) that includes locomotor (LOCO) and object control (OC) subtests. Most children were rated "average'' and "below average'' for LOCO skills but "poor'' and "below average'' for OC skills without significant gender differences on either subtest or overall FMS proficiency and without FMS mastery. These young Singaporean children failed to exhibit age-appropriate FMS proficiency despite early PE exposure, and they demonstrated lags in FMS compared with the TGMD-2 U.S. normative sample. We discuss implications for sports competence perception, difficulty in coping with later movement learning expectations and reduced later motivation to participate in PE and play. We also discuss implications for preschool and lower primary school PE curricula with a particular focus on both OC skills and LOCO skills requiring muscular fitness like hopping and jumping.

C1 [Mukherjee, Swarup; Fong, Leong Hin] Nanyang Technol Univ, Natl Inst Educ, Phys Educ & Sports Sci Acad Grp, 1 Nanyang Walk, Singapore 637616, Singapore.

[Jamie, Lye Ching Ting] Singapore Polytech, Sch Chem & Life Sci, Singapore, Singapore.

RP Mukherjee, S (通讯作者)，Nanyang Technol Univ, Natl Inst Educ, Phys Educ & Sports Sci Acad Grp, 1 Nanyang Walk, Singapore 637616, Singapore.

EM swarup.mukherjee@nie.edu.sg

RI Mukherjee, Swarup/R-5687-2016

OI Mukherjee, Swarup/0000-0001-7626-1299

FU Ministry of Education Singapore's Education Research Development Fund

[OER 41/12]; North Vista Primary School in Singapore; Zhangde Primary

School in Singapore; Fairfield Methodist School (Primary) in Singapore;

Changkat Primary School in Singapore

FX The author(s) disclosed receipt of the following financial support for

the research, authorship, and/or publication of this article: We wish to

acknowledge the Ministry of Education Singapore's Education Research

Development Fund Grant no. OER 41/12 SM to fund this study and the

support from North Vista Primary School, Zhangde Primary School,

Fairfield Methodist School (Primary), and Changkat Primary School in

Singapore.

CR [Anonymous], 2012, IBM SPSS STAT WIND V

Barnett LM, 2008, INT J BEHAV NUTR PHY, V5, DOI 10.1186/1479-5868-5-40

Barnett LM, 2009, J ADOLESCENT HEALTH, V44, P252, DOI 10.1016/j.jadohealth.2008.07.004

BARTON G. V., 1999, TEACHING ELEMENTARY, V10, P9

Blatchford P, 2003, BRIT J DEV PSYCHOL, V21, P481, DOI 10.1348/026151003322535183

Booth M, 1997, NSW SCH FITNESS PHYS

Bouffard M, 1996, ADAPT PHYS ACT Q, V13, P61, DOI 10.1123/apaq.13.1.61

Branta C, 1984, Exerc Sport Sci Rev, V12, P467

BUTCHER JE, 1989, J HUM MOVEMENT STUD, V16, P27

Colvin A., 2000, TEACHING NUTS BOLTS

Faigenbaum AD, 2013, J STRENGTH COND RES, V27, P323, DOI 10.1519/JSC.0b013e31827e135b

Fisher A, 2005, MED SCI SPORT EXER, V37, P684, DOI 10.1249/01.MSS.0000159138.48107.7D

Gallahue D.L., 2006, UNDERSTANDING MOTOR

Gilbey H, 2010, PEDIATR EXERC SCI, V7, P26, DOI 10.1123/pes.7.1.26

Goncalves H, 2007, REV PANAM SALUD PUBL, V22, P246, DOI 10.1590/S1020-49892007000900004

Goodway JD, 2003, ADAPT PHYS ACT Q, V20, P298, DOI 10.1123/apaq.20.3.298

Hardy LL, 2013, MED SCI SPORT EXER, V45, P1965, DOI 10.1249/MSS.0b013e318295a9fc

Harvey WJ, 2007, J ABNORM CHILD PSYCH, V35, P871, DOI 10.1007/s10802-007-9140-5

Holfelder B, 2014, PSYCHOL SPORT EXERC, V15, P382, DOI 10.1016/j.psychsport.2014.03.005

Ingle L, 2006, J SPORT SCI, V24, P987, DOI 10.1080/02640410500457117

Lopes VP, 2012, EUR J SPORT SCI, V12, P384, DOI 10.1080/17461391.2011.566368

Lubans DR, 2010, SPORTS MED, V40, P1019, DOI 10.2165/11536850-000000000-00000

Malina RM., 2004, GROWTH MATURATION PH, V2nd ed

Ministry of Education Singapore (MOE), 2006, PHYS SPORT ED

Morgan PJ, 2008, RES Q EXERCISE SPORT, V79, P506, DOI 10.1080/02701367.2008.10599517

Okely AD, 2004, J SCI MED SPORT, V7, P358, DOI 10.1016/S1440-2440(04)80031-8

Okely AD, 2004, RES Q EXERCISE SPORT, V75, P238, DOI 10.1080/02701367.2004.10609157

Okely AD, 2001, PEDIATR EXERC SCI, V13, P380, DOI 10.1123/pes.13.4.380

Olrich T. W., 2002, J PHYS ED RECREATION, V73, P26, DOI [10.1080/07303084.2002.10607843, DOI 10.1080/07303084.2002.10607843]

Pang AWY, 2009, RES SPORTS MED, V17, P125, DOI 10.1080/15438620902897516

Pellegrini AD, 2004, SOC DEV, V13, P107, DOI 10.1111/j.1467-9507.2004.00259.x

Southall JE, 2004, PEDIATR EXERC SCI, V16, P15, DOI 10.1123/pes.16.1.15

Spessato BC, 2013, EARLY CHILD DEV CARE, V183, P916, DOI 10.1080/03004430.2012.689761

Ting JLC, 2015, J PHYS ACT HEALTH, V12, P1213, DOI 10.1123/jpah.2014-0207

Tveter AT, 2010, GAIT POSTURE, V32, P259, DOI 10.1016/j.gaitpost.2010.05.009

Ulrich D.A, 2000, TEST GROSS MOTOR DEV, V2nd ed

Valentini NC, 2007, J SPORT EXERCISE PSY, V29, pS47

van Beurden E, 2002, J SCI MED SPORT, V5, P244, DOI 10.1016/S1440-2440(02)80010-X

Vandaele B, 2011, EUR PHYS EDUC REV, V17, P3, DOI 10.1177/1356336X11402268

Williams HG, 2008, OBESITY, V16, P1421, DOI 10.1038/oby.2008.214

Wong A.K.Y., 2006, ASIAN J PHYS ED RECR, V12, P23, DOI [10.24112/ajper.121132, DOI 10.24112/AJPER.121132]

NR 41

TC 21

Z9 21

U1 2

U2 31

PU SAGE PUBLICATIONS INC

PI THOUSAND OAKS

PA 2455 TELLER RD, THOUSAND OAKS, CA 91320 USA

SN 0031-5125

EI 1558-688X

J9 PERCEPT MOTOR SKILL

JI Percept. Mot. Skills

PD JUN

PY 2017

VL 124

IS 3

BP 584

EP 600

DI 10.1177/0031512517703005

PG 17

WC Psychology, Experimental

WE Social Science Citation Index (SSCI)

SC Psychology

GA EV4HZ

UT WOS:000401720900002

PM 28376671

DA 2022-08-17

ER

PT J

AU Augustijn, MJCM

D'Hondt, E

Van Acker, L

De Guchtenaere, A

Lenoir, M

Caeyenberghs, K

Deconinck, FJA

AF Augustijn, Mireille J. C. M.

D'Hondt, Eva

Van Acker, Lore

De Guchtenaere, Ann

Lenoir, Matthieu

Caeyenberghs, Karen

Deconinck, Frederik J. A.

TI Role of Motor Competence and Executive Functioning in Weight Loss: A

Study in Children with Obesity

SO JOURNAL OF DEVELOPMENTAL AND BEHAVIORAL PEDIATRICS

LA English

DT Article

DE obesity; children; motor competence; executive functioning;

multidisciplinary treatment program

ID BODY-MASS INDEX; OVERWEIGHT; ADOLESCENTS; COORDINATION; RELIABILITY;

SKILLS

AB Objective: This study aimed to compare motor competence and executive functioning (EF) between children with obesity and peers with healthy weight. Additionally, the predictive value of motor competence and EF in weight loss after a 5-month multidisciplinary residential treatment program was examined. Methods: Thirty-two children with obesity (7-11 years, 14 boys) and 32 age-matched controls (18 boys) performed 8 motor skill tasks and 4 tasks of EF (only at baseline). In the group of children with obesity, anthropometric measurements were performed at baseline and 5 months after the start of their treatment program. Also in control children, there was a time span of 5 months in between anthropometric measurements. Results: Lower levels of motor competence and reduced updating abilities, inhibition control, and planning skills were observed in children with obesity compared with healthy-weight controls. Within the total group, better general motor competence and balance skills were significantly associated with better updating, inhibition control, and planning. Finally, hierarchical regression analyses revealed that ball skills, balance skills, and inhibition/updating at baseline predicted 14% to 17% of the variance in weight loss after a 5-month treatment program in children with obesity. Conclusion: These results suggest that motor competence and EF are both relevant factors associated with childhood obesity. Moreover, these factors seem to be significant predictors of weight loss. Future (intervention) studies are needed to understand the impact of the difficulties in motor and EF on obesity-related behaviors as well as on short-term and, especially, long-term weight loss and maintenance.

C1 [Lenoir, Matthieu; Deconinck, Frederik J. A.] Univ Ghent, Dept Movement & Sports Sci, Ghent, Belgium.

[Augustijn, Mireille J. C. M.] Res Fdn Flanders FWO, Brussels, Belgium.

[D'Hondt, Eva] Vrije Univ Brussel, Dept Movement & Sports Sci, Fac Phys Educ & Physiotherapy, Brussels, Belgium.

[Van Acker, Lore; De Guchtenaere, Ann] Zeepreventorium VZW, De Haan, Belgium.

[Caeyenberghs, Karen] Australian Catholic Univ, Sch Psychol, Melbourne, Vic, Australia.

RP Augustijn, MJCM (通讯作者)，Univ Ghent, Fac Med & Hlth Sci, Dept Movement & Sports Sci, Watersportlaan 2, B-9000 Ghent, Belgium.

EM mireille.augustijn@ugent.be

RI Deconinck, Frederik J.A./H-1165-2016; De Guchtenaere, ann/AAJ-1666-2021;

D'Hondt, Eva/N-3253-2019

OI Deconinck, Frederik J.A./0000-0002-9064-9510; D'Hondt,

Eva/0000-0001-5646-2261; Caeyenberghs, Karen/0000-0001-7009-6843

FU Research Foundation Flanders (FWO) [3F000714]

FX The study was funded by the PhD fellowship of the Research Foundation

Flanders (FWO) awarded to M. J. C. M. Augustijn [3F000714].

CR Augustijn MJCM, 2017, BRAIN IMAGING BEHAV, P1

Barnett LM, 2009, J ADOLESCENT HEALTH, V44, P252, DOI 10.1016/j.jadohealth.2008.07.004

Blanco-Gomez A, 2015, ACTA PAEDIATR, V104, P619, DOI 10.1111/apa.12976

Cole TJ, 2012, PEDIATR OBES, V7, P284, DOI 10.1111/j.2047-6310.2012.00064.x

Cserjesi R, 2007, APPETITE, V49, P675, DOI 10.1016/j.appet.2007.04.001

D'Hondt E, 2014, OBESITY, V22, P1505, DOI 10.1002/oby.20723

D'Hondt E, 2009, ADAPT PHYS ACT Q, V26, P21, DOI 10.1123/apaq.26.1.21

Davis CL, 2007, RES Q EXERCISE SPORT, V78, P510, DOI 10.5641/193250307X13082512817660

Diamond A, 2000, CHILD DEV, V71, P44, DOI 10.1111/1467-8624.00117

Gentier I, 2013, RES DEV DISABIL, V34, P4043, DOI 10.1016/j.ridd.2013.08.040

Halberstadt J, 2016, APPETITE, V99, P82, DOI 10.1016/j.appet.2015.12.032

Hofmann W, 2009, J EXP SOC PSYCHOL, V45, P431, DOI 10.1016/j.jesp.2008.09.013

Kulendran M, 2014, INT J OBESITY, V38, P507, DOI 10.1038/ijo.2013.198

Liang J, 2013, INT J OBESITY, V38, P1

Lowe C, 1998, NEUROPSYCHOLOGIA, V36, P915, DOI 10.1016/S0028-3932(98)00036-0

Lyden J, 2018, PEDIATRICS, V141, DOI 10.1542/peds.141.1_MeetingAbstract.224

Nederkoorn C, 2007, BEHAV RES THER, V45, P1071, DOI 10.1016/j.brat.2006.05.009

Ng M, 2014, LANCET, V384, P766, DOI 10.1016/S0140-6736(14)60460-8

Pauli-Pott U, 2010, EUR CHILD ADOLES PSY, V19, P135, DOI 10.1007/s00787-009-0049-0

Ravanbakht SN, 2017, CURR PEDIAT REP, V5, P199

Rigoli D, 2012, DEV MED CHILD NEUROL, V54, P1025, DOI 10.1111/j.1469-8749.2012.04403.x

Robinson LE, 2015, SPORTS MED, V45, P1273, DOI 10.1007/s40279-015-0351-6

Smits-Engelsman B., 2010, MOVEMENT ASSESSMENT

Smits-Engelsman BCM, 2008, PHYS THER, V88, P286, DOI 10.2522/ptj.20070068

van der Fels IMJ, 2015, J SCI MED SPORT, V18, P697, DOI 10.1016/j.jsams.2014.09.007

van Egmond-Froehlich A, 2013, INT J EAT DISORDER, V46, P39, DOI 10.1002/eat.22043

Verbeken S, 2013, BEHAV RES THER, V51, P290, DOI 10.1016/j.brat.2013.02.006

Verdejo-Garcia A, 2010, OBESITY, V18, P1572, DOI 10.1038/oby.2009.475

Wu N, 2017, FRONT PSYCHOL, V8, DOI 10.3389/fpsyg.2017.00611

Yanovski JA, 2015, APPETITE, V93, P3, DOI 10.1016/j.appet.2015.03.028

NR 30

TC 12

Z9 13

U1 2

U2 11

PU LIPPINCOTT WILLIAMS & WILKINS

PI PHILADELPHIA

PA TWO COMMERCE SQ, 2001 MARKET ST, PHILADELPHIA, PA 19103 USA

SN 0196-206X

EI 1536-7312

J9 J DEV BEHAV PEDIATR

JI J. Dev. Behav. Pediatr.

PD OCT-NOV

PY 2018

VL 39

IS 8

BP 642

EP 651

DI 10.1097/DBP.0000000000000589

PG 10

WC Behavioral Sciences; Psychology, Developmental; Pediatrics

WE Science Citation Index Expanded (SCI-EXPANDED); Social Science Citation Index (SSCI)

SC Behavioral Sciences; Psychology; Pediatrics

GA HD5JA

UT WOS:000452563900005

PM 29877989

OA Green Published

DA 2022-08-17

ER

PT J

AU Adams, ILJ

Steenbergen, B

Lust, JM

Smits-Engelsman, BCM

AF Adams, Imke L. J.

Steenbergen, Bert

Lust, Jessica M.

Smits-Engelsman, Bouwien C. M.

TI Motor imagery training for children with developmental coordination

disorder - study protocol for a randomized controlled trial

SO BMC NEUROLOGY

LA English

DT Article

DE Developmental coordination disorder; DCD; Internal modeling deficit;

Motor imagery; Motor imagery training; Cognitive orientation to daily

occupational performance; CO-OP

ID PERFORMANCE; INTERVENTION; PREDICTION; CHILDHOOD; EFFICACY; BRAIN;

TASKS; MODEL; AGE

AB Background: Previous studies have shown that the predictive control of movements is impaired in children with Developmental Coordination Disorder (DCD), most likely due to a deficit in the internal modeling of movements. Motor imagery paradigms have been used to test this internal modeling deficit. The aim of the present study is to examine whether a training focused on the mental imagery of motor skills, can help to improve the motor abilities of children with DCD.

Methods/Design: A pre-post design will be used to examine the motor performance, motor imagery and motor planning abilities before and after a training of 9 weeks. Two groups will be included in this study (1) one receiving motor imagery (MI) training focused on the forward modeling of purposive actions, (2) one receiving Cognitive Orientation to daily Occupational Performance (CO-OP) training focused on identifying effective cognitive strategies that will increase motor competence. MI training will be given with the use of instruction videos of the motor skill that will be trained. Both groups will participate in 9 individual sessions of 45 min (once a week) with a paediatric physical or occupational therapist, added with homework sessions. Inclusion criteria are: (1) aged 7-12 years, (2) meeting the DSM-V criteria for DCD (motor performance substantially low (score on the m-ABC <= 16th percentile) and motor problems that interfere with daily life (DCDQ, and request for help at a paediatric physical or occupational therapist)). Exclusion criteria are IQ < 70 and other medical conditions causing the motor impairment.

Discussion: The results of this study will help to make treatment protocols for children with DCD more evidencebased. This study will increase our knowledge about the efficacy of both the MI training and CO-OP training, and both children with DCD and therapists will benefit from this knowledge.

C1 [Adams, Imke L. J.; Steenbergen, Bert; Lust, Jessica M.] Radboud Univ Nijmegen, Behav Sci Inst, NL-6500 HE Nijmegen, Netherlands.

[Steenbergen, Bert] Australian Catholic Univ, Sch Psychol, Melbourne, Vic 3065, Australia.

[Smits-Engelsman, Bouwien C. M.] Univ Cape Town, Dept Hlth & Rehabil Sci, Fac Hlth Sci, Grote Schuur Hosp, ZA-7925 Cape Town, South Africa.

RP Adams, ILJ (通讯作者)，Radboud Univ Nijmegen, Behav Sci Inst, POB 9104, NL-6500 HE Nijmegen, Netherlands.

EM i.adams@pwo.ru.nl

RI Adams, Imke/U-2504-2018

OI Adams, Imke/0000-0002-0950-9749; Steenbergen, Bert/0000-0001-8863-2624

FU Graduate School of the Behavioural Science Institute Nijmegen, Radboud

University Nijmegen

FX The MI/CO-OP trial is funded by the Graduate School of the Behavioural

Science Institute Nijmegen, Radboud University Nijmegen. We acknowledge

the contribution of physical therapists Rinske van Stee, Thomas Doll,

Max Keurentjes and Jesper Lange for arrangement of exercises and video

collection. We are grateful for the support of the clinicians working at

the participating centres.

CR Adams ILJ, 2014, NEUROSCI BIOBEHAV R, V47, P225, DOI 10.1016/j.neubiorev.2014.08.011

American Psychiatric Association, 2013, DIAGN STAT MAN MENT, V5th edition, DOI [10.1176/appi.books.9780890425596, DOI 10.1176/APPI.BOOKS.9780890425596]

Barnhart RC, 2003, PHYS THER, V83, P722, DOI 10.1093/ptj/83.8.722

Blank R, 2012, DEV MED CHILD NEUROL, V54, P54, DOI 10.1111/j.1469-8749.2011.04171.x

Bubic A, 2010, FRONT HUM NEUROSCI, V4, DOI 10.3389/fnhum.2010.00025

Cohen J., 1969, STAT POWER ANAL BEHA

Corcoran R., 2005, CAN ASS OCC THER C V

Coslett HB, 1998, BRAIN COGNITION, V37, P527, DOI 10.1006/brcg.1998.1011

Courtine G, 2004, COGNITIVE BRAIN RES, V22, P67, DOI 10.1016/j.cogbrainres.2004.07.008

Craje C, 2010, RES DEV DISABIL, V31, P1039, DOI 10.1016/j.ridd.2010.04.007

CUTHBERT BN, 1991, ADV PSYCHOPHYSIOLOGY, V4, P1

Decety J, 1996, BEHAV BRAIN RES, V72, P127

Decety J, 1996, COGNITIVE BRAIN RES, V3, P87, DOI 10.1016/0926-6410(95)00033-X

DECETY J, 1990, EUR ARCH PSY CLIN N, V240, P39, DOI 10.1007/BF02190091

Desmurget M, 2003, BRADFORD BOOKS, P289

Gaines R, 2008, J INTERPROF CARE, V22, P552, DOI 10.1080/13561820802039037

Hardy L, 1999, J SPORT EXERCISE PSY, V21, P95, DOI 10.1123/jsep.21.2.95

Hetu S, 2013, NEUROSCI BIOBEHAV R, V37, P930, DOI 10.1016/j.neubiorev.2013.03.017

Holmes PS, 2001, J APPL SPORT PSYCHOL, V13, P60, DOI 10.1080/10413200109339004

Hyde C, 2011, CHILD CARE HLTH DEV, V37, P111, DOI 10.1111/j.1365-2214.2010.01131.x

Hyde C, 2011, BRAIN COGNITION, V75, P232, DOI 10.1016/j.bandc.2010.12.004

Janssen J. J., 1994, Imagery in sports and physical performance, P1

JEANNEROD M, 1995, NEUROPSYCHOLOGIA, V33, P1419, DOI 10.1016/0028-3932(95)00073-C

Jelsma D, 2014, HUM MOVEMENT SCI, V33, P404, DOI 10.1016/j.humov.2013.12.007

Johnson-Frey SH, 2004, VIS COGN, V11, P371, DOI 10.1080/13506280344000329

Jongbloed-Pereboom M, 2013, J EXP CHILD PSYCHOL, V114, P295, DOI 10.1016/j.jecp.2012.08.008

Jongmans MJ, 2003, HUM MOVEMENT SCI, V22, P549, DOI 10.1016/j.humov.2003.09.009

Lang P.J., 1985, ANXIETY ANXIETY DISO

Malouin F, 2003, HUM BRAIN MAPP, V19, P47, DOI 10.1002/hbm.10103

Malouin F, 2013, FRONT HUM NEUROSCI, V7, DOI 10.3389/fnhum.2013.00576

Meichenbaum D., 1977, COGNITIVE BEHAV MODI

Miller LT, 2001, HUM MOVEMENT SCI, V20, P183, DOI 10.1016/S0167-9457(01)00034-3

Niemeijer AS, 2007, DEV MED CHILD NEUROL, V49, P406, DOI 10.1111/j.1469-8749.2007.00406.x

Polatajko H., 2004, ENABLING OCCUPATION

Polatajko Helene J., 2001, Physical and Occupational Therapy in Pediatrics, V20, P83, DOI 10.1300/J006v20n02_06

Polatajko Helene J., 2001, Physical and Occupational Therapy in Pediatrics, V20, P107, DOI 10.1300/J006v20n02_07

Sangster Claire A, 2005, Can J Occup Ther, V72, P67

Schoemaker M. M., 2003, Neural Plasticity, V10, P155, DOI 10.1155/NP.2003.155

Schoemaker M. M., 2008, COORDINATIEVRAGENLIJ

Schoemaker Marina M, 2015, Curr Dev Disord Rep, V2, P150

Schoemaker MM, 2005, CHILDREN DEV COORDIN, P212

Scholte E. M., 2004, ADHD VRAGENLIJST AVL

Schuster C, 2011, BMC MED, V9, DOI 10.1186/1741-7015-9-75

Shadmehr R, 2010, ANNU REV NEUROSCI, V33, P89, DOI 10.1146/annurev-neuro-060909-153135

Sharma N, 2006, STROKE, V37, P1941, DOI 10.1161/01.STR.0000226902.43357.fc

Smith D., 2001, Journal of Sport Behavior, V24, P408

Smith D, 2007, J APPL SPORT PSYCHOL, V19, P80, DOI 10.1080/10413200600944132

SMITH NC, 1992, J PSYCHOPHYSIOL, V6, P78

Smits-Engelsman B., 2005, SYSTEMATISCHE OPSPOR, P16

Smits-Engelsman BCM, 2013, HUM MOVEMENT SCI, V32, P1151, DOI 10.1016/j.humov.2012.06.006

Smits-Engelsman BCM, 2013, DEV MED CHILD NEUROL, V55, P229, DOI 10.1111/dmcn.12008

SMITSENGELSMAN BCM, 2010, HANDLEIDING MOVEMENT

ter Horst AC, 2010, EXP BRAIN RES, V203, P347, DOI 10.1007/s00221-010-2235-1

Thornton A, 2016, DISABIL REHABIL, V38, P979, DOI 10.3109/09638288.2015.1070298

Van Waelvelde H, 2012, SIGNAL, V21, P4

Wilson PH, 2013, DEV MED CHILD NEUROL, V55, P217, DOI 10.1111/j.1469-8749.2012.04436.x

Wilson PH, 2002, J CHILD NEUROL, V17, P491, DOI 10.1177/088307380201700704

Wilson PH, 2007, DEV COORDINATION DIS, P115

Wolpert DM, 2000, NAT NEUROSCI, V3, P1212, DOI 10.1038/81497

Wolpert DM, 1997, TRENDS COGN SCI, V1, P209, DOI 10.1016/S1364-6613(97)01070-X

Zwicker JG, 2015, PHYS OCCUP THER PEDI, V35, P163, DOI 10.3109/01942638.2014.957431

NR 61

TC 11

Z9 12

U1 2

U2 37

PU BMC

PI LONDON

PA CAMPUS, 4 CRINAN ST, LONDON N1 9XW, ENGLAND

EI 1471-2377

J9 BMC NEUROL

JI BMC Neurol.

PD JAN 12

PY 2016

VL 16

AR 5

DI 10.1186/s12883-016-0530-6

PG 9

WC Clinical Neurology

WE Science Citation Index Expanded (SCI-EXPANDED); Social Science Citation Index (SSCI)

SC Neurosciences & Neurology

GA DB3ES

UT WOS:000368393400003

PM 26758026

OA Green Published, gold

DA 2022-08-17

ER

PT J

AU Paquet, A

Olliac, B

Bouvard, MP

Golse, B

Vaivre-Douret, L

AF Paquet, Aude

Olliac, Bertrand

Bouvard, Manuel-Pierre

Golse, Bernard

Vaivre-Douret, Laurence

TI The Semiology of Motor Disorders in Autism Spectrum Disorders as

Highlighted from a Standardized Neuro-Psychomotor Assessment

SO FRONTIERS IN PSYCHOLOGY

LA English

DT Article

DE Autism Spectrum Disorders (ASD); children; motor disorder;

neuro-psychomotor functions; neurodevelopmental assessment

ID DEVELOPMENTAL COORDINATION DISORDER; HIGH-FUNCTIONING AUTISM;

ASPERGERS-SYNDROME; YOUNG-CHILDREN; IMPAIRMENT; DYSPRAXIA;

ABNORMALITIES; PERFORMANCE; SEVERITY; SKILLS

AB Background: Altered motor performance has been described in Autism Spectrum Disorders (ASD) with disturbances in walking; posture, coordination, or arm movements, but some individuals with ASD show no impairment of motor skills. The neuro-developmental processes that underpin the performance of neuro-psychomotor functions have not been widely explored, nor is it clear whether there are neuro-psychomotor functions specifically affected in ASD. Our objective was to focus on the semiology of motor disorders among children with ASD using a neuro-developmental assessment tool.

Method: Thirty-four children with ASD, with or without intellectual deficit (ID) were recruited in a child psychiatry department and Autism Resource Centers. Initial standard evaluations for diagnosis (psychiatric; psychological; psychomotor) were supplemented by a standardized assessment battery for neuro-developmental psychomotor functions (NP-MOT).

Results: The results of some NP-MOT tests differed between children with ASD with ID and those without. However, on the NP-MOT battery, neither of the two groups did well in the hi-manual and finger praxia tests (36 and 52% respectively failed). Manual and digital gnosopraxia showed some deficit (63 and 62% respectively failed). Postural deficits were found in tests for both static equilibrium (64%) and dynamic (52%). There were also difficulties in coordination between the upper and lower limbs in 58% of children. We found 75% failure in motor skills on the M-ABC test. Concerning muscular tone, significant laxity was observed in distal parts of the body (feet and hands), but hypertonia was observed in the proximal muscles of the lower limbs (reduced heel-ear angle).

Discussion: The results of manual and digital gnosopraxia tests point to a planning deficit in children with autism. A gesture programming deficit is also highlighted by the poor results in manual praxis, and by failures in the M-ABC tests despite prior training of the child. However, concerning global motor function, a significant difference was observed between children with and without ID. Our findings suggest a semiology of tone deregulation between proximal versus distal muscles, indeterminate tonic laterality, postural control deficit (proprioceptive), impairment of inter-hemispheric coordination (corpus callosum), and neurological soft signs such asdysdiadochokinesia, which leads us to hypothesize a general impairment of motor functions.

C1 [Paquet, Aude; Golse, Bernard; Vaivre-Douret, Laurence] Paris Descartes Univ, Sorbonne Paris Cite, Fac Med, Paris, France.

[Paquet, Aude; Golse, Bernard; Vaivre-Douret, Laurence] Necker Enfants Malad Univ Hosp, AP HP, Dept Child Psychiat, Paris, France.

[Paquet, Aude; Vaivre-Douret, Laurence] Univ Paris Saclay, INSERM, UMR1018, Villejuif, France.

[Paquet, Aude; Vaivre-Douret, Laurence] Univ Paris Saclay, CESP, Villejuif, France.

[Paquet, Aude; Vaivre-Douret, Laurence] Univ Paris 11, UVSQ, Villejuif, France.

[Paquet, Aude; Olliac, Bertrand] Esquirol Hosp, Dept Child & Adolescent Psychiat, Limoges, France.

[Olliac, Bertrand] Univ Limoges, Trop Neuroepidemiol, INSERM UMR 1094, Limoges, France.

[Bouvard, Manuel-Pierre] Univ Bordeaux, Perrens Hosp, Dept Child & Adolescent Psychiat, Bordeaux, France.

[Bouvard, Manuel-Pierre] Univ Bordeaux, Inst Neurosci Cognit & Integrat Aquitaine, CNRS, UMR 5287, Bordeaux, France.

[Vaivre-Douret, Laurence] Hop Cochin, AP HP, Dept Pediat, Child Dev, Paris, France.

[Vaivre-Douret, Laurence] Necker Enfants Malad Univ Hosp, Endocrinol Lab, Imagine Inst, Paris, France.

RP Paquet, A (通讯作者)，Paris Descartes Univ, Sorbonne Paris Cite, Fac Med, Paris, France.; Paquet, A (通讯作者)，Necker Enfants Malad Univ Hosp, AP HP, Dept Child Psychiat, Paris, France.; Paquet, A (通讯作者)，Univ Paris Saclay, INSERM, UMR1018, Villejuif, France.; Paquet, A (通讯作者)，Univ Paris Saclay, CESP, Villejuif, France.; Paquet, A (通讯作者)，Univ Paris 11, UVSQ, Villejuif, France.; Paquet, A (通讯作者)，Esquirol Hosp, Dept Child & Adolescent Psychiat, Limoges, France.

EM audepaquet@gmail.com

OI Paquet, Aude/0000-0002-4464-8592; Olliac, Bertrand/0000-0002-7442-2586

FU Caisse primaire d'assurance maladie de la Haute-Vienne; Fondation pour

la recherche en psychomotricite et maladies de civilization

FX This study was supported through funding provided by the Caisse primaire

d'assurance maladie de la Haute-Vienne; the Fondation pour la recherche

en psychomotricite et maladies de civilization.

CR ADRIEN JL, 1993, J AM ACAD CHILD PSY, V32, P617, DOI 10.1097/00004583-199305000-00019

Allen G, 2004, BIOL PSYCHIAT, V56, P269, DOI 10.1016/j.biopsych.2004.06.005

American of Psychiatric Association (APA), 2013, DSM 5 DIAGN STAT MAN

Bruininks H., 1978, BRUININKS OSERETSKY

Dowell LR, 2009, NEUROPSYCHOLOGY, V23, P563, DOI 10.1037/a0015640

Dziuk MA, 2007, DEV MED CHILD NEUROL, V49, P734, DOI 10.1111/j.1469-8749.2007.00734.x

Fournier KA, 2010, J AUTISM DEV DISORD, V40, P1227, DOI 10.1007/s10803-010-0981-3

Freitag CM, 2007, J AUTISM DEV DISORD, V37, P948, DOI 10.1007/s10803-006-0235-6

Gowen E, 2013, J AUTISM DEV DISORD, V43, P323, DOI 10.1007/s10803-012-1574-0

Green D, 2002, J CHILD PSYCHOL PSYC, V43, P655, DOI 10.1111/1469-7610.00054

Green D, 2009, DEV MED CHILD NEUROL, V51, P311, DOI 10.1111/j.1469-8749.2008.03242.x

Henderson S., 2007, MOVEMENT ASSESSMENT

HENDERSON SE, 1992, MOVEMENT ASSESSMENT

Hilton C, 2007, RES AUTISM SPECT DIS, V1, P339, DOI 10.1016/j.rasd.2006.12.003

Jansiewicz EM, 2006, J AUTISM DEV DISORD, V36, P613, DOI 10.1007/s10803-006-0109-y

Kashiwagi M, 2009, NEUROREPORT, V20, P1319, DOI 10.1097/WNR.0b013e32832f4d87

Kaufmann A. S., 2008, K ABC 2 BATTERIE EXA

Kroliczak G, 2009, CEREB CORTEX, V19, P2396, DOI 10.1093/cercor/bhn261

Lalanne C, 2012, BMC MED RES METHODOL, V12, DOI 10.1186/1471-2288-12-107

Liu T, 2013, RES AUTISM SPECT DIS, V7, P1244, DOI 10.1016/j.rasd.2013.07.002

Lord C., 1994, ADI AUTISM DIAGNOSIS

Maestro S, 2005, PSYCHOPATHOLOGY, V38, P26, DOI 10.1159/000083967

Mandelbaum DE, 2006, DEV MED CHILD NEUROL, V48, P33, DOI 10.1017/S0012162206000089

Miller M, 2014, BEHAV BRAIN RES, V269, P95, DOI 10.1016/j.bbr.2014.04.011

Ming X, 2007, BRAIN DEV-JPN, V29, P565, DOI 10.1016/j.braindev.2007.03.002

Pan CY, 2009, J AUTISM DEV DISORD, V39, P1694, DOI 10.1007/s10803-009-0813-5

Paquet A, 2016, CHILD NEUROPSYCHOL, V22, P763, DOI 10.1080/09297049.2015.1085501

Provost B, 2007, J AUTISM DEV DISORD, V37, P321, DOI 10.1007/s10803-006-0170-6

Sempe M, 1979, AUXOLOGIE METHODE SE

Shetreat-Klein M, 2014, BRAIN DEV-JPN, V36, P91, DOI 10.1016/j.braindev.2012.02.005

Staples KL, 2010, J AUTISM DEV DISORD, V40, P209, DOI 10.1007/s10803-009-0854-9

Vaivre-Douret L., 2006, BATTERIE EVALUATION

Vaivre-Douret L, 2011, DEV NEUROPSYCHOL, V36, P614, DOI 10.1080/87565641.2011.560696

VAIVREDOURET L, 1997, EVALUATION MOTRICITE

Van Waelvelde H, 2010, DEV MED CHILD NEUROL, V52, pe174, DOI 10.1111/j.1469-8749.2009.03606.x

Wechsler D, 2006, ECHELLE INTELLIGENCE

Wegiel J, 2014, ACTA NEUROPATHOL COM, V2, DOI 10.1186/s40478-014-0141-7

Whyatt CP, 2012, J AUTISM DEV DISORD, V42, P1799, DOI 10.1007/s10803-011-1421-8

Zwicker JG, 2012, PEDIATR NEUROL, V46, P162, DOI 10.1016/j.pediatrneurol.2011.12.007

NR 39

TC 30

Z9 31

U1 0

U2 14

PU FRONTIERS MEDIA SA

PI LAUSANNE

PA PO BOX 110, EPFL INNOVATION PARK, BUILDING I, LAUSANNE, 1015,

SWITZERLAND

SN 1664-1078

J9 FRONT PSYCHOL

JI Front. Psychol.

PD SEP 12

PY 2016

VL 7

AR 1292

DI 10.3389/fpsyg.2016.01292

PG 11

WC Psychology, Multidisciplinary

WE Social Science Citation Index (SSCI)

SC Psychology

GA DV3HD

UT WOS:000382810700001

PM 27672371

OA gold, Green Published

DA 2022-08-17

ER

PT J

AU Wang, HY

Huang, TH

Lo, SK

AF Wang, Hui-Yi

Huang, Tzu-Hsiu

Lo, Sing-Kai

TI Motor ability and adaptive function in children with attention deficit

hyperactivity disorder

SO KAOHSIUNG JOURNAL OF MEDICAL SCIENCES

LA English

DT Article

DE Adaptive function; Attention deficit hyperactivity disorder; Children

with developmental disability; Motor ability

ID DEFICIT/HYPERACTIVITY DISORDER; BEHAVIOR PROBLEMS; ADHD; COORDINATION;

BOYS; LIFE; TASK

AB Attention deficit hyperactivity disorder (ADHD) is a common neuropsychiatric disorder. Previous studies have reported that children with ADHD exhibit deficits of adaptive function and insufficient motor ability. The objective of this study was to investigate the association between adaptive function and motor ability in children with ADHD compared with a group of normal children. The study group included 25 children with ADHD (19 boys and 6 girls), aged from 4.6 years to 8.6 years (mean +/- standard deviation, 6.5 +/- 1.2). A group of 24 children without ADHD (normal children) were selected to match the children with ADHD on age and gender. The Movement Assessment Battery for Children, which includes three subtests, was used to assess the motor ability of the children of both groups. The Chinese version of Adaptive Behavior Scales, which consists of 12 life domains, was used to assess adaptive function of the children with ADHD. Compared with the normal children, children with ADHD exhibited poorer motor ability on all the three subtests of motor assessment. In the ADHD group, nine (36%) children had significant motor impairments and seven (28%) were borderline cases. A total of 10 (40%) children with ADHD had definite adaptive problems in one or more adaptive domains. With statistically controlling of IQ for the ADHD group, those children with impaired motor ability had significantly poorer behaviors in the adaptive domain of home living (p = 0.035). Moreover, children with ADHD who had severely impaired manual dexterity performed worse than the control group in the adaptive domains of home living (r = -0.47, p = 0.018), socialization (r= -0.49, p = 0.013), and self-direction (r = -0.41, p = 0.040). In addition, children with poorer ball skills had worse home living behavior (r = -0.56, p = 0.003). Children who had more impaired balance exhibited poorer performance in social behavior (r = -0.41, p = 0.040). This study found significant correlation between motor ability and adaptive function in children with ADHD, especially in their adaptive domains of home living, socialization, and self-direction. In clinical settings, identification of motor difficulties may have important implications for the understanding of relative factors in effective management of the adaptive dysfunction in children with ADHD. Copyright (C) 2011, Elsevier Taiwan LLC. All rights reserved.

C1 [Wang, Hui-Yi] Kaohsiung Med Univ, Dept Phys Therapy, Coll Hlth Sci, Kaohsiung 807, Taiwan.

[Huang, Tzu-Hsiu] Kaohsiung Med Univ, Chung Ho Mem Hosp, Dept Rehabil, Kaohsiung 807, Taiwan.

[Lo, Sing-Kai] Hong Kong Inst Educ, Fac Arts & Sci, Tai Po, Hong Kong, Peoples R China.

RP Wang, HY (通讯作者)，Kaohsiung Med Univ, Dept Phys Therapy, Coll Hlth Sci, 100 Shih Chuan 1st Rd, Kaohsiung 807, Taiwan.

EM hywang@kmu.edu.tw

OI LO, Sing Kai/0000-0001-8401-1759

FU Kaohsiung Medical University Chung-Ho Memorial Hospital, Taiwan [KMUH

4N-15]

FX This study was supported by a grant from the Kaohsiung Medical

University Chung-Ho Memorial Hospital, Taiwan (KMUH 4N-15).

CR American Psychological Association, 2000, DIAGN STAT MAN MENT, DOI DOI 10.1176/APPI.BOOKS.9780890423349

Barkley RA, 2006, J AM ACAD CHILD PSY, V45, P192, DOI 10.1097/01.chi.0000189134.97436.e2

Cheng Jia, 2007, Beijing Da Xue Xue Bao Yi Xue Ban, V39, P531

Clark C, 2002, J CHILD PSYCHOL PSYC, V43, P785, DOI 10.1111/1469-7610.00084

Crocker N, 2009, ALCOHOL CLIN EXP RES, V33, P2015, DOI 10.1111/j.1530-0277.2009.01040.x

Dunn L, 2009, PHYS OCCUP THER PEDI, V29, P258, DOI 10.1080/01942630903008350

Haywood K. M., 2001, LIFE SPAN MOTOR DEV

HENDERSON SE, 1992, MOVEMENT ASSESSMENT

HSU HL, 2004, CHINESE ADAPTIVE BEH

Jarratt KP, 2005, APPL NEUROPSYCHOL, V12, P83, DOI 10.1207/s15324826an1202_4

Jucaite A, 2003, DEV MED CHILD NEUROL, V45, P731, DOI 10.1017/S0012162203001373

Kaneko Fumiko, 2005, Phys Occup Ther Pediatr, V25, P45

Klassen AF, 2004, PEDIATRICS, V114, pE541, DOI 10.1542/peds.2004-0844

Leitner Y, 2007, J NEUROL, V254, P1330, DOI 10.1007/s00415-006-0522-3

Matejcek Z, 2003, NEUROENDOCRINOL LETT, V24, P148

Miyahara M, 2006, HUM MOVEMENT SCI, V25, P100, DOI 10.1016/j.humov.2005.11.004

Nijmeijer JS, 2008, CLIN PSYCHOL REV, V28, P692, DOI 10.1016/j.cpr.2007.10.003

Pan CY, 2009, J AUTISM DEV DISORD, V39, P1694, DOI 10.1007/s10803-009-0813-5

Piek JP, 1999, DEV MED CHILD NEUROL, V41, P159, DOI 10.1017/S0012162299000341

Pitcher TM, 2003, DEV MED CHILD NEUROL, V45, P525, DOI 10.1111/j.1469-8749.2003.tb00952.x

Pitcher TM, 2002, HUM MOVEMENT SCI, V21, P919, DOI 10.1016/S0167-9457(02)00167-7

Polanczyk G, 2007, CURR OPIN PSYCHIATR, V20, P386, DOI 10.1097/YCO.0b013e3281568d7a

RIEF SF, 2005, REACH TEACH CHILDREN, P4

STEIN MA, 1995, J CHILD PSYCHOL PSYC, V36, P663, DOI 10.1111/j.1469-7610.1995.tb02320.x

Stray LL, 2009, BEHAV BRAIN FUNCT, V5, DOI 10.1186/1744-9081-5-22

Sukhodolsky DG, 2005, AM J PSYCHIAT, V162, P1125, DOI 10.1176/appi.ajp.162.6.1125

Tzang RF, 2009, KAOHSIUNG J MED SCI, V25, P530, DOI 10.1016/S1607-551X(09)70545-5

Wechsler D., 2000, MANUAL WECHSLER PRES

Wechsler D., 2007, MANUAL WECHSLER INTE

Wolf LE, 2001, ANN NY ACAD SCI, V931, P396

NR 30

TC 19

Z9 23

U1 0

U2 18

PU ELSEVIER TAIWAN

PI TAIPEI

PA RM N-412, 4F, CHIA HSIN BUILDING 11, NO 96, ZHONG SHAN N ROAD SEC 2,

TAIPEI, 10449, TAIWAN

SN 1607-551X

J9 KAOHSIUNG J MED SCI

JI Kaohsiung J. Med. Sci.

PD OCT

PY 2011

VL 27

IS 10

BP 446

EP 452

DI 10.1016/j.kjms.2011.06.004

PG 7

WC Medicine, Research & Experimental

WE Science Citation Index Expanded (SCI-EXPANDED)

SC Research & Experimental Medicine

GA 838AP

UT WOS:000296258400004

PM 21943817

OA gold

DA 2022-08-17

ER

PT J

AU van Swieten, LM

van Bergen, E

Williams, JHG

Wilson, AD

Plumb, MS

Kent, SW

Mon-Williams, MA

AF van Swieten, Lisa M.

van Bergen, Elsje

Williams, Justin H. G.

Wilson, Andrew D.

Plumb, Mandy S.

Kent, Samuel W.

Mon-Williams, Mark A.

TI A Test of Motor (Not Executive) Planning in Developmental Coordination

Disorder and Autism

SO JOURNAL OF EXPERIMENTAL PSYCHOLOGY-HUMAN PERCEPTION AND PERFORMANCE

LA English

DT Article

DE motor planning; grip selection; bias; prehension; coordination; autism;

DCD

ID CHILDREN; PREHENSION; DYSFUNCTION; MOVEMENTS

AB Grip selection tasks have been used to test "planning" in both autism and developmental coordination disorder (DCD). We differentiate between motor and executive planning and present a modified motor planning task. Participants grasped a cylinder in 1 of 2 orientations before turning it clockwise or anticlockwise. The rotation resulted in a comfortable final posture at the cost of a harder initial reaching action on 50% of trials. We hypothesized that grip selection would be dominated by motoric developmental status. Adults were always biased towards a comfortable end-state with their dominant hand, but occasionally ended uncomfortably with their nondominant hand. Most 9- to 14-year-olds with and without autism also showed this "end-state comfort" bias but only 50% of 5- to 8-year-olds. In contrast, children with DCD were biased towards selecting the simplest initial movement. Our results are best understood in terms of motor planning, with selection of an easier initial grip resulting from poor reach-to-grasp control rather than an executive planning deficit. The absence of differences between autism and controls may reflect the low demand this particular task places on executive planning.

C1 [Mon-Williams, Mark A.] Univ Leeds, Inst Psychol Sci, Leeds LS2 9JT, W Yorkshire, England.

[van Swieten, Lisa M.] Vrije Univ Amsterdam, Res Inst MOVE, Fac Human Movement Sci, Amsterdam, Netherlands.

[van Bergen, Elsje] Univ Amsterdam, Dept Educ Sci, NL-1012 WX Amsterdam, Netherlands.

[Williams, Justin H. G.; Kent, Samuel W.] Univ Aberdeen, Coll Life Sci & Med, Aberdeen AB9 1FX, Scotland.

[Plumb, Mandy S.] Robert Gordon Univ, Sch Hlth Sci, Aberdeen AB9 1FR, Scotland.

RP Mon-Williams, MA (通讯作者)，Univ Leeds, Inst Psychol Sci, Leeds LS2 9JT, W Yorkshire, England.

EM pscmmw@leeds.ac.uk

RI Williams, Justin/AAE-5903-2020; Wilson, Andrew/A-7891-2008

OI Wilson, Andrew/0000-0002-0459-0728; van Bergen,

Elsje/0000-0002-5860-5745; Mon-Williams, Mark/0000-0001-7595-8545;

Plumb, Mandy/0000-0003-4838-8799

CR American Psychiatric Association, 1994, DIAGNOSTIC STAT MANU

Cohen RG, 2004, EXP BRAIN RES, V157, P486, DOI 10.1007/s00221-004-1862-9

de Lussanet MHE, 2002, BEHAV BRAIN RES, V129, P51, DOI 10.1016/S0166-4328(01)00320-5

de Lussanet MHE, 2001, EXP BRAIN RES, V137, P246, DOI 10.1007/s002210000607

HENDERSON SE, 1992, MOVEMENT ASSESSMENT

Hill EL, 2004, TRENDS COGN SCI, V8, P26, DOI 10.1016/j.tics.2003.11.003

HUGHES C, 1994, NEUROPSYCHOLOGIA, V32, P477, DOI 10.1016/0028-3932(94)90092-2

Hughes C, 1996, J AUTISM DEV DISORD, V26, P99, DOI 10.1007/BF02276237

Korkman M., 1998, NEPSY DEV NEUROPSYCH

Kuhtz-Buschbeck JP, 1998, EXP BRAIN RES, V122, P424, DOI 10.1007/s002210050530

Lord C, 2000, J AUTISM DEV DISORD, V30, P205, DOI 10.1023/A:1005592401947

LORD C, 1994, J AUTISM DEV DISORD, V24, P659, DOI 10.1007/BF02172145

Mon-Williams M, 2005, Q J EXP PSYCHOL-A, V58, P1249, DOI 10.1080/02724980443000575

ROSENBAUM D, 1990, ATTENTION PERFORM, V13, P321

ROSENBAUM DA, 1993, ATTENTION PERFORM, V14, P803

Rosenbaum DA, 2001, PSYCHOL REV, V108, P709, DOI 10.1037//0033-295X.108.4.709

Rosenbaum DA, 1996, ACTA PSYCHOL, V94, P59, DOI 10.1016/0001-6918(95)00062-3

Scheres A, 2004, ARCH CLIN NEUROPSYCH, V19, P569, DOI 10.1016/j.acn.2003.08.005

SHALLICE T, 1982, PHILOS T ROY SOC B, V298, P199, DOI 10.1098/rstb.1982.0082

Smyth MM, 1997, J CHILD PSYCHOL PSYC, V38, P1023, DOI 10.1111/j.1469-7610.1997.tb01619.x

Smyth MM, 2001, J MOTOR BEHAV, V33, P306, DOI 10.1080/00222890109601916

Tresilian JR, 2005, J MOTOR BEHAV, V37, P103

van Bergen E, 2007, EXP BRAIN RES, V178, P180, DOI 10.1007/s00221-006-0722-1

Weiss DJ, 2007, PSYCHOL SCI, V18, P1063, DOI 10.1111/j.1467-9280.2007.02026.x

NR 24

TC 84

Z9 85

U1 0

U2 35

PU AMER PSYCHOLOGICAL ASSOC

PI WASHINGTON

PA 750 FIRST ST NE, WASHINGTON, DC 20002-4242 USA

SN 0096-1523

EI 1939-1277

J9 J EXP PSYCHOL HUMAN

JI J. Exp. Psychol.-Hum. Percept. Perform.

PD APR

PY 2010

VL 36

IS 2

BP 493

EP 499

DI 10.1037/a0017177

PG 7

WC Psychology; Psychology, Experimental

WE Science Citation Index Expanded (SCI-EXPANDED); Social Science Citation Index (SSCI)

SC Psychology

GA 576CX

UT WOS:000276122700016

PM 20364932

OA Green Accepted

DA 2022-08-17

ER

PT J

AU Brumbacha, ACD

Goffmana, L

AF Brumbacha, Andrea C. DiDonato

Goffmana, Lisa

TI Interaction of Language Processing and Motor Skill in Children With

Specific Language Impairment

SO JOURNAL OF SPEECH LANGUAGE AND HEARING RESEARCH

LA English

DT Article

DE children; language; language disorders; specific language impairment;

speech motor control; speech production; syntax

ID DEVELOPMENTAL COORDINATION DISORDER; GRAMMATICAL MORPHOLOGY; SPEECH;

MOVEMENTS; DEFICITS; ACQUISITION; HYPOTHESIS; STABILITY; ABILITIES;

SEQUENCES

AB Purpose: To examine how language production interacts with speech motor and gross and fine motor skill in children with specific language impairment (SLI).

Method: Eleven children with SLI and 12 age-matched peers (4-6 years) produced structurally primed sentences containing particles and prepositions. Utterances were analyzed for errors and for articulatory duration and variability. Standard measures of motor, language, and articulation skill were also obtained.

Results: Sentences containing particles, as compared with prepositions, were less likely to be produced in a priming task and were longer in duration, suggesting increased difficulty with this syntactic structure. Children with SLI demonstrated higher articulatory variability and poorer gross and fine motor skills compared with aged-matched controls. Articulatory variability was correlated with generalized gross and fine motor performance.

Conclusions: Children with SLI show co-occurring speech motor and generalized motor deficits. Current theories do not fully account for the present findings, though the procedural deficit hypothesis provides a framework for interpreting overlap among language and motor domains.

C1 [Brumbacha, Andrea C. DiDonato; Goffmana, Lisa] Purdue Univ, W Lafayette, IN 47907 USA.

RP Goffmana, L (通讯作者)，Purdue Univ, W Lafayette, IN 47907 USA.

EM goffman@purdue.edu

OI Goffman, Lisa/0000-0002-7989-737X

FU National Institute on Deafness and Other Communication Disorders [R01

DC04826]; Purdue University; NATIONAL INSTITUTE ON DEAFNESS AND OTHER

COMMUNICATION DISORDERS [R01DC004826] Funding Source: NIH RePORTER

FX This research was funded by the National Institute on Deafness and Other

Communication Disorders Grant R01 DC04826. Andrea C. DiDonato Brumbach

was also supported by the Frederick N. Andrews Fellowship from Purdue

University. Portions of the research were presented at the Symposium for

Research in Child Language Disorders, Madison, WI. We thank Laurence

Leonard and Elaine Francis for their guidance and expertise and Janna

Berlin, Pat Deevy, Brooke Adams, Kelsey Pithoud, Rachel Brunner,

Michelle Wiersma, and Ilana Feld for their invaluable contributions. We

are also grateful to the children and families who participated in this

study.

CR Arbib MA, 2006, ACTION TO LANGUAGE VIA THE MIRROR NEURON SYSTEM, P1, DOI 10.1017/CBO9780511541599

Bankson N. W., 1990, BANKSON BERNTHAL TES

Bedore LM, 1998, J SPEECH LANG HEAR R, V41, P1185, DOI 10.1044/jslhr.4105.1185

BISHOP DVM, 1987, DEV MED CHILD NEUROL, V29, P442

Bruininks R.H., 2005, BRUININKS OSERETSKY

Burgemeister B., 1972, COLUMBIA MENTAL MATU

Caplan D, 2000, HUM BRAIN MAPP, V9, P65

Cappelle B., 2004, BELGIAN J LINGUISTIC, V18, P29

Carroll J. B., 1971, AM HERITAGE WORD FRE

DAWSON JI, 2003, STRUCTURED PHOTOGRAP

Dollaghan C, 1998, J SPEECH LANG HEAR R, V41, P1136, DOI 10.1044/jslhr.4105.1136

Folio M, 2000, PEABODY DEV MOTOR SC

Goffman L, 2004, J SPEECH LANG HEAR R, V47, P1088, DOI 10.1044/1092-4388(2004/081)

Goffman L, 1999, J SPEECH LANG HEAR R, V42, P1499, DOI 10.1044/jslhr.4206.1499

Goffman L, 2007, J SPEECH LANG HEAR R, V50, P444, DOI 10.1044/1092-4388(2007/031)

GREENFIELD PM, 1991, BEHAV BRAIN SCI, V14, P531, DOI 10.1017/S0140525X00071235

Hill E L, 1998, Laterality, V3, P295

Hill EL, 2001, INT J LANG COMM DIS, V36, P149, DOI 10.1080/13682820010019874

Hill EL, 1998, HUM MOVEMENT SCI, V17, P655, DOI 10.1016/S0167-9457(98)00017-7

Hill EL, 1998, DEV MED CHILD NEUROL, V40, P388

Huttenlocher J, 2004, J MEM LANG, V50, P182, DOI 10.1016/j.jml.2003.09.003

Iverson JM, 2010, J CHILD LANG, V37, P229, DOI 10.1017/S0305000909990432

Jancke L, 2007, BRAIN LANG, V102, P91, DOI 10.1016/j.bandl.2006.08.003

KAIL R, 1994, J SPEECH HEAR RES, V37, P418, DOI 10.1044/jshr.3702.418

Kent R. D., 2004, SPEECH MOTOR CONTROL, P3

Kleinow J, 2006, DEV PSYCHOBIOL, V48, P275, DOI 10.1002/dev.20141

Leonard L., 1998, CHILDREN SPECIFIC LA

Leonard LB, 2000, J MEM LANG, V43, P362, DOI 10.1006/jmla.1999.2689

Leonard LB, 1999, J SPEECH LANG HEAR R, V42, P678, DOI 10.1044/jslhr.4203.678

Locke JL, 1997, BRAIN LANG, V58, P265, DOI 10.1006/brln.1997.1791

Maner KJ, 2000, J SPEECH LANG HEAR R, V43, P560, DOI 10.1044/jslhr.4302.560

Marton K, 2009, J EXP CHILD PSYCHOL, V102, P1, DOI 10.1016/j.jecp.2008.07.007

Miller CA, 1998, J SPEECH LANG HEAR R, V41, P701, DOI 10.1044/jslhr.4103.701

Muursepp I, 2012, BRAIN DEV-JPN, V34, P128, DOI 10.1016/j.braindev.2011.02.002

Muursepp I, 2011, ACTA PAEDIATR, V100, P1038, DOI 10.1111/j.1651-2227.2011.02201.x

Nishitani N, 2000, P NATL ACAD SCI USA, V97, P913, DOI 10.1073/pnas.97.2.913

Noterdaeme M., 1998, J HUMAN MOVEMENT STU, V15, P151

Owen SE, 1997, CHILD CARE HLTH DEV, V23, P315, DOI 10.1046/j.1365-2214.1997.864864.x

POWELL RP, 1992, DEV MED CHILD NEUROL, V34, P755

Rice ML, 1998, J SPEECH LANG HEAR R, V41, P1412, DOI 10.1044/jslhr.4106.1412

Rizzolatti G, 2004, ANNU REV NEUROSCI, V27, P169, DOI 10.1146/annurev.neuro.27.070203.144230

SMITH A, 1995, EXP BRAIN RES, V104, P493

Smith A, 2000, J SPEECH LANG HEAR R, V43, P277, DOI 10.1044/jslhr.4301.277

SMITH A, 2004, SPEECH MOTOR CONTROL, P227

STARK RE, 1981, J SPEECH HEAR DISORD, V46, P114, DOI 10.1044/jshd.4602.114

STLOUIS K, 1987, ORAL SPEECH MECH SCR

Thelen E., 1994, DYNAMIC SYSTEMS APPR

Tomblin JB, 2007, LANG LEARN DEV, V3, P269, DOI 10.1080/15475440701377477

Ullman MT, 2005, CORTEX, V41, P399, DOI 10.1016/S0010-9452(08)70276-4

Walsh B, 2006, DEV PSYCHOBIOL, V48, P660, DOI 10.1002/dev.20185

WATKINS RV, 1991, J SPEECH HEAR RES, V34, P1130, DOI 10.1044/jshr.3405.1130

Zelaznik HN, 2010, J SPEECH LANG HEAR R, V53, P383, DOI 10.1044/1092-4388(2009/08-0204)

NR 52

TC 45

Z9 47

U1 1

U2 24

PU AMER SPEECH-LANGUAGE-HEARING ASSOC

PI ROCKVILLE

PA 10801 ROCKVILLE PIKE, ROCKVILLE, MD 20852-3279 USA

SN 1092-4388

EI 1558-9102

J9 J SPEECH LANG HEAR R

JI J. Speech Lang. Hear. Res.

PD FEB

PY 2014

VL 57

IS 1

BP 158

EP 171

DI 10.1044/1092-4388(2013/12-0215)

PG 14

WC Audiology & Speech-Language Pathology; Linguistics; Rehabilitation

WE Science Citation Index Expanded (SCI-EXPANDED); Social Science Citation Index (SSCI)

SC Audiology & Speech-Language Pathology; Linguistics; Rehabilitation

GA AZ0EM

UT WOS:000347919100014

PM 24023372

OA Green Accepted

DA 2022-08-17

ER

PT J

AU James, ME

Graham, JD

Chirico, D

King-Dowling, S

Cairney, J

AF James, Maeghan E.

Graham, Jeffrey D.

Chirico, Daniele

King-Dowling, Sara

Cairney, John

TI Investigating the mediating role of internalizing and externalizing

problems on physical fitness in children at risk for Developmental

Coordination Disorder

SO APPLIED PHYSIOLOGY NUTRITION AND METABOLISM

LA English

DT Article

DE self-regulation; executive functioning; motor coordination; physical fi

tness

ID CARDIORESPIRATORY FITNESS; PRESCHOOL-CHILDREN; MOTOR COORDINATION;

YOUNG-CHILDREN; ANXIETY; PERFORMANCE; CHILDHOOD; AGE

AB Children with Developmental Coordination Disorder (DCD) have poorer fitness and greater internalizing/externalizing problems compared with typically developing (TD) children. The purpose of this study was to examine the potential mediating role of internalizing/externalizing problems on the relationship between children at risk for DCD (DCDr) and physical fitness. Participants (N = 589) included 288 children with DCDr (Mage = 4.9, 67% male) and 301 TD children (Mage = 5.0, 48% male). Motor skills were assessed using the Movement Assessment Battery for Children-2nd edition (DCDr: at or below the 16th percentile, TD: 16th percentile). Parent-reported internalizing/externalizing problems were measured using the Child Behaviour Checklist. Physical fitness was measured using the Bruce protocol maximal treadmill test and a 30-second Wingate test. Tests for indirect (mediation) effects were assessed using the PROCESS v3.5 software macro. Children with DCDr had higher internalizing and externalizing problems (p < 0.001, d = 0.35???0.46) and poorer fitness levels (p < 0.001, hp2 = 0.05???0.09). Internalizing problems mediated fitness performance on both the treadmill and Wingate test; however, externalizing problems showed no mediating effects. Thus, interventions targeting internalizing problems may contribute to improving performance on fitness-based tasks among children with DCDr.

C1 [James, Maeghan E.] Univ Toronto, Fac Kinesiol & Phys Educ, Toronto, ON, Canada.

[Graham, Jeffrey D.; Chirico, Daniele; King-Dowling, Sara; Cairney, John] McMaster Univ, Dept Family Med, Infant & Child Hlth INCH Lab, Hamilton, ON, Canada.

[Graham, Jeffrey D.] Ontario Tech Univ, Fac Hlth Sci, Oshawa, ON, Canada.

[Chirico, Daniele] Univ Calgary, Fac Kinesiol, Calgary, AB, Canada.

[King-Dowling, Sara] Childrens Hosp Philadelphia, Div Oncol, Philadelphia, PA USA.

[Cairney, John] Univ Queensland, Sch Human Movement & Nutr Sci, St Lucia, Qld, Australia.

RP Cairney, J (通讯作者)，McMaster Univ, Dept Family Med, Infant & Child Hlth INCH Lab, Hamilton, ON, Canada.; Cairney, J (通讯作者)，Univ Queensland, Sch Human Movement & Nutr Sci, St Lucia, Qld, Australia.

EM j.cairney@uq.edu.au

OI King-Dowling, Sara/0000-0003-3670-8425

FU Canadian Institutes of Health Research [MOP 126015]; Canadian Institutes

of Health Research postdoctoral fellowship

FX The CATCH study is funded by the Canadian Institutes of Health Research

(MOP 126015) . Dr. King-Dowling was funded by a Canadian Institutes of

Health Research postdoctoral fellowship.

CR Achenbach T. M, 2013, DSM ORIENTED GUIDE A

Achenback T.M., 2000, MANUAL ASEBA PRESCHO, V30

Alesi M, 2019, EUR J SPEC NEEDS EDU, V34, P285, DOI 10.1080/08856257.2018.1468635

Anderssen SA, 2007, EUR J CARDIOV PREV R, V14, P526, DOI 10.1097/HJR.0b013e328011efc1

Armstrong N, 1994, Exerc Sport Sci Rev, V22, P435

BAROR O, 1987, SPORTS MED, V4, P381, DOI 10.2165/00007256-198704060-00001

Blank R, 2019, DEV MED CHILD NEUROL, V61, P242, DOI 10.1111/dmcn.14132

Bolin JH, 2014, J EDUC MEAS, V51, P335, DOI 10.1111/jedm.12050

BRUCE RA, 1973, AM HEART J, V85, P546, DOI 10.1016/0002-8703(73)90502-4

Cairney J, 2006, AM J HUM BIOL, V18, P66, DOI 10.1002/ajhb.20470

Cairney J, 2019, BMJ OPEN, V9, DOI 10.1136/bmjopen-2019-029784

Cairney J, 2015, BMC PUBLIC HEALTH, V15, DOI 10.1186/s12889-015-2582-8

Cairney J, 2017, J SCI MED SPORT, V20, P380, DOI 10.1016/j.jsams.2016.08.025

Cairney J, 2013, DEV REV, V33, P224, DOI 10.1016/j.dr.2013.07.002

Cairney J, 2011, BRIT J SPORT MED, V45, P1196, DOI 10.1136/bjsm.2009.069880

Carter T, 2021, J AFFECT DISORDERS, V285, P10, DOI 10.1016/j.jad.2021.02.026

Chia LC, 2013, RES DEV DISABIL, V34, P2098, DOI 10.1016/j.ridd.2013.03.023

COHEN J, 1992, PSYCHOL BULL, V112, P155, DOI 10.1037/0033-2909.112.1.155

EYSENCK MW, 1992, COGNITION EMOTION, V6, P409, DOI 10.1080/02699939208409696

Gibbs J, 2007, ARCH DIS CHILD, V92, P534, DOI 10.1136/adc.2005.088054

Gillberg C, 2004, EUR CHILD ADOLES PSY, V13, P80, DOI 10.1007/s00787-004-1008-4

Goulardins JB, 2015, BEHAV BRAIN RES, V292, P484, DOI 10.1016/j.bbr.2015.07.009

Harvey WJ, 2003, ADAPT PHYS ACT Q, V20, P1, DOI 10.1123/apaq.20.1.1

Hayes AF, 2013, PSYCHOL SCI, V24, P1918, DOI 10.1177/0956797613480187

Kaufman A. S., 2004, KBIT 2 KAUFMAN BRIEF

King-Dowling S, 2019, DEV MED CHILD NEUROL, V61, P1302, DOI 10.1111/dmcn.14237

King-Dowling S, 2018, MED SCI SPORT EXER, V50, P1442, DOI 10.1249/MSS.0000000000001590

Laukkanen JA, 2016, MAYO CLIN PROC, V91, P1183, DOI 10.1016/j.mayocp.2016.05.014

Li YC, 2018, PSYCHOL SPORT EXERC, V37, P244, DOI 10.1016/j.psychsport.2017.11.001

Lodewyk KR, 2016, PHYS EDUC SPORT PEDA, V21, P603, DOI 10.1080/17408989.2015.1095869

LONG BC, 1995, J APPL SPORT PSYCHOL, V7, P167, DOI 10.1080/10413209508406963

Malina RM, 2001, AM J HUM BIOL, V13, P162, DOI 10.1002/1520-6300(200102/03)13:2<162::AID-AJHB1025>3.0.CO;2-T

Mancini V, 2019, RES DEV DISABIL, V84, P96, DOI 10.1016/j.ridd.2018.07.003

Mancini VO, 2018, BRIT J EDUC PSYCHOL, V88, P363, DOI 10.1111/bjep.12187

Mancini VO, 2016, FRONT PSYCHOL, V7, DOI [10.3389/fpsyg.2016.00230, 10.3389/fpsyg.2016.00239]

Missiuna Cheryl, 2006, Paediatr Child Health, V11, P507

Missiuna C, 2014, RES DEV DISABIL, V35, P1198, DOI 10.1016/j.ridd.2014.01.007

Moshier SJ, 2016, BEHAV MODIF, V40, P178, DOI 10.1177/0145445515603704

Ortega FB, 2008, INT J OBESITY, V32, P1, DOI 10.1038/sj.ijo.0803774

Proudfoot NA, 2019, PEDIATRICS, V144, DOI 10.1542/peds.2018-2242

Raghuveer G, 2020, CIRCULATION, V142, pE101, DOI 10.1161/CIR.0000000000000866

Rivilis I, 2011, RES DEV DISABIL, V32, P894, DOI 10.1016/j.ridd.2011.01.017

Rodriguez MC, 2019, FRONT PEDIATR, V7, DOI 10.3389/fped.2019.00474

Semple RJ, 2005, J COGN PSYCHOTHER, V19, P379, DOI 10.1891/088983905780907702

Tackett J., 2012, HDB TEMPERAMENT, P562

Le TY, 2020, J SPORT EXERCISE PSY, V42, P407, DOI 10.1123/jsep.2019-0155

van der Cammen-van Zijp MHM, 2010, EUR J APPL PHYSIOL, V108, P393, DOI 10.1007/s00421-009-1236-x

Woodman T, 2003, J SPORT SCI, V21, P443, DOI 10.1080/0264041031000101809

Yap MBH, 2016, CLIN PSYCHOL REV, V50, P138, DOI 10.1016/j.cpr.2016.10.003

Zimmer C, 2020, ADAPT PHYS ACT Q, V37, P385, DOI 10.1123/apaq.2019-0193

NR 50

TC 0

Z9 0

U1 0

U2 0

PU CANADIAN SCIENCE PUBLISHING

PI OTTAWA

PA 65 AURIGA DR, SUITE 203, OTTAWA, ON K2E 7W6, CANADA

SN 1715-5312

EI 1715-5320

J9 APPL PHYSIOL NUTR ME

JI Appl. Physiol. Nutr. Metab.

PD MAY

PY 2022

VL 47

IS 5

BP 575

EP 581

DI 10.1139/apnm-2021-0369

EA FEB 2022

PG 7

WC Nutrition & Dietetics; Physiology; Sport Sciences

WE Science Citation Index Expanded (SCI-EXPANDED)

SC Nutrition & Dietetics; Physiology; Sport Sciences

GA 1L1MA

UT WOS:000793940300001

PM 35167349

DA 2022-08-17

ER

PT J

AU Rahimi-Golkhandan, S

Steenbergen, B

Piek, JP

Wilson, PH

AF Rahimi-Golkhandan, S.

Steenbergen, B.

Piek, J. P.

Wilson, P. H.

TI Deficits of hot executive function in developmental coordination

disorder: Sensitivity to positive social cues

SO HUMAN MOVEMENT SCIENCE

LA English

DT Article

DE Developmental coordination disorder (DCD); Hot executive function;

Cognitive control; Self-regulation; Go/no-go

ID SCHOOL-AGED CHILDREN; VENTROMEDIAL PREFRONTAL CORTEX; IOWA GAMBLING

TASK; DECISION-MAKING; MOTOR COORDINATION; EMOTION REGULATION; COGNITIVE

CONTROL; FUTURE CONSEQUENCES; FORCE CONTROL; ADOLESCENTS

AB Recent research shows that children with motor coordination problems (or developmental coordination disorder - DCD) show deficits in not only cool executive function (EF), but also hot EF. We aimed to determine whether this deficit of hot EF is due to heightened sensitivity to rewarding stimuli, specifically, or to a general deficit of cognitive control, like inhibition. Using two versions of a go/no-go task, one with neutral facial expressions and the other with happy and fearful faces, we compared 12 children with DCD with 28 typically-developing children, aged 7-12 years. Like earlier studies, children responded faster to happy faces. Both groups showed comparable accuracy in response to go targets, and also had similar commission errors, except when the no-go stimulus was a happy face. Importantly, the DCD group made significantly more commission errors to happy faces failing to suppress their response on more than half of the no-go trials. These results suggest a heightened sensitivity to emotionally significant distractors in DCD; this type of impulsivity may undermine self-regulation in DCD, with possible implications for adaptive function and emotional well-being. We argue that the interaction of cognitive control and emotion processing networks may be disrupted in DCD or delayed in development.(C) 2014 Elsevier B.V. All rights reserved.

C1 [Rahimi-Golkhandan, S.; Steenbergen, B.; Wilson, P. H.] Australian Catholic Univ, Sch Psychol, Melbourne, Vic 3450, Australia.

[Steenbergen, B.] Radboud Univ Nijmegen, Inst Behav Sci, NL-6525 ED Nijmegen, Netherlands.

[Piek, J. P.] Curtin Univ, Sch Psychol & Speech Pathol, Perth, WA 6845, Australia.

RP Wilson, PH (通讯作者)，Australian Catholic Univ, Sch Psychol, 115 Victoria Parade, Melbourne, Vic 3450, Australia.

EM peterh.wilson@acu.edu.au

RI Wilson, Peter H./E-2881-2018

OI Wilson, Peter H./0000-0003-3747-0287; Steenbergen,

Bert/0000-0001-8863-2624; Piek, Jan/0000-0003-3838-6773

CR American Psychiatric Association, 2013, DIAGN STAT MAN MENT, V5th edition, DOI [10.1176/appi.books.9780890425596, DOI 10.1176/APPI.BOOKS.9780890425596]

Bechara A, 2005, CURR OPIN NEUROL, V18, P734, DOI 10.1097/01.wco.0000194141.56429.3c

BECHARA A, 1994, COGNITION, V50, P7, DOI 10.1016/0010-0277(94)90018-3

Braver TS, 2001, CEREB CORTEX, V11, P825, DOI 10.1093/cercor/11.9.825

Cairney J, 2013, DEV MED CHILD NEUROL, V55, P55, DOI 10.1111/dmcn.12308

Cairney J, 2013, DEV REV, V33, P224, DOI 10.1016/j.dr.2013.07.002

Casey BJ, 2011, P NATL ACAD SCI USA, V108, P14998, DOI 10.1073/pnas.1108561108

Castellanos FX, 2006, TRENDS COGN SCI, V10, P117, DOI 10.1016/j.tics.2006.01.011

COLE PM, 1986, CHILD DEV, V57, P1309, DOI 10.2307/1130411

Crone EA, 2004, DEV NEUROPSYCHOL, V25, P251, DOI 10.1207/s15326942dn2503_2

Crone EA, 2003, PERS INDIV DIFFER, V35, P1625, DOI 10.1016/S0191-8869(02)00386-0

Crone EA, 2007, CHILD DEV, V78, P1288, DOI 10.1111/j.1467-8624.2007.01066.x

Cummins A, 2005, DEV MED CHILD NEUROL, V47, P437, DOI 10.1017/S001216220500085X

Damasio A. R., 2004, EMOTION EVOLUTION RA, P3

Damasio AR, 1998, BRAIN RES REV, V26, P83, DOI 10.1016/S0165-0173(97)00064-7

Damasio H, 1994, DESCARTES ERROR

Dennis TA, 2009, DEV NEUROPSYCHOL, V34, P85, DOI 10.1080/87565640802564887

Diamond A, 2013, ANNU REV PSYCHOL, V64, P135, DOI 10.1146/annurev-psych-113011-143750

Diamond A, 2011, SCIENCE, V333, P959, DOI 10.1126/science.1204529

Dinn WM, 2001, BRAIN COGNITION, V46, P114, DOI 10.1016/S0278-2626(01)80046-4

Duerden EG, 2013, BRAIN RES, V1533, P80, DOI 10.1016/j.brainres.2013.08.021

Dunn BD, 2006, NEUROSCI BIOBEHAV R, V30, P239, DOI 10.1016/j.neubiorev.2005.07.001

Durston S, 2002, NEUROIMAGE, V16, P449, DOI 10.1006/nimg.2002.1074

Eigsti IM, 2006, PSYCHOL SCI, V17, P478, DOI 10.1111/j.1467-9280.2006.01732.x

Ernst M, 2002, NEUROPSYCHOPHARMACOL, V26, P682, DOI 10.1016/S0893-133X(01)00414-6

Frank MJ, 2006, NEURAL NETWORKS, V19, P1120, DOI 10.1016/j.neunet.2006.03.006

Geuze RH, 2001, HUM MOVEMENT SCI, V20, P7, DOI 10.1016/S0167-9457(01)00027-6

Green D, 2008, HUM MOVEMENT SCI, V27, P363, DOI 10.1016/j.humov.2008.02.009

Groenewegen Henk J., 2003, Neural Plasticity, V10, P107, DOI 10.1155/NP.2003.107

Hare TA, 2005, BIOL PSYCHIAT, V57, P624, DOI 10.1016/j.biopsych.2004.12.038

HARE TA, 2005, COGNITION BRAIN BEHA, V9, P273

Hare TA, 2008, BIOL PSYCHIAT, V63, P927, DOI 10.1016/j.biopsych.2008.03.015

Hinson JM, 2002, COGN AFFECT BEHAV NE, V2, P341, DOI 10.3758/CABN.2.4.341

Hongwanishkul D, 2005, DEV NEUROPSYCHOL, V28, P617, DOI 10.1207/s15326942dn2802_4

Hooper CJ, 2004, DEV PSYCHOL, V40, P1148, DOI 10.1037/0012-1649.40.6.1148

Hoyle R. H., 2011, NAT RES COUNC WORKSH

Huizenga HM, 2007, DEVELOPMENTAL SCI, V10, P814, DOI 10.1111/j.1467-7687.2007.00621.x

Jokic CS, 2011, EDUC PSYCHOL REV, V23, P75, DOI 10.1007/s10648-010-9148-1

Kashiwagi M., 2013, FUNCTIONAL BRAIN MAP, P37

Lagattuta KH, 2011, DEVELOPMENTAL SCI, V14, P481, DOI 10.1111/j.1467-7687.2010.00994.x

Leonard HC, 2014, CHILD ADOL MENT H-UK, V19, P163, DOI 10.1111/camh.12055

LUNDYEKMAN L, 1991, J COGNITIVE NEUROSCI, V3, P367, DOI 10.1162/jocn.1991.3.4.367

McCarron L. T., 1997, MAND MCCARRON ASSESS

MISCHEL W, 1972, J PERS SOC PSYCHOL, V21, P204, DOI 10.1037/h0032198

Miyake A, 2000, COGNITIVE PSYCHOL, V41, P49, DOI 10.1006/cogp.1999.0734

Moffitt T. E, 2011, P NATL ACAD SCI

Murphy FC, 1999, PSYCHOL MED, V29, P1307, DOI 10.1017/S0033291799001233

Orzhekhovskaya N S, 1981, Neurosci Behav Physiol, V11, P379, DOI 10.1007/BF01184205

Piek JP, 2004, ARCH CLIN NEUROPSYCH, V19, P1063, DOI 10.1016/j.acn.2003.12.007

Piek JP, 1999, J INT NEUROPSYCH SOC, V5, P320, DOI 10.1017/S1355617799544032

Prencipe A, 2011, J EXP CHILD PSYCHOL, V108, P621, DOI 10.1016/j.jecp.2010.09.008

Querne L, 2008, BRAIN RES, V1244, P89, DOI 10.1016/j.brainres.2008.07.066

Rahimi-Golkhandan S, 2014, COGNITIVE DEV, V32, P23, DOI 10.1016/j.cogdev.2014.06.002

Schmahmann JD, 2006, BRAIN, V129, P290, DOI 10.1093/brain/awh729

Schneider W., 2002, E PRIME USERS GUIDE

Schulz KP, 2007, ARCH CLIN NEUROPSYCH, V22, P151, DOI 10.1016/j.acn.2006.12.001

SHODA Y, 1990, DEV PSYCHOL, V26, P978, DOI 10.1037/0012-1649.26.6.978

Skinner RA, 2001, HUM MOVEMENT SCI, V20, P73, DOI 10.1016/S0167-9457(01)00029-X

Smith DG, 2012, DEV PSYCHOL, V48, P1180, DOI 10.1037/a0026342

Somerville LH, 2011, J COGNITIVE NEUROSCI, V23, P2123, DOI 10.1162/jocn.2010.21572

Tan SK, 2001, ADAPT PHYS ACT Q, V18, P168, DOI 10.1123/apaq.18.2.168

Thompson R A, 1994, Monogr Soc Res Child Dev, V59, P25, DOI 10.2307/1166137

Toplak ME, 2005, BEHAV BRAIN FUNCT, V1, DOI 10.1186/1744-9081-1-8

Tottenham N, 2011, FRONT PSYCHOL, V2, DOI 10.3389/fpsyg.2011.00039

Urben S, 2012, DEV NEUROPSYCHOL, V37, P668, DOI 10.1080/87565641.2012.675378

Van Duijvenvoorde ACK, 2012, DEV PSYCHOL, V48, P192, DOI 10.1037/a0025601

van Duijvenvoorde ACK, 2010, DEV NEUROPSYCHOL, V35, P539, DOI 10.1080/87565641.2010.494749

Wilson P. H., 2013, DEV MED CHILD NEUROL

Wilson PH, 2003, HUM MOVEMENT SCI, V22, P515, DOI 10.1016/j.humov.2003.09.007

Wyatt T. M., 2013, SELF REGULATION PRES

Yerys BE, 2013, NEUROPSYCHOLOGY, V27, P537, DOI 10.1037/a0033615

Zelazo P. D., 2011, WILEY BLACKWELL HDB, P574, DOI DOI 10.1002/9781444325485.CH22

Zelazo PD, 2012, CHILD DEV PERSPECT, V6, P354, DOI 10.1111/j.1750-8606.2012.00246.x

Zwicker J. G., 2012, CHILD CARE HLTH DEV

Zwicker JG, 2012, EUR J PAEDIATR NEURO, V16, P573, DOI 10.1016/j.ejpn.2012.05.005

Zwicker JG, 2011, INT J DEV NEUROSCI, V29, P145, DOI 10.1016/j.ijdevneu.2010.12.002

NR 76

TC 10

Z9 11

U1 0

U2 36

PU ELSEVIER SCIENCE BV

PI AMSTERDAM

PA PO BOX 211, 1000 AE AMSTERDAM, NETHERLANDS

SN 0167-9457

EI 1872-7646

J9 HUM MOVEMENT SCI

JI Hum. Mov. Sci.

PD DEC

PY 2014

VL 38

BP 209

EP 224

DI 10.1016/j.humov.2014.09.008

PG 16

WC Neurosciences; Psychology; Psychology, Experimental; Sport Sciences

WE Science Citation Index Expanded (SCI-EXPANDED); Social Science Citation Index (SSCI)

SC Neurosciences & Neurology; Psychology; Sport Sciences

GA AY5GJ

UT WOS:000347600600019

PM 25457419

DA 2022-08-17

ER

PT J

AU Casnar, CL

Janke, KM

van der Fluit, F

Brei, NG

Klein-Tasman, BP

AF Casnar, Christy L.

Janke, Kelly M.

van der Fluit, Faye

Brei, Natalie G.

Klein-Tasman, Bonita P.

TI Relations between fine motor skill and parental report of attention in

young children with neurofibromatosis type 1

SO JOURNAL OF CLINICAL AND EXPERIMENTAL NEUROPSYCHOLOGY

LA English

DT Article

DE Attention; Fine motor skill; Neurofibromatosis type 1; Children;

Preschoolers

ID DEFICIT HYPERACTIVITY DISORDER; NEUROPSYCHOLOGICAL PROFILE;

LEARNING-DISABILITIES; ACADEMIC-PERFORMANCE; COGNITIVE DEFICITS; ADHD;

COORDINATION; ADOLESCENTS; BOYS; PROFICIENCY

AB Neurofibromatosis type 1 (NF1) is one of the most common genetic disorders presenting in approximately 1 in 3,500 live births. NF1 is a highly variable condition with a large number of complications. A common complication is neuropsychological problems, including developmental delays and learning difficulties that affect as many as 60% of patients. Research has suggested that school-aged children with NF1 often have poorer fine motor skills and are at greater risk for attention difficulties than the general population. Thirty-eight children with NF1 and 23 unaffected children between the ages of 4 and 6 years, who are enrolled in a study of early development in NF1, were included in the present study. Varying levels of fine motor functioning were examined (simple to complex fine motor tasks). For children with NF1, significant difficulties were demonstrated on lab-based mid-level and complex fine motor tasks, even after controlling for nonverbal reasoning abilities, but not on simple fine motor tasks. Parental report also indicated difficulties in everyday adaptive fine motor functioning. No significant correlations were found between complex fine motor ability and attention difficulties. This study provides much needed descriptive data on the early emergence of fine motor difficulties and attention difficulties in young children with NF1.

C1 [Casnar, Christy L.; van der Fluit, Faye; Brei, Natalie G.; Klein-Tasman, Bonita P.] Univ Wisconsin, Dept Psychol, Milwaukee, WI 53211 USA.

[Janke, Kelly M.] North Shore Long Isl Jewish Hlth Syst, Great Neck, NY USA.

RP Casnar, CL (通讯作者)，Univ Wisconsin, Dept Psychol, Milwaukee, WI 53211 USA.

EM clcasnar@uwm.edu

OI Klein-Tasman, Bonita/0000-0002-0932-1519

FU NF Midwest; NF MidAtlantic; University of Chicago CTSA [UL1 RR024999];

University of Wisconsin-Milwaukee Research Growth Initiative; NATIONAL

CENTER FOR RESEARCH RESOURCES [UL1RR024999] Funding Source: NIH RePORTER

FX This work was supported by the NF Midwest; NF MidAtlantic; University of

Chicago CTSA [grant number UL1 RR024999]; and the University of

Wisconsin-Milwaukee Research Growth Initiative.

CR Alloway TP, 2009, CHILD DEV, V80, P606, DOI 10.1111/j.1467-8624.2009.01282.x

Barkley RA, 1997, PSYCHOL BULL, V121, P65, DOI 10.1037/0033-2909.121.1.65

Billingsley RL, 2003, BRAIN LANG, V85, P125, DOI 10.1016/S0093-934X(02)00563-1

BRUININKS RH, 1996, SCALES INDEPENDENT B

Casey BJ, 2007, CURR OPIN NEUROL, V20, P119, DOI 10.1097/WCO.0b013e3280a02f78

Chapman CA, 1996, AM J MED GENET, V67, P127, DOI 10.1002/(SICI)1096-8628(19960409)67:2<127::AID-AJMG1>3.0.CO;2-P

Cohen J., 1988, STAT POWER ANAL SOCI

Conners CK., 1997, CONNERSRATING SCALES

Descheemaeker MJ, 2005, J INTELL DISABIL RES, V49, P33, DOI 10.1111/j.1365-2788.2005.00660.x

Diamond A, 2000, CHILD DEV, V71, P44, DOI 10.1111/1467-8624.00117

Dilts CV, 1996, J DEV BEHAV PEDIATR, V17, P229

Doyle S., 1995, OCCUPATIONAL THERAPY, V2, P229, DOI DOI 10.1002/0TI.6150020403

Egeland J, 2012, J LEARN DISABIL-US, V45, P361, DOI 10.1177/0022219411407922

ELLIOTT CD, 1990, DIFFERENTIAL ABILITY

Fliers E, 2008, J NEURAL TRANSM, V115, P211, DOI 10.1007/s00702-007-0827-0

Gilboa Y, 2010, RES DEV DISABIL, V31, P929, DOI 10.1016/j.ridd.2010.03.005

GRODZINSKY GM, 1992, DEV NEUROPSYCHOL, V8, P427, DOI 10.1080/87565649209540536

Hachon C, 2011, BRAIN DEV-JPN, V33, P52, DOI 10.1016/j.braindev.2009.12.008

HOFMAN KJ, 1994, J PEDIATR-US, V124, pS1, DOI 10.1016/S0022-3476(05)83163-4

Huijbregts S, 2010, DEV NEUROPSYCHOL, V35, P737, DOI 10.1080/87565641.2010.508670

Huson S. M., 1994, NEUROFIBROMATOSES CL

Hyman SL, 2007, J NEUROL NEUROSUR PS, V78, P1088, DOI 10.1136/jnnp.2006.108134
[truncated: 16,005,937 more chars]
